# Global, regional, and national burden of infertility attributable to polycystic ovary syndrome, 1990–2021: results from the Global Burden of Disease Study 2021

**DOI:** 10.3389/fpubh.2025.1693486

**Published:** 2025-11-10

**Authors:** Yufan Liang, Kexin Zhong, Zhuoyan Huang, Wenjing Shi, Yuhua Ou

**Affiliations:** 1 The Second Clinical Medical School, Guangzhou Medical University, Guangzhou, China; 2 Department of Gynecology, The Second Affiliated Hospital of Guangzhou Medical University, Guangzhou, China

**Keywords:** Global Burden of Disease, infertility, polycystic ovary syndrome, prevalence, years lived with disability

## Abstract

**Background:**

Analyzing the temporal trends, inequalities, and predictions of the infertility burden among women of childbearing age due to polycystic ovary syndrome.

**Methods:**

We employed a connection point regression analysis to carefully examine the temporal trends of PCOS-related infertility from 1990 to 2021. An age-period-cohort model was used to assess changes in prevalence across different ages, time periods, and birth cohorts. We also used Bayesian predictive models to forecast future burdens, conducted decomposition analyses to identify key drivers, and assessed health inequalities.

**Results:**

From 1990 to 2021, the global age-standardized prevalence rate of PCOS-related infertility increased from 475.54 to 638.15 per 100,000 population, and the years lived with disability (YLDs) increased from 2.77 to 3.67; the number of patients increased from 6.316 million to 12.467 million, with the growth rate of secondary infertility being higher than that of primary infertility. High SDI regions had a high base but showed a slowing growth trend, while middle SDI regions exhibited the fastest “catch-up” growth; East Asia and South Asia ranked among the top in annual growth rate. The global age peak was between 40 and 44 years, with the peak for primary infertility advancing to 20–24 years. It is projected that the number of patients will reach 22.43 million by 2050, of which secondary infertility will account for 82.8%. The absolute gap between high-SDI and low-SDI countries widened, while the relative gap narrowed; the driving factors were mainly Epidemic changes in high SDI regions and population growth in low SDI regions.

**Conclusion:**

PCOS-related infertility has evolved into a global public health crisis, requiring urgent implementation of precise prevention and control measures based on the life cycle and SDI gradient, as well as promotion of transnational technology sharing and data standardization to avoid its systemic erosion of the reproductive health of the next generation of women.

## Introduction

Infertility remains a critical global issue in reproductive health, with female factors contributing to approximately half of all cases. Clinically, infertility is defined as the inability to achieve pregnancy after 12 months of regular, unprotected sexual intercourse and is recognized as one of the most prevalent chronic conditions affecting women worldwide (1). According to the most recent report released by the World Health Organization in April 2023, the global prevalence of infertility is estimated at 17.5%, marking a substantial rise from the estimated 10% reported in 2016 (2). This upward trend underscores the growing significance of infertility as a major public health concern.

Multiple etiological factors contribute to female infertility, including ovulatory dysfunction, advanced maternal age (particularly over 35 years), and endocrine or metabolic disorders such as thyroid dysfunction, diabetes, obesity, and hypertension (3). Among these, polycystic ovary syndrome (PCOS) is the most prevalent cause of anovulatory infertility, accounting for up to 80% of such cases (4). PCOS is a complex endocrine condition characterized by a combination of hyperandrogenism (manifesting as hirsutism and acne) (5), chronic anovulation (including oligomenorrhea, infertility, and abnormal uterine bleeding) (6), and frequently has a genetic predisposition. It is considered the most common endocrine disorder among women of reproductive age, with a global prevalence ranging between 8% and 10% in premenopausal women (7).

The function of the hypothalamic–pituitary–ovarian axis is disrupted by elevated androgen levels in PCOS, resulting in ovulatory dysfunction (8). This disruption is typically accompanied by arrested follicular development and reduced oocyte quality (9), ultimately contributing to infertility. In addition to impairing fertility, PCOS-related infertility has been associated with increased rates of anxiety and depression. It is also frequently accompanied by sexual dysfunction and sexual distress, commonly manifested as reduced libido and dyspareunia, which adversely affect patients’ mental health, marital relationships, and overall well-being (10). Therefore, improving the understanding of the burden of PCOS-related infertility is essential for the primary prevention and management of infertility. It is recommended that screening and targeted interventions be strengthened in countries with a high disease burden.

The Global Burden of Disease (GBD) study is an international collaborative initiative designed to assess disease prevalence and temporal trends, to quantify the impact of various diseases, injuries, and risk factors on global health. Analyses of GBD data can reveal associations between demographic, geographic, and social determinants and PCOS-related infertility, potentially identifying novel etiologies and contributing risk factors. Although numerous studies have examined PCOS, research specifically addressing PCOS-related infertility remains relatively limited. To date, only two retrospective analyses using GBD 2019 data have been published (11, 12). These studies lack the latest burden changes in the post-pandemic phase and fail to conduct an in-depth analysis of the impact of risk factors such as environment and lifestyle. Furthermore, no published studies have modeled or predicted the future burden of PCOS-related infertility (13). Therefore, the present study employed the GBD 2021 dataset and medical data analysis to evaluate the global and regional burden of PCOS-related infertility using metrics including prevalent cases, years lived with disability (YLDs), age-standardized prevalence rate (ASPR), and age-standardized YLD rate (ASYR). Age-specific trends were examined, and future burdens (2022–2050) were projected to inform prevention strategies and healthcare planning for PCOS-related infertility, to improve women’s health outcomes.

## Materials and methods

### Data sources

This study is based on data from the Global Burden of Disease Study 2021 (GBD 2021), which systematically estimated the epidemiological burden of 371 diseases and injuries across 204 countries and regions from 1990 to 2021. Data were derived from multiple population-based surveys, including administrative records, disease registries, Demographic and Health Surveys (DHS), World Fertility Surveys (WFS), Reproductive Health Surveys (RHS), and Family and Fertility Surveys (FFS). These data are publicly accessible through the Institute for Health Metrics and Evaluation (IHME) Global Health Data Exchange.^1^

The Socio-demographic Index (SDI) developed by IHME measures socioeconomic development by synthesizing three indicators: total fertility rate among women under 25, average years of schooling for those aged 15 and older, and lagged per capita income distribution. The SDI ranges from 0 to 1, with higher values indicating greater development. Countries are categorized into five tiers: low, lower-middle, middle, upper-middle, and high.

Infertility is defined as a reproductive health disorder where a couple of reproductive age fails to conceive after at least 12 months of regular unprotected intercourse. According to the World Health Organization (WHO) and GBD classifications, it is categorized into primary and secondary infertility. Primary infertility refers to a woman who has never successfully conceived, often associated with ovulation disorders or congenital abnormalities; Secondary infertility occurs when a woman who has previously conceived or given birth fails to conceive again within 1 year, typically associated with infections or acquired reproductive dysfunction (14). This study follows the GBD 2021 classification system, incorporating both primary and secondary infertility to comprehensively assess the disease burden of polycystic ovary syndrome (PCOS)-related infertility.

PCOS diagnosis adheres to internationally accepted standards recommended by the American College of Obstetricians and Gynecologists (ACOG), including criteria from the National Institutes of Health (NIH), Rotterdam, and the Androgen Excess–PCOS Society (AE-PCOS). All three frameworks center diagnosis on chronic anovulation and hyperandrogenism, requiring exclusion of other secondary causes (12). The clinically most prevalent Rotterdam criteria require fulfillment of at least two of three components: ovulatory dysfunction, hyperandrogenism, and polycystic ovarian morphology. This study defines PCOS cases according to the ACOG framework and Rotterdam criteria.

Analyzed metrics included number of affected individuals, disability-adjusted life years (YLDs), age-standardized prevalence rate (ASPR), and age-standardized YLD rate (ASYR). All metrics were age-standardized using the GBD standard population to ensure temporal and spatial comparability. The 95% uncertainty interval (UI) was calculated using the 2.5th and 97.5th percentiles from 1,000 samples drawn from the model’s posterior distribution to quantify the range of estimation variability (15).

### Statistical analysis

To reveal temporal trends in the global burden of polycystic ovary syndrome (PCOS)-related infertility from 1990 to 2021, this study employed a joinpoint regression model (16). This model identifies significant turning points in the time series, dividing the overall trend into distinct phases. It calculates the annual percentage change (APC) for each phase and the overall average annual percentage change (AAPC). The model was constructed using log-linear regression and fitted with weighted least squares to fully utilize the standard errors provided by GBD data, enhancing the robustness of estimates. The optimal number of joinpoints (0–5) was determined via Monte Carlo permutation testing, with a significance level set at *α* < 0.05 and Bonferroni correction applied to control for multiple comparisons.

To further analyze the independent effects of age, time, and birth cohort on disease burden, an Age–Period–Cohort (APC) model (17) was employed. The model simultaneously estimated overall temporal trends (net drift) and age-specific trends (local drift) for each age group, with statistical significance assessed via Wald *χ*^2^ tests. Longitudinal age curves, period-specific relative risks (RR), and cohort-specific RR were used to characterize prevalence patterns across age, period, and cohort. Analysis employed 5-year age groups paired with 5-year time periods, yielding seven age cohorts (15–49 years) and six periods (1992–2021), forming 12 partially overlapping 10-year birth cohorts (1942–1951 to 1997–2006).

To forecast future trends, this study further applied a Bayesian Age–Period–Cohort (BAPC) model, performing inference within an Integrated Nested Laplace Approximation (INLA) framework (18). The model employs a log-linear structure based on the Poisson distribution, incorporating second-order random walk (RW2) and independent random effects to smooth temporal fluctuations. Predictions, integrated with World Health Organization (WHO) population projections, estimate the number of individuals affected by PCOS-related infertility and age-standardized prevalence rates for 2022–2050.

To identify drivers of disease burden changes, the Das Gupta decomposition method was applied to separate contributions from population growth, aging, and epidemiological factors between 1990 and 2021, quantifying their relative impacts on overall burden shifts.

Furthermore, to assess the inequity in disease burden distribution across countries with varying socioeconomic development levels, health inequity analysis was conducted using the Slope Inequality Index (SII) and the Concentration Index (CI). The SII estimates the absolute difference in disease burden between the country with the lowest and highest Socio-Demographic Index (SDI) through robust weighted regression (rlm). The CI, calculated based on the Lorenz concentration curve, reflects the relative concentration of disease burden across different SDI distributions (19).

All statistical analyses and visualizations were performed using R software (version 4.5.1). Detailed data sources and statistical methodologies are described in Supplementary materials.

## Results

### Prevalence and changes in years lived with disability of infertility caused by polycystic ovarian syndrome among women of reproductive age

**Figure 1:**
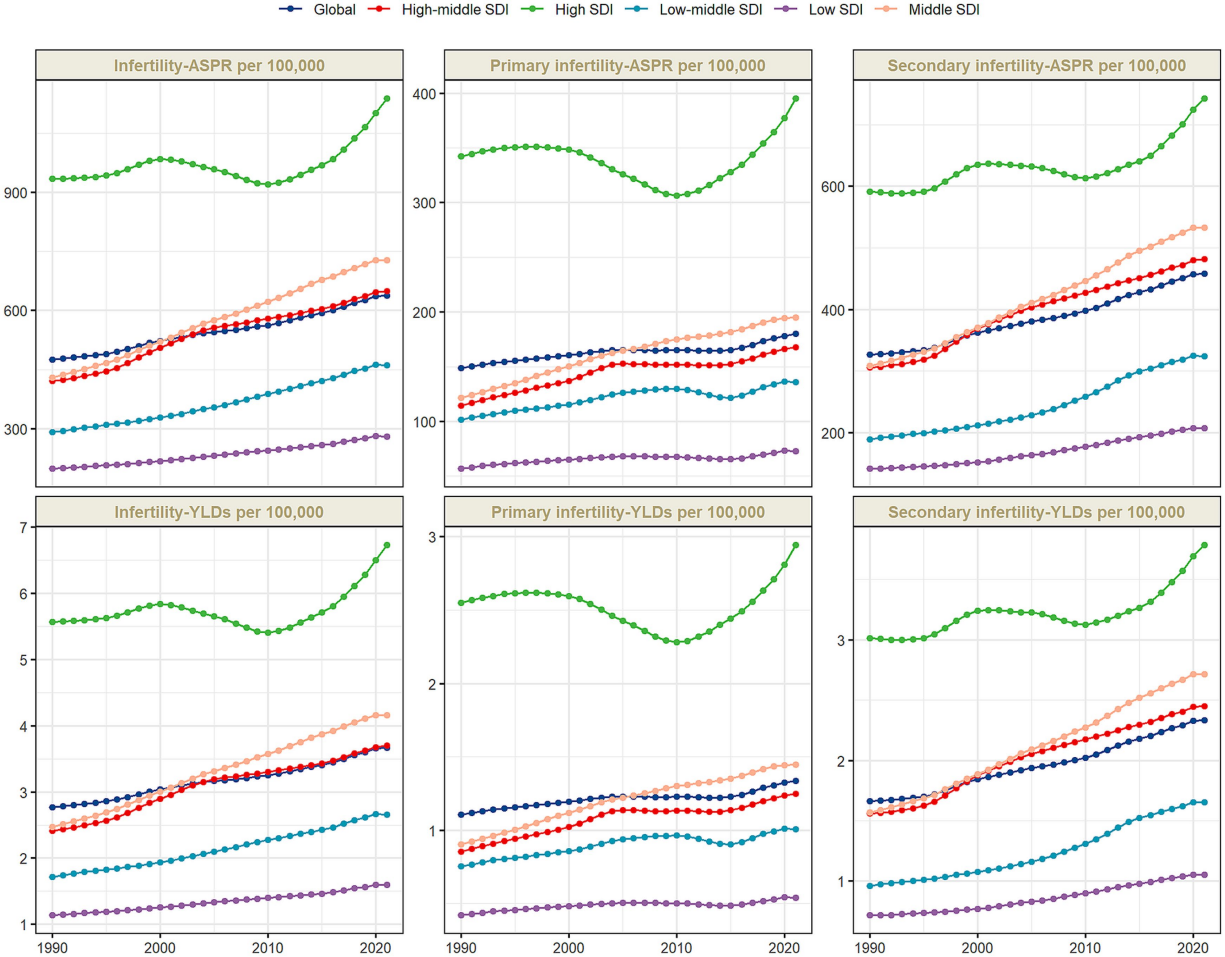
The age-standardized prevalence rate (ASPR), and the age-standardized YLD rate (ASYR) of infertility attributable to polycystic ovary syndrome among women of reproductive age (15–49 years) by SDI, 1990–2021.

From 1990 to 2021, the ASPR of infertility caused by Polycystic ovarian syndrome increased from 475.54 per 100,000 (95% UI: 293.03–725.57) to 638.15 per 100,000 (95% UI: 388.26–982.60), with an AAPC of 0.96% (95% CI: 0.95–0.98); meanwhile, the ASYR increased from 2.77 (95% UI: 1.05–6.26) per 100,000 to 3.67 per 100,000 (95% UI: 1.36–8.33), with an AAPC of 0.92% (0.90–0.93) (Supplementary Table S1). In terms of the absolute number of cases, the global total increased from 6,315,531 (95% UI: 3,908,231–9,583,536) to 12,465,534 (95% UI: 7,573,847–19,225,837) over the 30 years (Table 1). When subdivided by infertility type, primary infertility corresponded to ASPR [AAPC: 0.62% (95% CI: 0.60–0.63)] and ASYR [AAPC: 0.61% (95% CI: 0.60–0.62)] (Supplementary Table S2; Figure 1). Secondary infertility ASPR [AAPC: 1.12% (95%CI: 1.10–1.13)], ASYR [AAPC: 1.12% (95%CI: 1.10–1.13)] (Supplementary Table S2; Figure 1).

**Table 1:** The prevalence and YLDs of infertility (primary infertility and secondary infertility) due to PCOS at the global and regional levels in 1990 and 2021.

Location	Infertility				Primary infertility				Secondary infertility			
	Prevalence		YLDs		Prevalence		YLDs		Prevalence		YLDs	
	No. (95% UI)		No. (95% UI)		No. (95% UI)		No. (95% UI)		No. (95% UI)		No. (95% UI)	
	1990	2021	1990	2021	1990	2021	1990	2021	1990	2021	1990	2021
Global	6,315,531 (3,908,231, 9,583,536)	12,465,534 (7,573,847, 19,225,837)	37,030 (14,137, 83,610)	71,555 (26,407, 162,585)	2,074,117 (766,046, 4,112,151)	3,452,839 (1,013,714, 7,702,491)	15,435 (4,499, 38,980)	25,668 (6,143, 69,096)	4,241,414 (2,075,989, 7,313,059)	9,012,694 (4,331,151, 15,261,346)	21,595 (6,895, 53,071)	45,887 (14,427, 114,832)
Socio-demographic index												
High SDI	2,189,557 (1,276,601, 3,450,404)	2,841,407 (1,684,955, 4,422,844)	13,012 (4,680, 29,047)	16,633 (6,008, 37,428)	785,608 (284,271, 1,603,321)	914,345 (274,348, 1,951,881)	5,856 (1,628, 14,617)	6,811 (1,620, 17,961)	1,403,949 (598,924, 2,569,972)	1,927,062 (812,634, 3,426,723)	7,156 (2092, 18,158)	9,822 (2,903, 24,794)
High-middle SDI	1,176,367 (721,018, 1,786,823)	1,992,443 (1,174,463, 3,156,668)	6,768 (2,554, 15,669)	11,212 (4,069, 25,842)	328,189 (112,156, 690,783)	453,476 (124,303, 1,088,422)	2,448 (653, 6,555)	3,378 (714, 9,453)	848,178 (435,647, 1,419,497)	1,538,967 (762,541, 2,608,901)	4,320 (1,410, 10,495)	7,834 (2,455, 19,725)
Middle SDI	1,926,915 (1,170,971, 2,942,268)	4,503,200 (2,684,593, 7,063,720)	11,180 (4,145, 25,340)	25,635 (9,259, 59,126)	587,706 (198,936, 1,231,665)	1,158,802 (301,480, 2,708,374)	4,365 (1,152, 11,444)	8,609 (1859, 24,107)	1,339,209 (664,152, 2,277,490)	3,344,398 (1,612,758, 5,698,513)	6,815 (2,181, 17,078)	17,026 (5,259, 42,581)
Low-middle SDI	794,720 (491,363, 1,254,633)	2,342,928 (1,424,007, 3,692,986)	4,743 (1776, 10,713)	13,570 (4,996, 31,194)	300,467 (126,845, 575,644)	706,146 (210,537, 1,561,528)	2,231 (715, 5,583)	5,239 (1,262, 14,130)	494,253 (247,341, 861,094)	1,636,782 (770,523, 2,823,558)	2,512 (798, 6,253)	8,331 (2,579, 20,875)
Low SDI	223,480 (136,656, 359,250)	776,734 (476,985, 1,230,592)	1,300 (483, 2,978)	4,454 (1,647, 10,176)	70,553 (25,458, 144,915)	217,451 (70,556, 466,113)	523 (141, 1,352)	1,612 (399, 4,278)	152,927 (76,653, 263,985)	559,283 (287,910, 947,488)	776 (245, 1929)	2,842 (910, 7,147)
Region												
Andean Latin America	54,275 (33,157, 82,307)	176,064 (82,248, 296,137)	308 (112, 703)	977 (305, 2,351)	13,209 (6,142, 24,201)	33,934 (7,825, 83,984)	99 (29, 244)	823 (155, 2,483)	41,067 (23,396, 65,468)	142,130 (51,487, 261,104)	209 (71, 488)	724 (190, 1889)
Australasia	52,125 (19,272, 100,895)	81,323 (30,709, 155,563)	307 (79, 769)	475 (125, 1,191)	17,245 (4,699, 42,093)	25,157 (6,976, 60,401)	128 (27, 368)	3,009 (696, 8,017)	34,880 (9,286, 77,500)	56,166 (15,435, 123,148)	179 (34, 527)	287 (57, 826)
Caribbean	42,476 (24,927, 67,296)	66,560 (39,007, 107,609)	252 (91, 579)	384 (136, 902)	15,156 (5,372, 31,266)	19,530 (5,445, 44,351)	113 (32, 297)	3,336 (800, 8,760)	27,320 (13,203, 48,565)	47,030 (23,261, 83,377)	139 (43, 359)	239 (76, 597)
Central Asia	23,271 (13,569, 37,862)	45,180 (26,357, 72,733)	134 (46, 314)	256 (89, 610)	6,792 (1813, 15,436)	11,282 (2,732, 27,855)	51 (10, 142)	188 (38, 536)	16,479 (7,754, 29,297)	33,898 (16,353, 59,232)	84 (25, 213)	172 (52, 434)
Central Europe	22,000 (12,316, 37,168)	21,791 (12,680, 35,187)	124 (42, 298)	121 (41, 287)	5,293 (982, 13,885)	4,374 (814, 11,650)	39 (6, 122)	253 (45, 766)	16,707 (7,686, 30,621)	17,418 (8,707, 30,035)	85 (25, 219)	88 (27, 221)
Central Latin America	351,048 (205,695, 560,601)	689,094 (414,345, 1,087,081)	2030 (727, 4,784)	3,873 (1,440, 9,013)	101,933 (33,258, 225,042)	155,551 (46,187, 367,938)	760 (194, 2,107)	325 (63, 926)	249,114 (116,649, 441,560)	533,543 (270,085, 915,166)	1,270 (384, 3,197)	2,715 (896, 6,676)
Central Sub-Saharan Africa	22,185 (13,144, 36,632)	88,814 (51,731, 144,997)	127 (43, 302)	505 (177, 1,171)	5,894 (1,318, 14,031)	23,087 (4,198, 56,551)	44 (6, 130)	1,157 (264, 3,326)	16,291 (7,608, 28,982)	65,727 (29,991, 117,526)	83 (24, 216)	334 (93, 867)
East Asia	1,131,880 (655,534, 1,822,438)	1,993,543 (1,128,348, 3,292,108)	6,153 (2076, 14,319)	10,765 (3,663, 25,561)	168,137 (23,397, 476,438)	267,149 (44,309, 762,200)	1,247 (146, 4,048)	297 (68, 820)	963,743 (507,485, 1,617,327)	1,726,394 (901,510, 2,957,751)	4,906 (1,575, 12,294)	8,781 (2,743, 22,044)
Eastern Europe	45,925 (26,425, 75,347)	50,457 (29,253, 82,441)	268 (95, 621)	289 (100, 676)	14,473 (3,235, 34,312)	13,856 (2,871, 34,223)	108 (20, 306)	145 (33, 394)	31,453 (14,226, 57,739)	36,601 (17,137, 66,022)	160 (47, 418)	186 (55, 478)
Eastern Sub-Saharan Africa	89,321 (54,468, 142,871)	284,221 (173,294, 453,513)	511 (189, 1,160)	1,606 (584, 3,686)	24,485 (9,288, 49,265)	69,039 (20,616, 152,995)	182 (52, 467)	33 (5, 104)	64,836 (34,731, 109,635)	215,182 (114,247, 363,248)	329 (108, 795)	1,094 (349, 2,717)
High-income Asia Pacific	609,860 (277,211, 1,060,201)	555,398 (246,047, 969,144)	3,434 (1,021, 8,254)	3,087 (903, 7,490)	139,217 (33,955, 351,777)	110,671 (26,556, 283,057)	1,037 (205, 3,121)	103 (17, 305)	470,643 (178,243, 884,736)	444,727 (164,912, 832,550)	2,398 (621, 6,263)	2,264 (588, 5,958)
High-income North America	731,694 (394,701, 1,211,634)	1,053,154 (617,322, 1,643,169)	4,471 (1,524, 9,988)	6,315 (2,258, 14,019)	314,095 (87,909, 692,315)	404,222 (114,516, 887,976)	2,340 (533, 6,110)	84 (14, 244)	417,598 (127,680, 850,680)	648,932 (236,858, 1,225,508)	2,131 (493, 5,844)	3,306 (856, 8,552)
North Africa and Middle East	490,982 (304,402, 763,664)	1,326,036 (789,807, 2,112,035)	3,039 (1,168, 6,896)	7,844 (2,874, 18,023)	229,626 (115,965, 400,804)	462,718 (142,950, 1,008,588)	1711 (607, 3,957)	3,442 (842, 8,996)	261,356 (129,856, 457,721)	863,318 (364,645, 1,560,892)	1,328 (426, 3,294)	4,401 (1,258, 11,409)
Oceania	7,433 (4,294, 12,462)	22,379 (13,257, 36,260)	43 (15, 99)	126 (45, 296)	2038 (428, 5,132)	5,333 (2037, 10,694)	15 (2, 45)	4,881 (1,079, 13,581)	5,395 (2,317, 9,910)	17,046 (9,630, 28,570)	27 (8, 72)	87 (30, 212)
South Asia	626,521 (390,835, 974,710)	2,198,417 (1,355,099, 3,436,096)	3,756 (1,415, 8,543)	12,709 (4,691, 29,199)	245,680 (82,719, 495,367)	658,771 (172,470, 1,498,775)	1821 (486, 4,710)	4,051 (927, 11,507)	380,841 (162,243, 693,368)	1,539,646 (699,665, 2,655,515)	1935 (555, 5,004)	7,828 (2,356, 19,968)
Southeast Asia	701,271 (413,590, 1,124,731)	1,909,729 (1,133,945, 3,067,369)	4,144 (1,462, 9,398)	11,001 (3,931, 25,438)	247,068 (84,156, 526,342)	545,322 (150,423, 1,287,882)	1834 (482, 4,796)	1984 (267, 6,589)	454,202 (215,550, 796,597)	1,364,407 (641,747, 2,357,364)	2,310 (706, 5,784)	6,950 (2,128, 17,465)
Southern Latin America	52,414 (30,487, 88,729)	117,138 (67,883, 195,748)	310 (107, 724)	690 (241, 1,600)	18,691 (5,240, 44,154)	39,968 (11,005, 93,061)	139 (30, 381)	40 (11, 104)	33,723 (13,259, 64,584)	77,169 (31,224, 145,849)	172 (44, 451)	393 (105, 1,024)
Southern Sub-Saharan Africa	44,921 (26,010, 72,056)	88,848 (51,545, 146,740)	261 (92, 600)	509 (175, 1,184)	13,836 (3,597, 31,184)	24,321 (5,026, 61,718)	102 (21, 282)	653 (132, 1830)	31,085 (14,663, 54,794)	64,527 (28,796, 114,929)	158 (49, 406)	328 (96, 852)
Tropical Latin America	83,636 (46,972, 137,265)	135,259 (77,582, 218,651)	504 (174, 1,155)	792 (281, 1837)	33,654 (8,258, 75,703)	43,751 (10,543, 104,531)	249 (51, 678)	511 (122, 1,404)	49,982 (19,067, 98,269)	91,507 (38,379, 170,183)	255 (66, 690)	467 (132, 1,235)
Western Europe	1,044,990 (638,046, 1,621,549)	1,218,181 (703,901, 1,936,970)	6,359 (2,395, 14,530)	7,277 (2,602, 16,763)	434,399 (184,264, 836,336)	446,756 (131,066, 967,890)	3,245 (1,015, 7,854)	171 (25, 496)	610,592 (281,665, 1,086,313)	771,424 (309,542, 1,394,028)	3,114 (941, 7,900)	3,941 (1,114, 10,196)
Western Sub-Saharan Africa	87,303 (53,106, 140,786)	343,948 (208,615, 553,574)	497 (181, 1,137)	1954 (699, 4,480)	23,196 (8,378, 47,607)	88,045 (22,579, 204,566)	172 (47, 437)	180 (31, 535)	64,107 (33,367, 108,951)	255,902 (125,754, 445,553)	326 (105, 809)	1,301 (405, 3,230)

YLDs, years lived with disability; UI, uncertainty interval.

**Figure 2:**
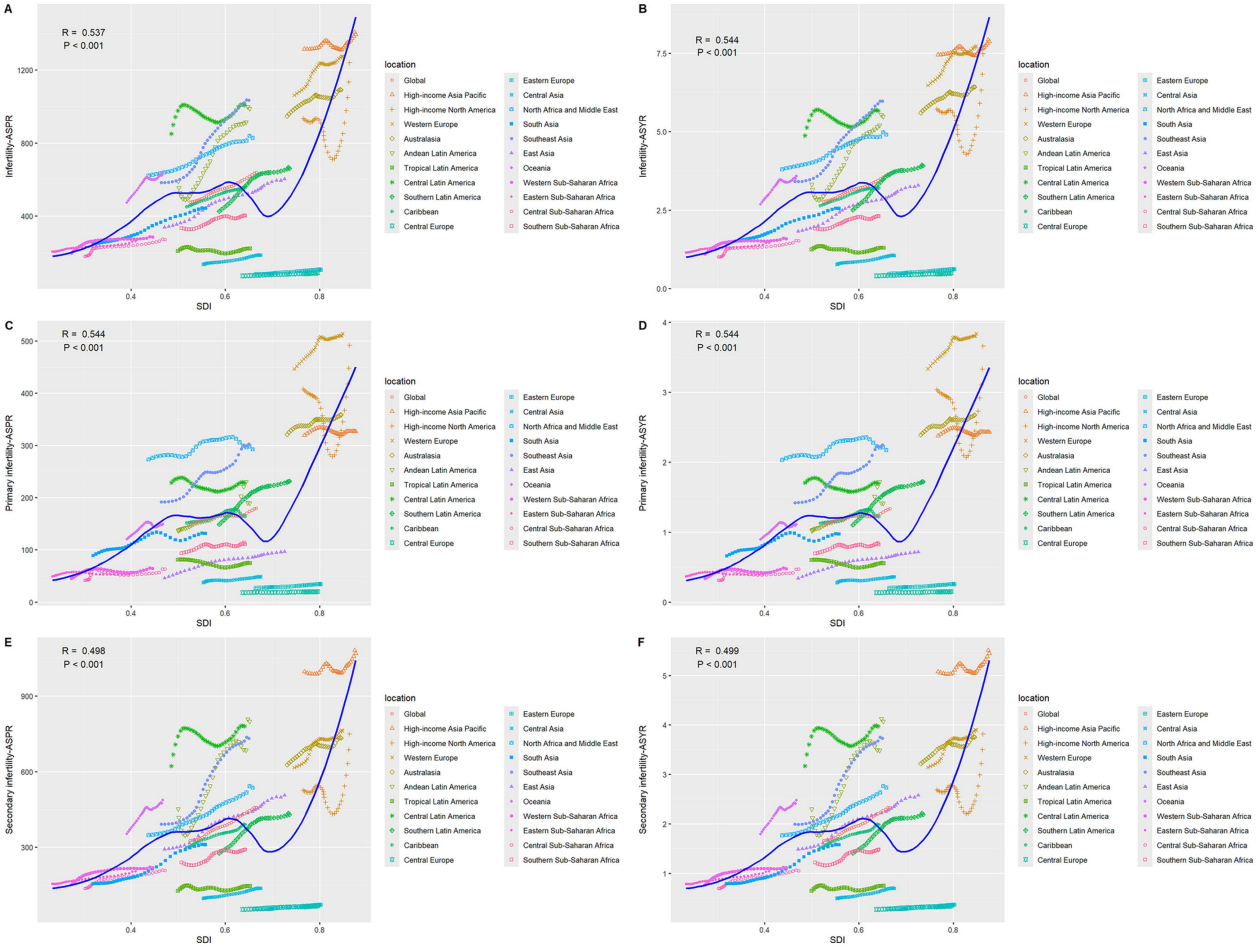
The correlation between age-standardized prevalence and YLDs rate and SDI for GBD regions for infertility attributable to polycystic ovarian syndrome from 1990 to 2021. **(A)** ASPR of infertility. **(B)** ASYR of infertility. **(C)** ASPR of primary infertility. **(D)** ASYR of primary infertility. **(E)** ASPR of secondary infertility. **(F)** ASYR of secondary infertility.

The disease burden shows a significant stratification feature with the Socio-Demographic Index (SDI). In 2021, high-SDI regions had the highest ASPR and ASYR globally, which were 1,137.85 per 100,000 (95% UI: 673.39–1771.12) and 6.73 per 100,000 (95% UI: 2.42–15.14), respectively, but with the slowest growth rates, with AAPCs of 0.63% (95%CI: 0.62–0.65) and 0.60% (95%CI: 0.58–0.62) respectively (Supplementary Table S1; Figure 1). In middle-SDI regions, ASPR increased from 429.86 per 100,000 (95% UI: 260.36–660.04) to 727.72 per 100,000 (95% UI: 434.04–1138.92) [AAPC: 1.72% (95%CI: 1.72–1.74)]; ASYR increased from 2.47 per 100,000 (95% UI: 0.91–5.62) to 4.16 per 100,000 (95% UI: 1.50–9.59) [AAPC: 1.70% (95%CI: 1.68–1.72)] (Supplementary Table S1). Although low-SDI regions had the lowest baseline, with ASPR of 280.24 per 100,000 (95% UI: 170.95–444.96) and ASYR (95% UI: 0.58–3.65) of 1.59 per 100,000 in 2021, the AAPCs of ASPR and ASYR from 1990 to 2021 reached 1.14% (95%CI: 1.13–1.16) and 1.11% (95%CI: 1.09–1.12), respectively, showing an obvious catch-up growth (Supplementary Table S1; Figure 1). Studies have shown (Figure 2) that ASPR (*R* = 0.537, 0.544, 0.498, *p* < 0.001) and ASYR (*R* = 0.544, 0.544, 0.499, *p* < 0.001) for infertility caused by PCOS, primary infertility, and secondary infertility are significantly positively correlated with SDI.

Supplementary Table S5 and Supplementary Figure S1 show that the prevalence of PCOS-related infertility has a unimodal distribution with age: globally, it peaks at 40–44 years old and then drops sharply; in high SDI regions, the peak is the highest and shifts to the right, rising to 1,363 per 100,000 (95% UI: 823–2055) at 35–39 years old and remaining at 1334 per 100,000 (95% UI: 808–2002) at 40–44 years old; in low SDI regions, the peak is only 304 per 100,000 (95% UI: 181–478) and declines earlier. The peak of primary infertility occurs at 20–24 years old, then decreases with age, reaching only 43.2 per 100,000 (95% UI: 24–74) at 45–49 years old. The peak of secondary infertility is more to the right, reaching 659 per 100,000 (95% UI: 388–1,024) globally at 40–44 years old, 1,142 per 100,000 (95% UI: 690–1761) in high SDI regions in the same age group, and only 260 per 100,000 (95% UI: 154–410) in low SDI regions. Overall, the age group of 25–44 years is the period with concentrated risk, and the peak height increases with the SDI.

**Figure 3:**
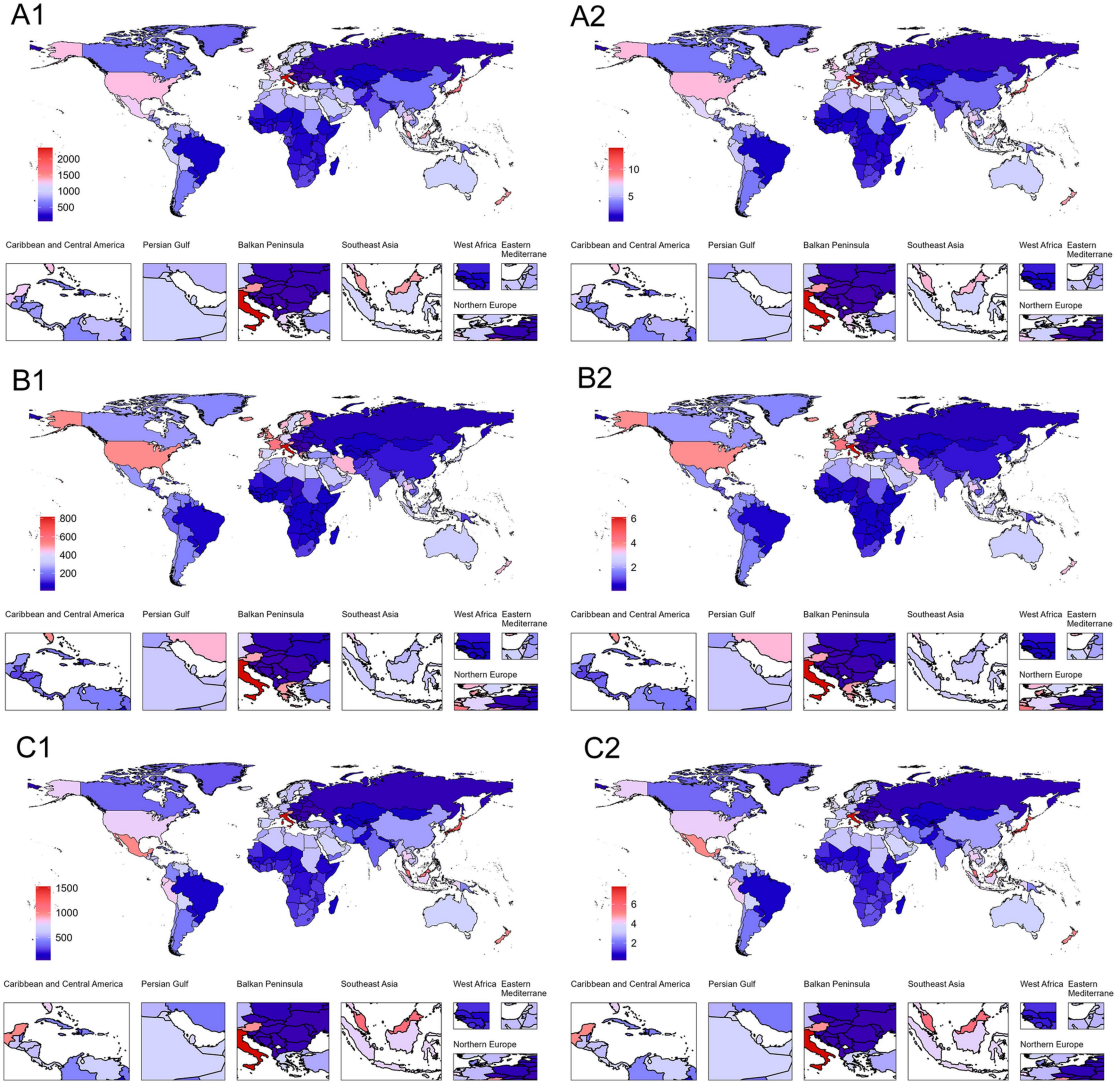
Global distribution of ASPR and ASYR for PCOS-related infertility in 2021. **(A1)** Infertility (ASPR). **(B1)** Primary infertility (ASPR). **(C1)** Secondary infertility (ASPR). **(A2)** Infertility (ASYR). **(B2)** Primary infertility (ASYR). **(C2)** Secondary infertility (ASYR). Blue represents the lowest values, and red indicates the highest values. The red-to-blue gradient effectively visualizes.

The geographical distribution exhibits extreme disparities (Figure 3). From a regional perspective, East Asia and South Asia lead with AAPCs of 1.89% (95% CI: 1.87–1.91) and 1.93% (95% CI: 1.91–1.95), respectively. In contrast, tropical Latin America shows nearly stagnant growth, with an AAPC of only 0.15% (95% CI: 0.1–0.19), the lowest among all analyzed regions. Andean Latin America and Southeast Asia both demonstrate relatively high growth rates, with AAPCs of 1.77% (95% CI: 1.63–1.88) and 1.88% (95% CI: 1.86–1.91), reflecting the escalating health burden related to PCOS within these regions. Notably, high-income Asia Pacific [AAPC: 0.21% (95% CI: 0.19–0.23)], Western Europe [AAPC: 0.60% (95% CI: 0.59–0.61)], and North America [AAPC: 0.93% (95% CI: 0.87–0.99)] exhibit relatively slow growth despite their higher baseline values (Supplementary Table S2).

Between 1990 and 2021, there were significant disparities in ASPR and ASYR levels attributable to infertility caused by PCOS among different countries (Figure 3). Austria had the highest ASPR value, with its overall ASPR reaching 1447.20 per 100,000 population (95% UI: 833.58–2335.49) in 2021, far exceeding other countries, while Bosnia and Herzegovina recorded the lowest ASPR at only 72.54 per 100,000 population (95% UI: 39.31–124.77). In terms of AAPC, Equatorial Guinea showed the fastest growth rate, with an AAPC for ASPR of 2.77% (95% CI: 2.73–2.83). In contrast, countries with the slowest growth or even negative trends, such as Brazil, had an AAPC for ASPR of merely 0.12% (95% CI: 0.07–0.16). Additionally, China experienced a significant rise in ASPR during this period, increasing from 333.44 per 100,000 population (95% UI: 193.80–547.28) to 601.73 per 100,000 population (95% UI: 343.11–988.13), with an AAPC of 1.93% (95% CI: 1.90–1.95), placing it among the countries with relatively rapid growth rates (Supplementary Table S4).

### Age-period-cohort model

**Figure 4:**
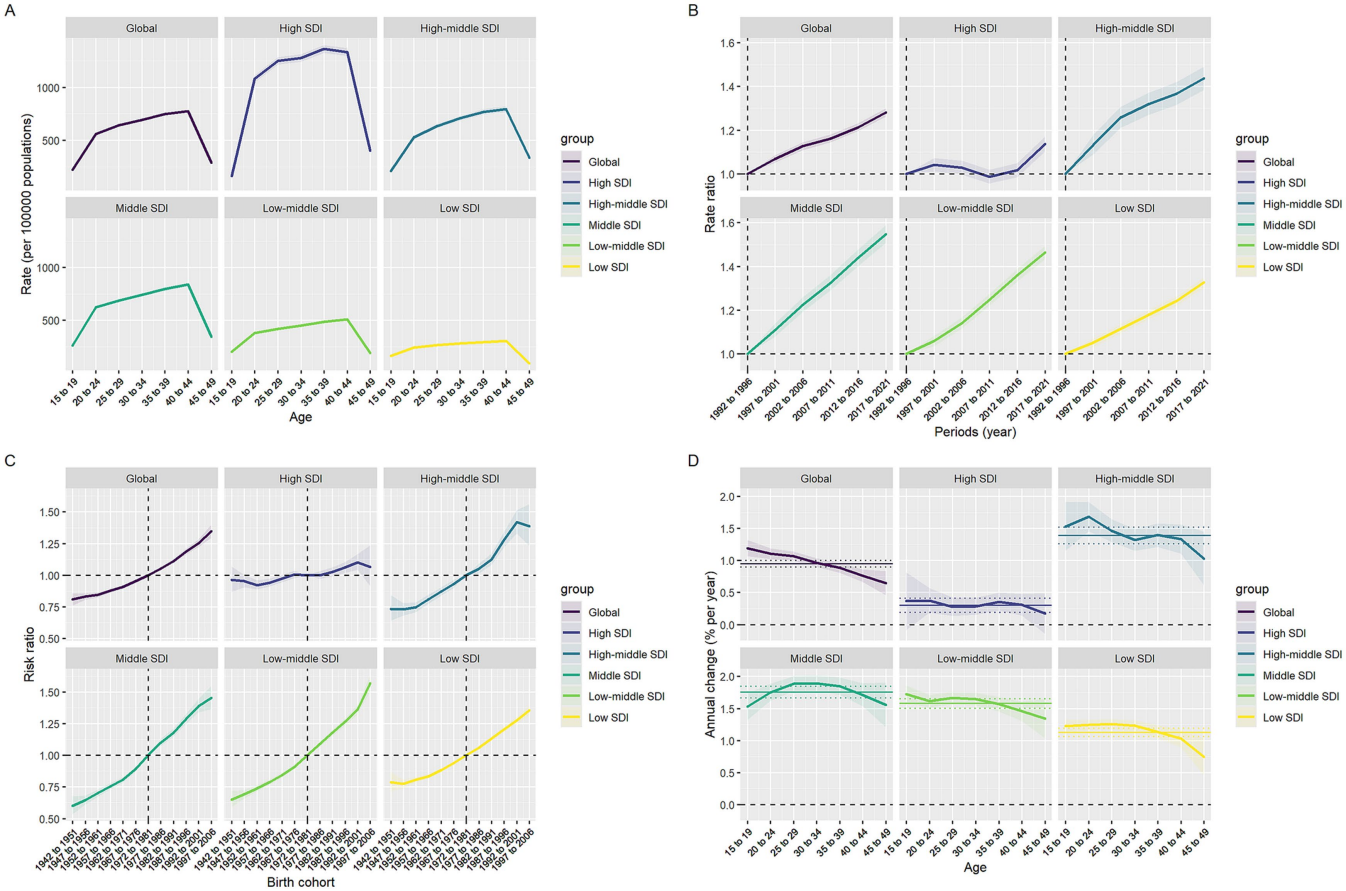
Effects of age, period, and birth cohort on infertility-associated polycystic ovary syndrome prevalence across SDI quintiles. **(A)** Age effects are represented by fitted longitudinal age-specific rates. **(B)** Period effects are demonstrated through relative period risks. **(C)** Cohort effects are displayed using relative cohort risks. **(D)** Annual percentage changes are shown for each age-specific group through yearly variations.

The age-period-cohort model system delineates the global trends of PCOS-related primary and secondary infertility from 1990 to 2021, revealing heterogeneity in the effects of the three temporal dimensions (Supplementary Tables S5–S8, Figure 2, and Supplementary Figures S2, S3). In the age dimension (Supplementary Table S5), the prevalence rates of both primary and secondary infertility followed an inverted U-shaped curve, initially rising and then declining, though with distinct peak ages and magnitudes. Globally, secondary infertility peaked at ages 35–39, while primary infertility peaked earlier at ages 20–24. In high-SDI countries, both types of infertility exceeded 1,100 per 100,000 at ages 35–39, whereas in low-SDI countries, the peak rates were only 260–390 per 100,000. In the period dimension, the relative risks across all SDI levels showed a continuous increase from 1992 to 2021, though the rate of increase varied by type and resource level. Globally, the relative risk of secondary infertility increased from 1.00 (reference) to 1.37 (95% CI: 1.33–1.40), while that of primary infertility only rose to 1.13 (95% CI: 1.07–1.19). From the SDI perspective, the relative risk of secondary infertility in low-middle SDI countries reached 1.61 (95% CI: 1.52–1.70) in 2021, significantly higher than the 1.14 (95% CI: 1.11–1.18) in high SDI countries; primary infertility also showed a similar gradient, but with smaller increases than secondary infertility (see Figure 4).

The cohort effect (Supplementary Table S7) further indicated that women born later faced higher risks, with a pronounced socioeconomic gradient. Using the 1972–1981 birth cohort as reference, the RR of secondary infertility globally rose to 1.61 (95% CI: 1.49–1.74) for those born in 1997–2006, while primary infertility only reached 1.10 (95% CI: 1.00–1.22). Among low-middle SDI countries, the secondary infertility RR for births during 1997–2006 reached 2.24 (95% CI: 1.99–2.53), while the primary infertility RR was 1.30 (95% CI: 1.23–1.38). In high SDI countries, the corresponding values were only 1.04 (95% CI: 0.91–1.12) and 1.02 (95% CI: 0.97–1.08), respectively. Local drift analysis (Supplementary Table S8) revealed that the increase in secondary infertility consistently exceeded that of primary infertility within the same age groups, with a declining trend as age advanced. Globally, secondary infertility reached 1.91%/year (95% CI: 1.65–2.16) as early as 15–19 years, while primary infertility was only 0.42%. By 45–49 years, these rates decreased to 0.65 and 0.36%, respectively. When stratified by SDI, low- to middle-income SDI countries exhibited the most rapid growth—secondary infertility in the 15–19 age group increased to 3.17%/year (95% CI: 2.75–3.59), with primary infertility also reaching 0.40%. In contrast, high SDI countries recorded only 0.26 and 0.53% for the same age group, with primary infertility showing negative drift (−0.34% to −0.57%) after age 30. Countries with middle-high and middle SDI levels fall between the two, but overall still maintain a sustained annual increase of 1–2%.

### BAPC model projection

**Figure 5:**
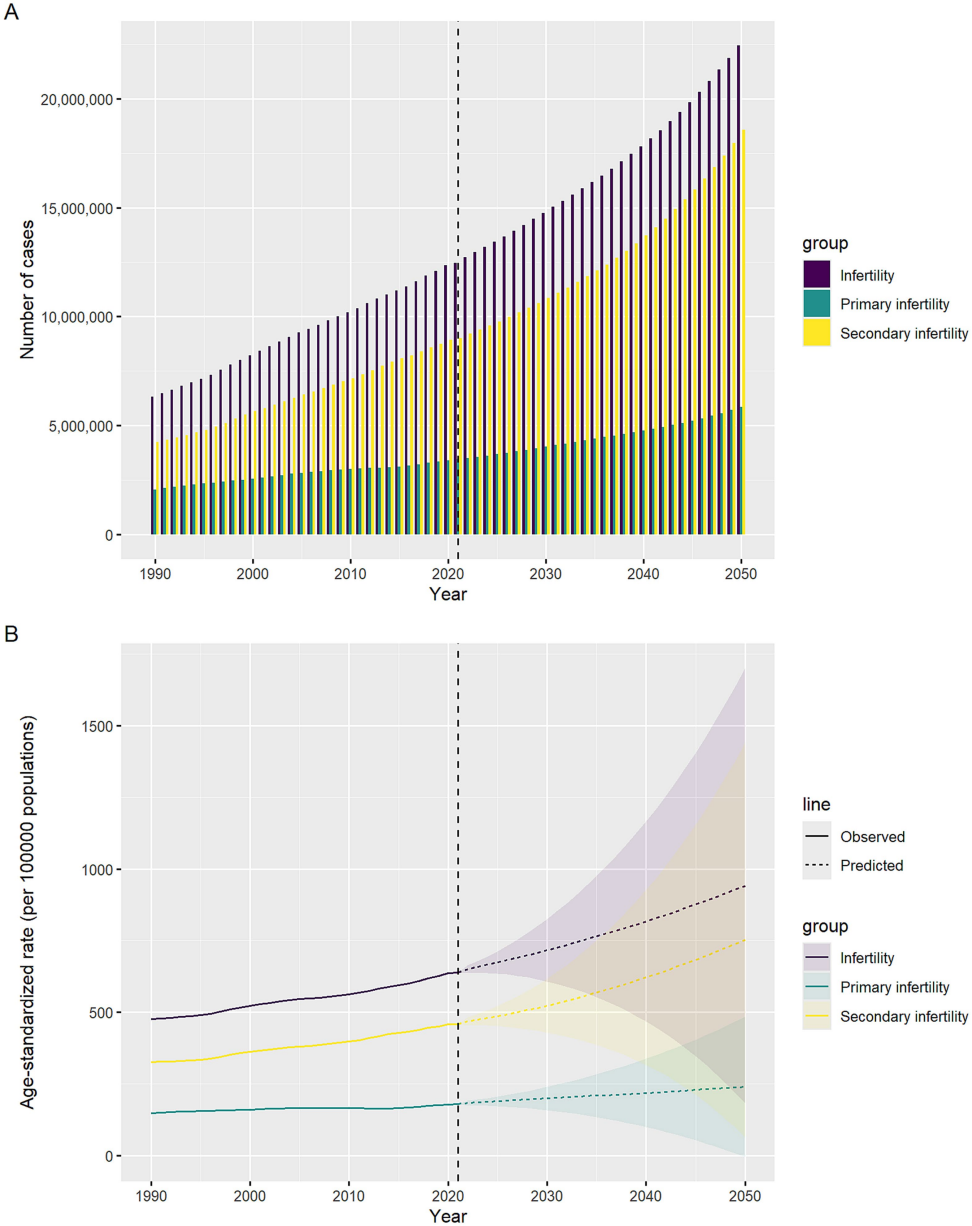
Temporal trends in infertility cases and ASPR attributed to polycystic ovary syndrome, 1990–2050. The horizontal axis indicates years, while the vertical axis shows either case counts or ASPR. **(A)** Case counts. **(B)** ASPR.

Prospective simulations based on the BAPC framework indicate that the disease burden of PCOS-related infertility will continue to expand by 2050. The ASPR of total infertility is projected to further rise to 942 per 100,000 (95% CI: 182–1702) by 2050, corresponding to a total of 22.43 million cases (95% CI: 3.02–42.02 million). The ASPR of primary infertility is expected to reach 240 per 100,000 (95% CI: −5.1–484) by 2050, with the number of cases potentially reaching 5.86 million (95% CI: 0.3–12.80 million). The burden of secondary infertility is growing even more rapidly, with its ASPR projected to reach 752 per 100,000 (95% CI: 63–1,441) by 2050, and the number of cases potentially rising to 18.57 million (95% CI: 0.56–37.40 million). Figure 5 visually demonstrates the exponential upward trend in the burden of these three disease categories from 1990 to 2050, with the curve for secondary infertility being the steepest.

**Figure 6:**
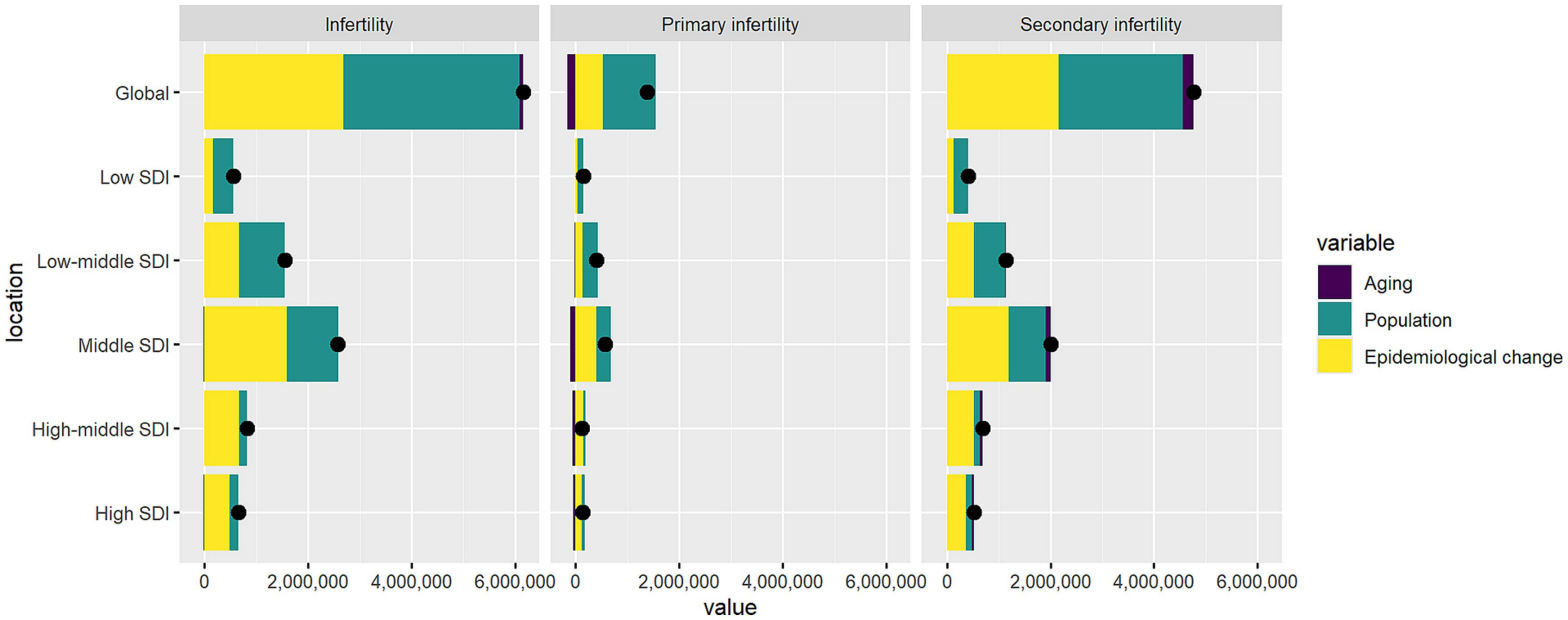
The impact of demographic ageing, population growth, and epidemiological transitions on infertility prevalence attributable to endometriosis globally and across SDI regions, 1990–2021. Black dots indicate the net total change in disease burden, consistent with the sum of the two components.

### Decomposition analysis

Through decomposition analysis, we partitioned the net changes in the burden of PCOS-related infertility from 1990 to 2021 into the independent contributions of population aging, population growth, and epidemiological changes (Figure 6; Supplementary Table S11). Globally, the total number of infertility cases increased by 6.15 million, with population growth contributing 55.3%, epidemiological changes accounting for 43.7%, and population aging contributing only 0.95%.

When further classified, primary infertility increased by 1.38 million cases, primarily driven by population growth (73.9%), while epidemiological changes (–11.9%) and aging (–0.5%) showed negative effects. In contrast, secondary infertility increased by 4.77 million cases, with population growth and epidemiological changes contributing 50.0% and 45.4%, respectively, and aging accounting for 4.7%.

Across the SDI spectrum, the expansion of the disease burden in high- and high-middle-SDI regions was mainly driven by rising prevalence, with epidemiological changes contributing 74.3% and 81.8%, respectively. Conversely, in low- and low-middle-SDI regions, population growth played a dominant role (71.0% and 56.8%), while aging contributed minimally (–0.1%). Middle-SDI regions represented a transitional pattern, with epidemiological changes accounting for 61.8%, population growth 38.6%, and aging showing a negative effect (–0.4%). Overall, population aging exerted a limited direct influence on the global scale.

### Analysis of health inequities

**Figure 7:**
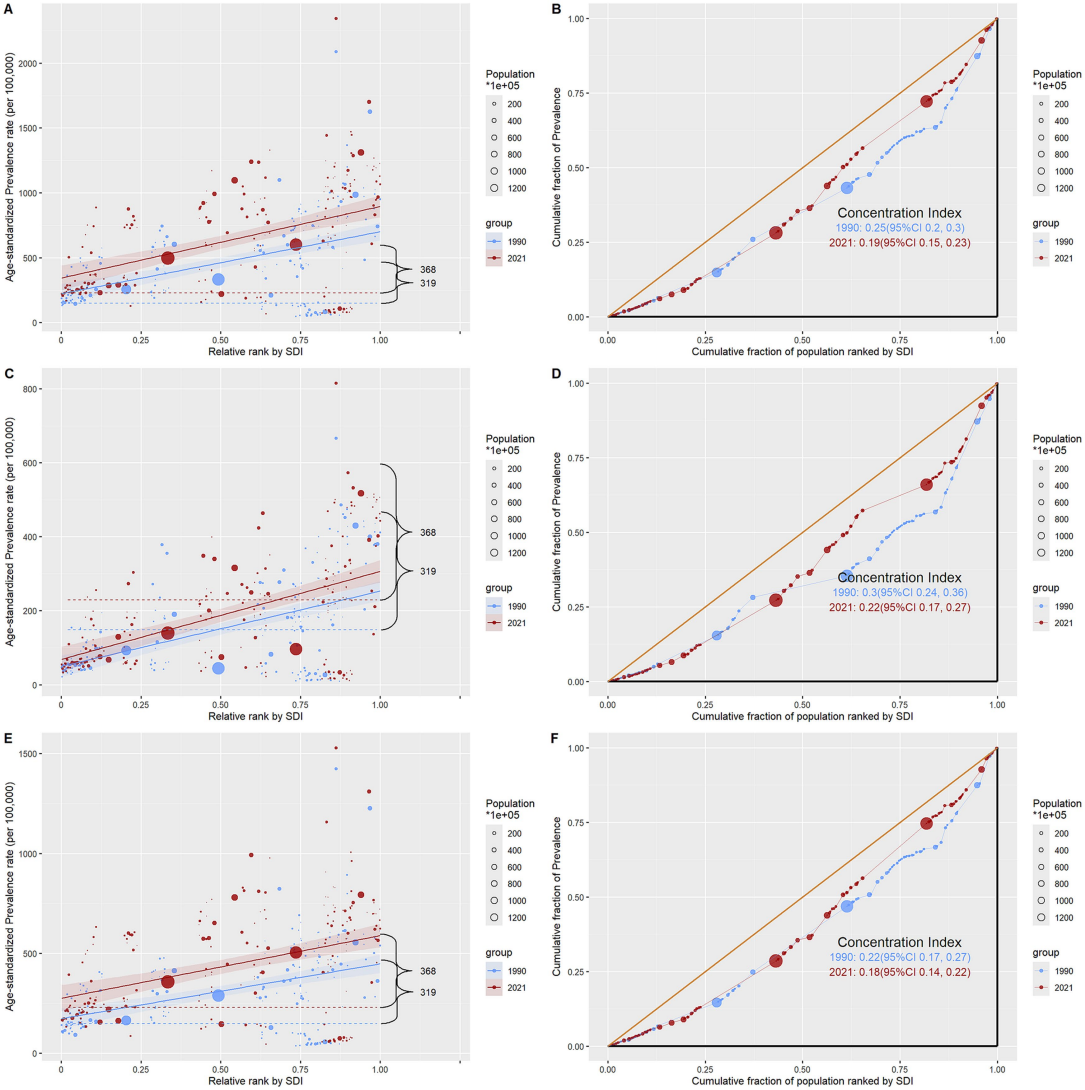
Regression curves of health inequalities and concentration curves for the prevalence of infertility caused by polycystic ovary syndrome worldwide, in 1990 and 2021. **(A,B)** Infertility. **(C,D)** Primary infertility. **(E,F)** Secondary infertility.

Health disparities across the socioeconomic development gradient exhibited a composite pattern of “widening absolute gaps and narrowing relative gaps” from 1990 to 2021, with a direction opposite to the classical “health gradient.” The slope index in Supplementary Table S12 indicates that the absolute inequality values for total infertility, primary infertility, and secondary infertility all increased from approximately 258 (95% CI: 200–316) in 1990 to 324 (95% CI: 243–405) in 2021 (*p* < 0.001), suggesting a further expansion of absolute disparities between high and low Socio-demographic Index (SDI) countries over time. However, the concentration index in Supplementary Table S13 reveals a consistent decline in relative inequality: the concentration index for total infertility decreased from 0.247 (95% CI: 0.196–0.298) to 0.191 (95% CI: 0.152–0.230); primary infertility declined from 0.300 (95% CI: 0.236–0.364) to 0.220 (95% CI: 0.165–0.274); and secondary infertility dropped from 0.222 (95% CI: 0.171–0.274) to 0.179 (95% CI: 0.142–0.217), all with statistically significant differences (*p* < 0.001). According to the regression curve in Figure 7, the positive *k* value indicates a positive association between ASPR and SDI, meaning that countries with higher SDI also exhibit higher ASPR. The concentration curve lies below the line of equality and shows a gradual flattening over time, further confirming that high-SDI populations bear a greater disease burden, while relative inequality is diminishing.

## Discussion

PCOS is the most common endocrine disorder and the leading cause of infertility in women of reproductive age (20) with impacts extending far beyond reproductive impairment to include metabolic disorders, cardiovascular risks, and psychosocial burdens. This study found that the global disease burden of Polycystic ovarian syndrome (PCOS)-related infertility significantly increased from 1990 to 2021, with key heterogeneities manifested as: secondary infertility grew faster than primary infertility; high-SDI regions bore the heaviest burden but showed slowing growth rates, while low- and middle-SDI regions, particularly middle-SDI areas, exhibited a “catch-up” surge; East Asia, South Asia, and other regions experienced the fastest growth, highlighting the urgency of addressing this condition as a global reproductive health priority. Clarifying its spatiotemporal distribution and driving mechanisms.

### Key findings and interpretation

We found that from 1990 to 2021, the global disease burden of infertility caused by PCOS showed a continuous and significant upward trend. Both the age-standardized prevalence rate (ASPR) and age-standardized years lived with disability rate (ASYR) of PCOS-related infertility increased persistently, with the absolute number of cases rising from 6.3155 million to 12.4655 million. This upward trend may be viewed as reflecting the combined effects of enhanced diagnostic capabilities and genuine epidemiological dynamics. As one of the most common endocrine disorders among women of reproductive age, PCOS affects 7–15% of reproductive-aged women worldwide. It is not only a leading cause of infertility but also significantly associated with a markedly increased lifetime risk of comorbidities such as type 2 diabetes mellitus, mental illnesses, and gynecological cancers (20, 21). Advances in diagnostic technology are considered one of the major contributors to the increasing burden. High-income countries have widely adopted the more sensitive Rotterdam diagnostic criteria, which more than doubles the case identification rate compared to the traditional NIH standards (22, 23). Additionally, multiple studies indicate that the relaxation of diagnostic thresholds under the Rotterdam criteria has significantly increased phenotypic heterogeneity, leading to reduced comparability of reported prevalence rates across countries (24). Meanwhile, the potential increase in true morbidity should not be overlooked, as environmental endocrine disruptors such as bisphenol A and phthalates may interfere with fetal follicular development and the regulation of sex hormones (25, 26). The global spread of sedentary lifestyles and high-sugar, high-fat diets has also been associated with an increased susceptibility to PCOS among younger generations of women (27, 28). Certain studies have indicated that the clustering of metabolic syndrome is becoming a key factor in the rising trend of PCOS illness (29). Therefore, the surge in the burden of PCOS-related infertility is likely the result of a complex interplay among advances in diagnostic capability, increased health awareness, and genuine epidemiological shifts.

The study further revealed a significant stratification of disease burden across the SDI gradient: high-SDI regions exhibited a “high baseline, low growth rate” pattern, whereas low- and middle-SDI regions showed a “low baseline, rapid catch-up.” High-SDI countries had the highest ASPR and ASYR globally, but the slowest AAPC. A plausible explanation for this observation is that their well-established medical infrastructure, including widespread application of the Rotterdam criteria, broad access to ultrasound technology, and high disease awareness, leading to more complete case detection (25, 30). For instance, Austria’s exceptionally high ASPR reflects superior diagnostic capacity (25). Furthermore, the prevalence of Western dietary patterns, obesity, and insulin resistance has substantially increased the true disease burden in these regions (30). The study by Joham et al. (31) also indicated that the prevalence of obesity among women with PCOS in high-income countries is much higher than in the general population, suggesting that nutrition-related risk factors remain decisively influential beyond diagnostic capacity McDermott et al. (32). In contrast, although low-SDI regions have the lowest baseline ASPR, their AAPC is rising rapidly, with middle-SDI countries showing particularly significant increases. This trend appears to coincide temporally with the accelerating process of global urbanization. The westernization of dietary patterns, decreased physical activity, and increased exposure to environmental pollutants collectively act as catalysts for PCOS incidence (25, 28). The World Health Organization has warned that developing countries are becoming new epicenters of obesity and diabetes mellitus, a shift directly linked to the penetration of ultra-processed foods and lifestyle transitions (26). More importantly, in these countries, abnormal menstruation during puberty and weight management are generally neglected, leading to a lack of early PCOS intervention and an expanding proportion of latent cases (33). The “transitional zone” nature of middle-SDI countries exposes them to a dual challenge: on one hand, improvements in medical diagnostic capacity have increased case detection, while on the other hand, urbanization pressures, nutritional imbalances, and environmental pollution may collectively contribute to a genuine rise in morbidity (29, 34).

It is noteworthy that the positive correlation between SDI and the burden of PCOS-related infertility not only reflects genuine epidemiological differences but also permeates the profound influences of healthcare accessibility, the prevalence of diagnostic criteria, and societal awareness levels. For instance, the rapid rise of ASPR in China benefits from both the improvement of primary healthcare systems and reflects policy focus on reproductive health; whereas the relatively high prevalence of PCOS in urban India suggests a potential role of urbanization in shaping the distribution of the disease (35). Scholars have pointed out that in countries like China and Singapore, the recognition rate of PCOS among women in rural areas is significantly lower than in urban areas, partly attributed to traditional perceptions that “menstruation irregular is not serious” (36). Although low-SDI regions report a lower burden, the growth rate warns of severe underdiagnosis (34). This “passive detection” model obscures the true epidemic level, as the low ASPR in Bosnia implies underreporting due to shortages in medical services (25).

From the perspective of age distribution, overall infertility exhibits a unimodal trend, with the global prevalence peak occurring at 40–44 years; however, primary infertility and secondary infertility display distinctly different age trajectories: the former peaks earlier at 20–24 years and subsequently declines, while the latter is concentrated at 40–44 years and demonstrates a “late-onset, affluent-type” characteristic with increasing SDI. This divergence reveals underlying differences in etiological mechanisms—primary infertility may more prominently reflect the early effects of hyperandrogenism during puberty and follicular developmental disturbance, particularly evident in young women with obesity and menstrual irregularities (29, 31). Numerous studies indicate that PCOS patients with hyperandrogenic phenotypes exhibit irregular menstruation and insulin resistance as early as puberty, which, if unaddressed, leads to a “metabolic-reproductive vicious cycle” (32, 37). The high incidence of secondary infertility is associated with the accumulation of long-term metabolic burden, persistent insulin resistance, and natural decline in ovarian reserve (22, 38). Researchers have found that although some women with PCOS can achieve pregnancy, they have fewer children than the general population, require a longer time to achieve their first delivery, and often experience further deterioration in insulin metabolism indicators postpartum (39, 40), highlighting the progressive erosion of the disease on reproductive potential. Additionally, individual lifestyle factors such as irregular routines, chronic pressure, and depressive disorder have been confirmed to have significant correlations with secondary PCOS-related fertility disorders (26).

Through the APC model analysis, we further dissected the triple time-dimensional effects. In terms of age effect, the risk of secondary infertility peaked at 35–39 years, while primary infertility was concentrated in 20–24 years, confirming physiological subtype differences. From the period effect perspective, the global relative risk (RR) of PCOS-related infertility has been continuously rising since 1992, with low-to-middle SDI countries exhibiting particularly accelerated growth in secondary infertility. Regarding the key cohort effect, the illness risk was significantly elevated in later birth cohorts. Using the 1972–1981 cohort as reference, the global RR of secondary infertility surged among those born in 1997–2006, with primary infertility also showing a synchronous increase. This risk escalation warrants high vigilance, as it may result from a complex interplay of environmental and social factors, such as fetal gonadal regulation disruption by environmental endocrine disruptors (25, 41). Puberty-onset Obesity, vitamin D deficiency, and poor dietary behaviors have been proven closely associated with abnormal follicular development (33, 34), while Sedentary lifestyles, high-calorie diets, delayed marriage/childbearing, and smoking are systematically accumulating in later birth cohorts as lifestyle risk factors (23, 27). Certain transgenerational studies indicate that daughters of mothers with PCOS have a significantly higher likelihood of developing elevated androgen levels and decreased Insulin sensitivity during Puberty (34), suggesting that the “familial clustering effect” in metabolic and hormonal pathways may accelerate risk transmission across cohorts. More importantly, many Low- and middle-income countries currently lack a systematic screening mechanism for PCOS during Puberty, leading to a large number of patients seeking medical attention for the first time due to infertility only at the stage of marriage and childbearing (22). The existence of such screening gaps not only delays the intervention window but also contributes to the formation of a “secondary-dominant” disease spectrum. Therefore, from a public health perspective, it is crucial to emphasize the impact of the lack of Puberty health education on the risk trajectory of the entire reproductive life cycle (42).

Based on the BAPC model, the global burden of PCOS-related infertility is projected to continue expanding by 2050, with the total number of illness cases potentially rising to 22.43 million. Secondary infertility will emerge as the core challenge, with its age-standardized prevalence rate expected to increase to 752 per 100,000, accounting for 82.8% of total PCOS infertility cases (approximately 18.57 million). This trend aligns closely with the currently observed period and cohort effects, indicating a future triple challenge: an enlarged population with fertility barriers, a surge in assisted reproductive demands, and mounting pressure on the healthcare system. The explosive growth of secondary infertility may involve the combined effects of multiple factors: Against the global backdrop of delayed first-childbearing age, uncontrolled PCOS-related insulin resistance and metabolic disorders will significantly exacerbate the “reproductive dilemma” among older women. Meanwhile, existing medical resource allocations predominantly favor first-time Pregnancy populations, resulting in long-term neglect of the specific needs of secondary infertility within policy frameworks (29). To address this trend, it is essential to prioritize the establishment of a reproductive management network in high-burden countries, strengthen the long-term follow-up mechanism for metabolic recovery in the stage of convalescence among women with PCOS, and target Obesity prevention and control as a key intervention (27, 32). Additionally, rolling out a remote monitoring-based metabolic assessment and electronic health education system among women with secondary infertility will help bridge the contact barriers between older patients and health services (33).

To elucidate the driving mechanisms behind the 6.15 million new cases from 1990 to 2021, we employed decomposition analysis to reveal the tripartite contributions of population growth, aging, and epidemiological changes. At the global level, the expansion of the population base and the rise in true illness rates were the primary drivers, while the direct impact of population aging was minimal. A deeper stratification by infertility types showed that the increase in primary infertility was mainly driven by the growth of the reproductive-age population, with its epidemiological contribution even turning negative, suggesting a potential stabilization of this burden. In contrast, the surge in secondary infertility exhibited a dual-engine driving pattern—dominated jointly by population growth and epidemiological changes, with aging also exerting a slight positive influence. When analyzed along the SDI gradient, the heterogeneity of driving mechanisms becomes more pronounced. The expansion of burden in high and middle-high SDI countries primarily stems from epidemiological shifts, which may reflect both the genuine disease-promoting effects of Western dietary patterns and the obesity epidemic (30), as well as the amplifying role of widespread diagnostic criteria and advancements in ultrasound technology on case reporting (25). In contrast, growth in low and low-middle SDI regions heavily depends on population base expansion, with limited healthcare resources leading to a significant number of unrecognized cases, where patients often present with “infertility” as their initial complaint (24, 34). Notably, the contribution of aging is negative across all SDI groups, consistent with PCOS predominantly affecting young women, further emphasizing that prevention and control efforts should focus on the 25–44 age group (26, 42).

The analysis of health inequalities across the SDI gradient in infertility caused by PCOS reveals that the absolute burden disparity between high-SDI and low-SDI countries continues to widen, while relative inequality steadily declines. This seemingly paradoxical outcome stems from the complex interplay of multidimensional factors. On one hand, developed countries, with their robust medical infrastructure—such as Austria’s diagnostic systems and sensitive diagnostic criteria (Rotterdam criteria)—more fully reflect the substantial reproductive health pressure imposed by Western lifestyles and the obesity epidemic (22), and more comprehensively capture the true disease burden (25, 34), indicating that, even in resource-rich settings, PCOS remains a major reproductive endocrine disorder requiring significant investment from health systems. On the other hand, low-SDI regions face limitations due to disparities in specialized service accessibility, diagnostic capacity, and disease management, as well as cultural perception differences, such as ultrasound equipment shortages in Bosnia and the perception of obesity as a health symbol in sub-Saharan Africa, leading to significant underdiagnosis (27). More notably, certain groups face compounded barriers. For instance, Muslim immigrant women in Austria experience additional pressure when seeking infertility treatment due to language barriers, gender quarantine taboos, and privacy dilemmas requiring male relatives to act as translators (36). Ethnic minority women often encounter “multiple marginalization” in accessing assisted reproduction, with their infertility symptoms frequently misattributed to cultural behavioral differences, leading to systemic neglect. Meanwhile, rapid urbanization and lifestyle changes in low-SDI regions are driving a “catch-up” rise in the true incidence of PCOS, reflected in higher growth rates, thereby promoting the convergence of relative disparities (43). However, this “relative improvement” should not obscure the substantial unmet healthcare needs that persist in low-SDI regions. Women in these areas may still lack timely diagnosis and treatment due to cultural perceptions, economic barriers, and limited healthcare resources, resulting in a severe underestimation of the true disease burden (44).

Framed within the United Nations Sustainable Development Goals (SDGs), this dual pattern carries profound public health implications. The widening of absolute inequality calls for high-SDI countries to manage PCOS as a life-course chronic condition under SDG 3.4 for reducing the burden of non-communicable diseases. The convergence of relative inequality, for low- and middle-SDI countries, implies more complex challenges in pursuing SDG 3.8 (universal health coverage) and SDG 5 (gender equality): it requires both strengthening primary screening to bridge diagnostic gaps and community-level interventions targeting modifiable risk factors such as obesity, to curb the rapid rise in true incidence and prevent further widening of absolute disparities in the future. Infertility and its psychosocial consequences are deeply linked to women’s social status and self-realization (SDG 5), and ensuring equitable access to reproductive health services for all women is central to achieving health-related sustainable development goals.

Based on the above findings, we propose that future intervention strategies should implement stratified management according to different PCOS phenotypic characteristics and age-of-onset distributions. For primary PCOS, proactive identification during puberty and older lifestyle interventions should be prioritized. For secondary PCOS, enhanced reproductive function monitoring postpartum and in the older period is essential to bridge the current care gap of “losing follow-up after childbirth” (26, 36, 38). High-SDI countries should focus on key life cycle stages, strengthening androgen regulation during puberty and postpartum metabolic management to prevent the progression of mild PCOS to infertility. Roll out validated lifestyle interventions and integrate successful experiences into the primary healthcare system (41); additionally, introduce AI-based menstrual cycle tracking and ultrasound screening systems to enhance dynamic identification of mild PCOS (35). Medium-SDI countries need to prioritize improving grassroots screening capabilities and establish population management networks centered on schools and first-time mothers. Prioritize interventions targeting modifiable risk factors (such as processed food consumption and sedentary behavior) and localize effective strategies from high-SDI countries (25, 30). Low-SDI countries should prioritize increasing early identification rates, exploring innovative approaches like portable ultrasound devices and AI-assisted preliminary screening tools to overcome resource limitations, with particular attention to rural adolescent populations (35, 37). Global health organizations should promote the establishment of a standardized PCOS registry system to address the representational gap of GBD in low-resource regions. Only through precise gradient strategies and global collaboration can we tackle this quietly spreading reproductive health crisis, ultimately advancing the SDGs’ goals of reproductive health equity and gender equality.

### Advantages and limitations

This investigation was conducted using standardized data from the Global Burden of Disease (GBD) 2021 database, covering 204 countries and regions. It established a broad temporal framework, providing a macro perspective for assessing the global burden of infertility associated with polycystic ovary syndrome (PCOS). Addressing the issue of non-homogeneous baseline data quality and monitoring systems across different global regions, the GBD data leveraged its core strengths: The DisMod-MR Bayesian meta-regression modeling framework systematically integrates heterogeneous data sources, including epidemiological surveys, registry data, systematic reviews, and hospital records. Through covariate adjustment and hierarchical prior distributions, it achieves standardized estimates across geographical and temporal dimensions, thereby enhancing the global comparability and representativeness of results at the methodological level (11, 12).

Temporal trends in PCOS-related infertility burden were systematically evaluated through the integration of Joinpoint regression, age-period-cohort (APC) models, and Bayesian age-period-cohort (BAPC) models. The influence of demographic and socioeconomic determinants on disease burden was further examined through decomposition and health inequity analyses, providing an evidence base for targeted therapeutic interventions (6).

Despite offering a comprehensive evaluation of PCOS-related infertility burden, several methodological limitations were acknowledged in this investigation. First, although the GBD modeling approach offers significant advantages in handling data heterogeneity, we must acknowledge that the non-homogeneity of input data remains one of the primary limitations of this study. Reporting systems for PCOS and infertility vary across countries and regions in terms of diagnostic criteria, data coverage, and monitoring depth. Particularly in low- and lower-middle-income countries, inadequate baseline monitoring and sparse samples significantly widen the model’s uncertainty interval (45). Therefore, although the GBD modeling process achieves standardized estimates technically, the precision of the output results remains dependent on the completeness and consistency of the input data. This may lead to increased bias or uncertainty in burden estimates across different regions. Future research should further integrate high-quality raw data and prospective clinical evidence to validate and optimize global estimation results. Second, evolving diagnostic criteria for PCOS throughout the study period, particularly the widespread implementation of the 2003 Rotterdam consensus, may have confounded temporal trend assessments (46). Additionally, the employed APC model possesses inherent identifiability constraints, while the BAPC model failed to incorporate potential healthcare policy modifications or therapeutic advances when generating future projections, thereby potentially compromising predictive accuracy. Finally, although the Socio-demographic Index (SDI) functions as a comprehensive proxy for socioeconomic development and captures national-level educational attainment, economic status, and fertility patterns, it cannot adequately reflect the substantial influence of cultural heterogeneity, gender disparities, or healthcare accessibility on PCOS diagnostic practices and therapeutic outcomes. Consequently, interpretation of these findings necessitates careful consideration of the aforementioned constraints, and future investigations should prioritize the incorporation of higher-quality primary data to enhance the reliability and precision of epidemiological conclusions.

## Conclusion

Globally, PCOS-related infertility has evolved from a marginal issue into a major public health challenge spanning multiple dimensions of physiology, psychology, and society. Its continuous expansion not only reflects the complex interaction of diagnostic capabilities, lifestyles, and environmental exposure but also exposes the deep fractures between medical resource allocation, cultural cognition, and policy responses. Faced with the upcoming wave of tens of millions of new cases, countries urgently need to shift toward precise prevention and control centered on the life cycle and measured by social gradients. Only by integrating early puberty identification, metabolic risk blocking, long-term post-partum follow-up, and empowerment of vulnerable populations into coherent actions, and bridging the resource gap through cross-border data sharing and technology downscaling, can we curb the systematic erosion of PCOS infertility on the reproductive health of the next generation of women and truly realize the global health vision of “leaving no one behind.”

## Data Availability

Publicly available datasets were analyzed in this study. This data can be found at: all data used from the GHDx platform (https://vizhub.healthdata.org/gbd-results/).

## Footnotes

^1^ https://vizhub.healthdata.org/gbd-results/
