# Supplementary material for: Global, regional, and national burden of infertility attributable to polycystic ovary syndrome, 1990–2021: results from the Global Burden of Disease Study 2021
Source: Front Public Health. 2025 Nov 10;13:1693486. doi: 10.3389/fpubh.2025.1693486 (PMC12640974; doi:10.3389/fpubh.2025.1693486)
Supplement: Supplementary file 1 [file Presentation_1.pdf]

Supplementary methods

Data Sources

This study utilizes data from the 2021 Global Burden of Disease Study (GBD 2021), which systematically estimated the epidemiological burden of 371 diseases and injuries across 204 countries and regions from 1990 to 2021. This data is publicly available through the Institute for Health Metrics and Evaluation (IHME) Global Health Data Exchange platform (https://vizhub.healthdata.org/gbd-results/). We primarily extracted data on women of reproductive age (15–49 years) from population-based surveys, including the Demographic and Health Surveys (DHS), World Fertility Surveys (WFS), Reproductive Health Surveys (RHS), and Family and Fertility Surveys (FFS). The Socio-Demographic Index (SDI) developed by IHME serves as a composite measure of socioeconomic development and its impact on health (1). It is calculated as the geometric mean of three indicators: total fertility rate among women under 25, average years of schooling for those aged 15 and older, and lagged per capita income distribution. SDI values range from 0 to 1, with higher values indicating greater socioeconomic development. Countries and regions are categorized into five tiers based on their SDI: low, lower-middle, middle, upper-middle, and high.

This study examines the disease burden of female infertility and its subtypes (primary and secondary infertility). Infertility is defined as a reproductive health disorder where a couple of reproductive age fails to conceive after at least 12 months of regular unprotected intercourse. According to the Global Burden of Disease (GBD)

study and World Health Organization (WHO) classifications, infertility can be further categorized into primary and secondary infertility. Primary infertility refers to cases where the female partner has never achieved pregnancy despite a fertile male partner, often associated with congenital reproductive system abnormalities or ovulation disorders. Secondary infertility denotes a history of successful pregnancy or childbirth followed by at least one year of unsuccessful attempts to conceive, typically linked to infections, inflammation, or acquired reproductive system dysfunction (2). This study adheres to the disease classification and modeling methodology of GBD 2021, explicitly including both primary and secondary infertility cases in the analysis to comprehensively assess the epidemiological burden of PCOS-related infertility among women of reproductive age (15–49 years).

Within the Global Burden of Disease (GBD) research framework, the definition of polycystic ovary syndrome (PCOS) references the standardized diagnostic criteria proposed by the American College of Obstetricians and Gynecologists (ACOG) (3). The ACOG system permits the use of three internationally recognized diagnostic approaches: the National Institutes of Health (NIH) criteria, the Rotterdam criteria, and the Androgen Excess–PCOS Society (AE-PCOS) criteria. All these criteria identify chronic anovulation and hyperandrogenism as core diagnostic features of PCOS, requiring exclusion of other conditions causing secondary hyperandrogenism or ovulatory dysfunction before diagnosis. (4). Among these, the Rotterdam criteria are most widely adopted in clinical and epidemiological studies. The Rotterdam criteria require fulfillment of at least two of the following three features: ovulatory dysfunction, clinical or biochemical evidence of hyperandrogenism, and ultrasound evidence of polycystic ovarian morphology (3). This study incorporates PCOS cases meeting the comprehensive definition under the ACOG framework, combined

with the Rotterdam criteria diagnostic features, and assesses the global burden of disease associated with infertility (including primary and secondary infertility) caused by PCOS.

To assess the global burden of polycystic ovary syndrome-related infertility among women of reproductive age (15–49 years) from 1990 to 2021, this study utilized data from the Global Burden of Disease (GBD) 2021 database. Analysis metrics included the number of people affected, years lost due to disability (YLDs), age-standardized prevalence rate (ASPR), and age-standardized YLD rate (ASYR). All indicators were age-standardized according to the GBD global standard population to ensure comparability across countries and time periods (5). Disability-adjusted life years (YLDs) were calculated by multiplying the prevalence of infertility by the corresponding disability weight (DW), reflecting the impact of the disease on healthy life expectancy.

GBD data are integrated from multiple sources, including administrative records, disease registries, population health surveys, and surveillance systems. Differences among countries in data coverage, diagnostic criteria, and monitoring quality may lead to variations in the accuracy and completeness of estimates across regions (4). To quantify the overall variability attributable to data differences, model assumptions, and parameter uncertainties, GBD provides an Uncertainty Interval (UI) for each estimate. The point estimates in this research report represent the mean of the model's posterior distribution. The corresponding 95% UI is determined by the 2.5th and 97.5th percentiles of 1,000 posterior samples, serving to quantify the range of variation in the estimates (6).

Joinpoint Regression Model

To reveal temporal trends in the global disease burden of polycystic ovary syndrome (PCOS)-related infertility from 1990 to 2021, this study employed a Joinpoint regression model for analysis. This model identifies joinpoints to segment the study period into distinct trend segments, fitting and optimizing trends within each segment (7). Consequently, compared to traditional regression models, Joinpoint regression enables a more detailed and precise assessment of specific disease change characteristics across different intervals throughout the entire timeframe (8). Considering the analysis of epidemiological trends in PCOS-related infertility prevalence, ASPR, YLDs, and ASYR, we constructed a log-linear regression model: $\ln(y) = \beta * x + \text{constant}$, where y represents incidence and x denotes the year of occurrence. Weighted least squares fitting was employed to fully utilize the standard errors provided by GBD data, thereby enhancing the robustness of estimates (9). Furthermore, to identify potential inflection points (joinpoints) in trend changes, the model employed the default grid search method (GSM) within Joinpoint software. GSM establishes joinpoints across all possible time combinations, calculates the mean squared errors (MSE) for each scenario, and selects the grid point with the smallest MSE as the joinpoint. Based on this, model optimization and determination of the optimal number of joinpoints utilize Joinpoint software's default Monte Carlo permutation test, identifying the optimal model at a significance level of $\alpha < 0.05$. We set the maximum number of potential joinpoints to 5 and the minimum to 0. The permutation test begins with the number of joinpoints $k = 0$ and $k\_max = 5$. If $k \neq k\_max$, k is set to k+1, and the test continues until the model corresponding to $k = k\_max$ is selected as the optimal model. To control for multiple comparison errors, the Bonferroni correction is further applied to adjust the significance level. Key

metrics for the joinpoint model include the Annual Percentage Change (APC) describing segmented trends and the Average Annual Percentage Change (AAPC) summarizing overall trends, along with their 95% Confidence Intervals (CI). The APC is calculated as:

$$\mathrm{APC} = \left(e^{\beta} - 1\right) * 100$$

Where β is the regression coefficient of the log-linear model lny = β*x + constant. AAPC is calculated as the weighted average of segmented APCs using the interval width w, representing the overall trend in prevalence from 1990 to 2021. The formula for AAPC is:

$$AAPC = \left(exp^{\Sigma w_i \beta_i / \Sigma w_i} - 1\right) * 100$$

Here, w_i denotes the width of each segment function interval (i.e., the number of years contained within the interval), while β_i represents the regression coefficient corresponding to each interval. AAPC > 0 indicates an upward trend in the relevant indicator, whereas AAPC < 0 signifies a downward trend (10).

Age–Period–Cohort Model

This study employed an Age–Period–Cohort (APC) model to examine the independent effects of different ages, periods, and birth cohorts on the prevalence of polycystic ovary syndrome (PCOS)-related infertility (11). Within the APC model, Net drift denotes the overall annual percentage change (APC) in prevalence throughout the study period, reflecting the combined effect of calendar time and consecutive birth cohorts on the overall log-linear trend. Local drift, in contrast, represents the log-linear trend within each age group stratified by calendar period and birth cohort—that is, the annual percentage change in prevalence for each age group (12). Statistical significance of trends was assessed using the Wald χ^2 test. Model results included longitudinal age curves, period rate ratios (RR), and cohort rate ratios (RR). Longitudinal age curves depict age-specific rates adjusted for period effects within the reference cohort; period RR measures risk changes across periods relative to the reference period, adjusted for age and nonlinear cohort effects; cohort RR measures risk differences across birth cohorts relative to the reference cohort, adjusted for age and nonlinear period effects. RR > 1 indicates increased risk compared to the reference group, while RR < 1 indicates reduced risk.

In the APC model, age intervals must match period intervals, so we paired five-year age groups with five-year time periods. Women of reproductive age (15–49 years) were divided into seven five-year age cohorts (15–19, 20–24, 25–29, 30–34, 35–39, 40–44, 45–49), and the study period from 1992 to 2021 was divided into six consecutive five-year periods (1992–1996, 1997–2001, 2002–2006, 2007–2011, 2012–2016, 2017–2021). The 1990–1991 period was excluded from analysis as it did not form a complete five-year interval. Birth cohorts were defined by subtracting age from the period of event occurrence (i.e., cohort = period-age), yielding 12 partially overlapping ten-year birth cohorts (1942–1951 to 1997–2006) based on the above division. The model used 1992–1996 as the reference period and 1972–

1981 as the reference birth cohort for calculating relative risks. The selection of the reference period and cohort is arbitrary and does not affect trend interpretation. Analysis using the APC model helps reveal the age dependency, generational differences, and temporal evolution of the burden of polycystic ovary syndrome-related infertility, providing epidemiological evidence for developing targeted reproductive health prevention and control strategies.

BAPC Model Predictions

To forecast future trends in polycystic ovary syndrome-related infertility globally and at the national level from 2022 to 2050, this study employed a Bayesian Age–Period–Cohort (BAPC) model based on the Integrated Nested Laplace Approximation (INLA) framework and reports the 95% credible intervals (Cl) of its posterior distribution. INLA achieves posterior inference through deterministic Laplace approximation, eliminating reliance on computationally intensive Markov Chain Monte Carlo (MCMC) sampling. This approach significantly enhances computational efficiency and convergence stability while maintaining precision (13, 14).

In this study, the BAPC model was constructed within a log-linear framework based on the Poisson distribution, assuming that prevalence is associated with three latent effects: age, period, and cohort. To smooth random fluctuations between adjacent time periods and cohorts, second-order random walk (RW2) structures were applied to the age, period, and cohort terms. Their precision parameters were assigned log-gamma prior distributions to account for uncertainty in smoothing levels. An additional independent and identically distributed (iid) random effects term was included to adjust for excessive dispersion beyond the structured trend, thereby

improving the robustness of short-term predictions (15). All other parameters in this model used default settings from the "BAPC" R package. Projections were based on the standard age structure from the Global Burden of Disease (GBD) 2021 database, combined with World Health Organization (WHO) population projections, to estimate the number of cases and age-standardized prevalence of PCOS-related infertility from 2022 to 2050.

Decomposition Analysis

To investigate the association between the burden of infertility associated with polycystic ovary syndrome and the Socio-Demographic Index (SDI), this study employed the decomposition analysis method developed by Das Gupta to identify the primary drivers of changes in the global burden of infertility attributable to polycystic ovary syndrome from 1990 to 2021. This method decomposes the total change in PCOS-related infertility prevalence into three independent components: population growth, population aging, and epidemiological factors (9, 16).

Specifically, we calculated the prevalence for each region using the following formula:

$$Prevalence_{a_y,p_y,e_y} = \sum_{i=1}^{7} \left(a_{i,y} * p_y * e_{i,y}\right)$$

$Prevalence_{a_y,p_y,e_y}$ denotes the prevalence resulting from the cumulative effects of population aging, population growth, and epidemiological changes in year y. $a_{i,y}$ represents the proportion of the i-th age group among the seven age groups in year y. p_y denotes the population size of age group i in year y. $e_{i,y}$ denotes the prevalence rate of age group i in year y.

This analytical framework enables systematic evaluation of temporal trends in demographic and epidemiological determinants, quantifying the relative contributions of each factor to changes in disease burden. It thereby reveals the key drivers behind the global increase in polycystic ovary syndrome-related infertility [7]. We determine the impact of a single factor on prevalence while holding other factors constant. Therefore, to calculate the contribution of population aging to prevalence change, the formula is as follows:

$$Effect_{2021} = \left[\frac{Prevalence_{a_{2021},p_{2021},e_{2021}} + Prevalence_{a_{2021},p_{1990},e_{1990}}}{3} + \frac{Prevalence_{a_{2021},p_{1990},e_{2021}} + Prevalence_{a_{2021},p_{2021},e_{1990}}}{6}\right] - \left[\frac{Prevalence_{a_{1990},p_{2021},e_{2021}} + Prevalence_{a_{1990},p_{1990},e_{1990}}}{3} + \frac{Prevalence_{a_{1990},p_{1990},e_{2021}} + Prevalence_{a_{1990},p_{2021},e_{1990}}}{6}\right]$$

Analysis of Health Inequality

To assess variations in the distribution of infertility burden caused by polycystic ovary syndrome (PCOS) across countries with differing socioeconomic development levels, this study employed the Slope Index of Inequality (SII) and Concentration Index (CI) to quantify health inequality. These metrics respectively reflect absolute and relative inequality in disease burden distribution across varying levels of socio-demographic development (17).

The SII measures absolute inequality in disease burden. We first ranked countries and regions in ascending order based on the Socio-demographic Index (SDI) and calculated the midpoint of the cumulative population distribution as the socioeconomic rank. The age-standardized prevalence rate (ASPR) among women aged 15–49 was then regressed against this cumulative rank. The slope of the regression model represents the SII, signifying the absolute difference in disease burden between countries at the highest and lowest ends of the SDI spectrum. To mitigate the impact of outliers and data heterogeneity on regression outcomes, robust weighted regression (rlm) was employed instead of ordinary least squares (lm). A larger absolute SII value indicates a more pronounced disparity in disease burden between countries (16).

CI measures relative disease burden inequality, calculated based on the Lorenz concentration curve. This curve ranks countries by socioeconomic development index (SDI) from low to high, plotting the cumulative proportion of population on the x-axis against the cumulative proportion of corresponding age-standardized prevalence rates (ASPR) on the y-axis. CI is obtained by numerically integrating the area under the curve, reflecting disparities in disease burden distribution across socioeconomic

strata. The CI ranges from −1 to 1, where CI = 0 indicates a perfectly equal distribution of disease burden across countries; CI > 0 indicates burden concentration in high-SDI countries; CI < 0 indicates burden concentration in low-SDI countries. A higher absolute value of CI signifies greater inequality in the distribution of PCOS-related infertility burden among countries and regions at different socioeconomic development levels (18).

Supplementary References:

1. Liu X, Zhang J, Wang S. Global, regional, and national burden of infertility attributable to PCOS, 1990-2019. Hum Reprod. 2024;39(1):108-18.
2. Chen Y, Liu C, Wang X, Liu Y, Liu H. Global, Regional and National Burden of Infertility due to Endometriosis: Results From the Global Burden of Disease Study 2021 and Forecast to 2044. Bjog. 2025;132(7):944-60.
3. Stener-Victorin E, Teede H, Norman RJ, Legro R, Goodarzi MO, Dokras A, et al. Polycystic ovary syndrome. Nat Rev Dis Primers. 2024;10(1):27.
4. Huo M, Wang Y, Yuan X, Yuan Y, Zhang X. Changing trends in the global burden of polycystic ovarian syndrome-related infertility over the past 30 years: retrospective data analysis of the global burden of disease study 2019. BMC Womens Health. 2025;25(1):35.
5. Global burden of 369 diseases and injuries in 204 countries and territories, 1990-2019: a systematic analysis for the Global Burden of Disease Study 2019. Lancet. 2020;396(10258):1204-22.
6. Rudd KE, Johnson SC, Agesa KM, Shackelford KA, Tsoi D, Kievlan DR, et al. Global, regional, and national sepsis incidence and mortality, 1990-2017: analysis for the Global Burden of Disease Study. Lancet. 2020;395(10219):200-11.
7. Kim HJ, Fay MP, Feuer EJ, Midthune DN. Permutation tests for joinpoint regression with applications to cancer rates. Stat Med. 2000;19(3):335-51.
8. Zhang J, Ma B, Han X, Ding S, Li Y. Global, regional, and national burdens of HIV and other sexually transmitted infections in adolescents and young adults aged 10-24 years from 1990 to 2019: a trend analysis based on the Global Burden of Disease Study 2019. Lancet Child Adolesc Health. 2022;6(11):763-76.
9. Hu J, Ke R, Teixeira W, Dong Y, Ding R, Yang J, et al. Global, Regional, and National Burden of CKD due to Glomerulonephritis from 1990 to 2019: A Systematic Analysis from the Global Burden of Disease Study 2019. Clin J Am Soc Nephrol. 2023;18(1):60-71.
10. Tuo Y, Li Y, Li Y, Ma J, Yang X, Wu S, et al. Global, regional, and national burden of thalassemia, 1990-2021: a systematic analysis for the global burden of disease study 2021. EClinicalMedicine. 2024;72:102619.
11. Rosenberg PS, Check DP, Anderson WF. A web tool for age-period-cohort analysis of cancer incidence and mortality rates. Cancer Epidemiol Biomarkers Prev.

2014;23(11):2296-302.
12. Zou Z, Liu G, Hay SI, Basu S, Belgaumi UI, Dhali A, et al. Time trends in tuberculosis mortality across the BRICS: an age-period-cohort analysis for the GBD 2019. EClinicalMedicine. 2022;53:101646.
13. Zhao H, Xu T, Shen H. Global, regional, and national disability-adjusted life years and prevalence of lymphatic filariasis from 1990 to 2021: A trend and health inequality analysis based on the global burden of disease study 2021. PLoS Negl Trop Dis. 2025;19(4):e0013017.
14. Knoll M, Furkel J, Debus J, Abdollahi A, Karch A, Stock C. An R package for an integrated evaluation of statistical approaches to cancer incidence projection. BMC Med Res Methodol. 2020;20(1):257.
15. Du Z, Chen W, Xia Q, Shi O, Chen Q. Trends and projections of kidney cancer incidence at the global and national levels, 1990-2030: a Bayesian age-period-cohort modeling study. Biomark Res. 2020;8:16.
16. Wang R, Chen Y, Shao X, Chen T, Zhong J, Ou Y, et al. Burden of Skin Cancer in Older Adults From 1990 to 2021 and Modelled Projection to 2050. JAMA Dermatol. 2025;161(7):715-22.
17. Mújica Ó J, Moreno CM. [From words to action: measuring health inequalities to "leave no one behind"Da retórica à ação: mensurar as desigualdades em saú de para não deixar ninguém atrás]. Rev Panam Salud Publica. 2019;43:e12.
18. Qu C, Liao S, Zhang J, Cao H, Zhang H, Zhang N, et al. Burden of cardiovascular disease among elderly: based on the Global Burden of Disease Study 2019. Eur Heart J Qual Care Clin Outcomes. 2024;10(2):143-53.

Table S1. Prevalence and years lived with disability of infertility due to PCOS and their average annual percentage changes from 1990 to 2021 at the global and regional levels.

	ASPR, per 100,000 (95% UI)		AAPC (95% CI)	ASYR, per 100,000 (95% UI)		AAPC (95% CI)
**Location**	**1990**	**2021**	**1990-2021**	**1990**	**2021**	**1990-2021**
Global	475.54 (293.03, 725.57)	638.15 (388.26, 982.60)	0.96 (0.95 to 0.98)	2.77 (1.05, 6.26)	3.67 (1.36, 8.33)	0.92 (0.9 to 0.93)
**Socio-demographic index**						
High SDI	933.61 (541.50, 1477.50)	1137.85 (673.39, 1771.12)	0.63 (0.62 to 0.65)	5.57 (1.99, 12.45)	6.73 (2.42, 15.14)	0.6 (0.58 to 0.62)
High-middle SDI	420.18 (255.87, 641.49)	649.01 (384.87, 1020.25)	1.43 (1.42 to 1.44)	2.41 (0.91, 5.59)	3.70 (1.36, 8.49)	1.41 (1.4 to 1.42)
Middle SDI	429.86 (260.36, 660.04)	727.72 (434.04, 1138.92)	1.72 (1.7 to 1.74)	2.47 (0.91, 5.62)	4.16 (1.50, 9.59)	1.7 (1.68 to 1.72)
Low-middle SDI	290.66 (178.76, 460.68)	460.37 (279.07, 726.94)	1.5 (1.48 to 1.53)	1.71 (0.64, 3.88)	2.66 (0.98, 6.12)	1.43 (1.41 to 1.45)
Low SDI	198.14 (120.61, 319.55)	280.24 (170.95, 444.96)	1.14 (1.13 to 1.16)	1.14 (0.42, 2.61)	1.59 (0.58, 3.65)	1.11 (1.09 to 1.12)

Region						
Andean Latin America	588.53 (361.34, 888.78)	988.69 (460.41, 1667.29)	1.77 (1.63 to 1.88)	3.32 (1.21, 7.56)	5.48 (1.71, 13.21)	1.63 (1.56 to 1.69)
Australasia	947.97 (349.99, 1837.69)	1091.33 (412.72, 2083.40)	0.45 (0.44 to 0.47)	5.60 (1.44, 14.06)	6.42 (1.68, 16.16)	0.44 (0.43 to 0.46)
Caribbean	450.31 (265.11, 714.85)	554.29 (323.89, 896.19)	0.68 (0.67 to 0.69)	2.65 (0.96, 6.09)	3.20 (1.13, 7.52)	0.62 (0.61 to 0.63)
Central Asia	134.14 (78.24, 218.72)	184.63 (106.80, 298.04)	1.04 (1.03 to 1.06)	0.77 (0.26, 1.80)	1.05 (0.36, 2.51)	1.02 (1.01 to 1.05)
Central Europe	71.12 (39.57, 120.47)	87.39 (49.93, 141.39)	0.66 (0.66 to 0.67)	0.40 (0.14, 0.97)	0.49 (0.16, 1.17)	0.63 (0.62 to 0.63)
Central Latin America	850.81 (501.68, 1357.06)	1008.30 (605.07, 1592.27)	0.55 (0.52 to 0.58)	4.87 (1.76, 11.44)	5.67 (2.10, 13.21)	0.49 (0.46 to 0.52)
Central Sub-Saharan Africa	178.64 (105.38, 296.27)	270.88 (157.13, 444.18)	1.37 (1.33 to 1.42)	1.01 (0.34, 2.41)	1.52 (0.53, 3.53)	1.36 (1.31 to 1.4)
East Asia	337.67 (193.80, 547.28)	602.50 (343.11, 988.13)	1.89 (1.87 to 1.91)	1.82 (0.61, 4.28)	3.29 (1.12, 7.78)	1.92 (1.9 to 1.94)
Eastern Europe	80.96 (46.23,	104.79 (60.52,	0.85	0.48 (0.17, 1.10)	0.61 (0.21, 1.42)	0.83

	133.84)	171.82)	(0.84 to 0.86)			(0.82 to 0.84)
Eastern Sub-Saharan Africa	204.00 (123.63, 327.54)	261.48 (158.58, 418.64)	0.83 (0.8 to 0.86)	1.15 (0.42, 2.61)	1.46 (0.53, 3.37)	0.8 (0.77 to 0.83)
High-income Asia Pacific	1315.56 (588.50, 2294.91)	1396.20 (606.20, 2447.66)	0.21 (0.19 to 0.23)	7.45 (2.19, 17.93)	7.88 (2.26, 19.17)	0.19 (0.18 to 0.21)
High-income North America	934.15 (499.55, 1556.07)	1240.88 (726.05, 1936.11)	0.93 (0.87 to 0.99)	5.72 (1.94, 12.84)	7.48 (2.67, 16.61)	0.87 (0.8 to 0.93)
North Africa and Middle East	620.87 (382.18, 971.67)	828.11 (492.27, 1320.48)	0.95 (0.92 to 0.98)	3.80 (1.45, 8.66)	4.91 (1.80, 11.27)	0.83 (0.81 to 0.86)
Oceania	474.58 (276.27, 794.52)	637.62 (377.67, 1033.92)	0.94 (0.92 to 0.96)	2.69 (0.94, 6.27)	3.59 (1.29, 8.44)	0.91 (0.89 to 0.93)
South Asia	244.79 (152.00, 382.27)	441.86 (271.78, 691.57)	1.93 (1.91 to 1.95)	1.45 (0.54, 3.31)	2.55 (0.94, 5.87)	1.84 (1.81 to 1.86)
Southeast Asia	583.28 (344.42, 937.42)	1034.75 (614.12, 1663.61)	1.88 (1.86 to 1.91)	3.41 (1.20, 7.75)	5.98 (2.13, 13.84)	1.83 (1.81 to 1.85)
Southern Latin America	425.26 (247.42, 719.25)	661.08 (382.83, 1106.49)	1.46 (1.44 to 1.48)	2.51 (0.87, 5.85)	3.91 (1.36, 9.07)	1.46 (1.44 to 1.48)

Southern Sub-Saharan Africa	333.19 (195.25, 535.09)	401.86 (232.26, 665.34)	0.64 (0.61 to 0.66)	1.91 (0.68, 4.41)	2.30 (0.79, 5.36)	0.62 (0.59 to 0.64)
Tropical Latin America	208.31 (117.18, 342.68)	221.17 (125.49, 358.56)	0.15 (0.1 to 0.19)	1.25 (0.43, 2.87)	1.30 (0.46, 3.03)	0.1 (0.06 to 0.14)
Western Europe	1062.63 (647.14, 1655.66)	1275.81 (736.72, 2031.32)	0.6 (0.59 to 0.61)	6.48 (2.43, 14.84)	7.73 (2.76, 17.78)	0.57 (0.57 to 0.58)
Western Sub-Saharan Africa	196.73 (119.26, 317.66)	283.84 (171.45, 458.41)	1.21 (1.18 to 1.25)	1.10 (0.40, 2.54)	1.59 (0.57, 3.66)	1.2 (1.17 to 1.23)

ASPR, age-standardized prevalence rate; ASYR, age-standardized YLD rate; AAPC, average annual percentage change; UI, uncertainty interval; CI, confidence interval.

Table S2. Prevalence and years lived with disability of primary infertility and secondary infertility due to PCOS and their average annual percentage changes from 1990 to 2021 at the global and regional levels.

**Location**	**Primary infertility**						**Secondary infertility**					
	**ASPR, per 100,000 (95% UI)**		**AAPC (95% CI)**	**ASYR, per 100,000 (95% UI)**		**AAPC (95% CI)**	**ASPR, per 100,000 (95% UI)**		**AAPC (95% CI)**	**ASYR, per 100,000 (95% UI)**		**AAPC (95% CI)**
	**1990**	**2021**	**1990-2021**	**1990**	**2021**	**1990-2021**	**1990**	**2021**	**1990-2021**	**1990**	**2021**	**1990-2021**
Global	148.68 (54.54, 296.90)	179.84 (52.94, 399.91)	0.62 (0.6 to 0.63)	1.11 (0.32, 2.80)	1.34 (0.32, 3.59)	0.61 (0.6 to 0.62)	326.86 (161.07, 561.56)	458.30 (219.74, 776.83)	1.12 (1.1 to 1.13)	1.66 (0.53, 4.08)	2.33 (0.73, 5.84)	1.12 (1.1 to 1.13)
**Socio-demographic index**												
High SDI	342.18 (123.73, 701.39)	395.18 (120.17, 840.01)	0.45 (0.43 to 0.48)	2.55 (0.71, 6.38)	2.94 (0.71, 7.73)	0.45 (0.42 to 0.48)	591.43 (249.19, 1090.16)	742.67 (307.14, 1334.34)	0.74 (0.73 to 0.76)	3.02 (0.87, 7.69)	3.79 (1.10, 9.63)	0.74 (0.72 to 0.76)
High-middle SDI	114.28 (38.86, 241.39)	167.60 (46.93, 395.18)	1.24 (1.23 to 1.26)	0.85 (0.23, 2.29)	1.25 (0.27, 3.46)	1.24 (1.22 to 1.26)	305.90 (156.43, 513.21)	481.42 (234.41, 822.33)	1.5 (1.48 to 1.51)	1.56 (0.51, 3.79)	2.45 (0.76, 6.21)	1.49 (1.48 to 1.51)

Middle SDI	121.50 (41.13, 257.17)	194.71 (50.68, 452.92)	1.53 (1.52 to 1.54)	0.90 (0.24, 2.37)	1.45 (0.31, 4.05)	1.53 (1.52 to 1.54)	308.36 (155.55, 520.68)	533.01 (254.66, 912.19)	1.79 (1.78 to 1.81)	1.57 (0.51, 3.92)	2.71 (0.83, 6.81)	1.8 (1.78 to 1.81)
Low-middle SDI	101.40 (42.58, 196.82)	135.88 (40.37, 301.86)	0.93 (0.89 to 0.96)	0.75 (0.24, 1.89)	1.01 (0.24, 2.72)	0.93 (0.88 to 0.96)	189.26 (96.56, 326.07)	324.49 (153.39, 559.05)	1.77 (1.75 to 1.79)	0.96 (0.31, 2.38)	1.65 (0.51, 4.13)	1.77 (1.75 to 1.79)
Low SDI	56.71 (20.31, 118.14)	72.97 (23.81, 157.68)	0.83 (0.8 to 0.86)	0.42 (0.11, 1.09)	0.54 (0.13, 1.44)	0.83 (0.8 to 0.86)	141.44 (72.65, 242.05)	207.28 (108.66, 348.81)	1.25 (1.23 to 1.27)	0.72 (0.23, 1.77)	1.05 (0.34, 2.64)	1.27 (1.24 to 1.29)
**Region**												
Andean Latin America	136.78 (64.47, 248.02)	189.41 (43.43, 470.47)	0.96 (0.72 to 1.09)	1.02 (0.30, 2.50)	1.41 (0.25, 4.29)	0.94 (0.7 to 1.08)	451.75 (260.72, 715.93)	799.28 (289.13, 1471.49)	2.01 (1.78 to 2.2)	2.30 (0.78, 5.34)	4.07 (1.07, 10.62)	2.04 (1.79 to 2.23)
Australasia	320.83 (87.14, 784.96)	358.53 (99.03, 867.12)	0.36 (0.35 to 0.37)	2.39 (0.50, 6.88)	2.67 (0.54, 7.69)	0.37 (0.35 to 0.38)	627.14 (165.80, 1399.30)	732.80 (200.17, 1614.92)	0.51 (0.49 to 0.52)	3.21 (0.61, 9.52)	3.75 (0.73, 10.85)	0.51 (0.49 to 0.52)
Caribbean	151.62 (53.91, 315.24)	164.18 (45.42, 373.04)	0.25 (0.23 to 0.26)	1.13 (0.31, 2.97)	1.22 (0.27, 3.31)	0.24 (0.22 to 0.26)	298.69 (147.64, 525.60)	390.11 (192.61, 692.51)	0.87 (0.85 to 0.88)	1.52 (0.48, 3.89)	1.98 (0.63, 4.95)	0.87 (0.85 to 0.88)
Central Asia	37.10	48.35	0.86	0.28	0.36	0.88	97.04	136.28	1.11	0.49	0.69	1.12

	(10.01, 84.46)	(11.50, 118.49)	(0.84 to 0.89)	(0.05, 0.77)	(0.06, 1.05)	(0.85 to 0.91)	(46.51, 171.91)	(64.48, 240.48)	(1.09 to 1.12)	(0.15, 1.25)	(0.21, 1.75)	(1.09 to 1.15)
Central Europe	17.95 (3.35, 46.72)	20.00 (3.60, 52.72)	0.35 (0.34 to 0.36)	0.13 (0.02, 0.41)	0.15 (0.02, 0.47)	0.36 (0.34 to 0.38)	53.16 (23.91, 98.30)	67.40 (32.16, 117.89)	0.76 (0.75 to 0.77)	0.27 (0.08, 0.70)	0.34 (0.10, 0.87)	0.75 (0.74 to 0.76)
Central Latin America	228.72 (74.95, 505.10)	228.17 (67.48, 540.83)	-0.02 (-0.05 to 0)	1.70 (0.44, 4.70)	1.70 (0.39, 4.89)	-0.03 (-0.06 to 0)	622.09 (299.73, 1089.25)	780.13 (393.95, 1340.42)	0.72 (0.68 to 0.75)	3.17 (0.98, 7.92)	3.97 (1.31, 9.77)	0.72 (0.67 to 0.76)
Central Sub-Saharan Africa	41.90 (9.59, 101.00)	63.35 (11.95, 156.98)	1.4 (1.3 to 1.5)	0.31 (0.04, 0.93)	0.47 (0.07, 1.38)	1.42 (1.31 to 1.53)	136.74 (66.34, 240.68)	207.53 (97.81, 367.51)	1.38 (1.35 to 1.42)	0.70 (0.20, 1.80)	1.05 (0.30, 2.71)	1.39 (1.35 to 1.43)
East Asia	45.68 (6.37, 130.76)	96.40 (15.45, 269.58)	2.45 (2.43 to 2.47)	0.34 (0.04, 1.11)	0.72 (0.09, 2.36)	2.45 (2.43 to 2.46)	292.00 (154.36, 491.21)	506.10 (260.01, 872.22)	1.8 (1.77 to 1.82)	1.49 (0.48, 3.72)	2.58 (0.80, 6.50)	1.8 (1.77 to 1.82)
Eastern Europe	27.27 (6.07, 63.60)	33.76 (7.09, 79.84)	0.7 (0.68 to 0.73)	0.20 (0.04, 0.57)	0.25 (0.04, 0.72)	0.7 (0.69 to 0.73)	53.69 (23.60, 100.50)	71.03 (31.48, 131.96)	0.92 (0.9 to 0.93)	0.27 (0.08, 0.72)	0.36 (0.10, 0.94)	0.92 (0.91 to 0.93)
Eastern Sub-Saharan Africa	49.17 (18.80, 100.11)	57.36 (17.40, 128.45)	0.53 (0.47 to	0.36 (0.10, 0.94)	0.42 (0.10, 1.17)	0.53 (0.46 to	154.84 (84.92, 259.76)	204.13 (110.54, 342.05)	0.91 (0.88 to	0.79 (0.26, 1.89)	1.04 (0.34, 2.57)	0.92 (0.89 to

			0.59)			0.59)			0.94)			0.95)
High-income Asia Pacific	319.17 (78.19, 805.10)	326.44 (79.28, 838.45)	0.07 (0.06 to 0.08)	2.38 (0.47, 7.14)	2.43 (0.46, 7.38)	0.07 (0.06 to 0.07)	996.39 (366.81, 1892.01)	1069.76 (380.67, 2040.51)	0.25 (0.23 to 0.28)	5.08 (1.29, 13.37)	5.45 (1.37, 14.57)	0.25 (0.22 to 0.27)
High-income North America	407.68 (114.26, 902.85)	491.59 (140.24, 1078.95)	0.6 (0.54 to 0.65)	3.04 (0.69, 7.95)	3.66 (0.85, 9.74)	0.6 (0.54 to 0.65)	526.46 (159.11, 1081.41)	749.28 (269.83, 1423.07)	1.14 (1.09 to 1.19)	2.69 (0.61, 7.42)	3.82 (0.98, 9.92)	1.14 (1.08 to 1.19)
North Africa and Middle East	272.93 (137.70, 478.24)	292.22 (90.54, 635.16)	0.22 (0.19 to 0.26)	2.03 (0.72, 4.69)	2.17 (0.53, 5.67)	0.22 (0.18 to 0.25)	347.95 (176.58, 601.61)	535.89 (224.19, 973.07)	1.41 (1.36 to 1.44)	1.77 (0.58, 4.38)	2.73 (0.77, 7.10)	1.43 (1.37 to 1.51)
Oceania	120.85 (25.64, 305.06)	149.62 (57.42, 299.76)	0.69 (0.67 to 0.7)	0.89 (0.15, 2.71)	1.11 (0.30, 2.90)	0.7 (0.68 to 0.71)	353.73 (156.62, 641.25)	488.01 (275.66, 817.96)	1.01 (0.99 to 1.03)	1.80 (0.53, 4.64)	2.48 (0.84, 6.08)	1.01 (0.99 to 1.03)
South Asia	88.98 (29.33, 183.03)	131.39 (34.32, 299.68)	1.26 (1.21 to 1.32)	0.66 (0.17, 1.72)	0.97 (0.21, 2.71)	1.26 (1.21 to 1.32)	155.81 (68.27, 280.36)	310.47 (141.27, 535.78)	2.27 (2.23 to 2.3)	0.79 (0.23, 2.03)	1.58 (0.48, 4.02)	2.27 (2.23 to 2.3)
Southeast Asia	191.45 (65.48, 411.77)	302.30 (83.39, 713.45)	1.48 (1.44 to 1.52)	1.42 (0.37, 3.72)	2.25 (0.51, 6.39)	1.5 (1.46 to 1.53)	391.84 (189.01, 681.22)	732.45 (343.11, 1268.87)	2.06 (2.03 to 2.09)	1.99 (0.61, 4.96)	3.73 (1.14, 9.39)	2.07 (2.04 to 2.09)
Southern Latin	148.72	231.44	1.44	1.10	1.72	1.46	276.54	429.64	1.47	1.41	2.19	1.47

America	(41.54, 351.13)	(63.96, 540.32)	(1.44 to 1.45)	(0.24, 3.03)	(0.39, 4.76)	(1.45 to 1.48)	(109.67, 527.60)	(172.29, 815.10)	(1.44 to 1.49)	(0.36, 3.69)	(0.58, 5.72)	(1.44 to 1.49)
Southern Sub-Saharan Africa	93.66 (24.70, 214.25)	110.84 (22.93, 280.96)	0.61 (0.55 to 0.66)	0.69 (0.14, 1.92)	0.82 (0.14, 2.43)	0.6 (0.55 to 0.64)	239.52 (116.74, 416.72)	291.01 (129.06, 520.55)	0.64 (0.62 to 0.67)	1.22 (0.38, 3.10)	1.48 (0.43, 3.85)	0.64 (0.61 to 0.66)
Tropical Latin America	81.16 (19.92, 184.11)	74.47 (17.67, 177.47)	-0.28 (-0.3 to -0.25)	0.60 (0.12, 1.64)	0.55 (0.11, 1.58)	-0.27 (-0.29 to -0.24)	127.15 (49.73, 248.59)	146.70 (59.97, 276.09)	0.51 (0.46 to 0.58)	0.65 (0.17, 1.74)	0.75 (0.21, 1.99)	0.52 (0.47 to 0.58)
Western Europe	446.29 (188.55, 864.95)	513.94 (154.84, 1099.87)	0.44 (0.43 to 0.45)	3.33 (1.04, 8.11)	3.84 (0.94, 10.00)	0.44 (0.43 to 0.45)	616.35 (283.40, 1099.51)	761.87 (299.12, 1393.84)	0.72 (0.7 to 0.74)	3.14 (0.95, 7.99)	3.89 (1.08, 10.20)	0.71 (0.7 to 0.73)
Western Sub-Saharan Africa	45.24 (16.45, 93.56)	64.33 (16.64, 151.91)	1.21 (1.14 to 1.29)	0.34 (0.09, 0.85)	0.48 (0.10, 1.34)	1.22 (1.14 to 1.29)	151.49 (81.07, 254.33)	219.51 (111.34, 377.30)	1.2 (1.18 to 1.23)	0.77 (0.25, 1.90)	1.12 (0.35, 2.76)	1.21 (1.18 to 1.24)

ASPR, age-standardized prevalence rate; ASYR, age-standardized YLD rate; AAPC, average annual percentage change; UI, uncertainty interval; CI, confidence interval.

Table S3. The Prevalence and YLDs of infertility (primary infertility and secondary infertility) due to PCOS in different countries in 1990 and 2021.

Location	Infertility				Primary infertility				Secondary infertility			
	Prevalence No. (95% UI)		YLDs No. (95% UI)		Prevalence No. (95% UI)		YLDs No. (95% UI)		Prevalence No. (95% UI)		YLDs No. (95% UI)	
	1990	2021	1990	2021	1990	2021	1990	2021	1990	2021	1990	2021
**Afghanistan**	7835 (4657, 12883)	36127 (22264, 56355)	44 (15, 103)	205 (75, 474)	1584 (296, 4116)	9100 (4201, 16910)	12 (1, 38)	68 (19, 168)	6250 (2934, 10892)	27026 (15679, 43399)	32 (9, 80)	137 (48, 331)
**Albania**	470 (249, 820)	409 (226, 692)	3 (1, 7)	2 (1, 6)	145 (27, 375)	135 (51, 280)	1 (0, 4)	1 (0, 3)	325 (130, 633)	275 (143, 488)	2 (0, 5)	1 (0, 4)
**Algeria**	35480 (21140, 56684)	98991 (55619, 161547)	213 (75, 491)	574 (200, 1385)	13750 (4864, 27486)	29430 (7499, 68919)	102 (25, 261)	219 (43, 624)	21730 (9574, 39905)	69561 (29088, 125837)	110 (31, 286)	354 (97, 918)
**American Samoa**	91 (52, 150)	112 (64, 189)	1 (0, 1)	1 (0, 2)	26 (6, 67)	29 (6, 75)	0 (0, 1)	0 (0, 1)	65 (28, 120)	83 (37, 153)	0 (0, 1)	0 (0, 1)
**Andorra**	158 (88, 255)	250 (136, 413)	1 (0, 2)	1 (0, 3)	67 (19, 147)	89 (23, 202)	0 (0, 1)	1 (0, 2)	91 (34, 173)	162 (63, 303)	0 (0, 1)	1 (0, 2)
**Angola**	3818 (2277, 6397)	23085 (13531, 37668)	22 (7, 53)	131 (44, 312)	1005 (195, 2550)	5970 (1166, 14719)	7 (1, 23)	44 (5, 129)	2813 (1275, 5057)	17115 (7720, 30722)	14 (4, 38)	87 (23, 225)
**Antigua and Barbuda**	76 (43, 121)	142 (82, 235)	0 (0, 1)	1 (0, 2)	26 (5, 61)	44 (9, 106)	0 (0, 1)	0 (0, 1)	50 (22, 92)	98 (44, 181)	0 (0, 1)	1 (0, 1)
**Argentina**	33125	75729	195	446	11533	26279 (7329,	85	195	21592 (8544,	49450	110	251

	(19139, 56391)	(43998, 126604)	(67, 458)	(157, 1038)	(3102, 27596)	60952)	(18, 243)	(42, 543)	41162)	(19710, 93390)	(27, 287)	(66, 666)
**Armenia**	1116 (638, 1829)	1219 (706, 2012)	6 (2, 15)	7 (2, 17)	241 (38, 667)	218 (68, 513)	2 (0, 6)	2 (0, 5)	875 (413, 1536)	1001 (558, 1694)	4 (1, 12)	5 (2, 13)
**Australia**	41110 (15043, 78951)	63368 (23280, 125018)	242 (62, 612)	370 (94, 944)	13590 (3687, 33378)	19676 (5353, 47493)	101 (20, 287)	147 (28, 428)	27520 (7328, 60949)	43692 (11616, 98789)	141 (27, 414)	224 (44, 664)
**Austria**	27986 (16560, 44523)	29858 (17153, 47841)	166 (61, 378)	174 (61, 410)	9765 (2756, 21801)	9052 (2414, 21204)	73 (16, 200)	68 (14, 185)	18221 (7561, 32762)	20805 (8853, 36960)	93 (26, 242)	106 (30, 274)
**Azerbaijan**	2498 (1484, 4084)	5437 (3205, 8712)	14 (4, 34)	31 (10, 75)	798 (153, 1922)	1488 (286, 3719)	6 (0, 17)	11 (1, 34)	1701 (725, 3146)	3950 (1808, 7026)	9 (2, 24)	20 (5, 52)
**Bahamas**	422 (241, 693)	714 (406, 1201)	2 (1, 6)	4 (1, 10)	147 (32, 346)	229 (49, 549)	1 (0, 3)	2 (0, 5)	275 (119, 514)	485 (216, 900)	1 (0, 4)	2 (1, 6)
**Bahrain**	1007 (587, 1595)	3124 (1817, 5035)	6 (2, 14)	18 (6, 42)	356 (95, 795)	978 (254, 2274)	3 (1, 7)	7 (1, 20)	650 (256, 1212)	2147 (900, 3954)	3 (1, 9)	11 (3, 28)
**Bangladesh**	36605 (22231, 59280)	108080 (65682, 171742)	221 (72, 524)	635 (215, 1466)	15141 (7746, 25837)	35696 (18403, 61274)	113 (30, 280)	266 (76, 662)	21464 (12038, 35928)	72384 (42357, 117737)	108 (29, 269)	369 (112, 905)
**Barbados**	395 (226, 648)	456 (263, 743)	2 (1, 5)	3 (1, 6)	129 (28, 310)	139 (30, 339)	1 (0, 3)	1 (0, 3)	266 (117, 484)	316 (142, 578)	1 (0, 4)	2 (0, 4)
**Belarus**	2017 (1132,	2184 (1267, 3669)	12 (3, 29)	13 (4, 32)	639 (110, 1584)	601 (105, 1497)	5 (0, 15)	4 (0, 14)	1378 (593, 2594)	1583 (728,	7 (1, 19)	8 (2, 22)

	3389)									2885)		
**Belgium**	27222 (16265, 43391)	33341 (19827, 53751)	166 (61, 384)	197 (71, 463)	11428 (5798, 20699)	11564 (3338, 26069)	86 (29, 206)	86 (20, 237)	15794 (8147, 27000)	21777 (9232, 39198)	80 (26, 201)	111 (31, 289)
**Belize**	186 (108, 302)	745 (426, 1219)	1 (0, 3)	4 (2, 11)	86 (35, 163)	289 (69, 672)	1 (0, 2)	2 (0, 6)	100 (49, 176)	456 (187, 854)	1 (0, 1)	2 (1, 6)
**Benin**	2004 (1164, 3264)	10566 (6330, 16750)	11 (4, 27)	59 (20, 139)	423 (77, 1061)	2316 (944, 4630)	3 (0, 9)	17 (4, 43)	1581 (698, 2750)	8250 (4682, 13540)	8 (2, 21)	42 (13, 103)
**Bermuda**	118 (68, 196)	97 (55, 165)	1 (0, 2)	1 (0, 1)	35 (8, 87)	27 (6, 68)	0 (0, 1)	0 (0, 1)	83 (38, 149)	70 (32, 129)	0 (0, 1)	0 (0, 1)
**Bhutan**	306 (183, 497)	779 (456, 1249)	2 (1, 4)	5 (2, 10)	108 (26, 248)	256 (61, 600)	1 (0, 2)	2 (0, 6)	198 (77, 368)	523 (211, 955)	1 (0, 3)	3 (1, 7)
**Bolivia (Plurinational State of)**	9191 (5387, 14536)	28322 (12737, 48437)	52 (18, 124)	158 (48, 381)	2268 (955, 4338)	5979 (1408, 14734)	17 (4, 45)	44 (8, 133)	6924 (3801, 11328)	22343 (7717, 42506)	35 (12, 85)	114 (28, 307)
**Bosnia and Herzegovina**	554 (291, 973)	517 (286, 887)	3 (1, 8)	3 (1, 7)	122 (19, 341)	103 (17, 288)	1 (0, 3)	1 (0, 3)	432 (192, 810)	414 (198, 744)	2 (0, 6)	2 (0, 6)
**Botswana**	805 (469, 1292)	2817 (1585, 4564)	4 (1, 11)	16 (5, 37)	152 (46, 339)	508 (83, 1455)	1 (0, 3)	4 (0, 13)	654 (380, 1072)	2309 (1143, 3964)	3 (1, 8)	12 (3, 30)
**Brazil**	82185 (46079, 134940)	130648 (75024, 211241)	495 (171, 1138)	765 (271, 1776)	33158 (7991, 74771)	42339 (10240, 101280)	246 (49, 669)	315 (61, 895)	49027 (18438, 96645)	88309 (36965, 164521)	250 (64, 677)	450 (126, 1188)
**Brunei Darussalam**	458 (180, 875)	1251 (488, 2363)	3 (1, 7)	7 (2, 18)	139 (31, 370)	299 (69, 803)	1 (0, 3)	2 (0, 7)	319 (97, 667)	952 (299, 1964)	2 (0, 5)	5 (1, 13)

**Bulgaria**	1328 (722, 2287)	1124 (628, 1898)	7 (2, 19)	6 (2, 15)	274 (44, 767)	208 (33, 597)	2 (0, 7)	2 (0, 5)	1054 (494, 1941)	916 (448, 1647)	5 (1, 14)	5 (1, 12)
**Burkina Faso**	3517 (2121, 5717)	15140 (8682, 24667)	21 (7, 50)	85 (27, 202)	1292 (599, 2382)	3608 (717, 8706)	10 (2, 25)	27 (3, 82)	2225 (1164, 3836)	11532 (5003, 20461)	11 (3, 28)	59 (16, 152)
**Burundi**	2167 (1283, 3486)	5348 (3161, 8745)	13 (4, 31)	30 (10, 73)	649 (238, 1297)	1203 (466, 2453)	5 (1, 14)	9 (1, 25)	1518 (819, 2575)	4145 (2339, 6930)	8 (2, 19)	21 (6, 52)
**Cabo Verde**	161 (97, 263)	510 (307, 824)	1 (0, 2)	3 (1, 7)	39 (8, 98)	100 (21, 258)	0 (0, 1)	1 (0, 2)	121 (56, 215)	410 (201, 700)	1 (0, 2)	2 (1, 5)
**Cambodia**	10994 (6401, 18524)	34119 (19967, 56227)	63 (20, 148)	191 (65, 454)	2845 (531, 7469)	7408 (1465, 19647)	21 (3, 64)	55 (8, 174)	8150 (3956, 14411)	26711 (13433, 45775)	42 (12, 106)	136 (41, 341)
**Cameroon**	6636 (3989, 10532)	29676 (17566, 48194)	39 (13, 93)	173 (60, 405)	2062 (863, 3980)	9340 (2205, 21772)	15 (4, 39)	69 (12, 194)	4574 (2480, 7711)	20336 (8625, 37792)	23 (7, 58)	103 (26, 279)
**Canada**	37406 (20263, 62055)	51131 (26892, 85433)	227 (78, 518)	309 (105, 709)	15431 (4100, 34802)	20521 (5565, 46680)	114 (24, 306)	153 (31, 412)	21975 (6822, 43784)	30609 (9447, 61786)	112 (24, 302)	156 (34, 429)
**Central African Republic**	1401 (830, 2289)	3308 (1982, 5307)	8 (3, 20)	19 (7, 46)	456 (222, 821)	1057 (260, 2393)	3 (1, 8)	8 (1, 23)	945 (515, 1609)	2251 (951, 4116)	5 (1, 12)	12 (3, 30)
**Chad**	1778 (989, 2936)	8036 (4769, 13252)	10 (3, 24)	44 (14, 106)	322 (54, 845)	1521 (240, 4034)	2 (0, 8)	11 (1, 36)	1456 (633, 2574)	6515 (3116, 11280)	7 (2, 20)	33 (9, 85)
**Chile**	16093	35638	96 (33,	210	6024 (1754,	11769 (3209,	45	88 (18,	10069 (3862,	23869	51 (13,	122

	(9403, 26954)	(20482, 59554)	222)	(71, 488)	14066)	27507)	(10, 123)	252)	19376)	(9680, 44947)	134)	(33, 320)
**China**	1080023 (626242, 1739107)	1916743 (1083224, 3169296)	5867 (1979, 13608)	10349 (3520, 24592)	159275 (22078, 451116)	256404 (42330, 733013)	1181 (136, 3837)	1904 (255, 6343)	920748 (485311, 1543676)	1660339 (865836, 2847002)	4686 (1505, 11723)	8445 (2634, 21199)
**Colombia**	46912 (28584, 72768)	92922 (58272, 141917)	285 (105, 666)	551 (205, 1246)	19013 (9550, 32710)	33085 (16063, 58900)	143 (47, 353)	247 (80, 610)	27899 (15555, 46137)	59837 (34986, 94469)	142 (47, 348)	304 (103, 714)
**Comoros**	278 (165, 459)	610 (365, 994)	2 (1, 4)	4 (1, 8)	108 (29, 232)	220 (60, 492)	1 (0, 2)	2 (0, 5)	170 (65, 330)	390 (157, 743)	1 (0, 2)	2 (0, 5)
**Congo**	1292 (753, 2112)	4574 (2600, 7499)	7 (2, 17)	25 (8, 60)	279 (45, 742)	870 (135, 2367)	2 (0, 7)	7 (1, 20)	1013 (481, 1794)	3705 (1766, 6460)	5 (1, 14)	19 (5, 48)
**Cook Islands**	35 (20, 59)	47 (27, 79)	0 (0, 0)	0 (0, 1)	10 (2, 24)	12 (3, 30)	0 (0, 0)	0 (0, 0)	25 (11, 47)	35 (16, 64)	0 (0, 0)	0 (0, 0)
**Costa Rica**	5948 (3347, 10036)	12726 (7439, 20735)	34 (12, 81)	72 (25, 174)	1637 (315, 4325)	3105 (617, 8145)	12 (2, 37)	23 (3, 71)	4310 (1877, 7922)	9622 (4467, 17043)	22 (6, 55)	49 (15, 126)
**Coted'Ivoire**	5167 (3152, 8373)	21004 (12675, 33738)	29 (10, 68)	117 (39, 278)	1304 (535, 2587)	4490 (894, 11456)	10 (2, 25)	33 (4, 99)	3863 (2176, 6454)	16514 (8066, 28401)	19 (6, 48)	84 (24, 215)
**Croatia**	718 (393, 1242)	697 (383, 1182)	4 (1, 10)	4 (1, 10)	144 (23, 406)	132 (22, 371)	1 (0, 4)	1 (0, 3)	573 (267, 1062)	565 (272, 1006)	3 (1, 8)	3 (1, 8)
**Cuba**	15306 (8683, 24837)	15451 (8888, 25765)	90 (31, 214)	89 (31, 214)	5147 (1102, 12361)	4626 (990, 11240)	38 (6, 112)	34 (5, 100)	10159 (4385, 18881)	10826 (4937, 19968)	52 (15, 135)	55 (16, 140)

**Cyprus**	1654 (942, 2690)	4408 (2442, 7154)	10 (4, 24)	26 (9, 63)	714 (209, 1542)	1631 (422, 3642)	5 (1, 15)	12 (3, 34)	940 (350, 1768)	2776 (1089, 5191)	5 (1, 13)	14 (4, 38)
**Czechia**	1586 (857, 2711)	1729 (971, 2894)	9 (2, 23)	9 (3, 24)	302 (46, 873)	300 (47, 848)	2 (0, 8)	2 (0, 8)	1284 (608, 2318)	1429 (698, 2552)	7 (1, 18)	7 (2, 19)
**Democratic People's Republic of Korea**	19165 (10766, 31905)	25000 (14246, 42074)	106 (34, 265)	136 (43, 332)	3536 (514, 9853)	4016 (628, 11234)	26 (2, 90)	30 (2, 100)	15629 (7764, 27620)	20984 (10785, 36567)	80 (23, 206)	106 (32, 271)
**Democratic Republic of the Congo**	14899 (8525, 24752)	54250 (30765, 88990)	85 (27, 209)	309 (103, 729)	3947 (706, 9701)	14296 (2442, 35350)	30 (2, 93)	106 (11, 316)	10952 (4958, 19718)	39954 (18035, 72339)	56 (14, 148)	203 (54, 529)
**Denmark**	8976 (4724, 15693)	12146 (5859, 20993)	58 (19, 139)	77 (24, 179)	5036 (1906, 10387)	6230 (1638, 13613)	38 (10, 98)	46 (10, 123)	3940 (1618, 7733)	5916 (1896, 12579)	20 (5, 54)	30 (6, 85)
**Djibouti**	207 (124, 338)	1156 (700, 1909)	1 (0, 3)	6 (2, 15)	49 (10, 123)	240 (52, 615)	0 (0, 1)	2 (0, 6)	158 (74, 282)	916 (448, 1601)	1 (0, 2)	5 (1, 12)
**Dominica**	77 (43, 128)	100 (55, 165)	0 (0, 1)	1 (0, 1)	28 (6, 66)	33 (7, 79)	0 (0, 1)	0 (0, 1)	49 (20, 92)	67 (29, 124)	0 (0, 1)	0 (0, 1)
**Dominican Republic**	7283 (4285, 11513)	17549 (9909, 29102)	44 (15, 103)	102 (34, 245)	2973 (1377, 5418)	5215 (1071, 13046)	22 (7, 54)	39 (6, 118)	4310 (2342, 7317)	12333 (5605, 22482)	22 (7, 54)	63 (18, 165)
**Ecuador**	20111 (12466, 30172)	50105 (21435, 86820)	114 (40, 258)	281 (81, 694)	4826 (2227, 8718)	10697 (2520, 26853)	36 (11, 88)	80 (14, 237)	15285 (8897, 24016)	39407 (12780, 74726)	78 (26, 185)	201 (47, 538)

**Egypt**	98321 (60153, 155069)	241586 (138811, 392199)	623 (230, 1392)	1454 (514, 3367)	52625 (30343, 84217)	93015 (26897, 203171)	392 (143, 882)	692 (149, 1815)	45696 (24674, 78367)	148571 (56676, 278387)	231 (71, 553)	762 (190, 1991)
**El Salvador**	7475 (4410, 12145)	14149 (8019, 23490)	43 (15, 102)	80 (27, 189)	2348 (940, 4686)	3343 (673, 8522)	18 (5, 46)	25 (4, 75)	5128 (2714, 8857)	10806 (4423, 19785)	26 (8, 63)	55 (16, 142)
**Equatorial Guinea**	187 (111, 308)	1630 (934, 2614)	1 (0, 3)	9 (3, 22)	49 (10, 123)	408 (79, 1026)	0 (0, 1)	3 (0, 9)	138 (61, 248)	1222 (560, 2178)	1 (0, 2)	6 (2, 16)
**Eritrea**	1213 (736, 1968)	3905 (2326, 6353)	7 (2, 17)	22 (8, 54)	397 (192, 725)	1068 (246, 2531)	3 (1, 8)	8 (1, 24)	815 (462, 1363)	2836 (1285, 5079)	4 (1, 11)	14 (4, 38)
**Estonia**	317 (176, 532)	323 (184, 547)	2 (0, 5)	2 (1, 5)	101 (18, 246)	90 (16, 225)	1 (0, 2)	1 (0, 2)	216 (93, 404)	232 (106, 431)	1 (0, 3)	1 (0, 3)
**Eswatini**	654 (349, 1110)	1331 (742, 2169)	4 (1, 9)	8 (3, 19)	217 (42, 537)	406 (83, 1004)	2 (0, 5)	3 (0, 9)	437 (172, 830)	925 (378, 1701)	2 (1, 6)	5 (1, 12)
**Ethiopia**	17556 (10532, 28455)	65877 (38510, 107558)	101 (35, 234)	379 (135, 889)	4977 (1104, 11956)	18599 (4071, 44728)	37 (6, 110)	138 (24, 399)	12580 (5724, 22542)	47278 (21554, 85304)	64 (19, 163)	241 (68, 620)
**Fiji**	1231 (696, 2052)	2125 (1222, 3617)	7 (2, 17)	12 (4, 28)	389 (87, 932)	630 (147, 1559)	3 (0, 8)	5 (1, 14)	842 (346, 1579)	1495 (641, 2821)	4 (1, 11)	8 (2, 20)
**Finland**	11081 (6528, 17838)	12917 (7132, 21385)	68 (24, 157)	78 (27, 180)	4862 (2303, 8988)	5294 (1462, 11685)	36 (12, 85)	39 (9, 104)	6219 (3034, 10849)	7623 (2902, 14414)	32 (10, 79)	39 (10, 106)
**France**	137880	168051	869	1040	70712	76371	528	570	67168	91680	341	470

	(81739, 219417)	(95483, 276117)	(318, 1958)	(373, 2500)	(34946, 124898)	(24924, 163165)	(177, 1227)	(141, 1492)	(31306, 120408)	(32659, 172822)	(100, 844)	(117, 1279)
**Gabon**	588 (348, 948)	1967 (1156, 3198)	3 (1, 8)	11 (4, 26)	159 (32, 395)	487 (97, 1223)	1 (0, 4)	4 (0, 11)	430 (186, 758)	1481 (665, 2642)	2 (1, 6)	8 (2, 20)
**Gambia**	435 (261, 705)	1758 (1038, 2842)	2 (1, 6)	10 (3, 23)	111 (23, 271)	430 (85, 1066)	1 (0, 3)	3 (0, 10)	325 (146, 575)	1328 (606, 2336)	2 (0, 4)	7 (2, 17)
**Georgia**	2203 (1258, 3617)	2078 (1162, 3388)	13 (4, 31)	12 (4, 28)	561 (102, 1447)	463 (86, 1200)	4 (0, 13)	3 (0, 11)	1642 (750, 2962)	1615 (737, 2787)	8 (2, 22)	8 (2, 21)
**Germany**	151139 (83988, 247728)	174556 (91065, 291108)	952 (337, 2228)	1050 (346, 2433)	76806 (31311, 149677)	66977 (16662, 152875)	574 (162, 1389)	500 (92, 1367)	74332 (34170, 137830)	107579 (40392, 206866)	378 (105, 991)	549 (144, 1463)
**Ghana**	6859 (4216, 11099)	26742 (15912, 43277)	38 (13, 91)	149 (48, 346)	1562 (707, 2985)	5573 (1028, 14263)	12 (2, 31)	41 (4, 129)	5297 (3044, 8683)	21169 (10065, 36655)	27 (8, 66)	108 (30, 280)
**Greece**	25516 (13905, 41422)	26679 (14654, 43494)	155 (53, 371)	159 (53, 380)	10547 (2999, 23267)	9448 (2448, 21705)	79 (17, 215)	70 (14, 192)	14969 (5605, 28292)	17231 (6903, 31882)	77 (19, 210)	88 (24, 233)
**Greenland**	67 (37, 110)	72 (39, 119)	0 (0, 1)	0 (0, 1)	31 (8, 67)	31 (8, 68)	0 (0, 1)	0 (0, 1)	36 (10, 74)	41 (13, 83)	0 (0, 1)	0 (0, 1)
**Grenada**	78 (43, 127)	140 (80, 230)	0 (0, 1)	1 (0, 2)	28 (6, 65)	47 (10, 112)	0 (0, 1)	0 (0, 1)	50 (20, 92)	93 (41, 172)	0 (0, 1)	0 (0, 1)
**Guam**	296 (169, 493)	398 (231, 668)	2 (1, 4)	2 (1, 5)	81 (18, 207)	103 (23, 261)	1 (0, 2)	1 (0, 2)	216 (95, 389)	294 (130, 538)	1 (0, 3)	2 (0, 4)

**Guatemala**	9155 (5444, 14602)	32272 (18259, 54277)	51 (18, 120)	178 (61, 420)	1937 (748, 3921)	6302 (1105, 17526)	15 (3, 38)	47 (6, 154)	7218 (4186, 11831)	25970 (12385, 46290)	37 (12, 88)	132 (40, 337)
**Guinea**	2335 (1375, 3814)	8693 (5343, 13968)	13 (4, 32)	48 (16, 117)	484 (97, 1223)	1723 (664, 3422)	4 (0, 11)	13 (3, 34)	1851 (879, 3209)	6970 (4036, 11334)	9 (2, 25)	35 (11, 87)
**Guinea-Bissau**	393 (224, 660)	1374 (822, 2263)	2 (1, 5)	8 (3, 19)	101 (21, 248)	333 (68, 819)	1 (0, 2)	2 (0, 8)	292 (120, 531)	1040 (479, 1837)	1 (0, 4)	5 (1, 14)
**Guyana**	861 (494, 1383)	1201 (686, 1965)	5 (2, 12)	7 (2, 17)	299 (65, 703)	386 (84, 911)	2 (0, 6)	3 (0, 8)	561 (238, 1040)	815 (355, 1505)	3 (1, 8)	4 (1, 11)
**Haiti**	4832 (2921, 7768)	13216 (7858, 21006)	29 (10, 68)	75 (25, 173)	1804 (811, 3283)	3345 (1327, 6664)	13 (4, 34)	25 (6, 66)	3028 (1629, 5272)	9871 (5589, 16406)	15 (5, 39)	50 (16, 120)
**Honduras**	5264 (2978, 8876)	21109 (12071, 35002)	30 (10, 73)	118 (40, 280)	1183 (197, 3429)	4357 (769, 12062)	9 (1, 29)	32 (4, 105)	4080 (1879, 7419)	16752 (7899, 29946)	21 (6, 55)	85 (25, 220)
**Hungary**	1637 (877, 2762)	1643 (914, 2788)	9 (3, 22)	9 (3, 22)	325 (51, 940)	310 (52, 885)	2 (0, 9)	2 (0, 8)	1312 (609, 2363)	1333 (638, 2388)	7 (2, 18)	7 (2, 18)
**Iceland**	686 (383, 1113)	1042 (575, 1682)	4 (1, 10)	6 (2, 15)	298 (86, 641)	406 (106, 910)	2 (0, 6)	3 (1, 8)	388 (147, 736)	636 (246, 1188)	2 (1, 5)	3 (1, 9)
**India**	521799 (326655, 807165)	1887785 (1161559, 2952210)	3123 (1189, 7161)	10828 (4000, 24903)	202488 (64858, 411372)	531787 (120125, 1267591)	1500 (386, 3876)	3936 (774, 11365)	319311 (134857, 580953)	1355997 (611328, 2336178)	1623 (456, 4226)	6892 (2075, 17623)
**Indonesia**	289056	832688	1703	4781	99857	232480	740	1725	189199	600208	963	3056

	(165949, 469903)	(479864, 1362166)	(598, 3838)	(1667, 11057)	(25068, 224912)	(53200, 578915)	(155, 2043)	(334, 5000)	(81618, 342915)	(276401, 1052730)	(277, 2499)	(900, 7821)
**Iran (Islamic Republic of)**	85305 (50185, 136666)	204001 (119432, 324971)	549 (203, 1233)	1279 (466, 2884)	48580 (17582, 92209)	101672 (35076, 201181)	362 (102, 873)	758 (206, 1913)	36725 (11070, 77114)	102329 (34935, 205654)	187 (42, 519)	522 (127, 1390)
**Iraq**	29168 (17043, 47203)	86175 (49165, 139148)	173 (62, 408)	506 (178, 1165)	10558 (2725, 23997)	28874 (7520, 65826)	78 (16, 213)	214 (43, 592)	18611 (7163, 35246)	57301 (22912, 106431)	95 (24, 250)	292 (78, 753)
**Ireland**	8398 (4595, 13782)	13985 (7657, 22995)	51 (17, 121)	83 (28, 193)	3570 (1010, 7925)	5059 (1322, 11768)	27 (6, 72)	38 (8, 104)	4828 (1831, 9187)	8926 (3574, 16483)	25 (6, 67)	46 (13, 121)
**Israel**	9858 (5452, 16492)	23064 (12276, 38696)	60 (19, 143)	138 (45, 320)	3998 (1091, 9053)	8642 (2179, 19857)	30 (6, 80)	65 (13, 179)	5860 (2309, 11290)	14422 (5582, 27024)	30 (8, 79)	74 (19, 196)
**Italy**	301863 (139620, 530126)	283581 (147609, 461430)	1769 (555, 4330)	1656 (568, 3944)	95707 (21684, 241430)	86851 (22190, 200043)	715 (138, 2162)	650 (135, 1790)	206156 (74615, 403749)	196730 (78895, 360733)	1055 (263, 2796)	1005 (277, 2622)
**Jamaica**	2716 (1530, 4335)	4621 (2677, 7575)	16 (5, 38)	27 (9, 63)	973 (216, 2199)	1474 (324, 3465)	7 (1, 21)	11 (2, 31)	1743 (732, 3292)	3146 (1406, 5768)	9 (2, 24)	16 (4, 41)
**Japan**	530158 (244670, 917557)	435775 (195651, 755251)	2972 (899, 7090)	2419 (723, 5843)	115309 (28216, 291947)	84546 (20249, 215672)	859 (170, 2589)	630 (119, 1903)	414850 (160830, 773971)	351229 (132736, 652545)	2113 (553, 5505)	1789 (469, 4696)
**Jordan**	5490 (3275, 8688)	25341 (15199, 39613)	34 (12, 76)	150 (54, 351)	2522 (1024, 4765)	8658 (3895, 16056)	19 (6, 45)	65 (20, 157)	2968 (1281, 5526)	16683 (9105, 27458)	15 (4, 39)	85 (28, 210)

**Kazakhstan**	6028 (3509, 9982)	9410 (5399, 15424)	35 (11, 85)	53 (17, 129)	1924 (665, 3956)	2388 (434, 6075)	14 (3, 39)	18 (2, 56)	4104 (2138, 7217)	7022 (3185, 12488)	21 (6, 52)	36 (10, 93)
**Kenya**	12100 (6816, 19841)	39724 (23886, 64135)	69 (24, 161)	223 (80, 512)	3276 (605, 8195)	8810 (1721, 22531)	24 (4, 73)	65 (11, 196)	8824 (4007, 15841)	30914 (15024, 52975)	45 (13, 115)	158 (48, 402)
**Kiribati**	99 (57, 164)	241 (141, 395)	1 (0, 1)	1 (0, 3)	28 (6, 71)	65 (14, 166)	0 (0, 1)	0 (0, 1)	71 (31, 132)	176 (78, 323)	0 (0, 1)	1 (0, 2)
**Kuwait**	3844 (2252, 6196)	15825 (9301, 25422)	23 (8, 52)	91 (33, 215)	1316 (360, 2963)	4453 (1156, 10605)	10 (2, 27)	33 (7, 94)	2527 (1020, 4680)	11372 (4922, 20106)	13 (3, 33)	58 (17, 151)
**Kyrgyzstan**	1444 (852, 2333)	2786 (1661, 4522)	8 (3, 20)	16 (5, 37)	372 (66, 942)	644 (118, 1679)	3 (0, 9)	5 (0, 15)	1072 (492, 1904)	2142 (1013, 3806)	5 (1, 14)	11 (3, 28)
**Lao People's Democratic Republic**	4718 (2707, 7898)	17841 (10620, 29619)	28 (9, 65)	103 (36, 241)	1604 (348, 3773)	5303 (1252, 13314)	12 (2, 33)	39 (7, 117)	3114 (1426, 5605)	12538 (5706, 22195)	16 (4, 40)	64 (18, 165)
**Latvia**	528 (296, 895)	412 (232, 686)	3 (1, 8)	2 (1, 6)	173 (58, 347)	105 (17, 264)	1 (0, 3)	1 (0, 3)	355 (182, 634)	307 (145, 557)	2 (0, 5)	2 (0, 4)
**Lebanon**	5461 (3186, 8862)	14138 (8378, 22988)	32 (11, 74)	81 (28, 188)	1617 (392, 3819)	3674 (854, 8973)	12 (2, 35)	27 (5, 79)	3844 (1618, 6948)	10464 (4643, 18533)	20 (5, 50)	53 (15, 136)
**Lesotho**	933 (538, 1514)	1884 (1082, 3074)	5 (2, 13)	11 (4, 26)	278 (50, 677)	565 (101, 1398)	2 (0, 6)	4 (1, 13)	655 (287, 1195)	1318 (586, 2408)	3 (1, 9)	7 (2, 17)
**Liberia**	1102 (662,	4030 (2404,	6 (2,	22 (7,	327 (140,	841 (157,	2 (1,	6 (1,	775 (423,	3189	4 (1,	16 (5,

	1764)	6561)	15)	54)	619)	2159)	6)	19)	1317)	(1536, 5518)	10)	41)
**Libya**	7204 (4069, 11616)	17419 (9954, 28399)	43 (15, 101)	103 (36, 243)	2739 (970, 5575)	5912 (1605, 13288)	20 (5, 52)	44 (9, 121)	4464 (1937, 8192)	11507 (4649, 21404)	23 (6, 58)	59 (16, 153)
**Lithuania**	694 (399, 1164)	587 (331, 984)	4 (1, 10)	3 (1, 8)	204 (75, 408)	140 (22, 359)	2 (0, 4)	1 (0, 3)	491 (258, 866)	446 (213, 806)	2 (1, 6)	2 (1, 6)
**Luxembourg**	1003 (555, 1641)	1986 (1101, 3273)	6 (2, 14)	12 (4, 28)	430 (122, 916)	762 (207, 1716)	3 (1, 9)	6 (1, 16)	573 (214, 1097)	1224 (472, 2324)	3 (1, 8)	6 (2, 17)
**Madagascar**	5213 (3130, 8351)	16202 (9863, 26519)	31 (10, 74)	95 (33, 222)	1648 (733, 3139)	5562 (1415, 12597)	12 (3, 32)	41 (7, 115)	3565 (1927, 6049)	10640 (4389, 19608)	18 (5, 45)	54 (13, 142)
**Malawi**	5957 (3633, 9539)	13079 (7625, 20844)	34 (12, 78)	72 (24, 171)	1516 (668, 2797)	2359 (805, 4962)	11 (3, 28)	17 (3, 51)	4441 (2525, 7405)	10720 (6143, 17406)	22 (7, 54)	54 (17, 132)
**Malaysia**	39382 (22926, 64561)	124874 (72927, 204267)	221 (73, 515)	693 (244, 1592)	8891 (1825, 23567)	24820 (5277, 64508)	66 (10, 209)	184 (31, 569)	30491 (15493, 52598)	100054 (50302, 173975)	155 (47, 383)	509 (160, 1275)
**Maldives**	270 (151, 434)	1426 (870, 2291)	2 (1, 4)	8 (3, 19)	109 (28, 232)	447 (225, 818)	1 (0, 2)	3 (1, 8)	162 (68, 297)	979 (575, 1601)	1 (0, 2)	5 (2, 12)
**Mali**	2765 (1672, 4430)	12658 (7731, 20511)	16 (5, 37)	72 (26, 170)	829 (398, 1507)	3192 (1317, 6301)	6 (1, 16)	24 (6, 59)	1936 (1082, 3208)	9466 (5320, 15688)	10 (3, 25)	48 (16, 121)
**Malta**	913 (512, 1489)	1229 (674, 2014)	6 (2, 13)	7 (2, 17)	359 (99, 816)	450 (116, 1014)	3 (1, 7)	3 (1, 9)	554 (216, 1022)	780 (301, 1452)	3 (1, 8)	4 (1, 11)

**Marshall Islands**	44 (25, 73)	108 (61, 181)	0 (0, 1)	1 (0, 1)	13 (3, 33)	29 (6, 74)	0 (0, 0)	0 (0, 1)	31 (13, 58)	79 (35, 145)	0 (0, 0)	0 (0, 1)
**Mauritania**	1110 (653, 1826)	3648 (2151, 5927)	6 (2, 15)	21 (7, 50)	348 (115, 715)	1109 (241, 2626)	3 (1, 7)	8 (1, 24)	762 (349, 1384)	2539 (1060, 4675)	4 (1, 10)	13 (3, 34)
**Mauritius**	2642 (1561, 4444)	4241 (2510, 6991)	15 (5, 35)	24 (8, 57)	770 (173, 1909)	1072 (257, 2732)	6 (1, 17)	8 (1, 23)	1872 (874, 3323)	3169 (1507, 5574)	10 (3, 24)	16 (5, 41)
**Mexico**	233092 (125389, 383863)	433619 (258917, 692240)	1339 (448, 3189)	2410 (881, 5617)	64306 (15229, 158821)	86402 (16944, 224503)	478 (90, 1405)	642 (106, 2006)	168786 (68531, 313876)	347217 (169132, 604164)	862 (236, 2261)	1768 (563, 4410)
**Micronesia (Federated States of)**	130 (72, 218)	205 (117, 341)	1 (0, 2)	1 (0, 3)	38 (8, 97)	57 (12, 145)	0 (0, 1)	0 (0, 1)	92 (39, 173)	149 (64, 277)	0 (0, 1)	1 (0, 2)
**Monaco**	78 (43, 127)	89 (49, 147)	0 (0, 1)	1 (0, 1)	30 (8, 66)	33 (9, 75)	0 (0, 1)	0 (0, 1)	48 (19, 90)	57 (22, 106)	0 (0, 1)	0 (0, 1)
**Mongolia**	653 (364, 1068)	1495 (857, 2451)	4 (1, 9)	9 (3, 21)	189 (33, 458)	364 (65, 941)	1 (0, 4)	3 (0, 9)	465 (201, 861)	1132 (531, 2009)	2 (1, 7)	6 (2, 15)
**Montenegro**	101 (54, 175)	115 (63, 194)	1 (0, 1)	1 (0, 2)	22 (4, 62)	23 (4, 66)	0 (0, 1)	0 (0, 1)	79 (36, 146)	91 (43, 164)	0 (0, 1)	0 (0, 1)
**Morocco**	37095 (22563, 58693)	79303 (46662, 130047)	232 (84, 529)	472 (169, 1107)	18435 (9637, 31002)	29014 (7978, 65173)	138 (45, 317)	216 (47, 587)	18660 (10019, 32077)	50290 (19220, 95728)	95 (29, 233)	256 (66, 673)
**Mozambique**	5980 (3565,	22085 (13274,	34 (11, 82)	125 (43,	1551 (333, 3821)	5915 (1282, 14460)	11 (1, 35)	43 (6, 131)	4429 (2039, 7990)	16170 (7291,	22 (6, 60)	82 (22, 209)

	9962)	35788)		290)						28471)		
**Myanmar**	47656 (27641, 79511)	131154 (80603, 206295)	281 (90, 665)	764 (273, 1761)	16429 (3620, 39823)	41558 (19849, 74850)	122 (18, 357)	310 (100, 749)	31227 (14000, 56738)	89596 (53470, 145396)	159 (43, 419)	454 (155, 1065)
**Namibia**	830 (489, 1338)	2202 (1265, 3646)	5 (2, 11)	13 (4, 30)	223 (88, 459)	602 (110, 1568)	2 (0, 5)	4 (1, 14)	607 (334, 1026)	1600 (684, 2907)	3 (1, 8)	8 (2, 22)
**Nauru**	16 (9, 27)	26 (15, 44)	0 (0, 0)	0 (0, 0)	4 (1, 11)	7 (2, 18)	0 (0, 0)	0 (0, 0)	12 (5, 21)	19 (9, 35)	0 (0, 0)	0 (0, 0)
**Nepal**	7012 (4188, 11494)	21608 (13032, 34088)	41 (14, 100)	132 (44, 313)	2521 (601, 5738)	9237 (4466, 16077)	19 (3, 55)	69 (20, 173)	4490 (1737, 8335)	12372 (6676, 21170)	23 (5, 61)	63 (18, 160)
**Netherlands**	36051 (20518, 58903)	40612 (22304, 66766)	219 (76, 508)	245 (83, 576)	15245 (4440, 32650)	16005 (4366, 35804)	114 (26, 304)	119 (26, 328)	20807 (7799, 39508)	24607 (9433, 46539)	106 (27, 287)	126 (31, 338)
**New Zealand**	11015 (4100, 21889)	17955 (7264, 32855)	65 (17, 169)	105 (29, 249)	3655 (992, 9245)	5481 (1457, 13028)	27 (6, 81)	41 (9, 113)	7360 (1943, 16868)	12474 (3706, 25837)	38 (7, 117)	64 (13, 176)
**Nicaragua**	4870 (2711, 8189)	13924 (8032, 22720)	28 (9, 67)	79 (28, 187)	1361 (267, 3592)	3391 (705, 8719)	10 (2, 32)	25 (4, 77)	3508 (1428, 6582)	10533 (4600, 18963)	18 (5, 46)	54 (15, 136)
**Niger**	2688 (1616, 4267)	11112 (6690, 18501)	16 (5, 38)	63 (21, 157)	926 (437, 1681)	2775 (576, 6871)	7 (2, 18)	21 (2, 64)	1762 (960, 2956)	8337 (3754, 14733)	9 (3, 23)	43 (11, 114)
**Nigeria**	43305 (25722,	165579 (94722,	246 (87,	947 (330,	11031 (2415,	44905 (8281, 109763)	82 (15,	333 (49,	32274 (14481,	120674 (54914,	164 (48,	613 (179,

	70399)	268399)	565)	2170)	26351)		235)	968)	57303)	215627)	412)	1552)
**Niue**	3 (2, 6)	4 (2, 7)	0 (0, 0)	0 (0, 0)	1 (0, 2)	1 (0, 2)	0 (0, 0)	0 (0, 0)	3 (1, 5)	3 (1, 5)	0 (0, 0)	0 (0, 0)
**North Macedonia**	274 (147, 474)	390 (218, 665)	2 (0, 4)	2 (1, 5)	59 (10, 169)	75 (13, 212)	0 (0, 2)	1 (0, 2)	215 (96, 402)	315 (155, 564)	1 (0, 3)	2 (0, 4)
**Northern Mariana Islands**	114 (68, 184)	106 (60, 181)	1 (0, 2)	1 (0, 1)	32 (7, 80)	26 (6, 69)	0 (0, 1)	0 (0, 1)	82 (36, 150)	79 (34, 150)	0 (0, 1)	0 (0, 1)
**Norway**	10634 (6260, 17443)	13863 (8007, 22845)	64 (23, 149)	83 (30, 195)	4333 (1399, 9317)	5333 (1665, 11671)	32 (8, 88)	40 (10, 109)	6301 (2570, 11981)	8530 (3454, 16024)	32 (9, 84)	44 (12, 114)
**Oman**	2031 (1212, 3303)	9964 (5765, 15990)	12 (4, 28)	58 (20, 137)	685 (175, 1548)	2993 (763, 6953)	5 (1, 14)	22 (4, 64)	1347 (535, 2499)	6971 (2939, 12488)	7 (2, 18)	36 (10, 92)
**Pakistan**	60798 (35183, 99681)	180165 (99165, 290576)	368 (125, 875)	1110 (364, 2683)	25421 (3732, 61972)	81795 (10540, 199225)	189 (22, 554)	608 (60, 1785)	35377 (8530, 75749)	98370 (17694, 216012)	180 (29, 505)	502 (66, 1461)
**Palau**	31 (17, 50)	37 (20, 63)	0 (0, 0)	0 (0, 1)	8 (2, 21)	9 (2, 22)	0 (0, 0)	0 (0, 0)	22 (10, 41)	28 (13, 52)	0 (0, 0)	0 (0, 0)
**Palestine**	2767 (1642, 4441)	10299 (6055, 16632)	16 (6, 38)	61 (22, 141)	1003 (264, 2249)	3504 (919, 8006)	7 (1, 21)	26 (5, 73)	1765 (670, 3347)	6795 (2721, 12572)	9 (2, 23)	35 (9, 91)
**Panama**	3332 (1873, 5531)	8875 (5164, 14641)	19 (6, 45)	49 (17, 116)	709 (116, 1988)	1718 (296, 4855)	5 (1, 18)	13 (2, 42)	2624 (1234, 4703)	7157 (3507, 12665)	13 (4, 35)	36 (11, 92)
**Papua New**	3940	15520	22 (8,	87 (30,	1009 (197,	3450 (1439,	7 (1,	26 (7,	2931 (1252,	12070	15 (4,	61 (21,

**Guinea**	(2270, 6675)	(9273, 25154)	53)	204)	2629)	6737)	24)	67)	5430)	(6971, 19958)	39)	150)
**Paraguay**	1451 (848, 2424)	4610 (2582, 7604)	9 (3, 21)	27 (9, 65)	497 (177, 999)	1412 (283, 3476)	4 (1, 11)	10 (1, 31)	954 (483, 1739)	3198 (1315, 5955)	5 (1, 13)	16 (4, 44)
**Peru**	24973 (14525, 39976)	97638 (47083, 167982)	142 (47, 331)	537 (171, 1286)	6115 (2696, 11847)	17258 (3614, 43625)	46 (12, 119)	129 (20, 404)	18858 (10094, 31489)	80380 (30648, 149175)	96 (31, 234)	409 (109, 1062)
**Philippines**	91898 (53389, 154802)	291387 (170847, 484286)	537 (178, 1263)	1723 (585, 4039)	29560 (4643, 82209)	101695 (16221, 282848)	219 (30, 716)	754 (100, 2374)	62338 (20966, 118921)	189692 (59485, 366287)	318 (77, 829)	969 (222, 2603)
**Poland**	9531 (5390, 15912)	9009 (5455, 14024)	55 (19, 130)	50 (18, 118)	2665 (530, 6717)	1908 (359, 4935)	20 (3, 59)	14 (2, 44)	6867 (3043, 12663)	7101 (3594, 11949)	35 (10, 91)	36 (11, 91)
**Portugal**	21586 (12029, 34857)	26646 (14586, 43707)	132 (46, 308)	158 (54, 370)	9181 (2652, 19951)	9763 (2585, 22136)	68 (16, 185)	72 (16, 202)	12405 (4541, 23322)	16883 (6598, 31661)	63 (16, 172)	86 (23, 225)
**Puerto Rico**	6362 (3572, 10542)	6143 (3528, 10158)	37 (12, 89)	36 (12, 84)	2045 (430, 4931)	1852 (393, 4545)	15 (2, 45)	14 (2, 41)	4316 (1944, 7931)	4290 (1962, 7865)	22 (6, 58)	22 (6, 57)
**Qatar**	731 (424, 1195)	6015 (3462, 9769)	4 (2, 10)	35 (12, 81)	235 (61, 549)	1710 (430, 4139)	2 (0, 5)	13 (2, 36)	496 (206, 898)	4304 (1873, 7684)	3 (1, 7)	22 (6, 56)
**Republic of Korea**	72962 (29251, 134119)	102152 (41343, 189464)	424 (108, 1056)	572 (157, 1449)	21983 (4961, 56405)	22777 (5205, 60503)	164 (27, 488)	169 (28, 521)	50979 (16105, 104784)	79375 (26352, 158864)	260 (53, 717)	403 (95, 1089)

**Republic of Moldova**	753 (431, 1270)	855 (495, 1408)	4 (1, 11)	5 (1, 13)	248 (50, 595)	248 (49, 601)	2 (0, 6)	2 (0, 6)	505 (218, 942)	607 (279, 1113)	3 (0, 7)	3 (1, 9)
**Romania**	3107 (1686, 5451)	3037 (1673, 5231)	18 (4, 46)	17 (5, 43)	683 (112, 1907)	595 (93, 1705)	5 (0, 18)	4 (0, 16)	2424 (1097, 4498)	2442 (1177, 4411)	12 (2, 35)	12 (3, 33)
**Russian Federation**	31718 (18232, 52242)	36666 (21160, 60074)	183 (64, 430)	209 (73, 492)	9412 (1966, 22722)	9647 (1925, 24110)	70 (12, 200)	72 (12, 212)	22306 (10145, 40558)	27019 (12739, 48781)	113 (34, 291)	137 (41, 352)
**Rwanda**	3359 (1928, 5607)	9519 (5058, 15838)	20 (6, 47)	54 (17, 128)	1069 (393, 2143)	2350 (423, 6125)	8 (2, 22)	17 (2, 55)	2290 (1179, 4035)	7169 (2936, 12925)	12 (3, 29)	36 (9, 95)
**Saint Kitts and Nevis**	53 (30, 85)	106 (60, 178)	0 (0, 1)	1 (0, 1)	19 (4, 45)	33 (7, 79)	0 (0, 0)	0 (0, 1)	34 (14, 64)	74 (33, 135)	0 (0, 0)	0 (0, 1)
**Saint Lucia**	149 (84, 247)	256 (147, 413)	1 (0, 2)	1 (0, 4)	56 (12, 130)	80 (17, 193)	0 (0, 1)	1 (0, 2)	94 (38, 175)	176 (79, 321)	0 (0, 1)	1 (0, 2)
**Saint Vincent and the Grenadines**	106 (59, 173)	160 (90, 265)	1 (0, 2)	1 (0, 2)	40 (8, 91)	52 (11, 125)	0 (0, 1)	0 (0, 1)	66 (27, 126)	108 (48, 199)	0 (0, 1)	1 (0, 1)
**Samoa**	242 (134, 414)	426 (239, 712)	1 (0, 3)	2 (1, 6)	69 (14, 179)	115 (25, 295)	1 (0, 2)	1 (0, 3)	173 (72, 330)	311 (133, 575)	1 (0, 2)	2 (0, 4)
**San Marino**	65 (36, 105)	85 (46, 141)	0 (0, 1)	1 (0, 1)	28 (8, 60)	31 (8, 71)	0 (0, 1)	0 (0, 1)	37 (14, 70)	54 (20, 100)	0 (0, 0)	0 (0, 1)
**Sao Tome and Principe**	50 (25, 83)	162 (85, 262)	0 (0, 1)	1 (0, 2)	12 (2, 30)	35 (6, 87)	0 (0, 0)	0 (0, 1)	38 (15, 68)	127 (52, 224)	0 (0, 0)	1 (0, 2)
**Saudi Arabia**	24573 (14259,	106442 (61493,	145 (51,	614 (215,	8502 (2210, 19494)	31377 (8011, 73415)	63 (13,	232 (46,	16071 (6346, 29995)	75065 (32095,	82 (21, 210)	382 (108,

	39257)	172773)	338)	1430)			177)	666)		134143)		992)
**Senegal**	3812 (2323, 6120)	10835 (6655, 17198)	22 (7, 54)	62 (22, 148)	1264 (594, 2287)	3098 (1337, 5843)	9 (2, 24)	23 (6, 60)	2548 (1399, 4304)	7737 (4260, 12759)	13 (4, 33)	39 (12, 99)
**Serbia**	1290 (702, 2263)	1492 (835, 2495)	7 (2, 19)	8 (2, 21)	265 (42, 744)	298 (49, 833)	2 (0, 7)	2 (0, 8)	1025 (470, 1897)	1194 (571, 2130)	5 (1, 14)	6 (1, 16)
**Seychelles**	164 (96, 272)	300 (179, 496)	1 (0, 2)	2 (1, 4)	52 (12, 128)	78 (19, 195)	0 (0, 1)	1 (0, 2)	113 (51, 201)	223 (106, 391)	1 (0, 1)	1 (0, 3)
**Sierra Leone**	1725 (1029, 2803)	6653 (3955, 10869)	10 (3, 23)	37 (12, 88)	331 (59, 877)	1237 (216, 3301)	2 (0, 8)	9 (1, 30)	1394 (691, 2410)	5415 (2679, 9314)	7 (2, 18)	28 (8, 71)
**Singapore**	6282 (2480, 11752)	16220 (6732, 29437)	36 (9, 90)	90 (26, 222)	1786 (408, 4645)	3048 (680, 8115)	13 (2, 40)	23 (3, 73)	4495 (1387, 9195)	13171 (4760, 25350)	23 (5, 62)	67 (16, 179)
**Slovakia**	766 (412, 1300)	979 (553, 1631)	4 (1, 11)	5 (2, 14)	155 (24, 447)	184 (30, 533)	1 (0, 4)	1 (0, 5)	611 (283, 1097)	794 (389, 1402)	3 (1, 9)	4 (1, 11)
**Slovenia**	286 (164, 494)	332 (184, 570)	2 (0, 4)	2 (0, 4)	47 (14, 112)	38 (6, 125)	0 (0, 1)	0 (0, 1)	240 (126, 425)	294 (146, 516)	1 (0, 3)	1 (0, 4)
**Solomon Islands**	326 (185, 541)	1157 (658, 1929)	2 (1, 5)	7 (2, 16)	99 (19, 251)	323 (66, 813)	1 (0, 2)	2 (0, 7)	228 (94, 427)	833 (365, 1547)	1 (0, 3)	4 (1, 11)
**Somalia**	3239 (1972, 5317)	10843 (6599, 18013)	18 (6, 44)	62 (21, 146)	853 (182, 2033)	2974 (647, 7179)	6 (1, 19)	22 (3, 67)	2386 (1123, 4246)	7870 (3616, 14249)	12 (3, 31)	40 (10, 103)
**South Africa**	34774 (19805,	67771 (39389,	203 (70,	390 (133,	11321 (2336,	19449 (3959, 49807)	84 (14,	144 (24,	23453 (10032,	48322 (20963,	119 (34,	246 (70,

	56244)	112377)	468)	918)	26725)		238)	432)	42955)	87030)	308)	645)
**South Sudan**	2764 (1671, 4466)	5407 (3271, 8806)	16 (5, 38)	31 (10, 76)	799 (176, 1909)	1470 (317, 3572)	6 (1, 17)	11 (1, 34)	1965 (886, 3534)	3938 (1813, 7026)	10 (3, 26)	20 (5, 55)
**Spain**	71284 (39832, 119798)	103168 (52477, 172672)	439 (147, 1047)	603 (194, 1459)	31708 (12793, 63960)	32195 (7699, 76500)	239 (66, 617)	240 (45, 685)	39577 (18741, 73119)	70973 (28662, 132031)	201 (58, 507)	363 (96, 967)
**Sri Lanka**	33194 (19540, 53897)	62870 (36958, 103685)	199 (69, 466)	360 (126, 843)	12978 (5708, 24103)	17036 (4200, 42081)	97 (30, 235)	126 (23, 373)	20216 (10999, 34106)	45834 (21540, 80700)	103 (32, 251)	234 (70, 583)
**Sudan**	19949 (12149, 31934)	85851 (51001, 135367)	115 (41, 272)	482 (169, 1107)	6095 (2806, 11134)	19637 (4025, 50408)	45 (13, 111)	145 (22, 435)	13855 (7577, 23124)	66213 (31019, 113933)	70 (22, 176)	337 (102, 832)
**Suriname**	440 (248, 719)	867 (486, 1439)	3 (1, 6)	5 (2, 12)	158 (34, 367)	279 (60, 668)	1 (0, 3)	2 (0, 6)	282 (119, 530)	588 (261, 1073)	1 (0, 4)	3 (1, 8)
**Sweden**	16629 (9587, 27443)	21190 (12255, 34834)	100 (34, 236)	126 (44, 296)	6428 (2008, 14272)	7681 (2375, 17181)	48 (12, 132)	57 (14, 156)	10201 (4083, 19123)	13509 (5409, 25381)	52 (14, 137)	69 (19, 178)
**Switzerland**	17540 (9629, 28462)	21966 (12248, 35818)	107 (38, 252)	131 (45, 313)	7349 (2074, 16086)	8234 (2154, 18423)	55 (12, 146)	61 (13, 169)	10191 (3806, 19457)	13732 (5357, 25727)	52 (13, 140)	70 (18, 189)
**Syrian Arab Republic**	16738 (9747, 26473)	30701 (17489, 50641)	99 (35, 230)	177 (61, 410)	5689 (2072, 11363)	8919 (2172, 21156)	42 (11, 106)	66 (13, 183)	11049 (5250, 20008)	21782 (8950, 40413)	56 (17, 143)	111 (31, 287)
**Taiwan (Province of**	32692 (18818,	51800 (30684,	179 (59,	280 (98,	5325 (873, 15281)	6729 (1248, 18834)	40 (4, 131)	50 (6, 166)	27367 (14075,	45071 (24016,	140 (43,	230 (75,

**China)**	53710)	84973)	436)	675)					47467)	76256)	357)	565)
**Tajikistan**	1301 (727, 2175)	3416 (2047, 5506)	8 (2, 19)	20 (7, 51)	415 (81, 1007)	1131 (447, 2222)	3 (0, 9)	8 (1, 24)	887 (362, 1657)	2285 (1202, 3945)	5 (1, 12)	12 (3, 30)
**Thailand**	108636 (64737, 176954)	199298 (118024, 330761)	674 (239, 1538)	1165 (412, 2737)	51384 (24459, 93480)	63117 (17208, 148411)	383 (121, 921)	471 (99, 1332)	57252 (30240, 99888)	136182 (60245, 243473)	291 (91, 713)	694 (205, 1784)
**Timor-Leste**	888 (526, 1489)	2523 (1460, 4325)	5 (2, 12)	15 (5, 35)	314 (77, 725)	904 (222, 2135)	2 (0, 6)	7 (1, 19)	574 (256, 1051)	1619 (703, 2969)	3 (1, 8)	8 (2, 21)
**Togo**	1459 (874, 2380)	5769 (3446, 9406)	8 (3, 20)	33 (11, 77)	427 (153, 881)	1418 (297, 3464)	3 (1, 9)	11 (1, 32)	1031 (527, 1792)	4351 (1971, 7761)	5 (1, 13)	22 (6, 56)
**Tokelau**	2 (1, 3)	3 (2, 5)	0 (0, 0)	0 (0, 0)	1 (0, 1)	1 (0, 2)	0 (0, 0)	0 (0, 0)	2 (1, 3)	2 (1, 4)	0 (0, 0)	0 (0, 0)
**Tonga**	163 (90, 280)	251 (143, 419)	1 (0, 2)	1 (0, 3)	46 (9, 118)	66 (14, 172)	0 (0, 1)	0 (0, 1)	118 (50, 221)	185 (82, 342)	1 (0, 2)	1 (0, 2)
**Trinidad and Tobago**	1396 (818, 2258)	2210 (1284, 3587)	9 (3, 20)	13 (4, 31)	590 (259, 1108)	680 (153, 1644)	4 (1, 11)	5 (1, 15)	806 (415, 1432)	1530 (686, 2776)	4 (1, 10)	8 (2, 20)
**Tunisia**	12412 (7543, 20247)	25536 (15025, 41400)	77 (28, 182)	150 (52, 348)	6025 (2977, 10858)	8651 (2416, 19367)	45 (15, 111)	64 (13, 177)	6387 (3124, 11613)	16885 (6768, 31173)	32 (10, 81)	86 (23, 226)
**Turkey**	80668 (49077, 126652)	165967 (97337, 267207)	510 (186, 1171)	965 (344, 2245)	41753 (21130, 71998)	51045 (12562, 120314)	313 (105, 731)	381 (73, 1083)	38915 (18976, 68364)	114922 (48078, 208631)	197 (56, 483)	584 (155, 1516)

**Turkmenistan**	1214 (681, 2007)	2426 (1382, 4002)	7 (2, 18)	14 (4, 34)	347 (61, 870)	613 (108, 1578)	3 (0, 8)	4 (0, 14)	866 (378, 1583)	1813 (827, 3303)	4 (1, 13)	9 (2, 24)
**Tuvalu**	14 (8, 23)	25 (15, 41)	0 (0, 0)	0 (0, 0)	4 (1, 9)	7 (1, 17)	0 (0, 0)	0 (0, 0)	10 (4, 19)	18 (8, 33)	0 (0, 0)	0 (0, 0)
**Uganda**	8393 (5075, 13361)	27285 (16505, 42949)	49 (16, 116)	152 (52, 365)	2565 (1237, 4620)	5881 (2227, 11700)	19 (5, 48)	43 (9, 119)	5828 (3262, 9651)	21404 (12142, 35184)	29 (9, 73)	109 (34, 262)
**Ukraine**	9897 (5702, 16305)	9431 (5492, 15403)	59 (17, 144)	55 (17, 135)	3696 (890, 8412)	3025 (687, 7220)	28 (3, 83)	23 (2, 71)	6202 (2543, 11799)	6406 (2811, 11839)	31 (6, 89)	32 (7, 86)
**United Arab Emirates**	2649 (1566, 4244)	16676 (9667, 27172)	16 (6, 36)	94 (33, 218)	880 (236, 2004)	4100 (1018, 10031)	7 (1, 18)	31 (5, 89)	1768 (726, 3210)	12576 (5829, 22319)	9 (2, 23)	64 (19, 161)
**United Kingdom**	155932 (91189, 256501)	202395 (115063, 331135)	950 (344, 2216)	1218 (428, 2844)	65441 (21285, 138562)	78263 (23879, 171377)	488 (125, 1309)	585 (139, 1588)	90491 (36038, 171304)	124132 (49043, 231275)	462 (127, 1219)	633 (175, 1639)
**United Republic of Tanzania**	15438 (9217, 24985)	45458 (27851, 72664)	87 (30, 205)	250 (83, 594)	3840 (1713, 7195)	8274 (3172, 16966)	29 (7, 72)	62 (11, 169)	11599 (6474, 19623)	37185 (21435, 61031)	59 (18, 142)	189 (61, 445)
**United States of America**	181 (101, 305)	133 (77, 222)	1 (0, 3)	1 (0, 2)	59 (13, 145)	41 (9, 100)	0 (0, 1)	0 (0, 1)	122 (54, 227)	93 (42, 172)	2019 (467, 5551)	3149 (818, 8137)
**United States Virgin Islands**	694204 (373575, 1149605)	1001935 (588468, 1560485)	4243 (1442, 9466)	6006 (2151, 13334)	298626 (83435, 657917)	383664 (108611, 842226)	2225 (507, 5821)	2856 (659, 7596)	395578 (120677, 806073)	618271 (226826, 1162883)	1 (0, 2)	0 (0, 1)

**Uruguay**	3193 (1846, 5385)	5764 (3312, 9721)	19 (6, 44)	34 (12, 77)	1134 (306, 2628)	1918 (532, 4442)	8 (2, 24)	14 (3, 39)	2060 (800, 3948)	3846 (1552, 7222)	10 (3, 28)	20 (5, 51)
**Uzbekistan**	6813 (3736, 11187)	16913 (9634, 27295)	39 (12, 95)	95 (30, 227)	1946 (367, 4862)	3974 (761, 10269)	14 (1, 47)	30 (3, 92)	4867 (2078, 8955)	12939 (6064, 22759)	25 (6, 69)	66 (17, 167)
**Vanuatu**	181 (101, 300)	582 (332, 976)	1 (0, 2)	3 (1, 8)	52 (11, 131)	163 (35, 414)	0 (0, 1)	1 (0, 4)	128 (55, 239)	419 (181, 777)	1 (0, 2)	2 (1, 6)
**Venezuela (Bolivarian Republic of)**	35000 (19612, 57556)	59496 (35009, 99025)	201 (69, 487)	335 (117, 781)	9438 (1793, 24697)	13849 (2690, 37065)	70 (11, 220)	103 (14, 313)	25562 (11444, 46744)	45648 (21569, 82304)	130 (37, 337)	232 (70, 582)
**Viet Nam**	70757 (41431, 116154)	204342 (119281, 336716)	409 (134, 947)	1157 (387, 2710)	21918 (4741, 54678)	48643 (11466, 119659)	162 (24, 497)	363 (63, 1091)	48839 (22444, 86527)	155699 (74606, 270369)	247 (70, 626)	794 (240, 2010)
**Yemen**	11986 (7323, 19514)	45319 (27307, 73428)	71 (26, 166)	267 (95, 617)	4552 (1220, 9858)	15570 (3936, 35735)	33 (6, 94)	115 (20, 321)	7435 (2807, 13960)	29748 (11798, 54237)	38 (10, 101)	152 (38, 397)
**Zambia**	5392 (3211, 8734)	17474 (9963, 28030)	30 (10, 73)	98 (33, 231)	1170 (483, 2311)	4054 (775, 10434)	9 (2, 24)	30 (4, 91)	4222 (2385, 7083)	13420 (6199, 23641)	21 (7, 52)	68 (19, 177)
**Zimbabwe**	6925 (4091, 11137)	12844 (7298, 21381)	39 (13, 93)	72 (23, 168)	1645 (557, 3420)	2790 (476, 7594)	12 (2, 33)	21 (2, 68)	5280 (2978, 8799)	10054 (4711, 17846)	27 (8, 66)	51 (14, 132)

YLDs, years lived with disability; UI, uncertainty interval.

Table S4. Prevalence and years lived with disability of infertility (primary infertility and secondary infertility) due to PCOS and their average annual percentage changes from 1990 to 2021 in different countries.

Location	Infertility						Primary infertility						Secondary infertility					
	ASPR, per 100,000 (95% UI)		AAPC (95% CI)	ASYR, per 100,000 (95% UI)		AAPC (95% CI)	ASPR, per 100,000 (95% UI)		AAPC (95% CI)	ASYR, per 100,000 (95% UI)		AAPC (95% CI)	ASPR, per 100,000 (95% UI)		AAPC (95% CI)	ASYR, per 100,000 (95% UI)		AAPC (95% CI)
	1990	2021	1990-2021	1990	2021	1990-2021	1990	2021	1990-2021	1990	2021	1990-2021	1990	2021	1990-2021	1990	2021	1990-2021
**Afghanistan**	364.93 (218.14, 598.80)	503.87 (309.55, 786.56)	1.09 (1.06 to 1.12)	2.01 (0.69, 4.74)	2.82 (1.03, 6.56)	1.13 (1.1 to 1.15)	69.40 (13.88, 181.50)	109.92 (50.84, 204.95)	1.52 (1.48 to 1.55)	0.51 (0.05, 1.62)	0.82 (0.23, 2.05)	1.51 (1.47 to 1.55)	295.53 (143.89, 508.87)	393.95 (232.51, 628.42)	0.99 (0.95 to 1.02)	1.50 (0.44, 3.73)	2.00 (0.71, 4.81)	0.98 (0.95 to 1.01)
**Albania**	54.86 (29.32, 95.63)	67.52 (36.85, 114.93)	0.69 (0.67 to 0.71)	0.32 (0.08, 0.82)	0.40 (0.11, 0.99)	0.74 (0.7 to 0.77)	15.82 (2.96, 41.32)	22.26 (8.22, 47.23)	1.17 (1.11 to 1.22)	0.12 (0.01, 0.40)	0.17 (0.03, 0.49)	1.2 (1.14 to 1.27)	39.05 (16.24, 75.00)	45.26 (23.43, 81.08)	0.47 (0.45 to 0.5)	0.20 (0.04, 0.57)	0.23 (0.05, 0.60)	0.41 (0.35 to 0.46)
**Algeria**	597.79 (356.54, 961.11)	877.25 (490.06, 1432.63)	1.26 (1.25 to 1.29)	3.55 (1.25, 8.23)	5.11 (1.78, 12.32)	1.2 (1.18 to 1.22)	217.63 (78.60, 437.71)	271.41 (69.35, 629.85)	0.75 (0.7 to 0.79)	1.62 (0.41, 4.14)	2.02 (0.40, 5.72)	0.76 (0.72 to 0.81)	380.16 (175.27, 687.99)	605.84 (246.93, 1106.86)	1.54 (1.51 to 1.57)	1.93 (0.55, 4.97)	3.09 (0.83, 8.03)	1.55 (1.51 to 1.57)

**American Samoa**	744.37 (421.67, 1237.39)	1007.58 (582.90, 1672.99)	0.96 (0.94 to 0.98)	4.25 (1.43, 10.14)	5.75 (1.97, 13.86)	0.96 (0.94 to 0.98)	197.21 (41.72, 508.74)	266.58 (58.32, 675.32)	0.96 (0.94 to 0.98)	1.46 (0.24, 4.41)	1.97 (0.34, 5.89)	0.96 (0.94 to 0.98)	547.16 (240.72, 1004.08)	740.99 (330.41, 1345.08)	0.97 (0.95 to 0.98)	2.78 (0.79, 7.32)	3.77 (1.09, 9.73)	0.97 (0.95 to 0.98)
**Andorra**	1002.08 (550.83, 1636.38)	1208.33 (657.77, 1989.06)	0.61 (0.6 to 0.62)	6.12 (2.10, 14.40)	7.36 (2.48, 16.95)	0.6 (0.59 to 0.61)	426.86 (121.68, 947.63)	504.95 (135.73, 1122.60)	0.54 (0.53 to 0.54)	3.18 (0.69, 8.43)	3.77 (0.79, 10.25)	0.54 (0.53 to 0.55)	575.22 (217.31, 1102.89)	703.38 (263.15, 1342.92)	0.66 (0.65 to 0.67)	2.94 (0.74, 7.88)	3.59 (0.92, 9.69)	0.66 (0.64 to 0.68)
**Angola**	165.48 (97.91, 278.85)	299.94 (174.43, 490.97)	1.97 (1.93 to 2)	0.93 (0.31, 2.28)	1.69 (0.56, 4.02)	1.96 (1.92 to 2)	38.67 (7.69, 99.27)	69.78 (14.05, 174.34)	1.94 (1.9 to 1.98)	0.28 (0.03, 0.90)	0.52 (0.06, 1.53)	1.97 (1.92 to 2.02)	126.81 (59.55, 225.63)	230.16 (107.00, 408.90)	1.97 (1.94 to 2)	0.65 (0.17, 1.69)	1.17 (0.31, 3.00)	1.96 (1.91 to 2.01)
**Antigua and Barbuda**	453.99 (257.64, 728.99)	597.27 (340.04, 986.61)	0.88 (0.86 to 0.9)	2.66 (0.87, 6.19)	3.49 (1.18, 8.14)	0.88 (0.86 to 0.9)	149.50 (30.62, 355.57)	193.74 (40.79, 464.20)	0.82 (0.8 to 0.84)	1.11 (0.17, 3.22)	1.44 (0.22, 4.14)	0.85 (0.83 to 0.87)	304.49 (133.67, 558.47)	403.53 (176.16, 748.47)	0.93 (0.9 to 0.95)	1.55 (0.43, 4.08)	2.05 (0.58, 5.31)	0.92 (0.9 to 0.95)
**Argentina**	417.48 (241.73, 708.68)	628.68 (364.88, 1053.23)	1.35 (1.32 to 1.37)	2.46 (0.84, 5.75)	3.72 (1.31, 8.66)	1.38 (1.35 to 1.41)	144.73 (39.00, 344.37)	223.52 (62.53, 519.26)	1.42 (1.4 to 1.43)	1.07 (0.22, 3.04)	1.66 (0.36, 4.63)	1.44 (1.41 to 1.45)	272.75 (108.12, 518.84)	405.16 (159.69, 768.42)	1.33 (1.3 to 1.36)	1.39 (0.34, 3.63)	2.06 (0.53, 5.47)	1.33 (1.29 to 1.37)
**Armenia**	122.86	164.24	0.94	0.69	0.91	0.93	26.62	32.72	0.63	0.20	0.25	0.66	96.24	131.52	1.03	0.49	0.67	1.02

	(69.08, 202.27)	(93.18, 274.41)	(0.91 to 0.97)	(0.21, 1.67)	(0.28, 2.28)	(0.89 to 0.97)	(4.06, 73.40)	(9.32, 79.69)	(0.61 to 0.66)	(0.01, 0.65)	(0.03, 0.75)	(0.61 to 0.7)	(44.64, 170.48)	(71.51, 227.62)	(1 to 1.05)	(0.13, 1.28)	(0.19, 1.69)	(0.97 to 1.07)
**Australia**	897.49 (327.91, 1726.33)	1016.77 (373.76, 2000.50)	0.4 (0.38 to 0.42)	5.31 (1.35, 13.44)	5.99 (1.51, 15.34)	0.39 (0.38 to 0.41)	304.65 (82.40, 750.39)	336.94 (91.01, 820.30)	0.33 (0.32 to 0.34)	2.27 (0.45, 6.45)	2.51 (0.48, 7.39)	0.33 (0.32 to 0.34)	592.84 (156.66, 1319.68)	679.83 (179.49, 1546.36)	0.45 (0.44 to 0.46)	3.04 (0.58, 8.97)	3.48 (0.67, 10.41)	0.45 (0.43 to 0.46)
**Austria**	1368.42 (808.52, 2189.80)	1447.20 (833.58, 2335.49)	0.15 (0.13 to 0.17)	8.10 (2.95, 18.58)	8.53 (3.01, 20.16)	0.13 (0.11 to 0.16)	475.13 (132.28, 1078.80)	484.10 (132.08, 1130.10)	0.04 (0 to 0.1)	3.53 (0.79, 9.83)	3.62 (0.79, 9.84)	0.03 (-0.02 to 0.1)	893.30 (376.26, 1605.07)	963.10 (401.84, 1743.83)	0.24 (0.21 to 0.25)	4.57 (1.29, 11.86)	4.91 (1.37, 12.81)	0.23 (0.22 to 0.24)
**Azerbaijan**	127.32 (75.37, 208.67)	196.53 (114.59, 316.50)	1.41 (1.39 to 1.43)	0.73 (0.23, 1.73)	1.13 (0.36, 2.76)	1.44 (1.4 to 1.48)	38.57 (7.21, 93.63)	58.48 (10.73, 143.79)	1.38 (1.34 to 1.42)	0.28 (0.02, 0.85)	0.43 (0.04, 1.34)	1.38 (1.32 to 1.43)	88.75 (38.90, 163.36)	138.05 (60.69, 250.79)	1.44 (1.42 to 1.46)	0.45 (0.10, 1.25)	0.70 (0.17, 1.86)	1.46 (1.43 to 1.5)
**Bahamas**	567.92 (324.22, 934.16)	672.33 (382.29, 1128.61)	0.56 (0.55 to 0.57)	3.33 (1.12, 7.84)	3.92 (1.33, 9.36)	0.55 (0.53 to 0.56)	188.32 (41.11, 447.87)	219.52 (47.01, 524.35)	0.5 (0.49 to 0.51)	1.40 (0.21, 4.09)	1.63 (0.28, 4.77)	0.49 (0.47 to 0.51)	379.59 (168.79, 705.00)	452.82 (199.76, 840.79)	0.59 (0.58 to 0.6)	1.94 (0.54, 5.11)	2.30 (0.63, 5.97)	0.57 (0.56 to 0.59)
**Bahrain**	839.18 (480.96,	954.90 (552.23,	0.44	4.91 (1.66,	5.57 (1.90,	0.44	274.99 (71.16,	305.53 (79.11,	0.38	2.04 (0.39,	2.26 (0.47,	0.34	564.19 (227.29,	649.36 (267.91,	0.49	2.87 (0.76,	3.31 (0.89,	0.49

	1347.90)	1539.92)	(0.43 to 0.47)	11.33)	12.75)	(0.42 to 0.47)	623.70)	709.45)	(0.36 to 0.41)	5.60)	6.34)	(0.31 to 0.37)	1051.69)	1202.83)	(0.47 to 0.51)	7.41)	8.55)	(0.47 to 0.51)
**Bangladesh**	145.33 (88.39, 235.25)	232.39 (141.10, 369.44)	1.5 (1.48 to 1.53)	0.86 (0.28, 2.05)	1.36 (0.46, 3.15)	1.48 (1.43 to 1.52)	53.82 (27.35, 92.35)	76.27 (39.27, 131.06)	1.14 (1.06 to 1.23)	0.40 (0.10, 1.01)	0.57 (0.16, 1.42)	1.13 (1.04 to 1.22)	91.51 (52.08, 152.57)	156.11 (91.29, 254.07)	1.74 (1.7 to 1.77)	0.46 (0.13, 1.15)	0.79 (0.24, 1.95)	1.76 (1.72 to 1.79)
**Barbados**	561.78 (319.58, 926.54)	657.29 (377.46, 1068.95)	0.52 (0.51 to 0.54)	3.28 (1.09, 7.73)	3.85 (1.32, 9.29)	0.53 (0.51 to 0.55)	183.08 (39.97, 442.16)	211.83 (45.53, 512.45)	0.47 (0.46 to 0.48)	1.35 (0.23, 3.91)	1.58 (0.26, 4.50)	0.5 (0.49 to 0.52)	378.70 (166.52, 693.12)	445.46 (196.05, 818.02)	0.54 (0.53 to 0.56)	1.92 (0.54, 5.19)	2.26 (0.64, 5.98)	0.55 (0.53 to 0.57)
**Belarus**	78.10 (43.33, 131.79)	104.30 (59.41, 175.30)	0.96 (0.94 to 0.99)	0.46 (0.12, 1.14)	0.61 (0.18, 1.52)	0.97 (0.95 to 1.01)	25.97 (4.35, 63.53)	33.95 (5.66, 80.97)	0.89 (0.88 to 0.92)	0.19 (0.01, 0.61)	0.25 (0.02, 0.76)	0.95 (0.89 to 0.99)	52.14 (22.02, 99.55)	70.35 (30.59, 131.69)	1 (0.98 to 1.02)	0.26 (0.06, 0.74)	0.36 (0.08, 1.00)	1.02 (0.99 to 1.07)
**Belgium**	1074.79 (640.22, 1716.41)	1305.92 (779.03, 2119.15)	0.67 (0.63 to 0.7)	6.58 (2.41, 15.26)	7.83 (2.83, 18.40)	0.59 (0.56 to 0.62)	461.41 (234.52, 838.00)	493.58 (145.23, 1107.04)	0.22 (0.2 to 0.23)	3.46 (1.15, 8.35)	3.68 (0.86, 10.05)	0.2 (0.18 to 0.21)	613.38 (312.73, 1054.97)	812.33 (337.31, 1487.47)	0.95 (0.9 to 1)	3.12 (0.99, 7.83)	4.15 (1.14, 10.89)	0.97 (0.92 to 1.01)
**Belize**	434.64 (255.75, 702.19)	615.74 (354.39, 1003.45)	1.15 (1.12	2.64 (0.92, 6.18)	3.67 (1.24, 8.63)	1.07 (1.03	180.63 (74.42, 343.77)	229.42 (54.76, 536.28)	0.79 (0.76	1.35 (0.35, 3.37)	1.70 (0.30, 4.84)	0.75 (0.72	254.01 (129.70, 441.42)	386.32 (161.89, 715.59)	1.38 (1.35	1.29 (0.40, 3.21)	1.97 (0.53, 5.15)	1.37 (1.33

			to 1.18)			to 1.12)			to 0.83)			to 0.79)			to 1.42)			to 1.4)
**Benin**	178.12 (102.55, 292.14)	321.32 (193.88, 508.40)	1.92 (1.89 to 1.95)	0.98 (0.31, 2.40)	1.77 (0.59, 4.20)	1.93 (1.88 to 1.98)	33.10 (6.03, 83.61)	60.31 (24.70, 120.36)	1.96 (1.89 to 2.04)	0.24 (0.02, 0.75)	0.45 (0.10, 1.13)	1.9 (1.82 to 1.98)	145.02 (66.02, 251.19)	261.01 (150.99, 424.24)	1.92 (1.89 to 1.95)	0.74 (0.20, 1.90)	1.33 (0.42, 3.26)	1.93 (1.88 to 1.97)
**Bermuda**	673.61 (377.32, 1125.99)	746.43 (417.27, 1261.15)	0.34 (0.32 to 0.37)	3.95 (1.32, 9.29)	4.36 (1.45, 10.23)	0.34 (0.31 to 0.36)	216.86 (44.95, 527.68)	234.88 (49.68, 576.81)	0.28 (0.25 to 0.32)	1.62 (0.26, 4.76)	1.74 (0.28, 5.18)	0.26 (0.23 to 0.29)	456.76 (203.19, 842.64)	511.55 (226.59, 955.50)	0.38 (0.36 to 0.4)	2.33 (0.64, 5.98)	2.61 (0.74, 6.66)	0.39 (0.37 to 0.41)
**Bhutan**	209.37 (124.23, 339.45)	368.32 (214.22, 591.39)	1.85 (1.83 to 1.87)	1.22 (0.41, 2.86)	2.15 (0.75, 4.95)	1.86 (1.85 to 1.88)	70.26 (17.05, 162.93)	121.32 (28.91, 284.56)	1.79 (1.77 to 1.81)	0.51 (0.07, 1.51)	0.90 (0.16, 2.61)	1.83 (1.8 to 1.86)	139.12 (55.18, 256.15)	247.00 (98.95, 452.69)	1.88 (1.87 to 1.9)	0.70 (0.18, 1.88)	1.25 (0.32, 3.33)	1.87 (1.85 to 1.89)
**Bolivia (Plurinational State of)**	623.90 (367.51, 985.54)	895.45 (401.87, 1534.48)	1.19 (1.14 to 1.24)	3.53 (1.25, 8.33)	4.99 (1.50, 12.04)	1.18 (1.13 to 1.24)	147.24 (63.02, 278.56)	186.05 (43.68, 459.19)	0.76 (0.71 to 0.8)	1.10 (0.29, 2.86)	1.38 (0.24, 4.14)	0.74 (0.7 to 0.78)	476.66 (264.83, 776.93)	709.40 (245.50, 1349.29)	1.32 (1.27 to 1.37)	2.42 (0.81, 5.81)	3.61 (0.88, 9.72)	1.33 (1.28 to 1.38)
**Bosnia and Herzegovina**	46.83 (24.41, 82.40)	72.54 (39.31, 124.77)	1.46 (1.43 to	0.26 (0.06, 0.68)	0.41 (0.10, 1.04)	1.5 (1.47 to	10.37 (1.63, 29.05)	15.87 (2.48, 44.38)	1.4 (1.38 to	0.08 (0.00, 0.28)	0.12 (0.01, 0.42)	1.51 (1.46 to	36.46 (16.12, 68.61)	56.68 (26.15, 103.04)	1.47 (1.44 to	0.18 (0.04, 0.51)	0.29 (0.06, 0.78)	1.5 (1.46 to

			1.49)			1.54)			1.43)			1.56)			1.51)			1.54)
**Botswana**	251.55 (148.72, 400.91)	404.75 (226.02, 658.90)	1.55 (1.51 to 1.58)	1.38 (0.44, 3.28)	2.23 (0.74, 5.31)	1.57 (1.53 to 1.61)	41.64 (13.39, 91.43)	74.50 (12.15, 213.41)	1.88 (1.84 to 1.93)	0.31 (0.05, 0.92)	0.55 (0.05, 1.86)	1.83 (1.77 to 1.89)	209.91 (123.91, 340.60)	330.25 (161.65, 571.15)	1.47 (1.44 to 1.5)	1.06 (0.33, 2.59)	1.68 (0.49, 4.26)	1.48 (1.44 to 1.52)
**Brazil**	209.59 (117.70, 344.91)	220.52 (125.21, 357.60)	0.12 (0.07 to 0.16)	1.26 (0.43, 2.89)	1.30 (0.46, 3.02)	0.07 (0.03 to 0.11)	81.90 (19.72, 186.30)	74.53 (17.75, 177.86)	-0.3 (-0.33 to - 0.28)	0.61 (0.12, 1.66)	0.55 (0.11, 1.58)	-0.29 (-0.32 to - 0.26)	127.68 (49.27, 250.30)	145.98 (59.49, 275.38)	0.48 (0.43 to 0.55)	0.65 (0.17, 1.75)	0.74 (0.20, 1.98)	0.49 (0.44 to 0.55)
**Brunei Darussalam**	673.97 (270.01, 1275.22)	951.31 (369.16, 1807.08)	1.14 (1.13 to 1.17)	3.86 (1.02, 9.72)	5.41 (1.44, 13.58)	1.11 (1.09 to 1.12)	181.39 (40.34, 484.18)	235.77 (54.36, 636.61)	0.85 (0.84 to 0.85)	1.35 (0.22, 4.14)	1.76 (0.29, 5.50)	0.85 (0.83 to 0.86)	492.59 (157.29, 1008.23)	715.54 (223.40, 1487.35)	1.25 (1.23 to 1.28)	2.51 (0.55, 6.90)	3.65 (0.84, 10.14)	1.26 (1.23 to 1.29)
**Bulgaria**	64.79 (34.97, 111.86)	82.68 (45.29, 140.17)	0.79 (0.78 to 0.8)	0.36 (0.09, 0.92)	0.46 (0.13, 1.15)	0.81 (0.78 to 0.84)	14.14 (2.28, 39.51)	17.87 (2.80, 50.84)	0.76 (0.74 to 0.77)	0.10 (0.01, 0.37)	0.13 (0.01, 0.47)	0.77 (0.73 to 0.81)	50.65 (23.18, 93.96)	64.81 (30.14, 118.76)	0.8 (0.79 to 0.81)	0.26 (0.05, 0.70)	0.33 (0.08, 0.89)	0.81 (0.77 to 0.85)
**Burkina Faso**	164.92 (99.40, 268.39)	269.22 (154.38, 441.79)	1.6 (1.56 to 1.63)	0.96 (0.32, 2.31)	1.50 (0.48, 3.57)	1.46 (1.43 to 1.51)	53.17 (24.66, 98.44)	56.86 (11.51, 138.70)	0.24 (0.17 to 0.31)	0.40 (0.09, 1.01)	0.42 (0.04, 1.29)	0.23 (0.15 to 0.31)	111.75 (60.17, 190.16)	212.36 (95.41, 374.52)	2.11 (2.07 to 2.14)	0.57 (0.16, 1.41)	1.08 (0.30, 2.79)	2.14 (2.1 to 2.19)

**Burundi**	168.84 (100.27, 271.73)	169.69 (101.59, 275.34)	0.02 (0.01 to 0.03)	0.97 (0.32, 2.36)	0.95 (0.31, 2.27)	-0.04 (-0.07 to -0.02)	45.44 (17.03, 90.46)	36.08 (14.58, 71.88)	-0.73 (-0.8 to -0.68)	0.34 (0.07, 0.94)	0.27 (0.05, 0.74)	-0.67 (-0.75 to -0.61)	123.40 (67.95, 207.73)	133.61 (76.99, 220.33)	0.28 (0.24 to 0.31)	0.63 (0.19, 1.55)	0.67 (0.21, 1.68)	0.28 (0.24 to 0.31)
**Cabo Verde**	199.08 (118.71, 328.23)	333.55 (199.69, 541.65)	1.68 (1.66 to 1.71)	1.11 (0.36, 2.65)	1.84 (0.63, 4.37)	1.66 (1.63 to 1.69)	41.24 (8.17, 104.57)	65.09 (13.26, 166.88)	1.5 (1.47 to 1.55)	0.31 (0.03, 0.94)	0.48 (0.06, 1.50)	1.48 (1.43 to 1.53)	157.84 (76.24, 277.74)	268.46 (131.14, 461.99)	1.73 (1.7 to 1.75)	0.80 (0.22, 2.07)	1.36 (0.39, 3.45)	1.72 (1.69 to 1.76)
**Cambodia**	441.04 (256.50, 745.42)	741.65 (430.44, 1230.32)	1.73 (1.68 to 1.79)	2.48 (0.81, 5.86)	4.15 (1.39, 9.91)	1.72 (1.66 to 1.78)	102.38 (19.31, 271.09)	160.36 (31.57, 425.77)	1.54 (1.46 to 1.64)	0.75 (0.09, 2.33)	1.19 (0.17, 3.77)	1.53 (1.43 to 1.64)	338.65 (166.74, 598.03)	581.29 (290.09, 1003.21)	1.8 (1.72 to 1.89)	1.73 (0.50, 4.37)	2.96 (0.88, 7.45)	1.8 (1.71 to 1.89)
**Cameroon**	272.80 (163.76, 433.58)	371.56 (217.98, 607.11)	1.02 (0.99 to 1.06)	1.56 (0.54, 3.75)	2.14 (0.73, 5.06)	1.02 (0.97 to 1.06)	75.17 (31.56, 145.27)	105.79 (25.01, 250.33)	1.08 (1.01 to 1.14)	0.56 (0.15, 1.41)	0.78 (0.13, 2.23)	1.06 (0.97 to 1.13)	197.63 (109.40, 329.56)	265.77 (116.99, 487.94)	0.95 (0.92 to 0.98)	1.01 (0.32, 2.50)	1.35 (0.35, 3.63)	0.95 (0.92 to 0.99)
**Canada**	483.90 (260.86, 809.12)	606.59 (320.09, 1015.33)	0.73 (0.73 to 0.74)	2.94 (1.00, 6.78)	3.69 (1.25, 8.49)	0.73 (0.72 to 0.75)	204.26 (54.30, 464.62)	253.55 (69.21, 578.56)	0.69 (0.68 to 0.7)	1.51 (0.32, 4.07)	1.89 (0.39, 5.10)	0.68 (0.66 to 0.7)	279.64 (85.38, 563.56)	353.04 (107.25, 719.13)	0.77 (0.76 to 0.78)	1.43 (0.31, 3.88)	1.80 (0.38, 4.99)	0.78 (0.75 to 0.81)
**Central**	217.50	239.77	0.33	1.25	1.39	0.36	63.25	69.44	0.32	0.47	0.52	0.36	154.25	170.34	0.33	0.78	0.87	0.37

**African Republic**	(127.80, 357.29)	(141.97, 385.91)	(0.31 to 0.36)	(0.43, 3.09)	(0.47, 3.33)	(0.33 to 0.39)	(30.64, 114.21)	(17.16, 159.60)	(0.29 to 0.35)	(0.13, 1.18)	(0.08, 1.50)	(0.31 to 0.4)	(84.45, 262.20)	(74.47, 306.03)	(0.31 to 0.36)	(0.23, 1.98)	(0.24, 2.26)	(0.34 to 0.4)
**Chad**	132.22 (72.69, 219.38)	205.32 (120.85, 341.15)	1.42 (1.38 to 1.45)	0.73 (0.22, 1.78)	1.11 (0.36, 2.67)	1.37 (1.34 to 1.41)	21.05 (3.64, 55.49)	33.08 (5.55, 88.60)	1.37 (1.22 to 1.48)	0.16 (0.01, 0.50)	0.24 (0.02, 0.79)	1.27 (1.13 to 1.41)	111.16 (49.46, 195.57)	172.24 (85.48, 296.83)	1.42 (1.4 to 1.44)	0.57 (0.15, 1.51)	0.87 (0.25, 2.21)	1.38 (1.35 to 1.41)
**Chile**	441.85 (257.19, 741.88)	736.85 (422.59, 1233.19)	1.66 (1.65 to 1.68)	2.62 (0.90, 6.07)	4.36 (1.47, 10.16)	1.65 (1.63 to 1.66)	156.40 (44.80, 369.04)	249.98 (68.60, 588.37)	1.52 (1.5 to 1.53)	1.16 (0.25, 3.21)	1.87 (0.39, 5.38)	1.51 (1.49 to 1.53)	285.45 (112.34, 544.89)	486.87 (196.75, 919.20)	1.75 (1.73 to 1.77)	1.45 (0.37, 3.79)	2.49 (0.66, 6.54)	1.76 (1.73 to 1.78)
**China**	333.44 (191.52, 540.45)	601.73 (342.11, 988.48)	1.93 (1.9 to 1.95)	1.80 (0.60, 4.21)	3.29 (1.12, 7.77)	1.96 (1.94 to 1.98)	44.69 (6.21, 127.90)	96.41 (15.38, 270.07)	2.52 (2.5 to 2.53)	0.33 (0.04, 1.08)	0.72 (0.09, 2.37)	2.52 (2.5 to 2.54)	288.75 (152.77, 485.36)	505.32 (259.21, 871.64)	1.83 (1.8 to 1.85)	1.47 (0.47, 3.68)	2.57 (0.80, 6.49)	1.83 (1.8 to 1.85)
**Colombia**	537.06 (326.74, 837.68)	693.62 (433.88, 1061.37)	0.83 (0.81 to 0.86)	3.24 (1.19, 7.53)	4.12 (1.53, 9.32)	0.79 (0.76 to 0.82)	206.43 (102.84, 357.07)	246.72 (118.87, 441.38)	0.56 (0.54 to 0.59)	1.56 (0.50, 3.83)	1.85 (0.59, 4.57)	0.54 (0.51 to 0.58)	330.63 (185.95, 546.91)	446.90 (260.98, 706.83)	1.01 (0.94 to 1.07)	1.68 (0.56, 4.10)	2.27 (0.77, 5.33)	1.02 (0.95 to 1.07)
**Comoros**	261.00 (154.28,	310.75 (185.38,	0.57	1.54 (0.53,	1.83 (0.64,	0.58	90.90 (24.60,	107.71 (29.22,	0.59	0.67 (0.11,	0.80 (0.15,	0.54	170.10 (69.18,	203.04 (83.39,	0.56	0.86 (0.22,	1.04 (0.26,	0.57

	432.93)	507.12)	(0.56 to 0.58)	3.62)	4.21)	(0.55 to 0.6)	200.72)	243.13)	(0.55 to 0.62)	1.87)	2.22)	(0.49 to 0.59)	322.19)	383.96)	(0.54 to 0.58)	2.27)	2.76)	(0.54 to 0.6)
**Congo**	228.43 (132.91, 376.29)	318.35 (180.87, 523.68)	1.08 (1.05 to 1.1)	1.26 (0.41, 3.00)	1.76 (0.58, 4.18)	1.1 (1.07 to 1.12)	42.20 (7.20, 113.94)	57.88 (9.28, 157.88)	1.03 (0.9 to 1.15)	0.31 (0.02, 1.01)	0.43 (0.04, 1.36)	1.07 (0.94 to 1.18)	186.23 (91.78, 326.98)	260.47 (125.11, 453.67)	1.1 (1.07 to 1.13)	0.95 (0.26, 2.46)	1.33 (0.39, 3.37)	1.1 (1.06 to 1.14)
**Cook Islands**	769.50 (441.06, 1295.37)	1112.15 (645.07, 1849.26)	1.19 (1.16 to 1.21)	4.39 (1.48, 10.54)	6.30 (2.15, 14.71)	1.16 (1.14 to 1.17)	197.24 (42.57, 504.54)	280.66 (62.86, 717.44)	1.12 (1.1 to 1.13)	1.47 (0.23, 4.49)	2.07 (0.35, 6.31)	1.11 (1.1 to 1.13)	572.27 (250.91, 1046.13)	831.49 (377.31, 1501.69)	1.2 (1.19 to 1.22)	2.92 (0.84, 7.55)	4.23 (1.24, 10.94)	1.19 (1.17 to 1.2)
**Costa Rica**	740.52 (416.82, 1251.40)	958.44 (554.81, 1572.03)	0.85 (0.84 to 0.86)	4.23 (1.45, 10.01)	5.45 (1.86, 13.19)	0.83 (0.82 to 0.84)	193.82 (37.07, 516.51)	240.80 (47.01, 634.27)	0.69 (0.68 to 0.71)	1.44 (0.21, 4.42)	1.80 (0.26, 5.55)	0.7 (0.68 to 0.72)	546.70 (243.71, 998.19)	717.63 (328.09, 1287.51)	0.89 (0.87 to 0.9)	2.79 (0.82, 6.94)	3.66 (1.07, 9.46)	0.88 (0.87 to 0.9)
**Coted'Ivoire**	183.67 (111.76, 298.43)	308.91 (184.55, 501.41)	1.71 (1.68 to 1.73)	1.01 (0.34, 2.42)	1.71 (0.56, 4.09)	1.7 (1.66 to 1.74)	39.61 (16.25, 78.98)	60.94 (12.21, 156.51)	1.44 (1.4 to 1.47)	0.29 (0.06, 0.78)	0.45 (0.06, 1.35)	1.42 (1.37 to 1.47)	144.06 (82.29, 239.49)	247.97 (122.59, 427.91)	1.8 (1.76 to 1.83)	0.72 (0.22, 1.80)	1.26 (0.36, 3.23)	1.83 (1.79 to 1.88)
**Croatia**	59.54 (32.09, 103.41)	79.71 (42.80, 135.70)	0.96 (0.95	0.33 (0.09, 0.87)	0.45 (0.12, 1.13)	0.98 (0.96	12.73 (1.95, 35.73)	16.79 (2.72, 47.10)	0.91 (0.9 to	0.09 (0.01, 0.33)	0.13 (0.01, 0.43)	0.96 (0.89	46.81 (21.26, 87.66)	62.92 (29.20, 113.82)	0.97 (0.96	0.24 (0.05, 0.67)	0.32 (0.07, 0.89)	1 (0.97

			to 0.97)			to 1)			0.93)			to 1.02)			to 0.98)			to 1.02)
**Cuba**	495.15 (281.81, 804.22)	647.27 (370.06, 1073.54)	0.87 (0.86 to 0.88)	2.89 (1.00, 6.87)	3.77 (1.29, 9.04)	0.87 (0.85 to 0.88)	157.54 (33.38, 382.60)	204.14 (42.73, 493.25)	0.85 (0.83 to 0.88)	1.17 (0.18, 3.46)	1.52 (0.23, 4.37)	0.85 (0.82 to 0.88)	337.62 (150.07, 619.74)	443.13 (200.03, 817.93)	0.88 (0.87 to 0.89)	1.72 (0.49, 4.45)	2.25 (0.65, 5.74)	0.88 (0.87 to 0.89)
**Cyprus**	821.00 (465.55, 1340.87)	1138.00 (616.65, 1865.66)	1.06 (1.03 to 1.08)	5.02 (1.75, 11.81)	6.95 (2.36, 16.57)	1.04 (1.01 to 1.07)	357.02 (104.39, 774.28)	478.94 (128.19, 1048.85)	0.94 (0.92 to 0.96)	2.65 (0.60, 7.31)	3.59 (0.77, 9.76)	0.97 (0.94 to 0.99)	463.98 (172.40, 875.25)	659.06 (247.14, 1263.84)	1.15 (1.12 to 1.18)	2.37 (0.57, 6.43)	3.36 (0.85, 9.15)	1.13 (1.1 to 1.16)
**Czechia**	62.50 (33.74, 106.82)	80.03 (44.30, 133.76)	0.83 (0.8 to 0.86)	0.35 (0.09, 0.90)	0.44 (0.12, 1.13)	0.83 (0.8 to 0.87)	12.59 (1.94, 36.08)	16.01 (2.39, 45.04)	0.82 (0.78 to 0.86)	0.09 (0.01, 0.32)	0.12 (0.01, 0.43)	0.87 (0.83 to 0.91)	49.91 (23.21, 90.64)	64.02 (29.83, 116.18)	0.83 (0.82 to 0.85)	0.25 (0.05, 0.70)	0.32 (0.07, 0.86)	0.78 (0.74 to 0.81)
**Democratic People's Republic of Korea**	340.16 (192.05, 564.56)	380.75 (216.95, 638.02)	0.35 (0.34 to 0.36)	1.87 (0.60, 4.66)	2.08 (0.66, 5.06)	0.33 (0.29 to 0.35)	58.11 (8.37, 163.66)	64.68 (9.76, 180.51)	0.34 (0.33 to 0.35)	0.43 (0.03, 1.48)	0.48 (0.04, 1.61)	0.33 (0.29 to 0.37)	282.05 (143.38, 493.05)	316.07 (162.21, 551.16)	0.35 (0.34 to 0.36)	1.44 (0.43, 3.68)	1.60 (0.48, 4.06)	0.34 (0.3 to 0.37)
**Democratic Republic of the Congo**	173.63 (99.60, 290.11)	253.08 (144.15, 416.83)	1.24 (1.18 to 1.3)	0.98 (0.31, 2.41)	1.42 (0.47, 3.35)	1.23 (1.16 to	40.60 (7.54, 101.00)	59.77 (10.66, 149.75)	1.36 (1.22 to 1.5)	0.30 (0.02, 0.97)	0.44 (0.05, 1.33)	1.33 (1.19 to	133.03 (62.93, 237.02)	193.31 (90.62, 346.40)	1.23 (1.21 to	0.68 (0.18, 1.79)	0.98 (0.27, 2.54)	1.23 (1.19 to

						1.29)						1.48)			1.26)			1.26)
**Denmark**	664.12 (345.18, 1173.29)	953.11 (459.18, 1645.52)	1.17 (1.14 to 1.2)	4.29 (1.38, 10.37)	6.04 (1.87, 14.10)	1.11 (1.08 to 1.14)	381.50 (143.17, 794.34)	499.35 (131.23, 1094.39)	0.87 (0.85 to 0.89)	2.85 (0.75, 7.46)	3.72 (0.77, 9.92)	0.86 (0.84 to 0.88)	282.62 (115.48, 555.93)	453.76 (144.99, 961.10)	1.57 (1.52 to 1.63)	1.44 (0.36, 3.85)	2.32 (0.49, 6.55)	1.57 (1.53 to 1.62)
**Djibouti**	208.71 (123.50, 344.79)	355.46 (213.97, 590.64)	1.75 (1.73 to 1.77)	1.16 (0.37, 2.78)	1.98 (0.68, 4.74)	1.76 (1.73 to 1.78)	43.34 (9.34, 111.15)	74.94 (16.00, 191.13)	1.79 (1.77 to 1.8)	0.32 (0.02, 1.01)	0.56 (0.06, 1.74)	1.79 (1.75 to 1.84)	165.37 (80.43, 293.94)	280.52 (135.02, 495.08)	1.74 (1.72 to 1.75)	0.84 (0.23, 2.18)	1.43 (0.41, 3.56)	1.72 (1.7 to 1.75)
**Dominica**	450.10 (255.34, 740.65)	609.08 (335.42, 1007.08)	0.98 (0.97 to 1)	2.64 (0.87, 6.16)	3.57 (1.16, 8.56)	0.99 (0.97 to 1.01)	151.19 (32.85, 358.20)	200.69 (43.55, 485.90)	0.91 (0.89 to 0.93)	1.12 (0.18, 3.24)	1.49 (0.24, 4.29)	0.94 (0.91 to 0.97)	298.91 (128.83, 551.69)	408.40 (179.32, 758.15)	1.01 (1 to 1.03)	1.52 (0.41, 3.99)	2.08 (0.58, 5.40)	1.02 (1 to 1.04)
**Dominican Republic**	379.92 (224.93, 600.31)	599.82 (337.58, 996.89)	1.5 (1.49 to 1.52)	2.27 (0.79, 5.36)	3.46 (1.16, 8.38)	1.38 (1.36 to 1.4)	146.57 (68.26, 267.39)	176.10 (35.54, 442.98)	0.6 (0.55 to 0.65)	1.09 (0.33, 2.69)	1.31 (0.19, 4.02)	0.6 (0.53 to 0.65)	233.34 (128.81, 393.73)	423.72 (193.65, 772.22)	1.95 (1.93 to 1.97)	1.18 (0.37, 2.94)	2.16 (0.61, 5.65)	1.97 (1.94 to 2)
**Ecuador**	804.41 (497.05, 1209.96)	1049.89 (448.36, 1820.99)	0.85 (0.78 to 0.91)	4.52 (1.60, 10.26)	5.88 (1.69, 14.55)	0.87 (0.8 to 0.94)	180.19 (83.42, 325.67)	220.90 (51.78, 555.41)	0.62 (0.58 to 0.68)	1.34 (0.40, 3.28)	1.65 (0.29, 4.91)	0.64 (0.61 to 0.69)	624.22 (365.03, 979.66)	828.99 (269.02, 1570.42)	0.91 (0.83 to 0.99)	3.17 (1.07, 7.53)	4.23 (0.99, 11.31)	0.93 (0.84 to 1)

**Egypt**	745.82 (452.30, 1181.97)	921.84 (527.40, 1503.28)	0.73 (0.68 to 0.79)	4.68 (1.72, 10.47)	5.53 (1.95, 12.84)	0.56 (0.52 to 0.6)	378.37 (218.28, 606.83)	348.43 (100.41, 763.87)	-0.25 (-0.29 to -0.21)	2.82 (1.03, 6.35)	2.59 (0.56, 6.82)	-0.26 (-0.3 to -0.22)	367.45 (198.66, 624.79)	573.41 (219.42, 1076.37)	1.47 (1.39 to 1.54)	1.86 (0.58, 4.44)	2.94 (0.73, 7.70)	1.51 (1.43 to 1.58)
**El Salvador**	581.02 (344.66, 941.93)	787.05 (444.87, 1309.29)	1.02 (0.99 to 1.04)	3.35 (1.17, 7.84)	4.43 (1.50, 10.50)	0.94 (0.91 to 0.97)	170.51 (68.54, 339.91)	180.50 (35.86, 465.38)	0.21 (0.17 to 0.24)	1.27 (0.34, 3.29)	1.35 (0.20, 4.11)	0.21 (0.17 to 0.24)	410.51 (222.18, 699.80)	606.55 (250.47, 1107.92)	1.3 (1.27 to 1.33)	2.08 (0.68, 5.02)	3.09 (0.88, 7.96)	1.3 (1.26 to 1.33)
**Equatorial Guinea**	190.37 (111.99, 314.05)	442.66 (251.67, 714.82)	2.77 (2.73 to 2.83)	1.07 (0.35, 2.57)	2.48 (0.83, 5.86)	2.77 (2.72 to 2.83)	44.81 (8.90, 113.14)	100.91 (19.80, 256.48)	2.71 (2.64 to 2.78)	0.33 (0.03, 1.02)	0.75 (0.10, 2.22)	2.71 (2.65 to 2.78)	145.56 (66.81, 259.04)	341.75 (159.19, 605.64)	2.81 (2.77 to 2.86)	0.74 (0.19, 1.95)	1.73 (0.49, 4.35)	2.81 (2.77 to 2.87)
**Eritrea**	152.77 (92.60, 248.29)	233.45 (137.90, 382.34)	1.4 (1.37 to 1.42)	0.88 (0.29, 2.13)	1.33 (0.45, 3.21)	1.33 (1.31 to 1.35)	44.09 (21.36, 80.67)	59.71 (13.73, 143.33)	1.04 (1 to 1.08)	0.33 (0.08, 0.86)	0.44 (0.06, 1.35)	0.95 (0.9 to 1)	108.67 (62.36, 180.76)	173.73 (80.08, 310.55)	1.54 (1.52 to 1.56)	0.55 (0.16, 1.41)	0.88 (0.24, 2.35)	1.55 (1.53 to 1.59)
**Estonia**	83.13 (45.73, 139.85)	118.27 (66.70, 200.49)	1.17 (1.14 to 1.19)	0.49 (0.13, 1.23)	0.69 (0.20, 1.72)	1.15 (1.11 to 1.18)	28.12 (4.87, 67.54)	37.95 (6.33, 91.39)	1 (0.97 to 1.02)	0.21 (0.01, 0.64)	0.28 (0.02, 0.86)	1.03 (0.99 to 1.06)	55.01 (23.17, 104.21)	80.32 (35.18, 152.34)	1.26 (1.24 to 1.28)	0.28 (0.06, 0.81)	0.41 (0.09, 1.12)	1.22 (1.17 to 1.26)
**Eswatini**	332.39	409.32	0.71	1.93	2.36	0.69	98.44	119.56	0.64	0.73	0.88	0.63	233.95	289.76	0.73	1.20	1.47	0.71

	(180.99, 558.89)	(228.61, 667.89)	(0.68 to 0.75)	(0.62, 4.62)	(0.78, 5.66)	(0.66 to 0.74)	(19.65, 245.12)	(24.41, 297.06)	(0.61 to 0.66)	(0.10, 2.15)	(0.12, 2.70)	(0.59 to 0.67)	(97.33, 430.77)	(120.17, 529.14)	(0.68 to 0.79)	(0.31, 3.23)	(0.39, 3.71)	(0.64 to 0.79)
**Ethiopia**	154.35 (92.24, 250.80)	235.08 (137.58, 385.99)	1.38 (1.36 to 1.4)	0.87 (0.31, 2.03)	1.34 (0.47, 3.15)	1.41 (1.4 to 1.43)	39.07 (8.68, 95.58)	59.17 (13.13, 144.65)	1.36 (1.31 to 1.4)	0.29 (0.05, 0.88)	0.44 (0.08, 1.28)	1.36 (1.3 to 1.41)	115.27 (54.71, 204.11)	175.91 (83.53, 314.70)	1.38 (1.36 to 1.41)	0.58 (0.17, 1.48)	0.90 (0.26, 2.29)	1.39 (1.36 to 1.42)
**Fiji**	622.60 (352.55, 1041.45)	928.18 (532.86, 1581.57)	1.28 (1.27 to 1.29)	3.60 (1.19, 8.54)	5.37 (1.81, 12.46)	1.29 (1.28 to 1.3)	187.38 (42.00, 452.12)	276.66 (64.36, 683.52)	1.26 (1.25 to 1.27)	1.39 (0.24, 4.08)	2.05 (0.37, 6.05)	1.27 (1.25 to 1.28)	435.22 (182.86, 812.50)	651.52 (277.88, 1232.22)	1.29 (1.28 to 1.3)	2.22 (0.59, 5.87)	3.31 (0.92, 8.65)	1.3 (1.29 to 1.32)
**Finland**	817.17 (479.00, 1323.01)	1098.98 (600.87, 1825.81)	0.96 (0.95 to 0.97)	5.05 (1.78, 11.70)	6.75 (2.33, 15.47)	0.94 (0.92 to 0.95)	379.45 (178.81, 710.07)	482.38 (134.78, 1056.14)	0.78 (0.77 to 0.78)	2.83 (0.90, 6.67)	3.59 (0.80, 9.42)	0.77 (0.76 to 0.78)	437.72 (209.69, 770.27)	616.60 (229.56, 1176.91)	1.11 (1.08 to 1.14)	2.23 (0.66, 5.60)	3.16 (0.79, 8.61)	1.11 (1.09 to 1.14)
**France**	933.11 (549.28, 1489.35)	1174.38 (673.55, 1936.78)	0.74 (0.73 to 0.75)	5.90 (2.16, 13.32)	7.36 (2.65, 17.71)	0.71 (0.69 to 0.72)	486.71 (240.80, 860.42)	573.22 (192.59, 1208.22)	0.52 (0.49 to 0.53)	3.63 (1.22, 8.46)	4.28 (1.08, 11.10)	0.52 (0.5 to 0.54)	446.39 (203.72, 805.48)	601.16 (210.30, 1151.67)	0.98 (0.96 to 0.99)	2.27 (0.65, 5.64)	3.08 (0.76, 8.48)	1.01 (0.99 to 1.02)
**Gabon**	265.86 (155.27,	400.46 (233.40,	1.36	1.50 (0.51,	2.25 (0.74,	1.33	61.31 (12.62,	91.02 (18.32,	1.3	0.46 (0.05,	0.67 (0.09,	1.25	204.55 (93.70,	309.44 (141.99,	1.37	1.04 (0.27,	1.57 (0.43,	1.36

	428.97)	652.27)	(1.32 to 1.39)	3.56)	5.30)	(1.31 to 1.36)	155.61)	231.25)	(1.27 to 1.33)	1.41)	2.01)	(1.21 to 1.29)	354.48)	546.74)	(1.34 to 1.41)	2.75)	4.07)	(1.33 to 1.39)
**Gambia**	186.54 (110.83, 305.31)	279.44 (163.67, 455.46)	1.33 (1.3 to 1.36)	1.04 (0.35, 2.49)	1.56 (0.51, 3.65)	1.34 (1.3 to 1.38)	40.79 (8.48, 101.45)	60.37 (12.11, 151.70)	1.31 (1.27 to 1.36)	0.30 (0.02, 0.96)	0.45 (0.04, 1.37)	1.34 (1.29 to 1.4)	145.75 (68.56, 256.84)	219.08 (103.77, 383.19)	1.33 (1.3 to 1.36)	0.74 (0.20, 1.91)	1.11 (0.31, 2.85)	1.35 (1.31 to 1.4)
**Georgia**	157.81 (89.42, 259.72)	270.45 (149.54, 441.82)	1.78 (1.74 to 1.81)	0.90 (0.29, 2.25)	1.54 (0.50, 3.74)	1.77 (1.74 to 1.8)	40.73 (7.21, 104.71)	67.68 (12.13, 173.24)	1.67 (1.64 to 1.69)	0.30 (0.02, 0.97)	0.50 (0.05, 1.56)	1.67 (1.63 to 1.7)	117.08 (52.97, 212.80)	202.77 (89.53, 355.73)	1.82 (1.78 to 1.85)	0.60 (0.15, 1.60)	1.04 (0.29, 2.69)	1.82 (1.79 to 1.86)
**Germany**	742.82 (409.37, 1233.54)	966.98 (495.49, 1624.45)	0.87 (0.85 to 0.88)	4.69 (1.64, 11.07)	5.89 (1.91, 13.66)	0.74 (0.72 to 0.75)	380.67 (152.24, 756.06)	402.08 (101.39, 912.27)	0.16 (0.14 to 0.19)	2.85 (0.79, 6.98)	3.00 (0.56, 8.16)	0.14 (0.12 to 0.18)	362.15 (166.91, 670.59)	564.90 (207.07, 1102.18)	1.47 (1.44 to 1.5)	1.84 (0.51, 4.83)	2.89 (0.74, 7.77)	1.48 (1.45 to 1.51)
**Ghana**	190.02 (116.38, 307.83)	287.22 (170.07, 467.60)	1.37 (1.29 to 1.45)	1.05 (0.35, 2.50)	1.59 (0.51, 3.72)	1.34 (1.24 to 1.46)	38.83 (17.70, 73.94)	56.23 (10.36, 144.84)	1.27 (0.94 to 1.62)	0.29 (0.06, 0.77)	0.42 (0.04, 1.31)	1.34 (1.05 to 1.69)	151.19 (87.88, 247.13)	230.99 (111.21, 400.27)	1.36 (1.34 to 1.38)	0.76 (0.24, 1.87)	1.18 (0.33, 3.04)	1.39 (1.35 to 1.42)
**Greece**	1002.72 (547.35, 1628.19)	1201.55 (657.76, 1955.09)	0.59 (0.58	6.11 (2.10, 14.61)	7.30 (2.47, 17.33)	0.57 (0.56	421.67 (120.07, 930.12)	492.57 (131.72, 1106.29)	0.49 (0.48	3.14 (0.70, 8.58)	3.68 (0.76, 9.77)	0.49 (0.47	581.05 (216.86, 1100.88)	708.98 (270.25, 1339.59)	0.64 (0.63	2.97 (0.74, 8.16)	3.63 (0.97, 9.75)	0.64 (0.63

			to 0.6)			to 0.58)			to 0.5)			to 0.5)			to 0.66)			to 0.65)
**Greenland**	415.32 (225.09, 696.43)	540.86 (290.15, 900.61)	0.87 (0.86 to 0.88)	2.54 (0.84, 5.81)	3.30 (1.12, 7.57)	0.86 (0.84 to 0.87)	182.80 (48.02, 413.11)	231.80 (61.95, 520.26)	0.78 (0.77 to 0.79)	1.36 (0.26, 3.67)	1.72 (0.36, 4.64)	0.77 (0.75 to 0.78)	232.52 (68.99, 473.11)	309.06 (93.78, 625.51)	0.94 (0.93 to 0.95)	1.19 (0.24, 3.33)	1.58 (0.33, 4.31)	0.95 (0.92 to 0.98)
**Grenada**	390.62 (220.64, 635.39)	552.53 (313.91, 906.13)	1.14 (1.12 to 1.16)	2.30 (0.75, 5.46)	3.24 (1.09, 7.67)	1.13 (1.11 to 1.16)	131.02 (27.09, 308.31)	182.27 (39.06, 435.72)	1.08 (1.06 to 1.1)	0.98 (0.15, 2.83)	1.35 (0.21, 3.89)	1.06 (1.03 to 1.08)	259.60 (111.85, 475.95)	370.26 (164.20, 681.78)	1.17 (1.15 to 1.2)	1.32 (0.35, 3.50)	1.89 (0.53, 4.99)	1.19 (1.16 to 1.21)
**Guam**	818.63 (461.91, 1377.60)	1122.39 (656.72, 1874.09)	1.02 (1 to 1.04)	4.68 (1.59, 11.18)	6.40 (2.21, 15.31)	1.01 (0.99 to 1.02)	214.25 (45.97, 554.58)	293.85 (66.37, 743.66)	1.02 (1 to 1.04)	1.59 (0.27, 4.77)	2.18 (0.39, 6.49)	1 (0.99 to 1.02)	604.38 (266.79, 1098.71)	828.54 (370.26, 1504.31)	1.02 (1 to 1.03)	3.08 (0.88, 8.04)	4.23 (1.20, 11.03)	1.01 (0.99 to 1.03)
**Guatemala**	507.11 (303.95, 809.31)	727.09 (412.64, 1220.95)	1.12 (1.03 to 1.18)	2.81 (1.00, 6.59)	4.00 (1.36, 9.41)	1.09 (1 to 1.14)	100.25 (40.09, 200.68)	134.27 (23.59, 377.27)	0.95 (0.94 to 0.96)	0.75 (0.18, 1.96)	0.99 (0.12, 3.30)	0.91 (0.88 to 0.94)	406.86 (238.34, 665.93)	592.81 (288.27, 1047.09)	1.16 (1.05 to 1.24)	2.06 (0.69, 4.97)	3.01 (0.92, 7.62)	1.16 (1.04 to 1.23)
**Guinea**	168.38 (98.60, 276.32)	254.90 (156.40, 409.85)	1.34 (1.32 to	0.93 (0.30, 2.28)	1.40 (0.47, 3.39)	1.35 (1.33 to	32.26 (6.50, 82.08)	45.97 (18.07, 91.08)	1.15 (1.05 to	0.23 (0.02, 0.76)	0.34 (0.07, 0.92)	1.31 (1.23 to 1.4)	136.11 (65.85, 235.53)	208.93 (122.10, 338.42)	1.39 (1.36 to	0.69 (0.19, 1.82)	1.05 (0.33, 2.62)	1.37 (1.34 to 1.4)

			1.37)			1.37)			1.23)						1.42)			
**Guinea-Bissau**	165.51 (93.65, 279.51)	255.10 (151.42, 424.38)	1.42 (1.39 to 1.45)	0.93 (0.30, 2.14)	1.43 (0.48, 3.42)	1.44 (1.4 to 1.47)	37.55 (7.85, 92.88)	56.61 (11.60, 140.40)	1.36 (1.32 to 1.4)	0.28 (0.02, 0.84)	0.42 (0.05, 1.30)	1.4 (1.33 to 1.47)	127.96 (54.47, 231.17)	198.49 (93.57, 351.17)	1.44 (1.41 to 1.47)	0.65 (0.17, 1.68)	1.01 (0.27, 2.62)	1.44 (1.4 to 1.47)
**Guyana**	410.67 (236.37, 662.55)	587.56 (334.97, 961.67)	1.19 (1.15 to 1.23)	2.38 (0.79, 5.58)	3.43 (1.18, 8.21)	1.21 (1.18 to 1.23)	130.88 (28.69, 312.36)	182.32 (39.19, 434.42)	1.12 (1.06 to 1.17)	0.96 (0.15, 2.73)	1.36 (0.20, 4.01)	1.13 (1.08 to 1.18)	279.79 (124.26, 510.91)	405.24 (180.07, 743.93)	1.22 (1.19 to 1.25)	1.42 (0.38, 3.76)	2.07 (0.58, 5.24)	1.25 (1.22 to 1.28)
**Haiti**	306.77 (184.92, 494.01)	366.67 (218.00, 583.25)	0.59 (0.58 to 0.6)	1.81 (0.64, 4.29)	2.07 (0.69, 4.80)	0.46 (0.44 to 0.48)	107.19 (48.23, 195.75)	92.44 (36.81, 183.98)	-0.48 (-0.53 to -0.43)	0.79 (0.22, 2.03)	0.68 (0.16, 1.82)	-0.49 (-0.58 to -0.43)	199.58 (109.16, 345.63)	274.23 (155.27, 455.96)	1.04 (1.01 to 1.07)	1.01 (0.31, 2.56)	1.39 (0.44, 3.34)	1.04 (1.01 to 1.08)
**Honduras**	499.52 (289.65, 831.42)	733.63 (420.76, 1213.49)	1.25 (1.22 to 1.26)	2.79 (0.93, 6.81)	4.07 (1.37, 9.69)	1.23 (1.21 to 1.25)	103.02 (17.73, 296.25)	143.77 (25.29, 400.27)	1.14 (1.08 to 1.21)	0.77 (0.09, 2.49)	1.07 (0.13, 3.49)	1.08 (1.02 to 1.15)	396.51 (191.19, 703.38)	589.86 (283.82, 1045.80)	1.28 (1.25 to 1.3)	2.02 (0.59, 5.25)	3.01 (0.91, 7.71)	1.28 (1.24 to 1.3)
**Hungary**	65.72 (34.99, 110.92)	81.03 (44.31, 137.45)	0.68 (0.67 to 0.7)	0.37 (0.10, 0.91)	0.45 (0.13, 1.11)	0.67 (0.64 to 0.69)	14.15 (2.25, 40.46)	17.17 (2.79, 48.96)	0.63 (0.62 to 0.64)	0.11 (0.01, 0.38)	0.13 (0.01, 0.45)	0.63 (0.58 to 0.68)	51.57 (23.25, 93.81)	63.86 (29.45, 115.83)	0.7 (0.68 to 0.71)	0.26 (0.06, 0.71)	0.32 (0.08, 0.86)	0.67 (0.64 to 0.71)

**Iceland**	1039.08 (575.83, 1693.16)	1268.70 (698.73, 2054.94)	0.65 (0.64 to 0.66)	6.34 (2.22, 14.96)	7.70 (2.64, 17.98)	0.63 (0.62 to 0.65)	442.62 (127.68, 960.28)	514.63 (135.35, 1154.35)	0.49 (0.47 to 0.5)	3.29 (0.73, 8.97)	3.85 (0.82, 10.41)	0.5 (0.48 to 0.51)	596.45 (226.47, 1129.34)	754.07 (290.19, 1417.46)	0.77 (0.76 to 0.78)	3.05 (0.77, 8.15)	3.85 (1.01, 10.15)	0.76 (0.75 to 0.77)
**India**	257.03 (160.05, 399.54)	496.11 (304.64, 777.05)	2.18 (2.15 to 2.21)	1.52 (0.58, 3.50)	2.84 (1.05, 6.55)	2.06 (2.03 to 2.08)	93.23 (29.21, 192.85)	139.07 (31.36, 332.11)	1.32 (1.28 to 1.37)	0.69 (0.17, 1.81)	1.03 (0.20, 2.98)	1.32 (1.27 to 1.37)	163.80 (70.81, 295.31)	357.05 (161.00, 615.85)	2.6 (2.56 to 2.64)	0.83 (0.24, 2.15)	1.81 (0.55, 4.64)	2.6 (2.56 to 2.65)
**Indonesia**	603.26 (346.77, 982.62)	1096.94 (631.48, 1797.37)	1.95 (1.92 to 1.98)	3.51 (1.23, 7.94)	6.32 (2.20, 14.62)	1.92 (1.9 to 1.93)	190.31 (46.99, 438.64)	316.39 (72.14, 787.24)	1.74 (1.62 to 1.83)	1.41 (0.29, 3.93)	2.35 (0.45, 6.81)	1.74 (1.61 to 1.83)	412.95 (182.34, 740.10)	780.56 (357.98, 1372.75)	2.08 (2.05 to 2.11)	2.10 (0.61, 5.42)	3.97 (1.17, 10.19)	2.08 (2.06 to 2.11)
**Iran (Islamic Republic of)**	663.63 (389.92, 1071.00)	869.51 (502.91, 1389.58)	0.89 (0.85 to 0.92)	4.22 (1.55, 9.51)	5.53 (2.01, 12.44)	0.91 (0.86 to 0.95)	355.38 (127.08, 687.12)	463.94 (164.19, 899.85)	0.88 (0.81 to 0.94)	2.64 (0.74, 6.45)	3.46 (0.96, 8.66)	0.89 (0.82 to 0.94)	308.25 (98.79, 634.50)	405.57 (130.54, 840.68)	0.92 (0.86 to 0.98)	1.57 (0.37, 4.30)	2.07 (0.48, 5.58)	0.92 (0.86 to 0.98)
**Iraq**	708.51 (412.03, 1155.82)	814.41 (464.52, 1318.36)	0.47 (0.45 to 0.49)	4.15 (1.49, 9.89)	4.76 (1.67, 10.98)	0.46 (0.42 to 0.49)	235.25 (60.56, 543.53)	262.67 (68.13, 603.33)	0.4 (0.36 to 0.43)	1.75 (0.35, 4.80)	1.94 (0.39, 5.42)	0.38 (0.34 to 0.41)	473.27 (190.96, 884.31)	551.75 (225.11, 1020.38)	0.51 (0.49 to 0.53)	2.41 (0.63, 6.33)	2.81 (0.76, 7.24)	0.52 (0.49 to 0.54)
**Ireland**	971.00	1177.77	0.63	5.94	7.15	0.62	413.58	480.08	0.48	3.08	3.58	0.48	557.42	697.68	0.73	2.85	3.57	0.73

	(530.96, 1589.43)	(645.00, 1938.38)	(0.61 to 0.64)	(2.02, 13.95)	(2.42, 16.47)	(0.61 to 0.64)	(118.01, 910.94)	(129.11, 1089.35)	(0.48 to 0.48)	(0.70, 8.26)	(0.77, 9.72)	(0.48 to 0.49)	(209.19, 1063.20)	(266.21, 1312.41)	(0.72 to 0.75)	(0.73, 7.77)	(0.95, 9.56)	(0.71 to 0.75)
**Israel**	813.37 (446.43, 1364.83)	1041.87 (556.33, 1743.23)	0.8 (0.79 to 0.81)	4.93 (1.60, 11.80)	6.27 (2.06, 14.47)	0.78 (0.76 to 0.79)	330.51 (90.62, 743.49)	401.57 (101.81, 917.89)	0.62 (0.61 to 0.63)	2.46 (0.51, 6.60)	3.00 (0.61, 8.28)	0.64 (0.62 to 0.65)	482.86 (185.54, 938.77)	640.30 (245.32, 1203.18)	0.91 (0.9 to 0.92)	2.47 (0.62, 6.59)	3.27 (0.84, 8.74)	0.91 (0.89 to 0.92)
**Italy**	2089.65 (962.82, 3673.85)	2345.40 (1203.36, 3818.99)	0.38 (0.36 to 0.41)	12.26 (3.84, 30.00)	13.93 (4.73, 33.17)	0.42 (0.39 to 0.44)	666.06 (150.59, 1681.79)	815.68 (213.41, 1858.69)	0.68 (0.65 to 0.71)	4.97 (0.96, 15.06)	6.11 (1.28, 16.68)	0.68 (0.65 to 0.71)	1423.60 (511.62, 2794.79)	1529.72 (599.62, 2844.24)	0.27 (0.24 to 0.3)	7.28 (1.81, 19.32)	7.82 (2.11, 20.59)	0.27 (0.24 to 0.3)
**Jamaica**	444.88 (252.24, 711.40)	592.34 (340.33, 973.76)	0.94 (0.92 to 0.96)	2.60 (0.87, 6.10)	3.46 (1.16, 8.09)	0.93 (0.9 to 0.95)	145.40 (32.53, 335.89)	190.02 (40.86, 448.08)	0.87 (0.84 to 0.91)	1.08 (0.18, 3.18)	1.42 (0.24, 4.06)	0.88 (0.85 to 0.92)	299.47 (132.41, 553.69)	402.31 (179.49, 741.40)	0.96 (0.93 to 0.98)	1.52 (0.41, 4.03)	2.05 (0.57, 5.28)	0.96 (0.94 to 0.99)
**Japan**	1626.58 (729.17, 2831.84)	1700.92 (751.12, 2953.00)	0.18 (0.16 to 0.2)	9.23 (2.74, 22.01)	9.58 (2.82, 23.14)	0.15 (0.13 to 0.17)	399.91 (98.88, 1008.64)	390.96 (94.93, 998.46)	-0.07 (-0.08 to -0.07)	2.98 (0.60, 8.95)	2.91 (0.55, 8.83)	-0.07 (-0.07 to -0.06)	1226.67 (451.53, 2333.35)	1309.96 (476.37, 2475.15)	0.24 (0.22 to 0.27)	6.25 (1.57, 16.53)	6.67 (1.70, 17.74)	0.24 (0.22 to 0.27)
**Jordan**	646.53 (387.74,	816.96 (490.31,	0.77	3.92 (1.43,	4.80 (1.73,	0.68	266.00 (109.54,	266.27 (120.83,	-0.08	1.98 (0.57,	2.00 (0.60,	-0.02	380.54 (176.77,	550.68 (303.17,	1.22	1.94 (0.56,	2.80 (0.92,	1.22

	1028.21)	1278.64)	(0.75 to 0.79)	8.92)	11.27)	(0.65 to 0.71)	508.28)	492.73)	(-0.15 to 0)	4.74)	4.84)	(-0.1 to 0.06)	687.41)	902.11)	(1.15 to 1.29)	4.92)	6.92)	(1.15 to 1.3)
**Kazakhstan**	142.13 (82.39, 236.30)	199.47 (113.29, 327.24)	1.1 (1.09 to 1.11)	0.83 (0.26, 2.01)	1.14 (0.36, 2.78)	1.07 (1.04 to 1.09)	45.11 (15.55, 93.10)	54.72 (9.82, 136.83)	0.63 (0.61 to 0.64)	0.34 (0.06, 0.91)	0.41 (0.04, 1.27)	0.59 (0.56 to 0.62)	97.02 (50.33, 171.84)	144.75 (63.50, 260.65)	1.32 (1.3 to 1.35)	0.49 (0.13, 1.22)	0.73 (0.19, 1.94)	1.35 (1.32 to 1.39)
**Kenya**	234.01 (134.16, 383.02)	295.39 (176.64, 479.52)	0.85 (0.68 to 1)	1.32 (0.47, 3.06)	1.64 (0.58, 3.79)	0.84 (0.67 to 1)	54.49 (10.54, 138.60)	59.42 (11.73, 154.00)	0.43 (0.23 to 0.62)	0.40 (0.07, 1.22)	0.44 (0.07, 1.34)	0.43 (0.24 to 0.63)	179.52 (86.46, 315.04)	235.97 (117.82, 402.50)	0.92 (0.76 to 1.07)	0.91 (0.27, 2.30)	1.20 (0.37, 3.05)	0.92 (0.76 to 1.07)
**Kiribati**	520.17 (295.68, 866.11)	747.12 (434.08, 1227.30)	1.2 (1.18 to 1.23)	2.97 (0.97, 7.20)	4.26 (1.47, 9.91)	1.18 (1.15 to 1.21)	137.82 (29.05, 350.43)	196.30 (41.57, 500.81)	1.14 (1.11 to 1.17)	1.02 (0.16, 3.14)	1.45 (0.25, 4.38)	1.15 (1.12 to 1.18)	382.35 (168.83, 705.95)	550.82 (244.80, 1010.15)	1.22 (1.19 to 1.24)	1.95 (0.53, 5.19)	2.81 (0.81, 7.31)	1.19 (1.16 to 1.22)
**Kuwait**	893.44 (514.35, 1459.66)	1069.42 (616.68, 1728.22)	0.61 (0.59 to 0.63)	5.23 (1.81, 12.11)	6.24 (2.22, 14.83)	0.59 (0.56 to 0.61)	287.59 (76.74, 657.46)	338.86 (89.26, 784.77)	0.56 (0.54 to 0.58)	2.14 (0.44, 5.88)	2.52 (0.52, 7.11)	0.57 (0.55 to 0.59)	605.84 (246.10, 1128.14)	730.56 (295.12, 1325.32)	0.63 (0.61 to 0.65)	3.09 (0.80, 7.96)	3.72 (1.00, 9.86)	0.63 (0.61 to 0.66)
**Kyrgyzstan**	132.53 (78.02, 214.84)	159.75 (94.96, 259.91)	0.61 (0.59	0.75 (0.24, 1.85)	0.90 (0.29, 2.13)	0.58 (0.55	31.88 (5.76, 81.36)	37.12 (6.69, 96.56)	0.5 (0.48	0.24 (0.02, 0.76)	0.27 (0.02, 0.86)	0.46 (0.41	100.65 (47.30, 177.77)	122.64 (57.75, 218.82)	0.64 (0.62	0.51 (0.14, 1.34)	0.62 (0.16, 1.59)	0.67 (0.63

			to 0.63)			to 0.61)			to 0.53)			to 0.51)			to 0.67)			to 0.71)
**Lao People's Democratic Republic**	495.78 (287.46, 827.26)	888.17 (525.80, 1480.58)	1.93 (1.91 to 1.94)	2.87 (0.94, 6.68)	5.12 (1.77, 12.01)	1.9 (1.88 to 1.92)	150.50 (32.94, 359.47)	255.89 (59.94, 646.59)	1.75 (1.73 to 1.76)	1.11 (0.17, 3.15)	1.90 (0.35, 5.68)	1.77 (1.74 to 1.8)	345.28 (162.74, 614.17)	632.28 (289.34, 1120.54)	1.98 (1.97 to 1.99)	1.76 (0.49, 4.42)	3.22 (0.93, 8.32)	1.99 (1.97 to 2.01)
**Latvia**	82.25 (45.78, 139.22)	108.66 (60.51, 180.31)	0.9 (0.89 to 0.91)	0.48 (0.14, 1.21)	0.63 (0.19, 1.54)	0.83 (0.8 to 0.86)	28.18 (9.10, 56.68)	31.88 (5.03, 77.61)	0.4 (0.39 to 0.42)	0.21 (0.03, 0.57)	0.24 (0.01, 0.75)	0.39 (0.36 to 0.42)	54.07 (27.48, 96.97)	76.78 (34.79, 141.51)	1.14 (1.12 to 1.16)	0.27 (0.06, 0.74)	0.39 (0.09, 1.07)	1.14 (1.11 to 1.16)
**Lebanon**	724.20 (420.44, 1179.06)	948.36 (560.04, 1544.62)	0.9 (0.88 to 0.92)	4.17 (1.46, 9.88)	5.44 (1.90, 12.68)	0.9 (0.89 to 0.91)	204.55 (49.20, 488.47)	258.55 (60.31, 623.40)	0.76 (0.74 to 0.78)	1.52 (0.28, 4.49)	1.93 (0.35, 5.50)	0.77 (0.75 to 0.78)	519.66 (222.75, 934.48)	689.82 (297.44, 1233.79)	0.94 (0.92 to 0.96)	2.64 (0.73, 6.75)	3.52 (0.98, 8.97)	0.96 (0.93 to 0.98)
**Lesotho**	245.14 (143.28, 398.08)	362.81 (209.72, 593.24)	1.3 (1.26 to 1.35)	1.41 (0.48, 3.37)	2.08 (0.71, 4.93)	1.29 (1.26 to 1.33)	68.35 (12.88, 168.35)	101.81 (18.47, 254.00)	1.33 (1.29 to 1.37)	0.51 (0.05, 1.52)	0.75 (0.10, 2.35)	1.25 (1.19 to 1.29)	176.79 (80.16, 318.20)	261.01 (119.90, 472.33)	1.31 (1.25 to 1.38)	0.90 (0.25, 2.40)	1.33 (0.37, 3.40)	1.3 (1.24 to 1.37)
**Liberia**	193.15 (115.44, 310.55)	286.29 (169.63, 468.49)	1.29 (1.25 to	1.09 (0.36, 2.57)	1.57 (0.52, 3.81)	1.18 (1.16 to	50.40 (21.60, 95.76)	54.66 (10.38, 142.14)	0.3 (0.21 to	0.37 (0.08, 0.96)	0.40 (0.04, 1.27)	0.29 (0.17 to	142.75 (78.96, 241.10)	231.63 (113.97, 399.33)	1.57 (1.53 to 1.6)	0.72 (0.21, 1.79)	1.17 (0.34, 2.99)	1.56 (1.52 to 1.6)

			1.32)			1.21)			0.39)			0.39)						
**Libya**	781.25 (450.16, 1259.10)	880.03 (502.77, 1431.22)	0.42 (0.41 to 0.44)	4.63 (1.61, 10.85)	5.22 (1.81, 12.31)	0.42 (0.41 to 0.43)	270.62 (98.82, 550.06)	309.76 (84.50, 691.11)	0.47 (0.45 to 0.48)	2.02 (0.52, 5.12)	2.31 (0.48, 6.31)	0.46 (0.44 to 0.47)	510.63 (237.88, 917.22)	570.27 (225.91, 1065.41)	0.39 (0.38 to 0.41)	2.61 (0.77, 6.61)	2.91 (0.76, 7.58)	0.39 (0.37 to 0.41)
**Lithuania**	75.62 (43.48, 126.76)	105.01 (58.59, 175.67)	1.08 (1.06 to 1.09)	0.44 (0.13, 1.06)	0.60 (0.17, 1.52)	1.06 (1.03 to 1.08)	22.87 (8.32, 45.76)	28.20 (4.08, 70.27)	0.69 (0.68 to 0.71)	0.17 (0.02, 0.47)	0.21 (0.01, 0.65)	0.69 (0.65 to 0.74)	52.75 (27.62, 93.45)	76.81 (35.80, 140.30)	1.23 (1.21 to 1.24)	0.27 (0.06, 0.69)	0.39 (0.10, 1.08)	1.27 (1.25 to 1.3)
**Luxembourg**	971.37 (531.13, 1607.03)	1205.75 (660.07, 1997.36)	0.7 (0.69 to 0.71)	5.97 (2.09, 14.00)	7.37 (2.49, 17.26)	0.68 (0.67 to 0.69)	427.43 (120.73, 918.70)	507.31 (140.30, 1133.30)	0.55 (0.55 to 0.56)	3.19 (0.71, 8.68)	3.79 (0.81, 10.36)	0.56 (0.55 to 0.57)	543.94 (201.36, 1050.21)	698.44 (262.83, 1343.38)	0.83 (0.81 to 0.84)	2.78 (0.69, 7.56)	3.58 (0.91, 9.58)	0.83 (0.81 to 0.85)
**Madagascar**	189.41 (113.41, 304.31)	220.41 (133.79, 362.01)	0.51 (0.5 to 0.53)	1.10 (0.36, 2.68)	1.28 (0.44, 3.00)	0.5 (0.49 to 0.52)	55.84 (25.53, 105.27)	69.19 (17.55, 159.72)	0.75 (0.72 to 0.79)	0.42 (0.10, 1.07)	0.51 (0.08, 1.45)	0.67 (0.63 to 0.71)	133.57 (73.71, 224.80)	151.23 (65.48, 274.78)	0.42 (0.4 to 0.43)	0.68 (0.20, 1.70)	0.77 (0.19, 2.00)	0.42 (0.4 to 0.44)
**Malawi**	257.19 (155.59, 414.25)	255.20 (151.13, 405.14)	0.01 (-0.04 to 0.06)	1.42 (0.50, 3.34)	1.39 (0.47, 3.29)	-0.08 (-0.13 to -0.04)	55.45 (24.47, 102.54)	40.90 (14.88, 84.12)	-0.96 (-1.05 to -0.87)	0.41 (0.10, 1.04)	0.30 (0.05, 0.87)	-0.94 (-1.03 to -0.85)	201.74 (115.66, 335.81)	214.30 (125.16, 345.57)	0.24 (0.13 to 0.31)	1.02 (0.33, 2.47)	1.09 (0.34, 2.62)	0.25 (0.2 to 0.29)

**Malaysia**	874.74 (505.68, 1444.35)	1442.49 (837.70, 2372.88)	1.64 (1.62 to 1.66)	4.88 (1.60, 11.37)	8.00 (2.80, 18.45)	1.61 (1.59 to 1.63)	183.79 (37.78, 489.16)	286.10 (60.38, 746.66)	1.44 (1.42 to 1.46)	1.36 (0.21, 4.34)	2.12 (0.35, 6.58)	1.43 (1.39 to 1.45)	690.95 (352.52, 1196.80)	1156.40 (579.30, 2020.03)	1.7 (1.67 to 1.72)	3.51 (1.06, 8.67)	5.88 (1.84, 14.76)	1.69 (1.66 to 1.71)
**Maldives**	574.40 (324.60, 922.74)	1170.32 (708.97, 1892.22)	2.32 (2.28 to 2.35)	3.37 (1.15, 7.80)	6.87 (2.49, 15.91)	2.33 (2.29 to 2.36)	194.26 (49.78, 431.20)	399.24 (199.26, 735.66)	2.34 (2.31 to 2.37)	1.44 (0.28, 4.00)	2.98 (1.01, 7.33)	2.38 (2.34 to 2.41)	380.14 (168.84, 676.42)	771.09 (446.45, 1276.09)	2.3 (2.25 to 2.35)	1.93 (0.55, 4.87)	3.89 (1.36, 9.35)	2.29 (2.26 to 2.32)
**Mali**	141.57 (85.57, 226.70)	225.04 (137.14, 363.63)	1.52 (1.5 to 1.54)	0.81 (0.27, 1.89)	1.26 (0.46, 3.00)	1.44 (1.42 to 1.47)	37.73 (18.14, 68.75)	50.07 (20.98, 98.41)	0.98 (0.93 to 1.03)	0.28 (0.07, 0.72)	0.37 (0.09, 0.94)	0.92 (0.87 to 0.96)	103.83 (58.75, 170.87)	174.97 (99.89, 286.86)	1.71 (1.68 to 1.74)	0.53 (0.15, 1.32)	0.89 (0.29, 2.23)	1.69 (1.65 to 1.73)
**Malta**	937.89 (520.98, 1536.35)	1229.01 (666.85, 2025.65)	0.88 (0.87 to 0.88)	5.74 (1.99, 13.50)	7.47 (2.50, 17.63)	0.85 (0.84 to 0.86)	400.38 (113.79, 893.61)	500.61 (131.65, 1117.01)	0.72 (0.72 to 0.73)	3.00 (0.67, 8.17)	3.74 (0.79, 10.13)	0.71 (0.7 to 0.72)	537.51 (201.26, 1013.62)	728.39 (273.70, 1379.28)	0.99 (0.98 to 1)	2.75 (0.71, 7.51)	3.73 (0.95, 10.16)	0.99 (0.98 to 1)
**Marshall Islands**	447.96 (257.70, 742.36)	732.33 (417.75, 1225.43)	1.57 (1.55 to 1.6)	2.56 (0.86, 5.98)	4.17 (1.44, 9.73)	1.57 (1.54 to 1.59)	120.11 (24.81, 305.81)	194.56 (41.89, 492.58)	1.54 (1.5 to 1.56)	0.89 (0.13, 2.75)	1.44 (0.24, 4.39)	1.55 (1.53 to 1.56)	327.85 (143.69, 602.52)	537.78 (238.50, 981.57)	1.59 (1.57 to 1.62)	1.67 (0.45, 4.32)	2.73 (0.76, 7.08)	1.59 (1.56 to 1.62)
**Mauritania**	230.57	334.88	1.23	1.32	1.91	1.21	64.68	91.38	1.14	0.48	0.68	1.14	165.90	243.50	1.26	0.84	1.24	1.26

	(134.33, 381.14)	(196.43, 546.60)	(1.2 to 1.26)	(0.46, 3.17)	(0.68, 4.54)	(1.18 to 1.24)	(21.82, 132.92)	(19.98, 220.36)	(1.1 to 1.18)	(0.10, 1.26)	(0.09, 2.00)	(1.1 to 1.18)	(77.56, 297.75)	(106.52, 440.98)	(1.24 to 1.28)	(0.24, 2.22)	(0.34, 3.25)	(1.23 to 1.28)
**Mauritius**	858.40 (501.66, 1457.32)	1329.34 (788.33, 2190.38)	1.44 (1.42 to 1.46)	4.94 (1.68, 11.38)	7.59 (2.64, 17.94)	1.41 (1.39 to 1.43)	242.83 (54.05, 606.57)	350.86 (84.08, 893.27)	1.2 (1.19 to 1.21)	1.81 (0.33, 5.26)	2.61 (0.49, 7.66)	1.19 (1.18 to 1.2)	615.58 (287.51, 1100.43)	978.48 (461.89, 1720.38)	1.53 (1.51 to 1.55)	3.13 (0.95, 7.78)	4.98 (1.49, 12.68)	1.52 (1.5 to 1.54)
**Mexico**	1100.76 (603.31, 1803.23)	1241.70 (740.28, 1982.77)	0.39 (0.33 to 0.45)	6.26 (2.14, 14.83)	6.91 (2.52, 16.10)	0.32 (0.26 to 0.38)	277.05 (65.20, 684.79)	249.30 (48.72, 648.42)	-0.36 (-0.39 to -0.33)	2.06 (0.39, 6.02)	1.85 (0.30, 5.80)	-0.35 (-0.39 to -0.33)	823.71 (349.92, 1503.69)	992.40 (481.66, 1729.52)	0.6 (0.54 to 0.67)	4.20 (1.19, 10.89)	5.05 (1.61, 12.61)	0.6 (0.53 to 0.66)
**Micronesia (Federated States of)**	565.19 (319.92, 941.57)	800.31 (459.32, 1321.56)	1.1 (1.08 to 1.12)	3.23 (1.08, 7.68)	4.57 (1.52, 10.98)	1.1 (1.07 to 1.12)	152.07 (31.75, 387.26)	211.33 (46.11, 540.23)	1.04 (1.02 to 1.06)	1.13 (0.17, 3.42)	1.56 (0.26, 4.76)	1.05 (1.02 to 1.07)	413.12 (181.43, 760.07)	588.98 (261.25, 1078.25)	1.12 (1.1 to 1.14)	2.11 (0.58, 5.42)	3.01 (0.83, 7.92)	1.12 (1.1 to 1.15)
**Monaco**	1061.80 (583.09, 1735.94)	1226.17 (675.89, 2007.14)	0.47 (0.46 to 0.47)	6.47 (2.23, 15.24)	7.46 (2.58, 17.45)	0.45 (0.44 to 0.46)	451.42 (125.92, 985.98)	505.99 (135.04, 1134.40)	0.36 (0.34 to 0.37)	3.36 (0.73, 9.01)	3.78 (0.79, 10.08)	0.37 (0.35 to 0.39)	610.39 (229.69, 1163.46)	720.19 (270.10, 1368.07)	0.54 (0.53 to 0.55)	3.11 (0.79, 8.48)	3.68 (0.91, 9.75)	0.55 (0.53 to 0.58)
**Mongolia**	124.23 (70.22,	177.20 (100.31,	1.18	0.71 (0.22,	1.01 (0.32,	1.17	31.91 (5.76,	46.09 (8.06,	1.22	0.23 (0.02,	0.34 (0.03,	1.22	92.33 (41.73,	131.10 (59.72,	1.14	0.47 (0.11,	0.67 (0.17,	1.16

	203.21)	290.62)	(1.16 to 1.2)	1.75)	2.48)	(1.15 to 1.19)	78.76)	118.25)	(1.2 to 1.25)	0.74)	1.08)	(1.18 to 1.26)	167.41)	235.93)	(1.13 to 1.16)	1.30)	1.78)	(1.14 to 1.17)
**Montenegro**	63.72 (34.36, 110.79)	81.45 (44.16, 138.21)	0.81 (0.79 to 0.83)	0.36 (0.09, 0.92)	0.46 (0.12, 1.16)	0.82 (0.8 to 0.84)	13.90 (2.24, 39.44)	17.92 (2.88, 50.14)	0.83 (0.82 to 0.85)	0.10 (0.01, 0.37)	0.13 (0.01, 0.48)	0.86 (0.83 to 0.9)	49.83 (22.43, 92.71)	63.53 (29.20, 115.41)	0.81 (0.79 to 0.83)	0.25 (0.05, 0.72)	0.32 (0.07, 0.86)	0.81 (0.78 to 0.84)
**Morocco**	584.36 (355.58, 927.62)	819.44 (482.24, 1342.70)	1.11 (1.08 to 1.14)	3.61 (1.31, 8.25)	4.89 (1.75, 11.45)	1.02 (0.99 to 1.04)	272.22 (142.14, 459.53)	303.39 (83.51, 679.32)	0.37 (0.34 to 0.4)	2.03 (0.67, 4.70)	2.26 (0.49, 6.13)	0.37 (0.33 to 0.4)	312.14 (170.20, 532.02)	516.06 (195.73, 984.72)	1.67 (1.62 to 1.71)	1.58 (0.50, 3.89)	2.63 (0.67, 6.91)	1.66 (1.61 to 1.7)
**Mozambique**	187.97 (111.40, 314.69)	287.20 (170.97, 467.80)	1.38 (1.36 to 1.41)	1.05 (0.35, 2.58)	1.61 (0.54, 3.76)	1.42 (1.39 to 1.45)	44.71 (9.77, 111.65)	67.47 (14.76, 168.52)	1.39 (1.33 to 1.44)	0.33 (0.04, 1.01)	0.49 (0.07, 1.52)	1.4 (1.36 to 1.46)	143.26 (67.84, 257.16)	219.73 (103.41, 383.34)	1.38 (1.36 to 1.39)	0.73 (0.19, 1.93)	1.11 (0.30, 2.83)	1.39 (1.36 to 1.41)
**Myanmar**	455.89 (265.71, 760.97)	873.94 (537.30, 1374.63)	2.14 (2.12 to 2.15)	2.65 (0.85, 6.28)	5.08 (1.82, 11.72)	2.13 (2.1 to 2.15)	142.28 (31.10, 350.05)	273.04 (130.30, 492.45)	2.12 (2.1 to 2.15)	1.06 (0.15, 3.14)	2.03 (0.66, 4.92)	2.13 (2.08 to 2.16)	313.61 (144.97, 565.22)	600.90 (359.34, 973.68)	2.15 (2.12 to 2.16)	1.60 (0.44, 4.17)	3.05 (1.04, 7.14)	2.13 (2.11 to 2.15)
**Namibia**	245.15 (146.23, 393.20)	327.61 (188.86, 541.91)	0.96 (0.93	1.39 (0.46, 3.35)	1.87 (0.61, 4.50)	0.99 (0.95	59.90 (24.55, 120.81)	86.15 (15.81, 225.12)	1.15 (1.06	0.45 (0.10, 1.22)	0.64 (0.07, 2.00)	1.14 (1.04	185.26 (105.03, 309.29)	241.46 (104.69, 435.56)	0.88 (0.85	0.94 (0.29, 2.39)	1.23 (0.32, 3.27)	0.88 (0.85

			to 0.98)			to 1.02)			to 1.24)			to 1.24)			to 0.9)			to 0.9)
**Nauru**	653.06 (371.07, 1096.32)	923.50 (544.61, 1542.63)	1.11 (1.08 to 1.13)	3.71 (1.23, 8.78)	5.26 (1.83, 12.59)	1.11 (1.09 to 1.13)	169.28 (36.30, 437.98)	237.90 (51.76, 613.88)	1.08 (1.05 to 1.11)	1.25 (0.19, 3.88)	1.77 (0.30, 5.43)	1.1 (1.08 to 1.13)	483.77 (215.56, 889.24)	685.60 (311.41, 1249.27)	1.11 (1.09 to 1.13)	2.46 (0.70, 6.38)	3.49 (1.02, 8.98)	1.12 (1.1 to 1.13)
**Nepal**	151.23 (89.81, 247.91)	229.63 (137.98, 362.93)	1.35 (1.32 to 1.38)	0.89 (0.30, 2.15)	1.40 (0.47, 3.33)	1.48 (1.43 to 1.53)	52.93 (12.77, 121.24)	96.99 (46.68, 169.45)	1.99 (1.94 to 2.05)	0.39 (0.05, 1.17)	0.73 (0.21, 1.82)	2.01 (1.93 to 2.09)	98.30 (38.35, 181.39)	132.64 (71.51, 227.11)	1 (0.94 to 1.05)	0.50 (0.11, 1.34)	0.67 (0.19, 1.72)	0.99 (0.93 to 1.05)
**Netherlands**	879.67 (496.72, 1446.16)	1087.40 (601.33, 1784.30)	0.68 (0.68 to 0.69)	5.37 (1.85, 12.48)	6.62 (2.25, 15.51)	0.67 (0.66 to 0.68)	377.98 (109.59, 813.99)	450.55 (124.17, 1003.81)	0.55 (0.53 to 0.57)	2.82 (0.65, 7.58)	3.36 (0.75, 9.22)	0.55 (0.54 to 0.57)	501.70 (184.76, 959.36)	636.85 (244.69, 1208.22)	0.77 (0.75 to 0.78)	2.55 (0.65, 6.96)	3.25 (0.80, 8.78)	0.79 (0.78 to 0.8)
**New Zealand**	1199.45 (445.67, 2386.68)	1469.73 (594.96, 2692.00)	0.66 (0.6 to 0.72)	7.08 (1.82, 18.40)	8.60 (2.39, 20.54)	0.62 (0.56 to 0.67)	400.29 (108.54, 1014.07)	463.11 (122.72, 1104.75)	0.46 (0.44 to 0.49)	2.98 (0.61, 8.93)	3.45 (0.74, 9.64)	0.47 (0.44 to 0.49)	799.16 (210.08, 1835.76)	1006.62 (298.78, 2088.90)	0.73 (0.66 to 0.8)	4.10 (0.78, 12.73)	5.14 (1.07, 14.29)	0.71 (0.64 to 0.77)
**Nicaragua**	542.51 (307.25, 902.77)	754.45 (434.08, 1233.31)	1.07 (1.06 to	3.08 (1.07, 7.35)	4.28 (1.49, 10.15)	1.06 (1.05 to	137.90 (27.22, 363.08)	181.33 (37.46, 468.17)	0.9 (0.87 to	1.03 (0.16, 3.18)	1.35 (0.21, 4.15)	0.9 (0.86 to	404.61 (171.82, 736.41)	573.12 (250.70, 1032.01)	1.13 (1.11 to	2.05 (0.58, 5.23)	2.92 (0.82, 7.40)	1.15 (1.14 to

			1.08)			1.08)			0.95)			0.94)			1.14)			1.16)
**Niger**	153.55 (91.87, 244.20)	206.94 (123.32, 345.54)	0.98 (0.96 to 1.01)	0.88 (0.29, 2.12)	1.16 (0.38, 2.88)	0.88 (0.86 to 0.91)	44.94 (21.11, 82.06)	43.14 (9.20, 108.91)	-0.14 (-0.26 to -0.04)	0.34 (0.08, 0.86)	0.32 (0.03, 1.01)	-0.14 (-0.22 to -0.07)	108.61 (60.14, 180.49)	163.80 (78.26, 286.03)	1.33 (1.3 to 1.36)	0.55 (0.16, 1.41)	0.84 (0.23, 2.23)	1.37 (1.33 to 1.4)
**Nigeria**	211.51 (124.90, 345.09)	288.04 (166.90, 468.30)	1.02 (0.98 to 1.06)	1.18 (0.41, 2.73)	1.62 (0.57, 3.73)	1.05 (1.01 to 1.08)	45.73 (10.08, 111.34)	68.16 (12.99, 170.18)	1.36 (1.31 to 1.41)	0.34 (0.06, 0.98)	0.51 (0.08, 1.48)	1.37 (1.32 to 1.43)	165.78 (77.23, 289.54)	219.88 (104.94, 385.40)	0.92 (0.88 to 0.96)	0.84 (0.25, 2.10)	1.12 (0.34, 2.81)	0.92 (0.88 to 0.96)
**Niue**	708.95 (409.56, 1172.90)	1046.85 (609.98, 1734.68)	1.25 (1.23 to 1.26)	4.04 (1.36, 9.67)	5.95 (2.04, 14.08)	1.24 (1.22 to 1.26)	182.41 (39.31, 472.13)	265.38 (57.90, 676.71)	1.2 (1.18 to 1.21)	1.36 (0.22, 4.13)	1.98 (0.35, 5.94)	1.18 (1.16 to 1.2)	526.54 (234.72, 949.10)	781.47 (351.29, 1422.88)	1.26 (1.24 to 1.27)	2.68 (0.76, 6.88)	3.97 (1.16, 10.15)	1.26 (1.24 to 1.27)
**North Macedonia**	53.56 (28.64, 92.74)	74.19 (40.40, 127.35)	1.07 (1.06 to 1.08)	0.30 (0.08, 0.76)	0.42 (0.12, 1.05)	1.09 (1.05 to 1.11)	11.69 (1.88, 33.29)	16.08 (2.53, 45.24)	1.04 (1.03 to 1.06)	0.09 (0.00, 0.30)	0.12 (0.01, 0.42)	1.13 (1.08 to 1.17)	41.87 (18.66, 78.45)	58.11 (27.31, 106.14)	1.08 (1.06 to 1.09)	0.21 (0.04, 0.58)	0.29 (0.07, 0.80)	1.11 (1.08 to 1.14)
**Northern Mariana Islands**	783.67 (456.73, 1284.06)	997.87 (584.39, 1657.06)	0.78 (0.77 to 0.79)	4.47 (1.53, 10.48)	5.68 (1.88, 13.12)	0.77 (0.76 to 0.78)	204.76 (43.57, 523.68)	258.03 (55.93, 668.31)	0.75 (0.74 to 0.76)	1.52 (0.25, 4.57)	1.91 (0.33, 5.92)	0.74 (0.73 to 0.76)	578.91 (256.65, 1060.52)	739.83 (331.38, 1360.91)	0.79 (0.78 to 0.8)	2.95 (0.86, 7.74)	3.76 (1.07, 9.69)	0.78 (0.77 to 0.79)

**Norway**	989.02 (580.39, 1628.49)	1128.40 (654.19, 1858.70)	0.44 (0.41 to 0.46)	6.02 (2.18, 13.95)	6.85 (2.45, 16.02)	0.43 (0.41 to 0.44)	413.11 (133.38, 890.35)	462.31 (146.94, 1006.79)	0.36 (0.35 to 0.37)	3.08 (0.79, 8.38)	3.45 (0.87, 9.41)	0.36 (0.35 to 0.37)	575.91 (230.99, 1102.72)	666.10 (267.40, 1258.08)	0.5 (0.46 to 0.52)	2.94 (0.82, 7.70)	3.40 (0.94, 8.91)	0.49 (0.46 to 0.52)
**Oman**	589.17 (347.68, 965.37)	959.71 (545.82, 1553.59)	1.6 (1.58 to 1.63)	3.44 (1.22, 8.17)	5.59 (1.95, 13.29)	1.6 (1.57 to 1.63)	186.37 (47.93, 425.88)	299.06 (76.47, 688.17)	1.56 (1.53 to 1.59)	1.39 (0.25, 3.89)	2.22 (0.44, 6.35)	1.54 (1.51 to 1.57)	402.80 (164.66, 744.18)	660.65 (268.55, 1206.75)	1.63 (1.62 to 1.66)	2.05 (0.54, 5.40)	3.37 (0.91, 8.81)	1.65 (1.63 to 1.68)
**Pakistan**	257.08 (149.93, 422.09)	292.21 (160.29, 471.70)	0.34 (0.18 to 0.46)	1.53 (0.52, 3.66)	1.79 (0.59, 4.34)	0.45 (0.31 to 0.57)	97.80 (14.16, 248.37)	129.68 (16.71, 320.29)	0.93 (0.76 to 1.06)	0.72 (0.08, 2.17)	0.96 (0.09, 2.85)	0.94 (0.78 to 1.08)	159.28 (42.05, 329.03)	162.53 (30.31, 353.14)	-0.05 (-0.2 to 0.08)	0.81 (0.15, 2.22)	0.83 (0.11, 2.40)	-0.03 (-0.18 to 0.1)
**Palau**	741.29 (419.40, 1221.93)	1045.20 (586.88, 1744.33)	1.08 (1.07 to 1.1)	4.23 (1.43, 10.08)	5.94 (2.05, 14.38)	1.08 (1.06 to 1.1)	190.76 (41.37, 490.26)	265.05 (59.47, 685.98)	1.05 (1.01 to 1.07)	1.42 (0.23, 4.27)	1.97 (0.34, 5.95)	1.04 (1.01 to 1.08)	550.53 (244.02, 999.08)	780.15 (346.41, 1406.73)	1.1 (1.08 to 1.12)	2.81 (0.79, 7.28)	3.97 (1.19, 10.35)	1.09 (1.08 to 1.11)
**Palestine**	617.84 (363.49, 998.44)	782.09 (457.67, 1270.23)	0.76 (0.75 to 0.77)	3.63 (1.28, 8.39)	4.58 (1.60, 10.69)	0.76 (0.74 to 0.77)	201.64 (52.58, 463.09)	248.94 (64.65, 576.31)	0.69 (0.68 to 0.7)	1.50 (0.28, 4.21)	1.85 (0.36, 5.21)	0.68 (0.67 to 0.7)	416.20 (168.24, 776.95)	533.15 (219.82, 979.42)	0.81 (0.8 to 0.82)	2.12 (0.56, 5.44)	2.73 (0.73, 7.13)	0.81 (0.79 to 0.83)
**Panama**	536.16	830.53	1.43	2.98	4.61	1.41	106.62	158.77	1.29	0.79	1.19	1.31	429.54	671.76	1.45	2.19	3.42	1.45

	(304.81, 888.54)	(482.43, 1371.35)	(1.41 to 1.45)	(0.98, 7.17)	(1.57, 10.89)	(1.38 to 1.43)	(17.81, 300.41)	(27.15, 451.60)	(1.27 to 1.31)	(0.09, 2.71)	(0.16, 3.89)	(1.27 to 1.35)	(208.26, 761.03)	(330.03, 1187.67)	(1.43 to 1.48)	(0.67, 5.64)	(1.06, 8.65)	(1.43 to 1.47)
**Papua New Guinea**	404.03 (234.31, 681.50)	582.63 (347.87, 944.66)	1.17 (1.14 to 1.19)	2.28 (0.78, 5.35)	3.26 (1.14, 7.65)	1.14 (1.12 to 1.17)	95.66 (18.98, 249.04)	128.20 (53.63, 249.99)	0.95 (0.93 to 0.97)	0.71 (0.09, 2.29)	0.95 (0.25, 2.47)	0.95 (0.93 to 0.98)	308.37 (135.90, 561.82)	454.43 (262.31, 751.02)	1.22 (1.2 to 1.25)	1.57 (0.44, 4.07)	2.31 (0.79, 5.64)	1.22 (1.19 to 1.25)
**Paraguay**	154.29 (90.03, 257.99)	241.38 (134.33, 399.24)	1.46 (1.44 to 1.48)	0.90 (0.29, 2.23)	1.40 (0.45, 3.43)	1.44 (1.42 to 1.47)	50.03 (18.30, 99.95)	72.71 (14.46, 179.64)	1.22 (1.21 to 1.25)	0.37 (0.07, 1.07)	0.54 (0.06, 1.62)	1.22 (1.19 to 1.25)	104.26 (53.62, 188.65)	168.67 (69.49, 314.45)	1.56 (1.53 to 1.58)	0.53 (0.14, 1.39)	0.86 (0.21, 2.31)	1.59 (1.57 to 1.62)
**Peru**	477.42 (280.30, 756.27)	989.23 (474.71, 1708.63)	2.49 (2.33 to 2.65)	2.70 (0.91, 6.28)	5.45 (1.73, 13.06)	2.37 (2.26 to 2.49)	113.59 (51.33, 216.12)	175.12 (36.45, 445.13)	1.31 (0.83 to 1.6)	0.85 (0.22, 2.18)	1.31 (0.20, 4.11)	1.31 (0.83 to 1.6)	363.83 (197.96, 599.24)	814.11 (309.14, 1517.13)	2.92 (2.66 to 3.22)	1.85 (0.60, 4.47)	4.14 (1.10, 10.77)	2.93 (2.68 to 3.23)
**Philippines**	599.46 (350.38, 1005.71)	991.72 (581.16, 1648.35)	1.69 (1.6 to 1.78)	3.47 (1.16, 8.15)	5.85 (1.99, 13.72)	1.75 (1.67 to 1.83)	181.85 (28.79, 508.86)	339.86 (53.93, 949.67)	2.06 (2.05 to 2.08)	1.35 (0.18, 4.37)	2.52 (0.33, 7.95)	2.06 (2.04 to 2.08)	417.60 (147.47, 784.82)	651.86 (207.97, 1252.56)	1.55 (1.44 to 1.66)	2.13 (0.54, 5.48)	3.33 (0.78, 8.90)	1.56 (1.45 to 1.67)
**Poland**	99.13 (55.37,	104.53 (62.05,	0.15	0.57 (0.20,	0.59 (0.21,	0.07	29.85 (6.00,	26.06 (4.69,	-0.44	0.22 (0.04,	0.19 (0.03,	-0.45	69.28 (29.33,	78.47 (37.27,	0.38	0.35 (0.10,	0.40 (0.12,	0.39

	166.66)	163.05)	(0.11 to 0.17)	1.36)	1.39)	(0.04 to 0.09)	74.16)	65.65)	(-0.48 to - 0.41)	0.65)	0.59)	(-0.48 to - 0.41)	130.32)	135.51)	(0.33 to 0.42)	0.93)	1.03)	(0.33 to 0.42)
**Portugal**	856.54 (478.43, 1381.52)	1118.67 (618.55, 1827.41)	0.87 (0.86 to 0.88)	5.24 (1.82, 12.22)	6.79 (2.35, 15.87)	0.84 (0.83 to 0.86)	368.54 (106.78, 798.46)	469.39 (128.65, 1042.65)	0.78 (0.76 to 0.79)	2.74 (0.63, 7.42)	3.48 (0.80, 9.53)	0.76 (0.75 to 0.78)	488.00 (177.69, 919.12)	649.28 (244.96, 1234.59)	0.93 (0.92 to 0.95)	2.50 (0.62, 6.77)	3.31 (0.86, 8.78)	0.91 (0.89 to 0.93)
**Puerto Rico**	664.59 (373.90, 1100.85)	836.90 (478.11, 1382.05)	0.75 (0.75 to 0.76)	3.89 (1.29, 9.25)	4.88 (1.65, 11.55)	0.74 (0.73 to 0.75)	212.93 (44.94, 513.19)	263.66 (55.10, 645.02)	0.69 (0.69 to 0.7)	1.59 (0.25, 4.67)	1.96 (0.31, 5.76)	0.69 (0.67 to 0.7)	451.67 (203.49, 829.01)	573.25 (258.22, 1055.81)	0.78 (0.77 to 0.79)	2.30 (0.64, 6.04)	2.92 (0.84, 7.67)	0.78 (0.77 to 0.79)
**Qatar**	893.63 (509.38, 1478.91)	1052.45 (588.90, 1729.91)	0.55 (0.53 to 0.56)	5.23 (1.85, 12.29)	6.14 (2.12, 14.48)	0.54 (0.53 to 0.56)	288.54 (75.34, 668.71)	327.77 (84.27, 773.63)	0.44 (0.41 to 0.46)	2.14 (0.42, 6.09)	2.44 (0.48, 6.77)	0.45 (0.43 to 0.47)	605.09 (245.73, 1118.92)	724.68 (294.54, 1331.51)	0.6 (0.59 to 0.61)	3.08 (0.83, 8.13)	3.70 (0.99, 9.72)	0.61 (0.59 to 0.62)
**Republic of Korea**	580.43 (236.65, 1062.66)	831.48 (329.77, 1556.65)	1.2 (1.15 to 1.27)	3.34 (0.86, 8.31)	4.72 (1.26, 12.04)	1.15 (1.1 to 1.2)	161.62 (36.33, 416.03)	211.05 (48.37, 565.44)	0.84 (0.81 to 0.87)	1.20 (0.20, 3.60)	1.57 (0.26, 4.86)	0.83 (0.8 to 0.86)	418.80 (137.74, 846.94)	620.43 (197.58, 1265.60)	1.32 (1.26 to 1.39)	2.14 (0.45, 5.80)	3.15 (0.70, 8.63)	1.31 (1.25 to 1.37)
**Republic of Moldova**	65.82 (37.50, 111.34)	97.44 (55.59, 160.57)	1.28 (1.26	0.39 (0.11, 0.97)	0.58 (0.17, 1.48)	1.28 (1.24	22.77 (4.56, 53.80)	33.57 (6.47, 77.63)	1.26 (1.2 to	0.17 (0.01, 0.52)	0.25 (0.02, 0.76)	1.25 (1.18	43.05 (18.23, 81.45)	63.88 (27.68, 121.18)	1.3 (1.25	0.22 (0.04, 0.62)	0.33 (0.07, 0.92)	1.34 (1.29

			to 1.29)			to 1.31)			1.31)			to 1.31)			to 1.34)			to 1.39)
**Romania**	55.29 (30.01, 96.95)	78.51 (42.73, 134.85)	1.18 (1.16 to 1.2)	0.31 (0.08, 0.81)	0.44 (0.12, 1.14)	1.15 (1.11 to 1.18)	12.36 (2.04, 34.37)	17.22 (2.68, 49.12)	1.1 (1.08 to 1.14)	0.09 (0.00, 0.33)	0.13 (0.01, 0.46)	1.11 (1.06 to 1.16)	42.93 (19.33, 79.74)	61.29 (28.59, 111.86)	1.18 (1.16 to 1.19)	0.22 (0.04, 0.62)	0.31 (0.07, 0.84)	1.18 (1.15 to 1.2)
**Russian Federation**	82.81 (47.15, 137.83)	108.23 (62.33, 177.77)	0.88 (0.86 to 0.89)	0.48 (0.17, 1.13)	0.63 (0.22, 1.47)	0.86 (0.85 to 0.87)	26.51 (5.51, 62.87)	33.53 (6.77, 80.29)	0.77 (0.75 to 0.79)	0.20 (0.03, 0.56)	0.25 (0.04, 0.72)	0.77 (0.75 to 0.79)	56.30 (24.73, 104.75)	74.70 (33.34, 138.79)	0.92 (0.91 to 0.93)	0.29 (0.08, 0.74)	0.38 (0.11, 0.99)	0.93 (0.91 to 0.93)
**Rwanda**	203.06 (117.57, 338.17)	265.17 (141.56, 440.38)	0.81 (0.64 to 0.93)	1.17 (0.37, 2.80)	1.49 (0.47, 3.55)	0.72 (0.54 to 0.83)	57.07 (21.34, 114.15)	61.73 (11.40, 160.75)	0.06 (-0.24 to 0.25)	0.43 (0.08, 1.16)	0.46 (0.05, 1.45)	0.02 (-0.27 to 0.21)	145.99 (76.81, 254.15)	203.45 (84.60, 363.91)	1.02 (0.94 to 1.09)	0.75 (0.22, 1.85)	1.04 (0.26, 2.68)	1.05 (0.97 to 1.11)
**Saint Kitts and Nevis**	513.17 (294.28, 825.75)	686.49 (382.04, 1150.61)	0.95 (0.94 to 0.96)	3.01 (1.01, 7.26)	4.00 (1.34, 9.39)	0.94 (0.92 to 0.95)	170.00 (37.06, 414.62)	220.64 (46.84, 530.50)	0.84 (0.83 to 0.85)	1.26 (0.21, 3.65)	1.64 (0.28, 4.73)	0.85 (0.82 to 0.89)	343.17 (150.05, 630.16)	465.85 (207.35, 862.12)	1 (0.98 to 1.01)	1.75 (0.48, 4.65)	2.37 (0.67, 6.17)	0.99 (0.97 to 1)
**Saint Lucia**	428.83 (244.97, 707.45)	569.84 (323.66, 920.48)	0.93 (0.91 to	2.53 (0.85, 6.00)	3.34 (1.10, 7.97)	0.92 (0.9 to 0.94)	145.14 (31.47, 345.94)	186.54 (39.32, 447.35)	0.82 (0.8 to 0.84)	1.08 (0.17, 3.17)	1.38 (0.21, 4.06)	0.82 (0.79 to	283.69 (122.26, 517.51)	383.30 (169.05, 704.40)	0.99 (0.97 to	1.45 (0.40, 3.80)	1.96 (0.54, 5.16)	0.99 (0.97 to 1)

			0.94)										0.85)			1.01)			
**Saint Vincent and the Grenadines**	393.21 (222.71, 637.94)	582.82 (325.09, 963.71)	1.29 (1.26 to 1.32)	2.32 (0.74, 5.51)	3.41 (1.15, 8.27)	1.28 (1.26 to 1.31)	132.27 (28.93, 311.30)	191.54 (39.87, 462.33)	1.22 (1.19 to 1.24)	0.99 (0.16, 2.87)	1.42 (0.23, 4.16)	1.19 (1.17 to 1.21)	260.94 (112.79, 481.04)	391.29 (172.11, 719.24)	1.34 (1.31 to 1.37)	1.33 (0.35, 3.47)	1.99 (0.55, 5.22)	1.34 (1.3 to 1.38)
**Samoa**	672.35 (380.13, 1141.18)	890.06 (502.65, 1481.87)	0.91 (0.89 to 0.92)	3.83 (1.27, 9.08)	5.06 (1.70, 11.81)	0.91 (0.88 to 0.92)	174.77 (36.64, 449.78)	227.85 (49.67, 584.20)	0.85 (0.82 to 0.88)	1.30 (0.20, 3.89)	1.69 (0.28, 5.03)	0.86 (0.83 to 0.89)	497.58 (219.89, 921.51)	662.21 (290.94, 1206.95)	0.93 (0.91 to 0.94)	2.53 (0.71, 6.69)	3.37 (0.96, 8.50)	0.93 (0.91 to 0.94)
**San Marino**	1022.15 (566.00, 1665.96)	1190.62 (650.14, 1948.21)	0.49 (0.48 to 0.5)	6.24 (2.16, 14.77)	7.25 (2.46, 17.18)	0.48 (0.47 to 0.49)	435.56 (124.40, 944.17)	493.61 (129.25, 1098.38)	0.41 (0.39 to 0.42)	3.25 (0.71, 8.98)	3.68 (0.75, 10.05)	0.4 (0.39 to 0.42)	586.59 (219.25, 1121.84)	697.01 (261.78, 1309.72)	0.57 (0.55 to 0.58)	2.99 (0.76, 7.97)	3.57 (0.92, 9.64)	0.57 (0.56 to 0.59)
**Sao Tome and Principe**	192.10 (96.96, 320.18)	285.37 (150.75, 462.55)	1.28 (1.25 to 1.31)	1.07 (0.32, 2.60)	1.58 (0.49, 3.76)	1.25 (1.22 to 1.28)	38.63 (7.00, 97.28)	56.29 (10.05, 141.49)	1.26 (1.21 to 1.31)	0.29 (0.02, 0.93)	0.41 (0.04, 1.30)	1.23 (1.16 to 1.31)	153.47 (60.47, 270.58)	229.08 (95.02, 400.36)	1.3 (1.26 to 1.33)	0.78 (0.19, 1.97)	1.16 (0.31, 2.94)	1.27 (1.24 to 1.31)
**Saudi Arabia**	740.58 (427.73, 1192.27)	1032.67 (586.90, 1683.07)	1.09 (1.07 to 1.12)	4.32 (1.53, 10.12)	6.00 (2.08, 14.05)	1.07 (1.04 to 1.11)	236.05 (61.27, 548.79)	319.83 (80.97, 742.69)	1.02 (1 to 1.04)	1.75 (0.35, 4.96)	2.37 (0.47, 6.78)	1.02 (0.99 to 1.04)	504.53 (206.63, 935.02)	712.83 (292.81, 1294.50)	1.14 (1.12 to 1.16)	2.57 (0.69, 6.60)	3.63 (0.99, 9.51)	1.13 (1.12 to 1.15)

**Senegal**	217.50 (132.16, 349.98)	272.97 (167.33, 433.69)	0.73 (0.71 to 0.74)	1.25 (0.41, 3.03)	1.55 (0.54, 3.70)	0.69 (0.66 to 0.72)	61.88 (29.17, 112.63)	70.73 (30.78, 133.37)	0.41 (0.36 to 0.45)	0.46 (0.11, 1.17)	0.52 (0.13, 1.37)	0.41 (0.35 to 0.46)	155.61 (87.52, 259.74)	202.25 (113.37, 331.03)	0.84 (0.82 to 0.85)	0.79 (0.24, 2.00)	1.03 (0.32, 2.59)	0.85 (0.82 to 0.87)
**Serbia**	55.57 (30.09, 97.58)	75.03 (41.40, 125.96)	0.98 (0.97 to 0.98)	0.31 (0.08, 0.81)	0.42 (0.11, 1.06)	0.99 (0.96 to 1.01)	11.85 (1.88, 33.12)	16.35 (2.62, 45.44)	1.06 (1.04 to 1.07)	0.09 (0.00, 0.32)	0.12 (0.01, 0.42)	1.09 (1.03 to 1.14)	43.72 (19.78, 81.35)	58.69 (27.14, 105.99)	0.95 (0.95 to 0.96)	0.22 (0.05, 0.62)	0.30 (0.06, 0.80)	0.98 (0.95 to 1.02)
**Seychelles**	910.80 (531.45, 1511.76)	1245.99 (746.58, 2050.01)	1.02 (1.01 to 1.04)	5.25 (1.74, 12.32)	7.15 (2.51, 16.58)	1.02 (1 to 1.03)	256.74 (57.84, 646.11)	340.28 (83.11, 846.15)	0.93 (0.9 to 0.95)	1.91 (0.34, 5.89)	2.53 (0.49, 7.36)	0.93 (0.9 to 0.95)	654.06 (307.55, 1161.36)	905.71 (427.00, 1586.64)	1.06 (1.05 to 1.08)	3.34 (0.99, 8.42)	4.62 (1.40, 11.74)	1.07 (1.06 to 1.09)
**Sierra Leone**	167.46 (98.77, 274.93)	286.74 (168.27, 472.06)	1.79 (1.73 to 1.85)	0.92 (0.29, 2.21)	1.57 (0.52, 3.78)	1.76 (1.71 to 1.8)	28.45 (5.09, 76.09)	46.70 (8.24, 126.03)	1.55 (1.38 to 1.67)	0.21 (0.02, 0.68)	0.34 (0.03, 1.13)	1.52 (1.32 to 1.66)	139.01 (70.09, 240.93)	240.04 (121.07, 411.90)	1.8 (1.77 to 1.83)	0.71 (0.19, 1.82)	1.22 (0.36, 3.12)	1.82 (1.79 to 1.85)
**Singapore**	643.86 (255.39, 1205.80)	963.55 (381.63, 1806.39)	1.32 (1.31 to 1.34)	3.69 (0.96, 9.21)	5.44 (1.49, 13.73)	1.28 (1.26 to 1.29)	175.63 (39.83, 461.06)	227.17 (50.94, 615.80)	0.83 (0.81 to 0.84)	1.30 (0.21, 3.98)	1.68 (0.26, 5.52)	0.82 (0.8 to 0.84)	468.23 (146.91, 953.78)	736.38 (246.93, 1481.18)	1.5 (1.48 to 1.52)	2.38 (0.53, 6.41)	3.76 (0.86, 10.46)	1.5 (1.48 to 1.52)
**Slovakia**	57.32	79.30	1.05	0.32	0.44	1.05	12.19	17.35	1.15	0.09	0.13	1.22	45.13	61.95	1.02	0.23	0.31	1

	(30.67, 97.60)	(43.63, 132.96)	(1.03 to 1.06)	(0.09, 0.85)	(0.13, 1.11)	(1.02 to 1.07)	(1.93, 34.97)	(2.74, 50.14)	(1.14 to 1.17)	(0.00, 0.33)	(0.01, 0.45)	(1.15 to 1.29)	(20.46, 81.77)	(28.67, 112.03)	(1.01 to 1.03)	(0.05, 0.64)	(0.07, 0.84)	(0.96 to 1.04)
**Slovenia**	56.56 (32.14, 98.34)	81.06 (44.21, 140.62)	1.18 (1.17 to 1.19)	0.31 (0.09, 0.79)	0.43 (0.12, 1.08)	1.12 (1.09 to 1.15)	9.38 (2.72, 22.73)	10.90 (1.60, 36.34)	0.5 (0.46 to 0.53)	0.07 (0.01, 0.23)	0.08 (0.00, 0.32)	0.5 (0.39 to 0.55)	47.19 (24.59, 84.49)	70.16 (33.88, 125.47)	1.31 (1.29 to 1.32)	0.24 (0.06, 0.60)	0.35 (0.09, 0.90)	1.3 (1.27 to 1.33)
**Solomon Islands**	442.28 (256.42, 730.72)	679.10 (388.39, 1130.19)	1.37 (1.35 to 1.39)	2.52 (0.82, 6.01)	3.88 (1.32, 9.15)	1.38 (1.36 to 1.4)	120.38 (24.75, 307.23)	182.85 (37.92, 459.40)	1.34 (1.32 to 1.36)	0.89 (0.12, 2.74)	1.35 (0.21, 4.07)	1.37 (1.35 to 1.39)	321.90 (142.74, 588.62)	496.25 (221.09, 911.47)	1.38 (1.37 to 1.41)	1.63 (0.46, 4.23)	2.53 (0.71, 6.59)	1.39 (1.37 to 1.42)
**Somalia**	188.43 (114.46, 310.95)	221.04 (133.77, 369.19)	0.5 (0.49 to 0.51)	1.07 (0.35, 2.52)	1.25 (0.42, 2.94)	0.53 (0.5 to 0.55)	46.77 (10.28, 112.22)	54.50 (12.05, 134.31)	0.5 (0.49 to 0.5)	0.35 (0.04, 1.05)	0.41 (0.05, 1.25)	0.49 (0.44 to 0.53)	141.66 (68.12, 251.11)	166.54 (79.56, 298.08)	0.5 (0.49 to 0.52)	0.72 (0.20, 1.85)	0.85 (0.23, 2.15)	0.53 (0.5 to 0.55)
**South Africa**	353.84 (203.91, 572.58)	427.98 (246.63, 713.24)	0.65 (0.63 to 0.68)	2.05 (0.71, 4.72)	2.47 (0.84, 5.83)	0.65 (0.61 to 0.68)	106.24 (22.22, 254.44)	126.94 (25.88, 324.29)	0.62 (0.58 to 0.66)	0.79 (0.14, 2.25)	0.94 (0.16, 2.81)	0.61 (0.58 to 0.64)	247.61 (110.37, 447.02)	301.04 (128.40, 547.77)	0.67 (0.63 to 0.71)	1.26 (0.37, 3.21)	1.53 (0.43, 4.03)	0.66 (0.62 to 0.7)
**South Sudan**	207.85 (125.16,	232.44 (140.23,	0.37	1.18 (0.40,	1.32 (0.45,	0.38	52.78 (11.71,	58.17 (12.84,	0.34	0.39 (0.05,	0.43 (0.05,	0.33	155.08 (73.72,	174.27 (83.13,	0.39	0.79 (0.21,	0.89 (0.24,	0.4

	337.75)	379.30)	(0.36 to 0.39)	2.80)	3.23)	(0.37 to 0.4)	128.40)	143.53)	(0.31 to 0.36)	1.16)	1.36)	(0.29 to 0.37)	274.45)	306.88)	(0.37 to 0.4)	2.02)	2.40)	(0.37 to 0.42)
**Spain**	746.88 (418.94, 1251.95)	1012.29 (504.67, 1695.06)	1 (0.97 to 1.02)	4.60 (1.54, 10.94)	6.06 (1.92, 14.57)	0.9 (0.88 to 0.92)	329.95 (133.84, 663.23)	375.04 (92.62, 872.01)	0.42 (0.39 to 0.44)	2.48 (0.69, 6.42)	2.80 (0.54, 7.81)	0.38 (0.36 to 0.41)	416.93 (198.45, 767.62)	637.25 (244.29, 1210.86)	1.38 (1.34 to 1.41)	2.11 (0.62, 5.32)	3.26 (0.83, 8.84)	1.4 (1.36 to 1.44)
**Sri Lanka**	714.32 (419.62, 1162.55)	1118.99 (658.95, 1840.63)	1.48 (1.45 to 1.52)	4.27 (1.47, 10.01)	6.43 (2.25, 15.06)	1.35 (1.32 to 1.39)	271.98 (119.95, 505.15)	314.15 (77.93, 771.87)	0.48 (0.44 to 0.52)	2.03 (0.62, 4.93)	2.32 (0.44, 6.85)	0.46 (0.42 to 0.51)	442.33 (240.43, 747.44)	804.84 (375.23, 1418.31)	1.98 (1.96 to 2.01)	2.24 (0.70, 5.52)	4.11 (1.21, 10.24)	2 (1.97 to 2.04)
**Sudan**	423.60 (256.03, 679.99)	754.69 (442.98, 1193.78)	1.9 (1.87 to 1.92)	2.41 (0.85, 5.71)	4.22 (1.46, 9.71)	1.82 (1.79 to 1.84)	114.66 (53.01, 209.64)	162.73 (33.57, 420.10)	1.13 (1.08 to 1.17)	0.85 (0.24, 2.10)	1.20 (0.18, 3.63)	1.11 (1.05 to 1.16)	308.94 (170.62, 514.13)	591.96 (278.53, 1015.41)	2.13 (2.1 to 2.16)	1.56 (0.50, 3.92)	3.01 (0.91, 7.43)	2.16 (2.13 to 2.19)
**Suriname**	447.94 (255.64, 730.94)	602.46 (337.00, 999.58)	0.97 (0.95 to 0.98)	2.63 (0.86, 6.28)	3.51 (1.19, 8.35)	0.95 (0.93 to 0.96)	148.04 (31.66, 350.81)	196.79 (41.88, 470.33)	0.91 (0.9 to 0.93)	1.11 (0.16, 3.25)	1.46 (0.22, 4.26)	0.89 (0.87 to 0.92)	299.90 (132.31, 551.61)	405.67 (179.09, 740.85)	0.98 (0.97 to 1)	1.53 (0.41, 3.97)	2.06 (0.58, 5.36)	0.98 (0.96 to 1)
**Sweden**	796.81 (462.05, 1317.99)	946.80 (550.85, 1557.39)	0.56 (0.53	4.84 (1.68, 11.46)	5.71 (2.03, 13.36)	0.54 (0.52	327.30 (103.34, 724.66)	375.24 (120.25, 827.29)	0.44 (0.42	2.45 (0.61, 6.69)	2.79 (0.71, 7.53)	0.43 (0.4 to	469.51 (185.22, 889.09)	571.57 (226.50, 1086.85)	0.64 (0.62	2.39 (0.64, 6.36)	2.91 (0.80, 7.59)	0.64 (0.62

			to 0.58)			to 0.55)			to 0.46)			0.45)			to 0.66)			to 0.66)
**Switzerland**	951.86 (519.33, 1552.05)	1067.50 (590.40, 1750.94)	0.37 (0.36 to 0.38)	5.83 (2.04, 13.79)	6.49 (2.23, 15.53)	0.35 (0.34 to 0.36)	411.63 (116.36, 906.49)	443.58 (119.09, 982.95)	0.23 (0.21 to 0.25)	3.07 (0.69, 8.24)	3.31 (0.73, 9.01)	0.24 (0.22 to 0.26)	540.23 (200.50, 1036.99)	623.92 (236.75, 1188.55)	0.47 (0.46 to 0.48)	2.76 (0.68, 7.47)	3.19 (0.81, 8.71)	0.47 (0.45 to 0.49)
**Syrian Arab Republic**	600.63 (352.64, 958.82)	810.14 (471.52, 1327.94)	0.99 (0.96 to 1.03)	3.48 (1.24, 8.16)	4.68 (1.63, 10.77)	0.98 (0.95 to 1.01)	179.17 (65.47, 363.04)	238.66 (59.83, 563.73)	0.95 (0.93 to 0.98)	1.33 (0.35, 3.37)	1.77 (0.35, 4.86)	0.94 (0.91 to 0.97)	421.47 (210.72, 749.45)	571.48 (237.83, 1049.43)	1.04 (1 to 1.07)	2.15 (0.66, 5.45)	2.92 (0.82, 7.53)	1.03 (1 to 1.05)
**Taiwan (Province of China)**	575.70 (326.84, 958.32)	903.13 (534.65, 1478.29)	1.45 (1.44 to 1.46)	3.16 (1.03, 7.71)	4.92 (1.72, 11.95)	1.44 (1.42 to 1.46)	93.03 (14.93, 267.65)	136.77 (24.30, 383.75)	1.24 (1.22 to 1.25)	0.69 (0.07, 2.30)	1.02 (0.12, 3.39)	1.26 (1.24 to 1.28)	482.66 (245.39, 849.02)	766.36 (401.91, 1300.98)	1.49 (1.48 to 1.51)	2.47 (0.75, 6.32)	3.90 (1.26, 9.65)	1.49 (1.48 to 1.51)
**Tajikistan**	103.74 (58.11, 173.51)	130.05 (77.61, 210.10)	0.74 (0.7 to 0.77)	0.60 (0.18, 1.47)	0.76 (0.25, 1.95)	0.78 (0.71 to 0.82)	29.35 (5.82, 72.37)	42.83 (16.74, 84.44)	1.22 (1.17 to 1.26)	0.22 (0.02, 0.66)	0.32 (0.05, 0.92)	1.2 (1.15 to 1.25)	74.38 (31.84, 136.70)	87.21 (45.59, 151.27)	0.54 (0.51 to 0.56)	0.38 (0.09, 1.01)	0.44 (0.12, 1.15)	0.5 (0.46 to 0.53)
**Thailand**	669.29 (398.03, 1090.68)	1234.34 (733.87, 2046.66)	2.02 (2 to 2.04)	4.14 (1.47, 9.45)	7.29 (2.59, 17.16)	1.87 (1.85 to	309.34 (147.06, 564.23)	423.73 (116.99, 988.14)	1.03 (0.98 to	2.31 (0.72, 5.54)	3.16 (0.67, 8.93)	1.04 (1 to 1.09)	359.95 (190.84, 628.39)	810.61 (354.40, 1445.43)	2.68 (2.64 to	1.83 (0.57, 4.50)	4.13 (1.22, 10.65)	2.68 (2.65 to

						1.89)			1.07)						2.71)			2.71)
**Timor-Leste**	469.13 (275.90, 789.97)	752.70 (437.97, 1280.82)	1.55 (1.54 to 1.57)	2.74 (0.89, 6.46)	4.39 (1.46, 10.35)	1.55 (1.53 to 1.56)	153.63 (36.77, 359.63)	237.19 (57.77, 567.31)	1.43 (1.41 to 1.44)	1.14 (0.20, 3.16)	1.76 (0.34, 5.11)	1.42 (1.4 to 1.44)	315.50 (143.12, 575.83)	515.51 (233.26, 927.74)	1.61 (1.59 to 1.63)	1.60 (0.44, 4.11)	2.62 (0.74, 6.65)	1.63 (1.61 to 1.65)
**Togo**	166.22 (99.34, 271.31)	263.75 (156.96, 432.22)	1.52 (1.46 to 1.58)	0.95 (0.31, 2.27)	1.49 (0.49, 3.49)	1.48 (1.43 to 1.54)	43.80 (15.98, 90.15)	61.70 (12.99, 151.32)	1.22 (1.12 to 1.33)	0.33 (0.06, 0.91)	0.46 (0.06, 1.40)	1.22 (1.09 to 1.34)	122.42 (63.87, 210.49)	202.05 (92.81, 359.82)	1.63 (1.59 to 1.67)	0.62 (0.18, 1.58)	1.03 (0.28, 2.59)	1.65 (1.6 to 1.7)
**Tokelau**	598.76 (345.54, 988.44)	951.88 (550.54, 1592.90)	1.51 (1.49 to 1.52)	3.41 (1.15, 8.16)	5.41 (1.85, 12.66)	1.48 (1.46 to 1.5)	156.98 (32.86, 406.71)	244.97 (53.78, 632.34)	1.44 (1.42 to 1.46)	1.16 (0.18, 3.46)	1.83 (0.30, 5.53)	1.47 (1.45 to 1.49)	441.78 (196.94, 796.96)	706.91 (317.81, 1285.19)	1.52 (1.5 to 1.54)	2.25 (0.64, 5.89)	3.59 (1.02, 9.09)	1.51 (1.5 to 1.53)
**Tonga**	755.72 (427.40, 1281.43)	1012.06 (580.03, 1681.18)	0.91 (0.89 to 0.93)	4.30 (1.41, 10.24)	5.75 (1.98, 13.55)	0.91 (0.89 to 0.92)	194.15 (40.85, 499.97)	258.34 (57.11, 669.94)	0.91 (0.88 to 0.93)	1.44 (0.24, 4.34)	1.91 (0.32, 5.76)	0.9 (0.87 to 0.91)	561.56 (250.61, 1026.68)	753.72 (340.65, 1381.43)	0.94 (0.91 to 0.97)	2.86 (0.81, 7.19)	3.84 (1.10, 9.70)	0.94 (0.93 to 0.97)
**Trinidad and Tobago**	446.16 (260.98, 724.84)	653.27 (375.60, 1061.37)	1.27 (1.24 to 1.31)	2.72 (0.94, 6.36)	3.85 (1.28, 9.23)	1.16 (1.13 to 1.18)	183.92 (80.50, 347.06)	219.71 (49.72, 520.45)	0.6 (0.57 to 0.64)	1.38 (0.39, 3.55)	1.64 (0.27, 4.88)	0.57 (0.54 to 0.62)	262.24 (135.61, 467.14)	433.56 (186.52, 800.67)	1.64 (1.61 to 1.68)	1.33 (0.41, 3.28)	2.21 (0.62, 5.87)	1.64 (1.59 to 1.68)

**Tunisia**	595.61 (360.03, 975.98)	835.41 (490.62, 1352.01)	1.11 (1.09 to 1.15)	3.68 (1.32, 8.65)	4.95 (1.74, 11.46)	0.98 (0.96 to 1.01)	275.17 (136.71, 495.26)	301.36 (85.06, 665.59)	0.35 (0.31 to 0.38)	2.06 (0.67, 5.04)	2.24 (0.48, 6.09)	0.3 (0.26 to 0.35)	320.44 (160.37, 579.50)	534.06 (206.80, 997.08)	1.71 (1.67 to 1.75)	1.63 (0.49, 4.09)	2.71 (0.70, 7.17)	1.7 (1.67 to 1.75)
**Turkey**	555.04 (336.55, 872.78)	772.95 (452.66, 1239.29)	1.08 (0.97 to 1.19)	3.48 (1.27, 8.01)	4.52 (1.61, 10.48)	0.89 (0.8 to 0.99)	276.89 (140.13, 477.71)	246.14 (60.40, 576.60)	-0.35 (-0.4 to - 0.3)	2.07 (0.69, 4.85)	1.84 (0.36, 5.20)	-0.36 (-0.42 to - 0.3)	278.14 (138.44, 485.59)	526.82 (216.63, 959.64)	2.21 (1.98 to 2.38)	1.41 (0.40, 3.45)	2.68 (0.70, 6.96)	2.22 (1.96 to 2.39)
**Turkmenistan**	132.11 (74.45, 219.06)	192.60 (109.63, 317.76)	1.24 (1.21 to 1.26)	0.75 (0.23, 1.90)	1.09 (0.34, 2.66)	1.2 (1.17 to 1.23)	34.51 (6.16, 87.27)	48.31 (8.42, 124.55)	1.12 (1.09 to 1.16)	0.26 (0.02, 0.80)	0.35 (0.03, 1.12)	1.11 (1.04 to 1.17)	97.60 (44.03, 176.40)	144.28 (65.99, 262.72)	1.28 (1.26 to 1.3)	0.50 (0.12, 1.40)	0.73 (0.18, 1.89)	1.27 (1.24 to 1.3)
**Tuvalu**	562.97 (323.26, 929.61)	866.37 (506.37, 1429.03)	1.37 (1.35 to 1.4)	3.22 (1.10, 7.71)	4.93 (1.69, 11.73)	1.36 (1.34 to 1.39)	148.65 (32.03, 377.22)	224.23 (48.66, 580.46)	1.3 (1.27 to 1.34)	1.10 (0.17, 3.36)	1.66 (0.28, 5.01)	1.29 (1.26 to 1.31)	414.33 (182.18, 759.02)	642.14 (284.94, 1167.16)	1.4 (1.38 to 1.43)	2.11 (0.60, 5.47)	3.27 (0.97, 8.43)	1.39 (1.37 to 1.42)
**Uganda**	212.99 (128.54, 339.00)	255.66 (154.41, 402.13)	0.61 (0.59 to 0.63)	1.21 (0.41, 2.89)	1.41 (0.48, 3.39)	0.5 (0.47 to 0.52)	57.24 (28.00, 102.67)	48.54 (19.07, 95.79)	-0.53 (-0.57 to - 0.5)	0.43 (0.10, 1.07)	0.36 (0.07, 0.97)	-0.57 (-0.63 to - 0.51)	155.74 (88.72, 255.27)	207.11 (119.18, 337.29)	0.96 (0.94 to 0.98)	0.79 (0.25, 1.95)	1.05 (0.34, 2.55)	0.96 (0.93 to 0.98)
**Ukraine**	77.62	93.39	0.63	0.47	0.56	0.59	30.47	34.77	0.46	0.23	0.26	0.47	47.15	58.62	0.73	0.24	0.30	0.7

	(44.57, 128.42)	(54.01, 153.46)	(0.6 to 0.67)	(0.13, 1.14)	(0.17, 1.37)	(0.56 to 0.62)	(7.43, 68.36)	(8.07, 79.12)	(0.41 to 0.52)	(0.03, 0.68)	(0.03, 0.79)	(0.41 to 0.52)	(18.90, 90.98)	(24.27, 112.01)	(0.7 to 0.76)	(0.05, 0.69)	(0.06, 0.81)	(0.66 to 0.74)
**United Arab Emirates**	743.19 (430.47, 1207.19)	968.74 (555.93, 1580.19)	0.87 (0.86 to 0.88)	4.33 (1.52, 10.08)	5.63 (1.98, 13.00)	0.88 (0.86 to 0.89)	233.63 (61.38, 537.16)	301.48 (78.47, 697.40)	0.85 (0.83 to 0.87)	1.74 (0.32, 4.81)	2.25 (0.43, 6.29)	0.85 (0.83 to 0.87)	509.56 (209.35, 935.62)	667.26 (274.28, 1220.76)	0.89 (0.88 to 0.9)	2.60 (0.69, 6.60)	3.39 (0.93, 8.70)	0.89 (0.88 to 0.91)
**United Kingdom**	1067.10 (621.56, 1764.92)	1286.11 (726.49, 2108.20)	0.6 (0.59 to 0.61)	6.51 (2.35, 15.25)	7.82 (2.73, 18.33)	0.59 (0.58 to 0.6)	452.59 (145.89, 968.07)	531.83 (165.45, 1157.67)	0.52 (0.51 to 0.53)	3.38 (0.86, 9.13)	3.97 (0.96, 10.77)	0.52 (0.51 to 0.54)	614.52 (243.08, 1166.06)	754.28 (293.16, 1419.38)	0.67 (0.66 to 0.68)	3.14 (0.86, 8.30)	3.85 (1.05, 10.04)	0.67 (0.66 to 0.68)
**United Republic of Tanzania**	254.36 (150.52, 414.55)	301.43 (184.32, 482.30)	0.56 (0.55 to 0.57)	1.41 (0.48, 3.32)	1.65 (0.55, 3.90)	0.52 (0.5 to 0.55)	52.31 (23.48, 98.11)	50.99 (20.30, 102.92)	-0.07 (-0.12 to -0.02)	0.39 (0.09, 0.99)	0.38 (0.07, 1.03)	-0.06 (-0.11 to -0.01)	202.05 (113.61, 340.71)	250.44 (145.83, 409.50)	0.69 (0.68 to 0.7)	1.02 (0.32, 2.47)	1.27 (0.41, 3.00)	0.74 (0.71 to 0.76)
**United States of America**	984.03 (525.02, 1639.02)	1311.21 (768.76, 2041.98)	0.94 (0.88 to 1)	6.03 (2.04, 13.50)	7.90 (2.82, 17.54)	0.87 (0.81 to 0.93)	430.17 (120.31, 952.09)	517.37 (147.39, 1134.40)	0.59 (0.52 to 0.64)	3.20 (0.73, 8.41)	3.85 (0.89, 10.23)	0.59 (0.52 to 0.64)	553.86 (167.06, 1137.72)	793.84 (287.57, 1500.92)	1.17 (1.11 to 1.22)	2.83 (0.65, 7.82)	4.04 (1.04, 10.49)	1.16 (1.1 to 1.21)
**United States Virgin Islands**	643.77 (359.87,	803.41 (465.53,	0.72	3.79 (1.26,	4.69 (1.58,	0.69	216.45 (46.56,	261.13 (55.74,	0.6	1.61 (0.26,	1.94 (0.33,	0.6	427.32 (187.12,	542.27 (240.30,	0.78	2.18 (0.57,	2.75 (0.78,	0.75

	1081.29)	1334.35)	(0.71 to 0.72)	9.13)	10.98)	(0.68 to 0.7)	522.33)	638.30)	(0.59 to 0.61)	4.74)	5.68)	(0.58 to 0.62)	798.58)	1013.18)	(0.76 to 0.79)	5.78)	7.20)	(0.74 to 0.77)
**Uruguay**	427.44 (247.51, 719.62)	688.14 (396.22, 1158.61)	1.58 (1.55 to 1.6)	2.52 (0.86, 5.85)	4.06 (1.38, 9.29)	1.57 (1.54 to 1.59)	152.03 (41.16, 351.74)	237.75 (66.20, 550.41)	1.47 (1.45 to 1.48)	1.12 (0.24, 3.20)	1.76 (0.37, 4.78)	1.48 (1.46 to 1.5)	275.40 (107.09, 527.22)	450.38 (179.19, 848.42)	1.64 (1.61 to 1.67)	1.40 (0.35, 3.69)	2.30 (0.60, 5.97)	1.63 (1.6 to 1.67)
**Uzbekistan**	134.21 (73.74, 221.20)	187.82 (105.65, 304.41)	1.09 (1.07 to 1.11)	0.76 (0.23, 1.85)	1.06 (0.33, 2.55)	1.08 (1.04 to 1.13)	34.58 (6.62, 87.24)	45.95 (8.54, 118.28)	0.9 (0.83 to 0.98)	0.26 (0.02, 0.84)	0.34 (0.03, 1.06)	0.97 (0.91 to 1.05)	99.64 (43.90, 180.88)	141.87 (65.17, 252.15)	1.14 (1.12 to 1.16)	0.51 (0.12, 1.39)	0.72 (0.19, 1.84)	1.16 (1.1 to 1.19)
**Vanuatu**	510.18 (288.22, 849.55)	742.42 (423.83, 1243.40)	1.2 (1.18 to 1.23)	2.91 (0.95, 6.80)	4.24 (1.44, 10.10)	1.21 (1.18 to 1.23)	136.09 (28.04, 344.67)	198.49 (42.40, 505.25)	1.21 (1.19 to 1.23)	1.01 (0.15, 3.05)	1.47 (0.24, 4.51)	1.21 (1.19 to 1.23)	374.08 (165.46, 689.34)	543.92 (240.58, 1002.13)	1.2 (1.18 to 1.23)	1.90 (0.53, 4.87)	2.77 (0.78, 7.29)	1.21 (1.18 to 1.24)
**Venezuela (Bolivarian Republic of)**	708.18 (399.34, 1162.02)	880.53 (518.20, 1463.54)	0.7 (0.68 to 0.71)	4.03 (1.38, 9.76)	4.98 (1.74, 11.63)	0.68 (0.66 to 0.7)	181.33 (34.63, 476.24)	218.36 (43.29, 574.86)	0.59 (0.58 to 0.61)	1.35 (0.20, 4.21)	1.62 (0.24, 4.89)	0.58 (0.55 to 0.61)	526.85 (241.38, 953.60)	662.16 (305.25, 1206.12)	0.74 (0.72 to 0.75)	2.69 (0.77, 6.91)	3.37 (1.00, 8.48)	0.73 (0.71 to 0.75)
**Viet Nam**	412.29 (241.74, 676.79)	776.30 (448.19, 1283.81)	2.09 (2.06	2.35 (0.77, 5.43)	4.43 (1.47, 10.41)	2.09 (2.06	115.00 (24.81, 289.62)	200.36 (48.01, 487.79)	1.83 (1.8 to	0.85 (0.12, 2.63)	1.50 (0.26, 4.47)	1.82 (1.78	297.29 (140.43, 522.47)	575.95 (270.30, 1012.70)	2.19 (2.15	1.50 (0.43, 3.76)	2.94 (0.87, 7.49)	2.22 (2.18

			to 2.11)			to 2.1)			1.88)			to 1.86)			to 2.23)			to 2.26)
**Yemen**	431.23 (260.66, 707.02)	537.12 (319.60, 875.00)	0.72 (0.7 to 0.74)	2.54 (0.92, 5.96)	3.15 (1.11, 7.32)	0.71 (0.68 to 0.72)	154.68 (41.64, 339.94)	176.72 (44.92, 408.02)	0.45 (0.43 to 0.47)	1.14 (0.21, 3.18)	1.31 (0.23, 3.67)	0.48 (0.45 to 0.51)	276.55 (107.66, 515.82)	360.40 (144.47, 655.82)	0.88 (0.85 to 0.91)	1.41 (0.36, 3.74)	1.84 (0.46, 4.82)	0.9 (0.86 to 0.93)
**Zambia**	287.45 (169.99, 468.21)	346.18 (197.28, 558.41)	0.61 (0.56 to 0.65)	1.58 (0.53, 3.83)	1.92 (0.65, 4.56)	0.7 (0.63 to 0.77)	51.82 (21.83, 101.25)	72.09 (14.00, 188.22)	1.36 (0.94 to 1.78)	0.39 (0.09, 1.04)	0.53 (0.06, 1.64)	1.22 (0.81 to 1.59)	235.63 (134.13, 394.68)	274.10 (130.78, 479.99)	0.46 (0.42 to 0.51)	1.20 (0.37, 2.92)	1.40 (0.40, 3.61)	0.48 (0.42 to 0.54)
**Zimbabwe**	287.08 (172.03, 462.46)	311.43 (179.05, 518.02)	0.23 (0.2 to 0.26)	1.60 (0.53, 3.78)	1.73 (0.56, 4.05)	0.24 (0.19 to 0.28)	58.85 (21.50, 119.61)	63.27 (11.09, 172.74)	0.25 (0.14 to 0.34)	0.44 (0.09, 1.17)	0.47 (0.05, 1.54)	0.21 (0.1 to 0.31)	228.22 (131.52, 378.90)	248.16 (119.31, 436.71)	0.24 (0.16 to 0.3)	1.16 (0.36, 2.83)	1.26 (0.35, 3.24)	0.23 (0.17 to 0.29)

Table S5. The results of age effect.

Age	Type	Location	Rate	CILo	CIHi
15 to 19	Infertility	Global	222.2684074	217.867	226.7588
20 to 24	Infertility	Global	558.1505411	550.0662	566.3537
25 to 29	Infertility	Global	639.8991711	631.2479	648.669
30 to 34	Infertility	Global	690.7246307	681.5012	700.0729
35 to 39	Infertility	Global	747.8290954	737.7094	758.0876
40 to 44	Infertility	Global	773.7393607	762.6855	784.9534
45 to 49	Infertility	Global	287.682142	281.1034	294.4148
15 to 19	Infertility	High-middle SDI	207.8330433	197.2102	219.0281
20 to 24	Infertility	High-middle SDI	528.5527607	509.1734	548.6697
25 to 29	Infertility	High-middle SDI	635.5128543	614.4751	657.2709
30 to 34	Infertility	High-middle SDI	706.434551	683.7295	729.8936
35 to 39	Infertility	High-middle SDI	768.7559951	743.7826	794.5679
40 to 44	Infertility	High-middle SDI	793.2777003	766.267	821.2405
45 to 49	Infertility	High-middle SDI	336.3982576	319.3809	354.3223
15 to 19	Infertility	High SDI	163.532533	153.1451	174.6245
20 to 24	Infertility	High SDI	1080.509181	1047.57	1114.484
25 to 29	Infertility	High SDI	1251.6696	1217.143	1287.176
30 to 34	Infertility	High SDI	1277.680986	1243.097	1313.227
35 to 39	Infertility	High SDI	1362.684871	1326.256	1400.114
40 to 44	Infertility	High SDI	1333.595372	1296.661	1371.582
45 to 49	Infertility	High SDI	403.0337616	385.1008	421.8019
15 to 19	Infertility	Low-middle SDI	204.1982923	199.6791	208.8198
20 to 24	Infertility	Low-middle SDI	379.6047101	372.6729	386.6654
25 to 29	Infertility	Low-middle SDI	419.7686919	412.3719	427.2982

30 to 34	Infertility	Low-middle SDI	452.1328334	444.1272	460.2828
35 to 39	Infertility	Low-middle SDI	485.3599688	476.4661	494.4199
40 to 44	Infertility	Low-middle SDI	510.7134539	500.6049	521.0261
45 to 49	Infertility	Low-middle SDI	189.2422263	182.8069	195.9041
15 to 19	Infertility	Low SDI	164.9025129	162.0866	167.7673
20 to 24	Infertility	Low SDI	243.7281179	240.0904	247.421
25 to 29	Infertility	Low SDI	267.0551637	263.1674	271.0003
30 to 34	Infertility	Low SDI	283.140279	278.9408	287.403
35 to 39	Infertility	Low SDI	294.7920659	290.1739	299.4837
40 to 44	Infertility	Low SDI	303.8397959	298.5948	309.177
45 to 49	Infertility	Low SDI	93.44560775	90.38405	96.61087
15 to 19	Infertility	Middle SDI	260.4269734	252.3975	268.7119
20 to 24	Infertility	Middle SDI	622.0073527	607.4336	636.9308
25 to 29	Infertility	Middle SDI	687.5918831	672.347	703.1824
30 to 34	Infertility	Middle SDI	741.042351	724.7645	757.6857
35 to 39	Infertility	Middle SDI	796.2724139	778.3766	814.5797
40 to 44	Infertility	Middle SDI	840.1851923	820.0904	860.7724
45 to 49	Infertility	Middle SDI	342.4836268	329.4452	356.0381
15 to 19	Primary infertility	Global	118.7090435	112.715	125.0218
20 to 24	Primary infertility	Global	288.0569332	276.7782	299.7953
25 to 29	Primary infertility	Global	246.6781856	236.917	256.8416
30 to 34	Primary infertility	Global	158.0787134	150.9545	165.5392
35 to 39	Primary infertility	Global	116.5278815	110.5137	122.8693
40 to 44	Primary infertility	Global	116.7322166	110.214	123.6359
45 to 49	Primary infertility	Global	43.15754219	39.27054	47.42928
15 to 19	Primary infertility	High-middle SDI	97.01548303	89.56691	105.0835

20 to 24	Primary infertility	High-middle SDI	257.0813194	242.1072	272.9816
25 to 29	Primary infertility	High-middle SDI	224.8573109	211.9697	238.5285
30 to 34	Primary infertility	High-middle SDI	133.9242858	125.0651	143.4111
35 to 39	Primary infertility	High-middle SDI	98.14820349	90.73625	106.1656
40 to 44	Primary infertility	High-middle SDI	97.25494508	89.35183	105.8571
45 to 49	Primary infertility	High-middle SDI	35.24537558	30.77194	40.36913
15 to 19	Primary infertility	High SDI	81.46282354	75.57444	87.80999
20 to 24	Primary infertility	High SDI	634.9462218	612.1888	658.5496
25 to 29	Primary infertility	High SDI	605.9127091	584.7798	627.8094
30 to 34	Primary infertility	High SDI	367.1188598	352.8206	381.9966
35 to 39	Primary infertility	High SDI	241.0838237	230.398	252.2653
40 to 44	Primary infertility	High SDI	202.6979433	192.7448	213.1651
45 to 49	Primary infertility	High SDI	56.34821878	51.73349	61.37458
15 to 19	Primary infertility	Low-middle SDI	129.0974711	123.288	135.1807
20 to 24	Primary infertility	Low-middle SDI	206.8326109	198.6961	215.3024
25 to 29	Primary infertility	Low-middle SDI	158.3034015	151.7481	165.1419
30 to 34	Primary infertility	Low-middle SDI	109.5486705	104.3539	115.0021
35 to 39	Primary infertility	Low-middle SDI	90.75652814	85.86203	95.93003
40 to 44	Primary infertility	Low-middle SDI	100.0732725	94.27932	106.2233
45 to 49	Primary infertility	Low-middle SDI	42.89713194	38.83951	47.37866
15 to 19	Primary infertility	Low SDI	88.50555032	83.50916	93.80088
20 to 24	Primary infertility	Low SDI	114.8258723	108.932	121.0387
25 to 29	Primary infertility	Low SDI	87.47268555	82.69677	92.52442
30 to 34	Primary infertility	Low SDI	60.56500347	56.71637	64.67479
35 to 39	Primary infertility	Low SDI	45.50485802	42.07737	49.21154
40 to 44	Primary infertility	Low SDI	43.6539932	39.87489	47.79126

45 to 49	Primary infertility	Low SDI	16.46061886	13.9441	19.4313
15 to 19	Primary infertility	Middle SDI	135.2705542	130.548	140.1639
20 to 24	Primary infertility	Middle SDI	294.7771769	286.3942	303.4056
25 to 29	Primary infertility	Middle SDI	234.7187727	227.8261	241.82
30 to 34	Primary infertility	Middle SDI	150.4754824	145.4024	155.7256
35 to 39	Primary infertility	Middle SDI	114.3762929	109.9275	119.0051
40 to 44	Primary infertility	Middle SDI	130.1698446	124.7942	135.7771
45 to 49	Primary infertility	Middle SDI	58.92449517	55.09808	63.01664
15 to 19	Secondary infertility	Global	105.1617066	100.96	109.5382
20 to 24	Secondary infertility	Global	272.1677937	264.4145	280.1484
25 to 29	Secondary infertility	Global	390.1031482	380.4025	400.0512
30 to 34	Secondary infertility	Global	528.7316242	516.5696	541.1799
35 to 39	Secondary infertility	Global	631.3825873	617.0716	646.0254
40 to 44	Secondary infertility	Global	658.567128	642.9576	674.5556
45 to 49	Secondary infertility	Global	245.1587675	235.9709	254.7044
15 to 19	Secondary infertility	High-middle SDI	111.3502888	105.4768	117.5509
20 to 24	Secondary infertility	High-middle SDI	273.555764	263.3501	284.1569
25 to 29	Secondary infertility	High-middle SDI	408.9548434	396.06	422.2696
30 to 34	Secondary infertility	High-middle SDI	568.6401219	552.3508	585.4099
35 to 39	Secondary infertility	High-middle SDI	670.020128	651.0598	689.5327
40 to 44	Secondary infertility	High-middle SDI	696.1954219	675.7016	717.3108
45 to 49	Secondary infertility	High-middle SDI	301.9959797	288.9364	315.6459
15 to 19	Secondary infertility	High SDI	81.36850248	73.98547	89.48829
20 to 24	Secondary infertility	High SDI	441.6442303	421.7776	462.4466
25 to 29	Secondary infertility	High SDI	637.1280025	613.203	661.9864
30 to 34	Secondary infertility	High SDI	904.9039026	874.4885	936.3772

35 to 39	Secondary infertility	High SDI	1127.580509	1091.613	1164.733
40 to 44	Secondary infertility	High SDI	1142.472897	1105.415	1180.773
45 to 49	Secondary infertility	High SDI	351.4050801	333.4301	370.3491
15 to 19	Secondary infertility	Low-middle SDI	80.44305351	74.83705	86.469
20 to 24	Secondary infertility	Low-middle SDI	175.0989522	165.4784	185.2789
25 to 29	Secondary infertility	Low-middle SDI	256.8863485	244.2514	270.1749
30 to 34	Secondary infertility	Low-middle SDI	337.8281226	322.0973	354.3272
35 to 39	Secondary infertility	Low-middle SDI	392.8809949	374.467	412.2005
40 to 44	Secondary infertility	Low-middle SDI	410.5209899	389.8727	432.2628
45 to 49	Secondary infertility	Low-middle SDI	146.0152896	133.4696	159.7402
15 to 19	Secondary infertility	Low SDI	79.87427842	75.9063	84.04968
20 to 24	Secondary infertility	Low SDI	130.5732321	125.0188	136.3745
25 to 29	Secondary infertility	Low SDI	177.2297038	170.29	184.4523
30 to 34	Secondary infertility	Low SDI	219.8042067	211.4612	228.4763
35 to 39	Secondary infertility	Low SDI	248.1503912	238.4621	258.2324
40 to 44	Secondary infertility	Low SDI	259.9634901	248.9433	271.4715
45 to 49	Secondary infertility	Low SDI	76.9806	70.85207	83.63923
15 to 19	Secondary infertility	Middle SDI	127.6303564	121.8786	133.6535
20 to 24	Secondary infertility	Middle SDI	330.1842184	319.3379	341.3989
25 to 29	Secondary infertility	Middle SDI	451.9863242	438.7895	465.5801
30 to 34	Secondary infertility	Middle SDI	588.5470519	572.4292	605.1187
35 to 39	Secondary infertility	Middle SDI	680.5926002	661.9734	699.7355
40 to 44	Secondary infertility	Middle SDI	708.3956655	687.8717	729.532
45 to 49	Secondary infertility	Middle SDI	282.852667	269.8656	296.4647

Table S6. The results of period effect.

Age	Type	Location	Rate	CILo	CIHi
1992 to 1996	Infertility	Global	1	1	1
1997 to 2001	Infertility	Global	1.069082054	1.052091135	1.086347371
2002 to 2006	Infertility	Global	1.126432145	1.108727366	1.144419645
2007 to 2011	Infertility	Global	1.161606957	1.143666116	1.179829238
2012 to 2016	Infertility	Global	1.213594297	1.195281631	1.232187528
2017 to 2021	Infertility	Global	1.28118469	1.262323559	1.300327636
1992 to 1996	Infertility	High-middle SDI	1	1	1
1997 to 2001	Infertility	High-middle SDI	1.132537957	1.089158715	1.177644917
2002 to 2006	Infertility	High-middle SDI	1.258800275	1.211061663	1.308420686
2007 to 2011	Infertility	High-middle SDI	1.319065444	1.269400318	1.370673712
2012 to 2016	Infertility	High-middle SDI	1.366611064	1.31562211	1.419576174
2017 to 2021	Infertility	High-middle SDI	1.436175164	1.383788641	1.4905449
1992 to 1996	Infertility	High SDI	1	1	1
1997 to 2001	Infertility	High SDI	1.041398426	1.01072787	1.07299968
2002 to 2006	Infertility	High SDI	1.028223724	0.996737921	1.060704127
2007 to 2011	Infertility	High SDI	0.987298826	0.956212904	1.019395333
2012 to 2016	Infertility	High SDI	1.016606438	0.984533733	1.049723961
2017 to 2021	Infertility	High SDI	1.136035487	1.10184051	1.171291685
1992 to 1996	Infertility	Low-middle SDI	1	1	1
1997 to 2001	Infertility	Low-middle SDI	1.058791761	1.035174294	1.082948059
2002 to 2006	Infertility	Low-middle SDI	1.141681787	1.116978758	1.166931147
2007 to 2011	Infertility	Low-middle SDI	1.247304848	1.221174233	1.273994604
2012 to 2016	Infertility	Low-middle SDI	1.358645052	1.330750818	1.387123985
2017 to 2021	Infertility	Low-middle SDI	1.463786195	1.433499289	1.494713002

1992 to 1996	Infertility	Low SDI	1	1	1
1997 to 2001	Infertility	Low SDI	1.051585707	1.031805174	1.071745449
2002 to 2006	Infertility	Low SDI	1.115270758	1.09500828	1.135908181
2007 to 2011	Infertility	Low SDI	1.179623387	1.158814479	1.200805961
2012 to 2016	Infertility	Low SDI	1.241102356	1.219412211	1.263178312
2017 to 2021	Infertility	Low SDI	1.326278092	1.302432347	1.350560421
1992 to 1996	Infertility	Middle SDI	1	1	1
1997 to 2001	Infertility	Middle SDI	1.10848413	1.078254055	1.13956174
2002 to 2006	Infertility	Middle SDI	1.225503836	1.192942185	1.258954265
2007 to 2011	Infertility	Middle SDI	1.325622745	1.29118492	1.360979078
2012 to 2016	Infertility	Middle SDI	1.438516497	1.401678245	1.476322915
2017 to 2021	Infertility	Middle SDI	1.547694577	1.508134876	1.588291964
1992 to 1996	Primary infertility	Global	1	1	1
1997 to 2001	Primary infertility	Global	1.036243398	0.9872596	1.087657573
2002 to 2006	Primary infertility	Global	1.069223529	1.018514621	1.122457087
2007 to 2011	Primary infertility	Global	1.069986344	1.019128851	1.123381774
2012 to 2016	Primary infertility	Global	1.063779144	1.012473824	1.117684271
2017 to 2021	Primary infertility	Global	1.128523587	1.071705078	1.188354438
1992 to 1996	Primary infertility	High-middle SDI	1	1	1
1997 to 2001	Primary infertility	High-middle SDI	1.105525328	1.029516038	1.187146392
2002 to 2006	Primary infertility	High-middle SDI	1.230375007	1.14595052	1.321019216
2007 to 2011	Primary infertility	High-middle SDI	1.23849272	1.152742021	1.330622282
2012 to 2016	Primary infertility	High-middle SDI	1.201405756	1.116337822	1.292956094
2017 to 2021	Primary infertility	High-middle SDI	1.223610831	1.132917672	1.321564226
1992 to 1996	Primary infertility	High SDI	1	1	1
1997 to 2001	Primary infertility	High SDI	1.014176576	0.974491665	1.055477603

2002 to 2006	Primary infertility	High SDI	0.974476097	0.934489416	1.016173803
2007 to 2011	Primary infertility	High SDI	0.911961649	0.873389167	0.952237653
2012 to 2016	Primary infertility	High SDI	0.925576238	0.886054821	0.96686046
2017 to 2021	Primary infertility	High SDI	1.015133338	0.971903951	1.060285528
1992 to 1996	Primary infertility	Low-middle SDI	1	1	1
1997 to 2001	Primary infertility	Low-middle SDI	1.053262155	0.999496933	1.109919531
2002 to 2006	Primary infertility	Low-middle SDI	1.15132104	1.093203502	1.212528259
2007 to 2011	Primary infertility	Low-middle SDI	1.212038775	1.150738403	1.276604647
2012 to 2016	Primary infertility	Low-middle SDI	1.177118793	1.115456352	1.242189935
2017 to 2021	Primary infertility	Low-middle SDI	1.304801891	1.231712739	1.382228113
1992 to 1996	Primary infertility	Low SDI	1	1	1
1997 to 2001	Primary infertility	Low SDI	1.06212582	0.9893186	1.140291163
2002 to 2006	Primary infertility	Low SDI	1.136323152	1.057930655	1.22052452
2007 to 2011	Primary infertility	Low SDI	1.161924599	1.078962453	1.251265759
2012 to 2016	Primary infertility	Low SDI	1.151683249	1.063664181	1.246985965
2017 to 2021	Primary infertility	Low SDI	1.285446957	1.176497136	1.404486104
1992 to 1996	Primary infertility	Middle SDI	1	1	1
1997 to 2001	Primary infertility	Middle SDI	1.088081635	1.048338086	1.129331901
2002 to 2006	Primary infertility	Middle SDI	1.181802583	1.13889385	1.226327937
2007 to 2011	Primary infertility	Middle SDI	1.272900866	1.226728529	1.320811065
2012 to 2016	Primary infertility	Middle SDI	1.36051068	1.309961909	1.413010025
2017 to 2021	Primary infertility	Middle SDI	1.492437467	1.433307248	1.554007068
1992 to 1996	Secondary infertility	Global	1	1	1
1997 to 2001	Secondary infertility	Global	1.095265839	1.063798835	1.127663632
2002 to 2006	Secondary infertility	Global	1.173133369	1.139592706	1.207661206
2007 to 2011	Secondary infertility	Global	1.229622564	1.195018841	1.265228295

2012 to 2016	Secondary infertility	Global	1.307735901	1.272079764	1.344391473
2017 to 2021	Secondary infertility	Global	1.367851257	1.332168263	1.404490044
1992 to 1996	Secondary infertility	High-middle SDI	1	1	1
1997 to 2001	Secondary infertility	High-middle SDI	1.148132672	1.107255987	1.190518405
2002 to 2006	Secondary infertility	High-middle SDI	1.276835097	1.231437573	1.323906222
2007 to 2011	Secondary infertility	High-middle SDI	1.354947651	1.306922978	1.404737056
2012 to 2016	Secondary infertility	High-middle SDI	1.425051846	1.375134426	1.476781269
2017 to 2021	Secondary infertility	High-middle SDI	1.498270825	1.447523682	1.550797057
1992 to 1996	Secondary infertility	High SDI	1	1	1
1997 to 2001	Secondary infertility	High SDI	1.068929043	1.028194841	1.11127702
2002 to 2006	Secondary infertility	High SDI	1.072375957	1.029221631	1.117339705
2007 to 2011	Secondary infertility	High SDI	1.035923542	0.992567119	1.081173822
2012 to 2016	Secondary infertility	High SDI	1.06048108	1.015688794	1.107248724
2017 to 2021	Secondary infertility	High SDI	1.180704132	1.132935472	1.230486892
1992 to 1996	Secondary infertility	Low-middle SDI	1	1	1
1997 to 2001	Secondary infertility	Low-middle SDI	1.085541838	1.017555525	1.158070546
2002 to 2006	Secondary infertility	Low-middle SDI	1.185348424	1.113059706	1.262332001
2007 to 2011	Secondary infertility	Low-middle SDI	1.336429559	1.257994151	1.419755383
2012 to 2016	Secondary infertility	Low-middle SDI	1.535610268	1.449224032	1.627145869
2017 to 2021	Secondary infertility	Low-middle SDI	1.606829148	1.518220228	1.700609611
1992 to 1996	Secondary infertility	Low SDI	1	1	1
1997 to 2001	Secondary infertility	Low SDI	1.064958664	1.010694057	1.122136762
2002 to 2006	Secondary infertility	Low SDI	1.14774317	1.091319068	1.207084549
2007 to 2011	Secondary infertility	Low SDI	1.245809048	1.187016716	1.307513334
2012 to 2016	Secondary infertility	Low SDI	1.340940541	1.279634117	1.405184114
2017 to 2021	Secondary infertility	Low SDI	1.39515224	1.331363039	1.461997755

1992 to 1996	Secondary infertility	Middle SDI	1	1	1
1997 to 2001	Secondary infertility	Middle SDI	1.119233188	1.079404782	1.160531202
2002 to 2006	Secondary infertility	Middle SDI	1.251989659	1.208452825	1.29709499
2007 to 2011	Secondary infertility	Middle SDI	1.365903121	1.319423847	1.414019719
2012 to 2016	Secondary infertility	Middle SDI	1.497114114	1.447322374	1.548618824
2017 to 2021	Secondary infertility	Middle SDI	1.597233019	1.545284784	1.650927611

Table S7. The results of cohort effect.

Cohort	Type	Location	Rate Ratio	CILo	CIHi
1942 to 1951	Infertility	Global	0.808488	0.760725	0.859251
1947 to 1956	Infertility	Global	0.83055	0.806276	0.855554
1952 to 1961	Infertility	Global	0.844926	0.826553	0.863708
1957 to 1966	Infertility	Global	0.876634	0.860868	0.892688
1962 to 1971	Infertility	Global	0.906215	0.891907	0.920752
1967 to 1976	Infertility	Global	0.95396	0.940116	0.968008
1972 to 1981	Infertility	Global	1	1	1
1977 to 1986	Infertility	Global	1.052042	1.036459	1.06786
1982 to 1991	Infertility	Global	1.111566	1.093899	1.129518
1987 to 1996	Infertility	Global	1.186669	1.165012	1.208728
1992 to 2001	Infertility	Global	1.251477	1.222432	1.281213
1997 to 2006	Infertility	Global	1.346149	1.29456	1.399794
1942 to 1951	Infertility	High-middle SDI	0.734399	0.640948	0.841475
1947 to 1956	Infertility	High-middle SDI	0.728993	0.680294	0.781177
1952 to 1961	Infertility	High-middle SDI	0.74568	0.707902	0.785474
1957 to 1966	Infertility	High-middle SDI	0.808129	0.774033	0.843728
1962 to 1971	Infertility	High-middle SDI	0.870626	0.838234	0.90427
1967 to 1976	Infertility	High-middle SDI	0.929833	0.897532	0.963296
1972 to 1981	Infertility	High-middle SDI	1	1	1
1977 to 1986	Infertility	High-middle SDI	1.051512	1.013018	1.091469
1982 to 1991	Infertility	High-middle SDI	1.124749	1.08046	1.170852
1987 to 1996	Infertility	High-middle SDI	1.278867	1.218914	1.341768
1992 to 2001	Infertility	High-middle SDI	1.418927	1.329412	1.51447
1997 to 2006	Infertility	High-middle SDI	1.385334	1.229404	1.561041

1942 to 1951	Infertility	High SDI	0.964101	0.868984	1.06963
1947 to 1956	Infertility	High SDI	0.95279	0.905041	1.003059
1952 to 1961	Infertility	High SDI	0.922008	0.886504	0.958934
1957 to 1966	Infertility	High SDI	0.941245	0.910005	0.973557
1962 to 1971	Infertility	High SDI	0.972786	0.94308	1.003427
1967 to 1976	Infertility	High SDI	1.006297	0.977276	1.036181
1972 to 1981	Infertility	High SDI	1	1	1
1977 to 1986	Infertility	High SDI	1.000131	0.969096	1.032159
1982 to 1991	Infertility	High SDI	1.024051	0.988704	1.060661
1987 to 1996	Infertility	High SDI	1.060867	1.0177	1.105864
1992 to 2001	Infertility	High SDI	1.102269	1.041168	1.166956
1997 to 2006	Infertility	High SDI	1.063402	0.915288	1.235486
1942 to 1951	Infertility	Low-middle SDI	0.650491	0.589052	0.718337
1947 to 1956	Infertility	Low-middle SDI	0.691599	0.65992	0.724799
1952 to 1961	Infertility	Low-middle SDI	0.739596	0.715366	0.764646
1957 to 1966	Infertility	Low-middle SDI	0.788438	0.767799	0.809632
1962 to 1971	Infertility	Low-middle SDI	0.843222	0.824442	0.86243
1967 to 1976	Infertility	Low-middle SDI	0.908639	0.890709	0.92693
1972 to 1981	Infertility	Low-middle SDI	1	1	1
1977 to 1986	Infertility	Low-middle SDI	1.088031	1.067492	1.108965
1982 to 1991	Infertility	Low-middle SDI	1.176341	1.152872	1.200289
1987 to 1996	Infertility	Low-middle SDI	1.264655	1.23675	1.29319
1992 to 2001	Infertility	Low-middle SDI	1.360159	1.324219	1.397074
1997 to 2006	Infertility	Low-middle SDI	1.567107	1.50709	1.629514
1942 to 1951	Infertility	Low SDI	0.787268	0.716516	0.865007
1947 to 1956	Infertility	Low SDI	0.773531	0.741563	0.806877

1952 to 1961	Infertility	Low SDI	0.80683	0.783298	0.831069
1957 to 1966	Infertility	Low SDI	0.834629	0.815359	0.854354
1962 to 1971	Infertility	Low SDI	0.881562	0.864528	0.898931
1967 to 1976	Infertility	Low SDI	0.9376	0.921846	0.953624
1972 to 1981	Infertility	Low SDI	1	1	1
1977 to 1986	Infertility	Low SDI	1.061173	1.044624	1.077985
1982 to 1991	Infertility	Low SDI	1.130702	1.112318	1.14939
1987 to 1996	Infertility	Low SDI	1.204964	1.18375	1.226559
1992 to 2001	Infertility	Low SDI	1.275247	1.249259	1.301776
1997 to 2006	Infertility	Low SDI	1.353227	1.315853	1.391663
1942 to 1951	Infertility	Middle SDI	0.601389	0.536331	0.674339
1947 to 1956	Infertility	Middle SDI	0.646175	0.611719	0.682572
1952 to 1961	Infertility	Middle SDI	0.700824	0.673674	0.729069
1957 to 1966	Infertility	Middle SDI	0.753886	0.730496	0.778025
1962 to 1971	Infertility	Middle SDI	0.805492	0.784242	0.827318
1967 to 1976	Infertility	Middle SDI	0.891292	0.869961	0.913146
1972 to 1981	Infertility	Middle SDI	1	1	1
1977 to 1986	Infertility	Middle SDI	1.099895	1.073425	1.127017
1982 to 1991	Infertility	Middle SDI	1.178982	1.148842	1.209911
1987 to 1996	Infertility	Middle SDI	1.285752	1.248067	1.324575
1992 to 2001	Infertility	Middle SDI	1.388581	1.336863	1.442299
1997 to 2006	Infertility	Middle SDI	1.454431	1.365233	1.549458
1942 to 1951	Primary infertility	Global	0.885014	0.685181	1.143128
1947 to 1956	Primary infertility	Global	0.915984	0.81	1.035835
1952 to 1961	Primary infertility	Global	0.94523	0.864848	1.033082
1957 to 1966	Primary infertility	Global	0.968345	0.904238	1.036998

1962 to 1971	Primary infertility	Global	0.955984	0.904567	1.010324
1967 to 1976	Primary infertility	Global	0.974833	0.92935	1.022542
1972 to 1981	Primary infertility	Global	1	1	1
1977 to 1986	Primary infertility	Global	1.024019	0.977433	1.072825
1982 to 1991	Primary infertility	Global	1.040479	0.991747	1.091605
1987 to 1996	Primary infertility	Global	1.092349	1.037824	1.149739
1992 to 2001	Primary infertility	Global	1.101638	1.035442	1.172066
1997 to 2006	Primary infertility	Global	1.098045	0.991337	1.216239
1942 to 1951	Primary infertility	High-middle SDI	0.955724	0.668982	1.36537
1947 to 1956	Primary infertility	High-middle SDI	0.895408	0.75177	1.066491
1952 to 1961	Primary infertility	High-middle SDI	0.912975	0.803903	1.036845
1957 to 1966	Primary infertility	High-middle SDI	0.954872	0.865208	1.053827
1962 to 1971	Primary infertility	High-middle SDI	0.923369	0.852122	1.000573
1967 to 1976	Primary infertility	High-middle SDI	0.933207	0.86969	1.001362
1972 to 1981	Primary infertility	High-middle SDI	1	1	1
1977 to 1986	Primary infertility	High-middle SDI	1.022265	0.953506	1.095982
1982 to 1991	Primary infertility	High-middle SDI	1.047685	0.975027	1.125756
1987 to 1996	Primary infertility	High-middle SDI	1.223376	1.131768	1.3224
1992 to 2001	Primary infertility	High-middle SDI	1.374009	1.245488	1.515792
1997 to 2006	Primary infertility	High-middle SDI	1.29611	1.084391	1.549165
1942 to 1951	Primary infertility	High SDI	1.117989	0.914306	1.367047
1947 to 1956	Primary infertility	High SDI	1.171845	1.066746	1.287299
1952 to 1961	Primary infertility	High SDI	1.103212	1.029548	1.182145
1957 to 1966	Primary infertility	High SDI	1.082731	1.025185	1.143507
1962 to 1971	Primary infertility	High SDI	1.057319	1.009831	1.107039
1967 to 1976	Primary infertility	High SDI	1.035137	0.993331	1.078702

1972 to 1981	Primary infertility	High SDI	1	1	1
1977 to 1986	Primary infertility	High SDI	0.986929	0.945547	1.030122
1982 to 1991	Primary infertility	High SDI	1.007439	0.963062	1.053861
1987 to 1996	Primary infertility	High SDI	1.065714	1.014949	1.119017
1992 to 2001	Primary infertility	High SDI	1.118102	1.049469	1.191224
1997 to 2006	Primary infertility	High SDI	1.102535	0.932877	1.303047
1942 to 1951	Primary infertility	Low-middle SDI	0.662768	0.493242	0.890561
1947 to 1956	Primary infertility	Low-middle SDI	0.67295	0.580155	0.780589
1952 to 1961	Primary infertility	Low-middle SDI	0.748155	0.674314	0.830083
1957 to 1966	Primary infertility	Low-middle SDI	0.826554	0.764707	0.893402
1962 to 1971	Primary infertility	Low-middle SDI	0.905059	0.851755	0.961698
1967 to 1976	Primary infertility	Low-middle SDI	0.962186	0.915	1.011807
1972 to 1981	Primary infertility	Low-middle SDI	1	1	1
1977 to 1986	Primary infertility	Low-middle SDI	1.012767	0.966404	1.061354
1982 to 1991	Primary infertility	Low-middle SDI	1.047189	0.998065	1.098729
1987 to 1996	Primary infertility	Low-middle SDI	1.086438	1.033381	1.14222
1992 to 2001	Primary infertility	Low-middle SDI	1.071956	1.012197	1.135243
1997 to 2006	Primary infertility	Low-middle SDI	1.103488	1.015938	1.198584
1942 to 1951	Primary infertility	Low SDI	0.632229	0.372736	1.072377
1947 to 1956	Primary infertility	Low SDI	0.681579	0.535269	0.86788
1952 to 1961	Primary infertility	Low SDI	0.719106	0.609742	0.848086
1957 to 1966	Primary infertility	Low SDI	0.79411	0.705851	0.893406
1962 to 1971	Primary infertility	Low SDI	0.91852	0.842488	1.001413
1967 to 1976	Primary infertility	Low SDI	0.98307	0.917579	1.053236
1972 to 1981	Primary infertility	Low SDI	1	1	1
1977 to 1986	Primary infertility	Low SDI	0.988322	0.929217	1.051187

1982 to 1991	Primary infertility	Low SDI	0.985559	0.925552	1.049456
1987 to 1996	Primary infertility	Low SDI	1.017566	0.954147	1.0852
1992 to 2001	Primary infertility	Low SDI	1.029316	0.959004	1.104783
1997 to 2006	Primary infertility	Low SDI	0.997101	0.906344	1.096947
1942 to 1951	Primary infertility	Middle SDI	0.596824	0.487922	0.730032
1947 to 1956	Primary infertility	Middle SDI	0.611399	0.552629	0.676418
1952 to 1961	Primary infertility	Middle SDI	0.694714	0.646404	0.746635
1957 to 1966	Primary infertility	Middle SDI	0.745341	0.705894	0.786992
1962 to 1971	Primary infertility	Middle SDI	0.781097	0.74878	0.81481
1967 to 1976	Primary infertility	Middle SDI	0.874728	0.844262	0.906295
1972 to 1981	Primary infertility	Middle SDI	1	1	1
1977 to 1986	Primary infertility	Middle SDI	1.100173	1.063855	1.137732
1982 to 1991	Primary infertility	Middle SDI	1.12893	1.090807	1.168385
1987 to 1996	Primary infertility	Middle SDI	1.181763	1.139129	1.225993
1992 to 2001	Primary infertility	Middle SDI	1.215545	1.163019	1.270444
1997 to 2006	Primary infertility	Middle SDI	1.215437	1.131923	1.305112
1942 to 1951	Secondary infertility	Global	0.799257	0.723654	0.882759
1947 to 1956	Secondary infertility	Global	0.818125	0.779304	0.858879
1952 to 1961	Secondary infertility	Global	0.827413	0.797874	0.858045
1957 to 1966	Secondary infertility	Global	0.857551	0.831721	0.884183
1962 to 1971	Secondary infertility	Global	0.891947	0.867789	0.916777
1967 to 1976	Secondary infertility	Global	0.945306	0.921369	0.969865
1972 to 1981	Secondary infertility	Global	1	1	1
1977 to 1986	Secondary infertility	Global	1.067111	1.039001	1.09598
1982 to 1991	Secondary infertility	Global	1.161444	1.127742	1.196154
1987 to 1996	Secondary infertility	Global	1.273112	1.228714	1.319114

1992 to 2001	Secondary infertility	Global	1.406498	1.342	1.474096
1997 to 2006	Secondary infertility	Global	1.611413	1.490556	1.74207
1942 to 1951	Secondary infertility	High-middle SDI	0.709079	0.632108	0.795422
1947 to 1956	Secondary infertility	High-middle SDI	0.7075	0.66711	0.750336
1952 to 1961	Secondary infertility	High-middle SDI	0.722867	0.691327	0.755846
1957 to 1966	Secondary infertility	High-middle SDI	0.785997	0.757003	0.816101
1962 to 1971	Secondary infertility	High-middle SDI	0.861093	0.832605	0.890556
1967 to 1976	Secondary infertility	High-middle SDI	0.930138	0.901054	0.960161
1972 to 1981	Secondary infertility	High-middle SDI	1	1	1
1977 to 1986	Secondary infertility	High-middle SDI	1.060884	1.025137	1.097878
1982 to 1991	Secondary infertility	High-middle SDI	1.165334	1.122053	1.210285
1987 to 1996	Secondary infertility	High-middle SDI	1.323586	1.261889	1.388299
1992 to 2001	Secondary infertility	High-middle SDI	1.466268	1.369842	1.569482
1997 to 2006	Secondary infertility	High-middle SDI	1.473228	1.301948	1.66704
1942 to 1951	Secondary infertility	High SDI	0.939813	0.835227	1.057495
1947 to 1956	Secondary infertility	High SDI	0.914038	0.861468	0.969816
1952 to 1961	Secondary infertility	High SDI	0.882215	0.842623	0.923667
1957 to 1966	Secondary infertility	High SDI	0.899554	0.86375	0.936842
1962 to 1971	Secondary infertility	High SDI	0.936289	0.900897	0.973072
1967 to 1976	Secondary infertility	High SDI	0.989146	0.953361	1.026275
1972 to 1981	Secondary infertility	High SDI	1	1	1
1977 to 1986	Secondary infertility	High SDI	1.013905	0.973833	1.055627
1982 to 1991	Secondary infertility	High SDI	1.050767	1.00242	1.101446
1987 to 1996	Secondary infertility	High SDI	1.071723	1.008765	1.138609
1992 to 2001	Secondary infertility	High SDI	1.094699	1.001951	1.196031
1997 to 2006	Secondary infertility	High SDI	1.040309	0.833332	1.298694

1942 to 1951	Secondary infertility	Low-middle SDI	0.654283	0.507366	0.843741
1947 to 1956	Secondary infertility	Low-middle SDI	0.700787	0.622506	0.788911
1952 to 1961	Secondary infertility	Low-middle SDI	0.741367	0.6812	0.806847
1957 to 1966	Secondary infertility	Low-middle SDI	0.783997	0.731937	0.839761
1962 to 1971	Secondary infertility	Low-middle SDI	0.829619	0.7813	0.880927
1967 to 1976	Secondary infertility	Low-middle SDI	0.889785	0.842649	0.939558
1972 to 1981	Secondary infertility	Low-middle SDI	1	1	1
1977 to 1986	Secondary infertility	Low-middle SDI	1.125833	1.067906	1.186902
1982 to 1991	Secondary infertility	Low-middle SDI	1.264379	1.193961	1.338952
1987 to 1996	Secondary infertility	Low-middle SDI	1.442643	1.350357	1.541236
1992 to 2001	Secondary infertility	Low-middle SDI	1.728714	1.592803	1.876223
1997 to 2006	Secondary infertility	Low-middle SDI	2.243775	1.988616	2.531672
1942 to 1951	Secondary infertility	Low SDI	0.825702	0.656788	1.038057
1947 to 1956	Secondary infertility	Low SDI	0.794964	0.717553	0.880726
1952 to 1961	Secondary infertility	Low SDI	0.828104	0.770308	0.890236
1957 to 1966	Secondary infertility	Low SDI	0.849332	0.801239	0.900312
1962 to 1971	Secondary infertility	Low SDI	0.877461	0.834457	0.922682
1967 to 1976	Secondary infertility	Low SDI	0.923593	0.883168	0.965867
1972 to 1981	Secondary infertility	Low SDI	1	1	1
1977 to 1986	Secondary infertility	Low SDI	1.092932	1.047818	1.139989
1982 to 1991	Secondary infertility	Low SDI	1.213414	1.160358	1.268897
1987 to 1996	Secondary infertility	Low SDI	1.344894	1.279327	1.413822
1992 to 2001	Secondary infertility	Low SDI	1.498072	1.411262	1.590221
1997 to 2006	Secondary infertility	Low SDI	1.705836	1.572126	1.850917
1942 to 1951	Secondary infertility	Middle SDI	0.604835	0.527058	0.69409
1947 to 1956	Secondary infertility	Middle SDI	0.655594	0.614105	0.699886

1952 to 1961	Secondary infertility	Middle SDI	0.704372	0.671787	0.738537
1957 to 1966	Secondary infertility	Middle SDI	0.757533	0.729062	0.787116
1962 to 1971	Secondary infertility	Middle SDI	0.812473	0.785952	0.839889
1967 to 1976	Secondary infertility	Middle SDI	0.896205	0.869144	0.924109
1972 to 1981	Secondary infertility	Middle SDI	1	1	1
1977 to 1986	Secondary infertility	Middle SDI	1.100634	1.066451	1.135913
1982 to 1991	Secondary infertility	Middle SDI	1.207063	1.166332	1.249217
1987 to 1996	Secondary infertility	Middle SDI	1.35822	1.30364	1.415086
1992 to 2001	Secondary infertility	Middle SDI	1.532753	1.451814	1.618204
1997 to 2006	Secondary infertility	Middle SDI	1.673396	1.525952	1.835087

Table S8. The results of local drift.

Age	Type	Location	Mean Percent Change per Calendar Year	CILo	CIHi
15 to 19	Infertility	Global	1.191265491	1.06395	1.318741
20 to 24	Infertility	Global	1.1065205	1.02409	1.189019
25 to 29	Infertility	Global	1.067131496	0.996782	1.13753
30 to 34	Infertility	Global	0.965747907	0.896675	1.034868
35 to 39	Infertility	Global	0.885349087	0.809118	0.961638
40 to 44	Infertility	Global	0.760387248	0.663639	0.857228
45 to 49	Infertility	Global	0.645321662	0.459566	0.831421
15 to 19	Infertility	High-middle SDI	1.529950043	1.150524	1.910799
20 to 24	Infertility	High-middle SDI	1.681704649	1.456972	1.906935
25 to 29	Infertility	High-middle SDI	1.464182589	1.2843	1.644385
30 to 34	Infertility	High-middle SDI	1.318345045	1.148721	1.488254
35 to 39	Infertility	High-middle SDI	1.394393167	1.210956	1.578163
40 to 44	Infertility	High-middle SDI	1.332847045	1.105804	1.5604
45 to 49	Infertility	High-middle SDI	1.029743978	0.612187	1.449034
15 to 19	Infertility	High SDI	0.363177975	-0.08848	0.816875
20 to 24	Infertility	High SDI	0.375768605	0.185447	0.566452
25 to 29	Infertility	High SDI	0.278091944	0.131326	0.425073
30 to 34	Infertility	High SDI	0.285250357	0.151518	0.419161
35 to 39	Infertility	High SDI	0.356165599	0.217777	0.494746
40 to 44	Infertility	High SDI	0.30744214	0.138492	0.476678
45 to 49	Infertility	High SDI	0.169937208	-0.147	0.487877
15 to 19	Infertility	Low-middle SDI	1.722228438	1.589437	1.855194
20 to 24	Infertility	Low-middle SDI	1.612568609	1.513837	1.711396

25 to 29	Infertility	Low-middle SDI	1.662608408	1.570257	1.755044
30 to 34	Infertility	Low-middle SDI	1.648320071	1.550948	1.745785
35 to 39	Infertility	Low-middle SDI	1.565225206	1.451562	1.679015
40 to 44	Infertility	Low-middle SDI	1.45531687	1.30303	1.607833
45 to 49	Infertility	Low-middle SDI	1.340172049	1.036801	1.644454
15 to 19	Infertility	Low SDI	1.223057478	1.12606	1.320148
20 to 24	Infertility	Low SDI	1.242322569	1.164581	1.320123
25 to 29	Infertility	Low SDI	1.255667641	1.179366	1.332026
30 to 34	Infertility	Low SDI	1.22965398	1.146181	1.313196
35 to 39	Infertility	Low SDI	1.134396548	1.03455	1.234341
40 to 44	Infertility	Low SDI	1.027695115	0.891247	1.164327
45 to 49	Infertility	Low SDI	0.74552825	0.460076	1.031792
15 to 19	Infertility	Middle SDI	1.531013936	1.324862	1.737585
20 to 24	Infertility	Middle SDI	1.752489355	1.617987	1.88717
25 to 29	Infertility	Middle SDI	1.887678072	1.770867	2.004623
30 to 34	Infertility	Middle SDI	1.895137972	1.776504	2.013911
35 to 39	Infertility	Middle SDI	1.846745301	1.710387	1.983286
40 to 44	Infertility	Middle SDI	1.712139303	1.533477	1.891116
45 to 49	Infertility	Middle SDI	1.555565419	1.205204	1.907139
15 to 19	Primary infertility	Global	0.421168104	0.089447	0.753989
20 to 24	Primary infertility	Global	0.511230097	0.298528	0.724383
25 to 29	Primary infertility	Global	0.507551858	0.303834	0.711683
30 to 34	Primary infertility	Global	0.338269734	0.098247	0.578868
35 to 39	Primary infertility	Global	0.29548337	-0.00577	0.597647
40 to 44	Primary infertility	Global	0.296696049	-0.10346	0.69845
45 to 49	Primary infertility	Global	0.363918662	-0.40942	1.14326

15 to 19	Primary infertility	High-middle SDI	1.345546082	0.77553	1.918786
20 to 24	Primary infertility	High-middle SDI	1.47576216	1.140151	1.812487
25 to 29	Primary infertility	High-middle SDI	1.019942143	0.711588	1.329241
30 to 34	Primary infertility	High-middle SDI	0.480104877	0.124841	0.836629
35 to 39	Primary infertility	High-middle SDI	0.409103477	-0.02908	0.849209
40 to 44	Primary infertility	High-middle SDI	0.334607339	-0.23739	0.909879
45 to 49	Primary infertility	High-middle SDI	0.010233276	-1.06657	1.098754
15 to 19	Primary infertility	High SDI	0.526329657	0.019994	1.035228
20 to 24	Primary infertility	High SDI	0.341723802	0.135438	0.548434
25 to 29	Primary infertility	High SDI	-0.03141281	-0.2032	0.140668
30 to 34	Primary infertility	High SDI	-0.343175009	-0.5357	-0.15028
35 to 39	Primary infertility	High SDI	-0.465527587	-0.70223	-0.22827
40 to 44	Primary infertility	High SDI	-0.57418706	-0.88226	-0.26516
45 to 49	Primary infertility	High SDI	-0.406177838	-1.00932	0.200636
15 to 19	Primary infertility	Low-middle SDI	0.400556665	0.124182	0.677694
20 to 24	Primary infertility	Low-middle SDI	0.470989317	0.264178	0.678227
25 to 29	Primary infertility	Low-middle SDI	0.676538443	0.456885	0.896672
30 to 34	Primary infertility	Low-middle SDI	0.894761525	0.62437	1.165879
35 to 39	Primary infertility	Low-middle SDI	1.234317383	0.884851	1.584995
40 to 44	Primary infertility	Low-middle SDI	1.627934896	1.145758	2.11241
45 to 49	Primary infertility	Low-middle SDI	1.643388119	0.737329	2.557597
15 to 19	Primary infertility	Low SDI	0.079670633	-0.24519	0.40559
20 to 24	Primary infertility	Low SDI	0.159718757	-0.10269	0.422822
25 to 29	Primary infertility	Low SDI	0.290629761	-0.00755	0.589697
30 to 34	Primary infertility	Low SDI	0.755263626	0.360564	1.151515
35 to 39	Primary infertility	Low SDI	1.351632692	0.808561	1.89763

40 to 44	Primary infertility	Low SDI	1.729222903	0.94917	2.515303
45 to 49	Primary infertility	Low SDI	1.846214666	0.242719	3.47536
15 to 19	Primary infertility	Middle SDI	0.757385416	0.525225	0.990082
20 to 24	Primary infertility	Middle SDI	1.24887616	1.094208	1.403781
25 to 29	Primary infertility	Middle SDI	1.689029882	1.533969	1.844327
30 to 34	Primary infertility	Middle SDI	1.867119232	1.677091	2.057502
35 to 39	Primary infertility	Middle SDI	1.899861487	1.654938	2.145375
40 to 44	Primary infertility	Middle SDI	1.844302598	1.513355	2.176329
45 to 49	Primary infertility	Middle SDI	1.564483317	0.94674	2.186007
15 to 19	Secondary infertility	Global	1.90698522	1.65061	2.164007
20 to 24	Secondary infertility	Global	1.610457207	1.444586	1.7766
25 to 29	Secondary infertility	Global	1.41665541	1.282404	1.551085
30 to 34	Secondary infertility	Global	1.213504725	1.091528	1.335628
35 to 39	Secondary infertility	Global	1.028774751	0.901655	1.156055
40 to 44	Secondary infertility	Global	0.827775748	0.669324	0.986477
45 to 49	Secondary infertility	Global	0.650151121	0.346291	0.954932
15 to 19	Secondary infertility	High-middle SDI	1.749683879	1.35589	2.145007
20 to 24	Secondary infertility	High-middle SDI	1.85159124	1.618137	2.085582
25 to 29	Secondary infertility	High-middle SDI	1.662164161	1.486094	1.83854
30 to 34	Secondary infertility	High-middle SDI	1.535928504	1.381573	1.690519
35 to 39	Secondary infertility	High-middle SDI	1.565093586	1.406251	1.724185
40 to 44	Secondary infertility	High-middle SDI	1.483849174	1.290796	1.677271
45 to 49	Secondary infertility	High-middle SDI	1.166727752	0.812829	1.521868
15 to 19	Secondary infertility	High SDI	0.255954024	-0.41288	0.929283
20 to 24	Secondary infertility	High SDI	0.429762682	0.134865	0.725528
25 to 29	Secondary infertility	High SDI	0.498724992	0.285797	0.712105

30 to 34	Secondary infertility	High SDI	0.588419976	0.414487	0.762655
35 to 39	Secondary infertility	High SDI	0.612228282	0.447912	0.776814
40 to 44	Secondary infertility	High SDI	0.47694067	0.282393	0.671866
45 to 49	Secondary infertility	High SDI	0.198727394	-0.16385	0.562619
15 to 19	Secondary infertility	Low-middle SDI	3.168744686	2.749992	3.589204
20 to 24	Secondary infertility	Low-middle SDI	2.626033672	2.319323	2.933664
25 to 29	Secondary infertility	Low-middle SDI	2.276332584	2.006904	2.546473
30 to 34	Secondary infertility	Low-middle SDI	1.974885427	1.712878	2.237568
35 to 39	Secondary infertility	Low-middle SDI	1.664546927	1.374555	1.955369
40 to 44	Secondary infertility	Low-middle SDI	1.370318042	0.986434	1.755662
45 to 49	Secondary infertility	Low-middle SDI	1.206883606	0.431151	1.988608
15 to 19	Secondary infertility	Low SDI	2.147941609	1.86125	2.43544
20 to 24	Secondary infertility	Low SDI	1.968748596	1.740942	2.197065
25 to 29	Secondary infertility	Low SDI	1.753958532	1.542833	1.965523
30 to 34	Secondary infertility	Low SDI	1.45155487	1.236536	1.66703
35 to 39	Secondary infertility	Low SDI	1.10812885	0.863029	1.353824
40 to 44	Secondary infertility	Low SDI	0.865019524	0.534489	1.196637
45 to 49	Secondary infertility	Low SDI	0.505103663	-0.18731	1.202322
15 to 19	Secondary infertility	Middle SDI	2.128505667	1.826955	2.430949
20 to 24	Secondary infertility	Middle SDI	2.133342863	1.940743	2.326307
25 to 29	Secondary infertility	Middle SDI	2.054216821	1.896146	2.212532
30 to 34	Secondary infertility	Middle SDI	1.93252469	1.782962	2.082308
35 to 39	Secondary infertility	Middle SDI	1.823760756	1.659493	1.988294
40 to 44	Secondary infertility	Middle SDI	1.673083599	1.460222	1.886392
45 to 49	Secondary infertility	Middle SDI	1.544638519	1.123284	1.967749

Table S9. The results of BAPC model to predict the temporal trend in the number of prevalence cases from 1990 to 2050.

Type	pred_case_val	pred_low	pred_up	year
Infertility	6315525.411	6297562.659	6333488.163	1990
Infertility	6490861.891	6472661.749	6509062.032	1991
Infertility	6655924.245	6637492.534	6674355.955	1992
Infertility	6821507.378	6802843.76	6840170.996	1993
Infertility	6984352.425	6965457.355	7003247.495	1994
Infertility	7151054.707	7131925.61	7170183.803	1995
Infertility	7337527.146	7318137.248	7356917.043	1996
Infertility	7556411.514	7536727.725	7576095.302	1997
Infertility	7793100.246	7773102.396	7813098.096	1998
Infertility	8022926.467	8002630.88	8043222.053	1999
Infertility	8232579.477	8212010.597	8253148.358	2000
Infertility	8429275.599	8408457.691	8450093.506	2001
Infertility	8641480.152	8620399.934	8662560.37	2002
Infertility	8857810.858	8836470.28	8879151.436	2003
Infertility	9069001.032	9047412.37	9090589.695	2004
Infertility	9262627.502	9240815.117	9284439.886	2005
Infertility	9445290.821	9423268.271	9467313.371	2006
Infertility	9630196.943	9607959.713	9652434.173	2007
Infertility	9819985.716	9797525.592	9842445.839	2008
Infertility	10013279.2	9990592.803	10035965.59	2009
Infertility	10197275.21	10174375.68	10220174.75	2010
Infertility	10392599.81	10369479.59	10415720.02	2011
Infertility	10604877.63	10581522.5	10628232.77	2012
Infertility	10820247.69	10796660.48	10843834.9	2013

Infertility	11022085.34	10998284.21	11045886.46	2014
Infertility	11195230.17	11171246.83	11219213.51	2015
Infertility	11385487.67	11361293.24	11409682.1	2016
Infertility	11623612.28	11599146.53	11648078.02	2017
Infertility	11870992.37	11846246.78	11895737.95	2018
Infertility	12088241.83	12063259.01	12113224.66	2019
Infertility	12344011.68	12318768.7	12369254.67	2020
Infertility	12465663.19	12440298.92	12491027.46	2021
Infertility	12715531.45	12245152.47	13185910.43	2022
Infertility	12950341.82	12381312.05	13519371.58	2023
Infertility	13190064.5	12476996.53	13903132.47	2024
Infertility	13433554.39	12535048.38	14332060.4	2025
Infertility	13682605.39	12560187.74	14805023.05	2026
Infertility	13941463.44	12557882.32	15325044.57	2027
Infertility	14210464.64	12531374.11	15889555.18	2028
Infertility	14485099.85	12477940.8	16492258.9	2029
Infertility	14759193.06	12392299.89	17126086.22	2030
Infertility	15034025.55	12274689.29	17793361.81	2031
Infertility	15314869.52	12128121.8	18501617.24	2032
Infertility	15601198.22	11952376.87	19250019.58	2033
Infertility	15890567.75	11745087.86	20036047.63	2034
Infertility	16178312.85	11501796.87	20854828.83	2035
Infertility	16474340.8	11228091.6	21720590.01	2036
Infertility	16789617.59	10928777.25	22650457.94	2037
Infertility	17117465.37	10596882.47	23638048.28	2038
Infertility	17455450.44	10228439.85	24682461.04	2039

Infertility	17801948.73	9819839.73	25784057.73	2040
Infertility	18163588.97	9371684.304	26955493.63	2041
Infertility	18550128.88	8884995.431	28215262.33	2042
Infertility	18959638.27	8353496.846	29565779.7	2043
Infertility	19390372.1	7770918.115	31009826.08	2044
Infertility	19839197.83	7130234.39	32548161.27	2045
Infertility	20307373.35	6425915.375	34188831.33	2046
Infertility	20800849.69	5653721.928	35947977.46	2047
Infertility	21319873.37	4805783.412	37833963.32	2048
Infertility	21863784.68	3934959.18	39854371.38	2049
Infertility	22431760.58	3016691.385	42017147	2050
Primary infertility	2074135.287	2064176.395	2084094.178	1990
Primary infertility	2135557.82	2125451.336	2145664.304	1991
Primary infertility	2192812.957	2182568.928	2203056.986	1992
Primary infertility	2245862.212	2235490.163	2256234.26	1993
Primary infertility	2295039.424	2284546.518	2305532.329	1994
Primary infertility	2341022.938	2330415.209	2351630.667	1995
Primary infertility	2386251.423	2375534.422	2396968.425	1996
Primary infertility	2430411.183	2419593.957	2441228.41	1997
Primary infertility	2474782.228	2463866.396	2485698.06	1998
Primary infertility	2519596.341	2508581.861	2530610.82	1999
Primary infertility	2565917.934	2554797.731	2577038.137	2000
Primary infertility	2617362.207	2606125.776	2628598.638	2001
Primary infertility	2674397.245	2663035.556	2685758.934	2002
Primary infertility	2732315.696	2720830.427	2743800.964	2003
Primary infertility	2786740.167	2775141.303	2798339.031	2004

Primary infertility	2832608.372	2820915.318	2844301.427	2005
Primary infertility	2871721.119	2859948.91	2883493.328	2006
Primary infertility	2909195.284	2897347.875	2921042.693	2007
Primary infertility	2946631.024	2934707.94	2958554.107	2008
Primary infertility	2983591.693	2971592.263	2995591.124	2009
Primary infertility	3016549.156	3004478.097	3028620.216	2010
Primary infertility	3041133.861	3028998.915	3053268.807	2011
Primary infertility	3057661.732	3045470.885	3069852.579	2012
Primary infertility	3071360.391	3059117.965	3083602.817	2013
Primary infertility	3087111.821	3074817.225	3099406.417	2014
Primary infertility	3111220.721	3098864.507	3123576.936	2015
Primary infertility	3156740.357	3144287.924	3169192.79	2016
Primary infertility	3222591.25	3210007.99	3235174.511	2017
Primary infertility	3292736.364	3280016.548	3305456.18	2018
Primary infertility	3350702.628	3337869.14	3363536.117	2019
Primary infertility	3407406.186	3394471.371	3420341	2020
Primary infertility	3452836.704	3439793.37	3465880.039	2021
Primary infertility	3509613.599	3190080.514	3829146.684	2022
Primary infertility	3567931.157	3218462.401	3917399.913	2023
Primary infertility	3627858.345	3236318.623	4019398.066	2024
Primary infertility	3688567.505	3242073.765	4135061.244	2025
Primary infertility	3751348.663	3236568.215	4266129.11	2026
Primary infertility	3817979.875	3221330.332	4414629.419	2027
Primary infertility	3888099.527	3196498.711	4579700.342	2028
Primary infertility	3960251.145	3161114.477	4759387.814	2029
Primary infertility	4032541.943	3113678.954	4951404.933	2030

Primary infertility	4105270.782	3054307.582	5156233.981	2031
Primary infertility	4179852.43	2983851.095	5375853.766	2032
Primary infertility	4254959.465	2901344.179	5608574.75	2033
Primary infertility	4329384.136	2805784.063	5852984.21	2034
Primary infertility	4402126.957	2696243.518	6108010.395	2035
Primary infertility	4474984.738	2573531.024	6376438.452	2036
Primary infertility	4549926.218	2438185.016	6661667.419	2037
Primary infertility	4625344.4	2288461.253	6962227.547	2038
Primary infertility	4701192.249	2123526.331	7278858.166	2039
Primary infertility	4777695.21	1942613.47	7612776.95	2040
Primary infertility	4856281.437	1745332.541	7967230.333	2041
Primary infertility	4938823.616	1531110.701	8346536.53	2042
Primary infertility	5026920.589	1298350.701	8755490.476	2043
Primary infertility	5121580.536	1052147.348	9198279.411	2044
Primary infertility	5222898.695	817759.2801	9677864.763	2045
Primary infertility	5331448.734	561507.9281	10198367.93	2046
Primary infertility	5447588.131	375808.951	10763614.14	2047
Primary infertility	5572982.828	198339.4726	11380830.95	2048
Primary infertility	5708579.374	73553.05418	12056769.65	2049
Primary infertility	5854827.746	3043.794912	12798016.58	2050
Secondary infertility	4241398.655	4226694.439	4256102.871	1990
Secondary infertility	4355308.818	4340419.46	4370198.175	1991
Secondary infertility	4463119.067	4448046.989	4478191.144	1992
Secondary infertility	4575652.782	4560392.316	4590913.248	1993
Secondary infertility	4689319.429	4673867.063	4704771.794	1994
Secondary infertility	4810015.086	4794365.045	4825665.128	1995

Secondary infertility	4951256.028	4935374.652	4967137.403	1996
Secondary infertility	5126000.099	5109843.63	5142156.568	1997
Secondary infertility	5318335.968	5301880.503	5334791.432	1998
Secondary infertility	5503362.564	5486626.245	5520098.882	1999
Secondary infertility	5666681.074	5649695.361	5683666.787	2000
Secondary infertility	5811913.132	5794711.331	5829114.932	2001
Secondary infertility	5967079.418	5949652.312	5984506.523	2002
Secondary infertility	6125490.807	6107839.433	6143142.18	2003
Secondary infertility	6282262.397	6264393.781	6300131.013	2004
Secondary infertility	6430015.1	6411943.815	6448086.386	2005
Secondary infertility	6573558.915	6555289.643	6591828.188	2006
Secondary infertility	6720994.496	6702518.682	6739470.31	2007
Secondary infertility	6873356.355	6854664.394	6892048.317	2008
Secondary infertility	7029704.067	7010792.034	7048616.101	2009
Secondary infertility	7180757.695	7161636.826	7199878.564	2010
Secondary infertility	7351497.82	7332144.274	7370851.367	2011
Secondary infertility	7547237.218	7527617.072	7566857.363	2012
Secondary infertility	7748895.046	7729006.745	7768783.346	2013
Secondary infertility	7934964.43	7914835.796	7955093.064	2014
Secondary infertility	8083965.471	8063652.103	8104278.839	2015
Secondary infertility	8228701.787	8208205.12	8249198.454	2016
Secondary infertility	8401022.497	8380300.895	8421744.099	2017
Secondary infertility	8578292.582	8557341.629	8599243.535	2018
Secondary infertility	8737570.932	8716421.769	8758720.095	2019
Secondary infertility	8936667.867	8915284.306	8958051.428	2020
Secondary infertility	9012770.156	8991304.402	9034235.909	2021

Secondary infertility	9217269.137	8698791.316	9735746.957	2022
Secondary infertility	9398413.339	8802482.858	9994343.82	2023
Secondary infertility	9585553.515	8876575.123	10294531.91	2024
Secondary infertility	9778499.577	8921458.742	10635540.41	2025
Secondary infertility	9977754.807	8939110.888	11016398.73	2026
Secondary infertility	10184651.73	8932124.641	11437178.81	2027
Secondary infertility	10401515.67	8904562.057	11898469.29	2028
Secondary infertility	10626113.62	8855229.917	12396997.32	2029
Secondary infertility	10854263.85	8780422.819	12928104.88	2030
Secondary infertility	11087392.53	8680477.02	13494308.04	2031
Secondary infertility	11328860.66	8556852.568	14100868.75	2032
Secondary infertility	11580766.42	8410600.242	14750932.6	2033
Secondary infertility	11842401.93	8239861.269	15444942.59	2034
Secondary infertility	12110139.54	8040243.637	16180035.45	2035
Secondary infertility	12392566.27	7815195.924	16969936.61	2036
Secondary infertility	12698451.7	7566868.033	17830035.36	2037
Secondary infertility	13023214.3	7288824.054	18757604.54	2038
Secondary infertility	13364331.93	6975888.576	19752775.28	2039
Secondary infertility	13719500.28	6622844.293	20816156.26	2040
Secondary infertility	14094110.86	6227907.825	21960313.89	2041
Secondary infertility	14495982.61	5789161.225	23202804	2042
Secondary infertility	14922213.29	5298758.274	24545668.3	2043
Secondary infertility	15370927.7	4749085.053	25992770.34	2044
Secondary infertility	15839609.71	4131970.912	27547248.5	2045
Secondary infertility	16330015.63	3440514.133	29220002.59	2046
Secondary infertility	16848304.25	2734053.462	31030574.17	2047

Secondary infertility	17394215.87	1962879.275	32989171.64	2048
Secondary infertility	17967658.18	1234555.637	35107400.53	2049
Secondary infertility	18568911.85	556892.5371	37398794.89	2050

Table S10. The results of BAPC model to predict the temporal trend in the number of ASPR from 1990 to 2050.

Type	pred_rate_val	pred_low	pred_up	year
Infertility	475.5376878	475.1642796	475.911096	1990
Infertility	478.2454046	477.8756397	478.6151695	1991
Infertility	480.8321527	480.4653715	481.1989339	1992
Infertility	483.4303574	483.0664433	483.7942715	1993
Infertility	486.4552357	486.0936492	486.8168221	1994
Infertility	489.427487	489.0682152	489.7867589	1995
Infertility	494.3605447	494.0024889	494.7186006	1996
Infertility	501.5553525	501.1975382	501.9131669	1997
Infertility	509.6006149	509.242775	509.9584548	1998
Infertility	517.108795	516.7510108	517.4665792	1999
Infertility	522.7883034	522.4312845	523.1453222	2000
Infertility	527.5237559	527.1677577	527.8797541	2001
Infertility	532.7632482	532.4081905	533.1183059	2002
Infertility	537.7960384	537.4420712	538.1500056	2003
Infertility	542.1938073	541.8411435	542.5464711	2004
Infertility	545.4549687	545.1039031	545.8060342	2005
Infertility	548.1391758	547.789783	548.4885686	2006
Infertility	551.1727833	550.8248096	551.520757	2007
Infertility	554.7174045	554.3705641	555.0642449	2008
Infertility	558.6536158	558.3076823	558.9995493	2009
Infertility	562.6308223	562.2855766	562.976068	2010
Infertility	567.8058468	567.460713	568.1509806	2011
Infertility	574.3394894	573.9938887	574.68509	2012
Infertility	581.4457194	581.0993343	581.7921046	2013

Infertility	588.1420887	587.7949362	588.4892411	2014
Infertility	593.5376054	593.1899982	593.8852125	2015
Infertility	600.0002272	599.6517952	600.3486593	2016
Infertility	609.0533315	608.7032808	609.4033822	2017
Infertility	618.5254064	618.1736109	618.877202	2018
Infertility	626.2322554	625.8792585	626.5852522	2019
Infertility	635.8340319	635.4793154	636.1887484	2020
Infertility	638.1542311	637.7997893	638.5086728	2021
Infertility	651.2933337	637.4263987	665.1602688	2022
Infertility	658.703143	638.3187146	679.0875713	2023
Infertility	666.2558872	637.4211835	695.0905908	2024
Infertility	673.9698898	635.1354341	712.8043455	2025
Infertility	681.9113077	631.6644472	732.1581683	2026
Infertility	690.2343808	627.1885261	753.2802356	2027
Infertility	698.7875638	621.6673925	775.907735	2028
Infertility	707.4862011	615.0687983	799.9036038	2029
Infertility	716.3368941	607.3965667	825.2772216	2030
Infertility	725.3806306	598.6427353	852.1185259	2031
Infertility	734.7490055	588.8581407	880.6398703	2032
Infertility	744.3177379	577.9533058	910.68217	2033
Infertility	754.0270811	565.8745476	942.1796147	2034
Infertility	763.8901047	552.6004908	975.1797186	2035
Infertility	773.9350052	538.0934724	1009.776538	2036
Infertility	784.2849267	522.3711273	1046.198726	2037
Infertility	794.8816387	505.3718245	1084.391453	2038
Infertility	805.7016875	487.0462885	1124.357086	2039

Infertility	816.7633179	467.3541301	1166.172506	2040
Infertility	828.0753917	446.2291772	1209.921606	2041
Infertility	839.7276975	423.6433669	1255.812028	2042
Infertility	851.6790824	399.5276966	1303.830468	2043
Infertility	863.885677	373.8060416	1353.965313	2044
Infertility	876.3224301	346.4038982	1406.240962	2045
Infertility	888.9564992	317.2357281	1460.67727	2046
Infertility	901.837228	286.2519676	1517.422488	2047
Infertility	914.950507	253.4014263	1576.499588	2048
Infertility	928.277422	218.6291308	1637.925713	2049
Infertility	941.8038524	181.8797363	1701.727969	2050
Primary infertility	148.677636	148.4740876	148.8811844	1990
Primary infertility	150.3794658	150.1768046	150.5821269	1991
Primary infertility	151.9331978	151.7313141	152.1350814	1992
Primary infertility	153.2452736	153.0442478	153.4462994	1993
Primary infertility	154.46597	154.2656837	154.6662563	1994
Primary infertility	155.4621028	155.2626484	155.6615571	1995
Primary infertility	156.5257286	156.3269214	156.7245358	1996
Primary infertility	157.5596493	157.3614338	157.7578649	1997
Primary infertility	158.5486363	158.3510424	158.7462301	1998
Primary infertility	159.5058853	159.3089158	159.7028548	1999
Primary infertility	160.4202356	160.2239573	160.6165138	2000
Primary infertility	161.579516	161.3837774	161.7752546	2001
Primary infertility	162.9390986	162.7438299	163.1343672	2002
Primary infertility	164.1738937	163.9792416	164.3685459	2003
Primary infertility	165.0335897	164.8398338	165.2273455	2004

Primary infertility	165.2866189	165.0941354	165.4791024	2005
Primary infertility	165.1272732	164.9362764	165.3182701	2006
Primary infertility	164.9475923	164.7580183	165.1371664	2007
Primary infertility	164.8791253	164.6908208	165.0674299	2008
Primary infertility	164.9371562	164.7499446	165.1243678	2009
Primary infertility	165.0618679	164.8755403	165.2481955	2010
Primary infertility	165.01156	164.8260528	165.1970672	2011
Primary infertility	164.716337	164.5316713	164.9010027	2012
Primary infertility	164.4577096	164.2737491	164.6416701	2013
Primary infertility	164.4864039	164.3028814	164.6699263	2014
Primary infertility	165.1172023	164.9336921	165.3007124	2015
Primary infertility	166.9960147	166.8117612	167.1802683	2016
Primary infertility	170.0023183	169.8166731	170.1879636	2017
Primary infertility	173.2480589	173.0608896	173.4352282	2018
Primary infertility	175.7996462	175.61136	175.9879324	2019
Primary infertility	178.2239731	178.0346675	178.4132786	2020
Primary infertility	179.8446066	179.6548038	180.0344094	2021
Primary infertility	183.0483907	174.2382507	191.8585307	2022
Primary infertility	185.0312025	174.2484318	195.8139733	2023
Primary infertility	186.9946724	173.5710098	200.4183349	2024
Primary infertility	188.9494973	172.2966074	205.6023872	2025
Primary infertility	190.9078515	170.4950691	211.3206338	2026
Primary infertility	192.8808416	168.2146749	217.5470083	2027
Primary infertility	194.8616858	165.4875487	224.2358229	2028
Primary infertility	196.8461032	162.3288491	231.3633573	2029
Primary infertility	198.8353711	158.7458027	238.9249394	2030

Primary infertility	200.8312233	154.7382363	246.9242103	2031
Primary infertility	202.8369472	150.3058569	255.3680375	2032
Primary infertility	204.8425841	145.4478878	264.2372803	2033
Primary infertility	206.841296	140.1608962	273.5216958	2034
Primary infertility	208.831117	134.4411225	283.2211115	2035
Primary infertility	210.811717	128.283362	293.3400719	2036
Primary infertility	212.7877353	121.6858875	303.8895831	2037
Primary infertility	214.7606932	114.6506343	314.8707522	2038
Primary infertility	216.7348407	107.1779207	326.2917608	2039
Primary infertility	218.7169604	99.26656307	338.1673578	2040
Primary infertility	220.712807	90.91250153	350.5131125	2041
Primary infertility	222.7283773	82.11129643	363.3454582	2042
Primary infertility	224.7654537	72.85948288	376.6714246	2043
Primary infertility	226.8237966	63.15071948	390.4968737	2044
Primary infertility	228.9037588	52.97761729	404.8299004	2045
Primary infertility	231.0060397	42.33231861	419.6797607	2046
Primary infertility	233.1329891	31.20820007	435.0577782	2047
Primary infertility	235.2842234	19.60065251	450.9677943	2048
Primary infertility	237.4579959	7.504001434	467.4119903	2049
Primary infertility	239.6527018	-5.087736188	484.3931397	2050
Secondary infertility	326.8616956	326.5485386	327.1748526	1990
Secondary infertility	327.8662086	327.5566293	328.1757878	1991
Secondary infertility	328.8989535	328.5924444	329.2054625	1992
Secondary infertility	330.1847925	329.8811658	330.4884192	1993
Secondary infertility	331.9887916	331.6874771	332.2901061	1994
Secondary infertility	333.963477	333.6644	334.2625539	1995

Secondary infertility	337.8330789	337.5350406	338.1311173	1996
Secondary infertility	343.9956511	343.6975156	344.2937866	1997
Secondary infertility	351.0534788	350.7549086	351.352049	1998
Secondary infertility	357.6055579	357.3066509	357.904465	1999
Secondary infertility	362.3698894	362.0714522	362.6683266	2000
Secondary infertility	365.9446588	365.6470934	366.2422243	2001
Secondary infertility	369.8240448	369.5272995	370.1207902	2002
Secondary infertility	373.6216203	373.3257806	373.91746	2003
Secondary infertility	377.1597109	376.8648503	377.4545715	2004
Secondary infertility	380.1673229	379.8735468	380.4610991	2005
Secondary infertility	383.0104756	382.7177315	383.3032198	2006
Secondary infertility	386.2240845	385.932111	386.516058	2007
Secondary infertility	389.8378653	389.5464217	390.1293088	2008
Secondary infertility	393.7170582	393.4259905	394.0081258	2009
Secondary infertility	397.5705233	397.2797067	397.8613398	2010
Secondary infertility	402.7959506	402.5047455	403.0871556	2011
Secondary infertility	409.6242836	409.3319957	409.9165715	2012
Secondary infertility	416.9884064	416.6947508	417.282062	2013
Secondary infertility	423.6551536	423.3603213	423.9499859	2014
Secondary infertility	428.4180124	428.1226396	428.7133851	2015
Secondary infertility	433.0017054	432.7058266	433.2975842	2016
Secondary infertility	439.0508349	438.753921	439.3477488	2017
Secondary infertility	445.2788268	444.9808178	445.5768357	2018
Secondary infertility	450.4336979	450.1349782	450.7324175	2019
Secondary infertility	457.6129997	457.3128865	457.9131128	2020
Secondary infertility	458.3064364	458.0070575	458.6058153	2021

Secondary infertility	468.6284945	454.113622	483.143367	2022
Secondary infertility	474.3124434	454.5456472	494.0792395	2023
Secondary infertility	480.2774014	453.6025135	506.9522893	2024
Secondary infertility	486.5333954	451.5811045	521.4856862	2025
Secondary infertility	493.1295682	448.6628825	537.5962539	2026
Secondary infertility	500.1417708	444.9724863	555.3110552	2027
Secondary infertility	507.49173	440.5045816	574.4788783	2028
Secondary infertility	515.1243785	435.2297006	595.0190563	2029
Secondary infertility	523.0397164	429.1326864	616.9467464	2030
Secondary infertility	531.2829415	422.209157	640.3567261	2031
Secondary infertility	539.9350497	414.4718971	665.3982024	2032
Secondary infertility	548.9324179	405.853519	692.0113168	2033
Secondary infertility	558.2361974	396.2906705	720.1817243	2034
Secondary infertility	567.8521962	385.7353253	749.9690672	2035
Secondary infertility	577.8158699	374.1420947	781.489645	2036
Secondary infertility	588.2024534	361.4831594	814.9217474	2037
Secondary infertility	598.9618519	347.6757703	850.2479335	2038
Secondary infertility	610.0523134	332.6344467	887.4701801	2039
Secondary infertility	621.4546993	316.2768295	926.6325691	2040
Secondary infertility	633.1610077	298.5166203	967.8053952	2041
Secondary infertility	645.2162925	279.2912405	1011.141344	2042
Secondary infertility	657.5925335	258.5225239	1056.662543	2043
Secondary infertility	670.2664587	236.1293724	1104.403545	2044
Secondary infertility	683.2211742	212.0273884	1154.41496	2045
Secondary infertility	696.4331203	186.1256532	1206.740587	2046
Secondary infertility	709.923903	158.3504522	1261.497354	2047

Secondary infertility	723.6912048	128.6356434	1318.746766	2048
Secondary infertility	737.7351894	96.91263391	1378.557745	2049
Secondary infertility	752.0568197	63.1096533	1441.003986	2050

Table S11. The results of decomposition analysis at the global and regional levels from 1990 to 2021.

location	sex	cause	rei	measure	Overll difference	Aging	Population	Epidemiological change
Global	Female	Polycystic ovarian syndrome	Infertility	Prevalence	6150002.67	58563.15 (0.95%)	3402776.34 (55.33%)	2688663.18 (43.72%)
High-middle SDI	Female	Polycystic ovarian syndrome	Infertility	Prevalence	816076.24	709.62 (0.09%)	147849.59 (18.12%)	667517.02 (81.8%)
High SDI	Female	Polycystic ovarian syndrome	Infertility	Prevalence	651849.9	-7913.54 (-1.21%)	175714.69 (26.96%)	484048.75 (74.26%)
Low-middle SDI	Female	Polycystic ovarian syndrome	Infertility	Prevalence	1548208.49	4304.4 (0.28%)	879583.19 (56.81%)	664320.91 (42.91%)
Low SDI	Female	Polycystic ovarian syndrome	Infertility	Prevalence	553254.01	-569.46 (-0.1%)	393002.83 (71.03%)	160820.64 (29.07%)
Middle SDI	Female	Polycystic ovarian syndrome	Infertility	Prevalence	2576284.38	-10501 (-0.41%)	994032.37 (38.58%)	1592753.01 (61.82%)
Global	Female	Polycystic ovarian syndrome	Primary infertility	Prevalence	1378722.2	-164186.67 (-11.91%)	1019170.32 (73.92%)	523738.54 (37.99%)

High-middle SDI	Female	Polycystic ovarian syndrome	Primary infertility	Prevalence	125287.87	-56972.89 (-45.47%)	37183.73 (29.68%)	145077.04 (115.79%)
High SDI	Female	Polycystic ovarian syndrome	Primary infertility	Prevalence	128736.61	-44644.54 (-34.68%)	59595.99 (46.29%)	113785.16 (88.39%)
Low-middle SDI	Female	Polycystic ovarian syndrome	Primary infertility	Prevalence	405679.35	-20637.2 (-5.09%)	291185.85 (71.78%)	135130.7 (33.31%)
Low SDI	Female	Polycystic ovarian syndrome	Primary infertility	Prevalence	146897.82	-914.4 (-0.62%)	115825.47 (78.85%)	31986.75 (21.77%)
Middle SDI	Female	Polycystic ovarian syndrome	Primary infertility	Prevalence	571095.59	-101179.18 (-17.72%)	275511.57 (48.24%)	396763.2 (69.47%)
Global	Female	Polycystic ovarian syndrome	Secondary infertility	Prevalence	4771280.48	222749.82 (4.67%)	2383606.01 (49.96%)	2164924.64 (45.37%)
High-middle SDI	Female	Polycystic ovarian syndrome	Secondary infertility	Prevalence	690788.37	57682.51 (8.35%)	110665.86 (16.02%)	522439.99 (75.63%)
High SDI	Female	Polycystic ovarian syndrome	Secondary infertility	Prevalence	523113.29	36731 (7.02%)	116118.7 (22.2%)	370263.6 (70.78%)

Low-middle SDI	Female	Polycystic ovarian syndrome	Secondary infertility	Prevalence	1142529.14	24941.6 (2.18%)	588397.34 (51.5%)	529190.2 (46.32%)
Low SDI	Female	Polycystic ovarian syndrome	Secondary infertility	Prevalence	406356.19	344.94 (0.08%)	277177.35 (68.21%)	128833.9 (31.7%)
Middle SDI	Female	Polycystic ovarian syndrome	Secondary infertility	Prevalence	2005188.8	90678.18 (4.52%)	718520.8 (35.83%)	1195989.81 (59.64%)

Table S12. The slope index of the cross-country inequality analysis.

Type	year	value	lower	upper	p
Infertility	1990	257.9504	199.6504	316.2504	<0.001
Infertility	2021	324.1287	242.934	405.3234	<0.001
Primary infertility	1990	257.9504	199.6504	316.2504	<0.001
Primary infertility	2021	324.1287	242.934	405.3234	<0.001
Secondary infertility	1990	257.9504	199.6504	316.2504	<0.001
Secondary infertility	2021	324.1287	242.934	405.3234	<0.001

Table S13. The concentration index of the cross-country inequality analysis.

type	year	Concentration_index	uci	lci	SE	P
Infertility	1990	0.24714656	0.298253776	0.196039344	0.02607511	<0.001
Infertility	2021	0.19094327	0.229965929	0.151920611	0.01990952	<0.001
Primary infertility	1990	0.3000209	0.363760022	0.236281778	0.03251996	<0.001
Primary infertility	2021	0.21957272	0.273661233	0.165484207	0.02759618	<0.001
Secondary infertility	1990	0.22245591	0.274122372	0.170789448	0.02636044	<0.001
Secondary infertility	2021	0.17937797	0.217098209	0.141657731	0.01924502	<0.001

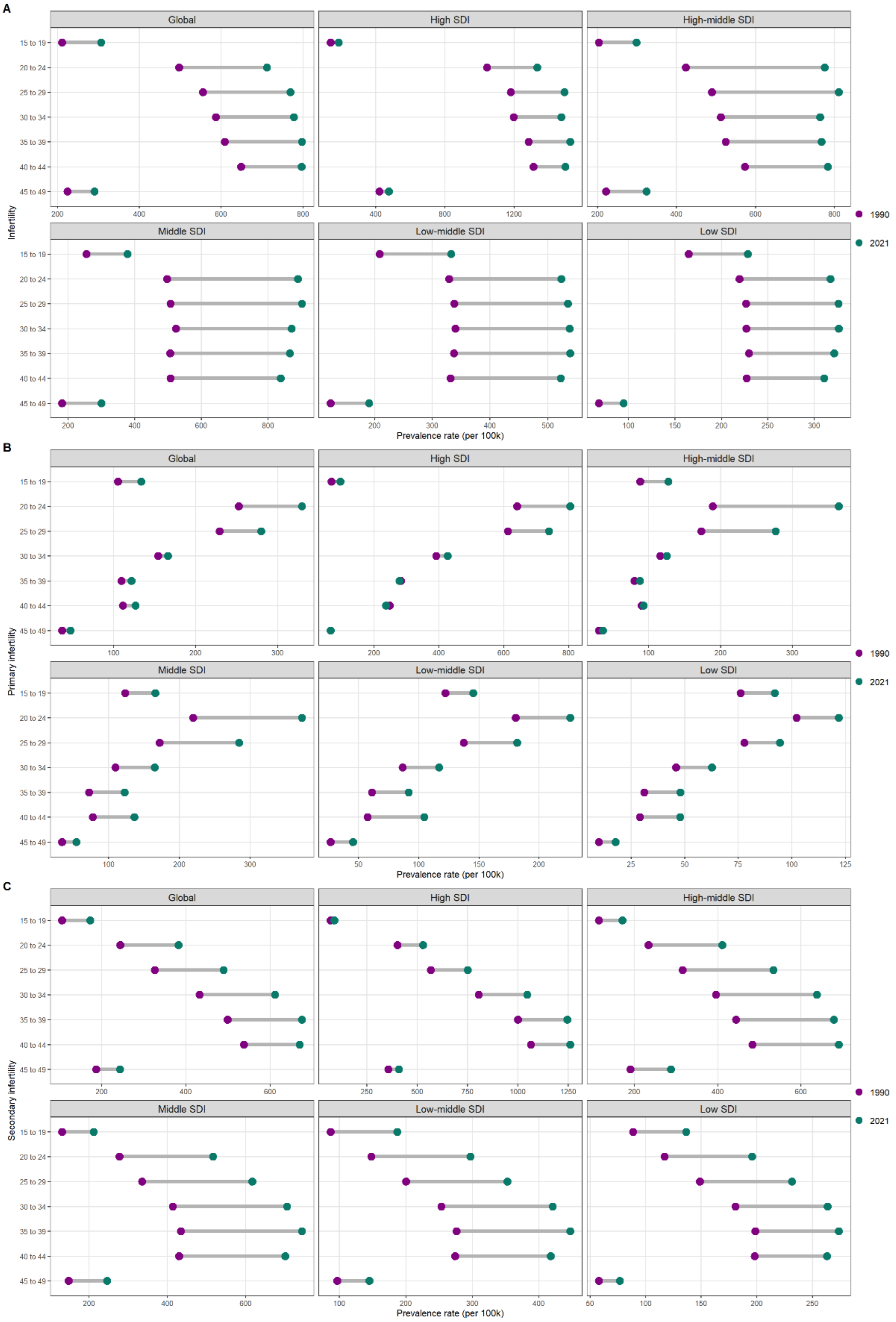

Figure S1 | ASPR of polycystic ovary syndrome-related infertility by age group (15-49 years) across SDI regions in 1990 versus 2021. (A) Infertility. (B) Primary infertility. (C) Secondary infertility.

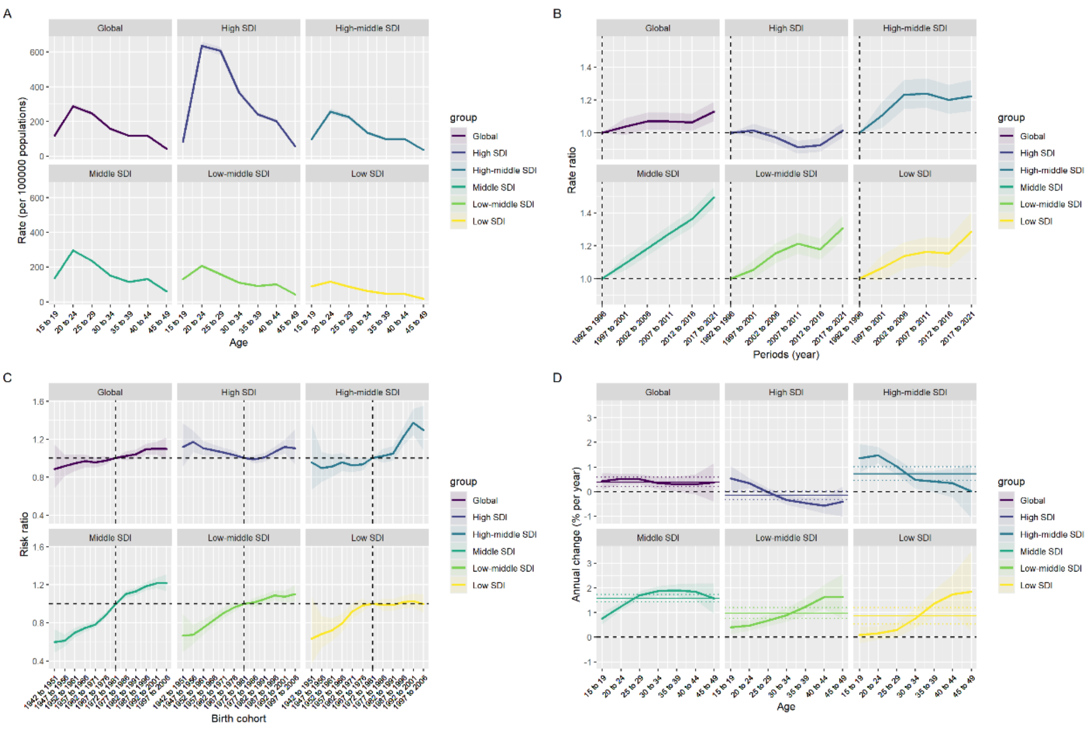

Figure S2 | Effects of age, period, and birth cohort on primary infertility-associated polycystic ovary syndrome prevalence across SDI quintiles. (A) Age effects are represented by fitted longitudinal age-specific rates. (B) Period effects are demonstrated through relative period risks. (C) Cohort effects are displayed using relative cohort risks. (D) Annual percentage changes are shown for each age-specific group through yearly variations.

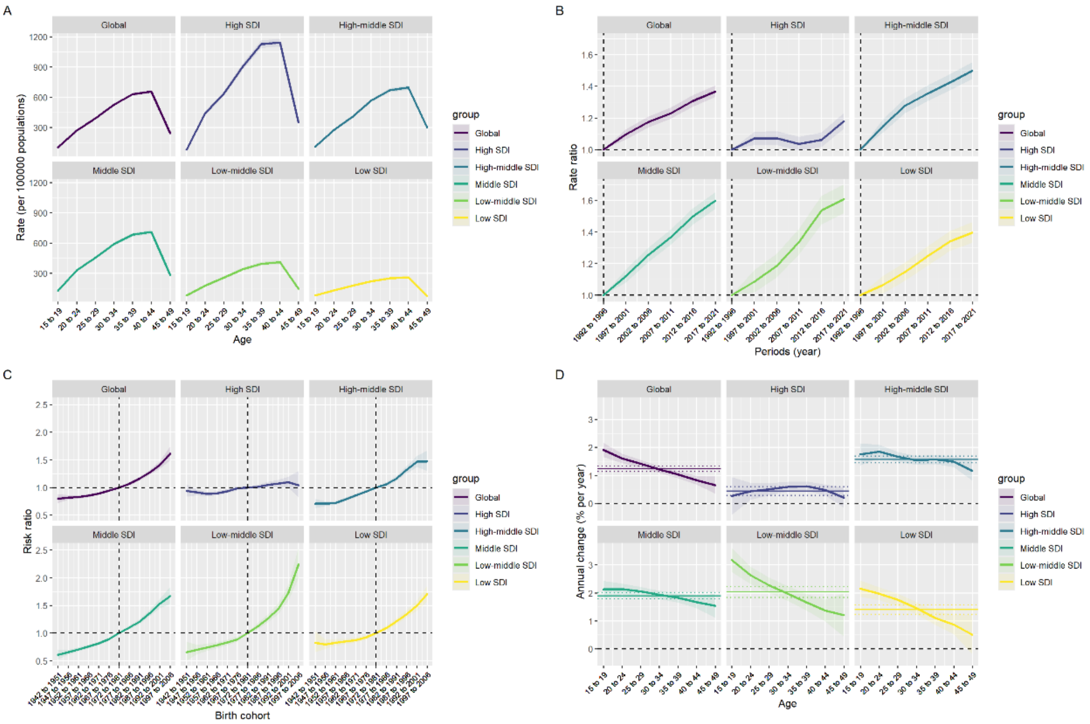

Figure S3 | Effects of age, period, and birth cohort on secondary infertility-associated polycystic ovary syndrome prevalence across SDI quintiles. (A) Age effects are represented by fitted longitudinal age-specific rates. (B) Period effects are demonstrated through relative period risks. (C) Cohort effects are displayed using relative cohort risks. (D) Annual percentage changes are shown for each age-specific group through yearly variation.
